# Whole-exon sequencing of human myeloma cell lines shows mutations related to myeloma patients at relapse with major hits in the DNA regulation and repair pathways

**DOI:** 10.1186/s13045-018-0679-0

**Published:** 2018-12-13

**Authors:** Benoît Tessoulin, Agnès Moreau-Aubry, Géraldine Descamps, Patricia Gomez-Bougie, Sophie Maïga, Alban Gaignard, David Chiron, Emmanuelle Ménoret, Steven Le Gouill, Philippe Moreau, Martine Amiot, Catherine Pellat-Deceunynck

**Affiliations:** 1 CRCINA, INSERM, CNRS, Université d’Angers, Université de Nantes, Nantes, France; 2 Service d’Hématologie Clinique, Unité d’Investigation Clinique, CHU, Nantes, France; 3 Bird Platform, Inserm 1087, Nantes, France; 4 Myelomax SAS, Nantes, France

**Keywords:** Myelomas, WES, Tumor suppressors, DNA repair, Fanconi pathway

## Abstract

**Background:**

Human myeloma cell lines (HMCLs) are widely used for their representation of primary myeloma cells because they cover patient diversity, although not fully. Their genetic background is mostly undiscovered, and no comprehensive study has ever been conducted in order to reveal those details.

**Methods:**

We performed whole-exon sequencing of 33 HMCLs, which were established over the last 50 years in 12 laboratories. Gene expression profiling and drug testing for the 33 HMCLs are also provided and correlated to exon-sequencing findings.

**Results:**

Missense mutations were the most frequent hits in genes (92%). HMCLs harbored between 307 and 916 mutations per sample, with *TP53* being the most mutated gene (67%). Recurrent bi-allelic losses were found in genes involved in cell cycle regulation (*RB1*, *CDKN2C*), the NFκB pathway (*TRAF3*, *BIRC2*), and the p53 pathway (*TP53*, *CDKN2A*). Frequency of mutations/deletions in HMCLs were either similar to that of patients (e.g., *DIS3*, *PRDM1*, *KRAS)* or highly increased (e.g., *TP53*, *CDKN2C*, *NRAS*, *PRKD2)*. MAPK was the most altered pathway (82% of HMCLs), mainly by *RAS* mutants. Surprisingly, HMCLs displayed alterations in epigenetic (73%) and Fanconi anemia (54%) and few alterations in apoptotic machinery. We further identified mutually exclusive and associated mutations/deletions in genes involved in the MAPK and p53 pathways as well as in chromatin regulator/modifier genes. Finally, by combining the gene expression profile, gene mutation, gene deletion, and drug response, we demonstrated that several targeted drugs overcome or bypass some mutations.

**Conclusions:**

With this work, we retrieved genomic alterations of HMCLs, highlighting that they display numerous and unprecedented abnormalities, especially in DNA regulation and repair pathways. Furthermore, we demonstrate that HMCLs are a reliable model for drug screening for refractory patients at diagnosis or at relapse.

**Electronic supplementary material:**

The online version of this article (10.1186/s13045-018-0679-0) contains supplementary material, which is available to authorized users.

## Background

Human myeloma cell lines (HMCLs) are widely used for their representation of primary myeloma cells because they cover patient diversity, although not fully [1]. HMCLs are mainly derived from refractory patients, mostly presenting with extramedullary disease and having thus received numerous classes of drugs inducing DNA damage, proteasome inhibition, immunomodulation, and anti-inflammation (e.g., melphalan, bendamustine, Velcade, Revlimid, and dexamethasone). However, HMCLs harbor the 14q32 abnormality, which occurs early at the MGUS stage, and display frequent mutations in *NRAS* and *KRAS*, as observed in patients at diagnosis (approximately 50% of patients) [2, 3]. By contrast, HMCLs display very frequent deletion and mutation in the *TP53* gene that are associated with resistance to treatments [4]. Indeed, it is well known that hits in the *TP53* gene (deletion and/or mutation) at diagnosis are associated with resistance and shortened survival and that their frequency increases with relapse [4, 5]. Thus, HMCLs are a mixture of abnormalities occurring both early and late in the time course of disease. Besides hits in the *TP53* and *RAS* genes, HMCLs have not been widely characterized for their global mutation profile and gene deletion. In the present work, using whole-exon sequencing (WES) in 33 HMCLs, we report common gene mutations and deletions. We analyzed the frequency of mutations/deletions in comparison with patients at diagnosis and relapse. We further identified hits preferentially associated with 14q32 translocations and analyzed responses to conventional and nonconventional drugs in relation to a mutation and/or deletion profile.

## Methods

### HMCLs and primary MM cells

HMCLs were previously characterized [1, 6, 7]. HMCLs were cultured in RPMI-5% fetal calf serum with or without 3 ng/ml of IL6 [1, 6, 7]. Gene expression profile of HMCLs has been previously published [1]. The gene expression profile of primary MM cells was assessed from 414 patients (Arkansas) as previously described [1, 8].

### Whole-exon sequencing

DNA sample processing was performed according to Agilent Technologies (Santa Clara, CA, USA) using the sureselect target enrichment system kit (Human all exon v6, library version 1.6). Sequencing was performed on HiSeq 2500, High Output in paired-end 2 × 100 bp. The reads were aligned (BWA-v0.7.10-r789) to the GRCh37 human reference genome. Duplicated reads were marked by the Picard tool (v1.119), indels were realigned around capture (± 500 bp), and base quality recalibration was finally performed (Genome Analysis Toolkit [GATK-v3.2.2]). In the absence of germline DNA, variants were called by the GATK unified genotyper. Variants were processed through vcf2maf-1.6.15 to obtain a final Mutant Annotation File (maf). The variants’ biological effects predictions were carried out using Ensembl’s VEP-annotator-v.86. Variant annotation database versions were as follows: ExAC-r0.3.1 [likely germline variants], dbSNP-v.144 [known variants], COSMIC-71 and ClinVar-v.201507 [clinical significance of known variants].

Variants that were present more than three times were removed, as well as variants with Global Allele Frequency in ExAC databases over 1% (with respect to ethnicity frequencies when known). Finally, clinically benign mutants, as annotated by ClinVar, were removed (“benign” or “likely benign”). Only protein-coding variants were used for subsequent analyses, and structural protein coding genes (actin, myosin, collagen, fibronectin, vitronectin, tenascin, laminin, titin, obscurin, plectin, aggrecan, and mucins) were removed.

Exon loss was estimated from the read depth using ExomeCOPY and CANOES. The results were validated by visual inspection of the BAM read depth in Integrative Genomics Viewer (IGV; Broad Institute). Genes with frequent variants were selected and were assessed by direct Sanger sequencing on cDNA.

### Functional assays

The cell count and viability were measured using the MTT assay. The cell cycle distribution was assessed by propidium iodide incorporation. Rb phosphorylation was assessed by western blotting (Cell Signaling; 4H1 and S807-811). The area under the curve (AUC) was estimated using Graphpad Prism v7.0 for palbociclib (0–1 μM), CX5461 (0–1 μM), and trametinib (0–25 nM). The responses to melphalan, bendamustine, FAS and TRAIL-R agonist antibodies, PRIMA-1^Met^, dexamethasone, RITA, ABT-737, and ABT-199 were previously reported [6, 9–14]. Results were scaled (mean-centered and standardized) to provide a *z*-score.

### Statistical analyses

Analyses were performed under R 3.4.4. Fisher’s test was carried out with the resampling of parameters for robustness. The somatic interaction plot code was adapted from Gerstung et al. [15]. Enrichment analyses were carried out by ReactomePA and clusterProfiler [16, 17], *p* values were adjusted for multiple testing by the false discovery rate (*q* = 0.05). For the Reactome determination, KEGG and GO annotations were used. MAF manipulation was performed using the maftools packages [18]. Oncoprints, heatmaps, and Chord-Diagrams were performed with ComplexHeatmap R-package. Considering the number of samples, the linear regressions between scores and drug responses were calculated by robust a linear regression using a M-estimator (rlm, MASS package) in order to discard outliers. Coefficients were further bootstrapped by Boot function (car package), with 5000 replicates (seed = 22,062,016) and considered significant if the 95% confidence interval (95% CI) did not overlap with zero, only β1 coefficients are presented in the text.

## Results

### Metrics and variant filtering

WES was performed in 33 HMCLs of European, American or Asian origin, 19 having been derived in the presence of exogenous IL6 (Additional file 1: Table S1). After global SNP enrichment analysis on 609,585 bi-allelic SNPs (SNPRelate package [19]), three groups of HMCLs were identified: a group gathering HMCLs of Pacific/Japanese origin (AMO-1, KMM1, KMS12PE, KMS11, NAN8, OPM2) and a cluster encompassing all other HMCLs except MM1S, which was individualized as African ethnicity (Additional file 1: Figure S1). To remove ethnic-related SNPs, HMCLs were filtered with Global Allele Frequencies, plus East Asian frequencies for the Pacific/Japanese cluster and African frequencies for MM1S. Because of the lack of normal DNA from patients from whom the HMCLs were derived, we could not easily discriminate the constitutive SNPs from the tumor-associated mutations. Thus, we excluded variants shared by more than 3 HMCLs of the 33: indeed, the most mutated genes in HMCLs and myeloma patients [20], i.e., *RAS* and *TP53*, never displayed more than three identical variants across the HMCL collection. For *NRAS*, the most frequent variant was c.38G-A (Gly12Asp) in JJN-3, Karpas620, and Nan7, while the only *TP53* shared variant was 406G-A (Karpas620, XG11). Variant effect predictions were carried out as described in the “Methods” section. We further removed variants of genes uniformly low expressed across the collection (maximum of the considered gene inferior to the first quartile mean expression of the microarray). After filtering, we retained 15,602 variants, spanning over 7641 genes (Maf file, Additional file 2). Most mutated samples were KMM1 and KMS12PE with 916 and 755 variants, respectively. The most frequent variant was missense (*n* = 14,309; 92%), while frameshifts occurred in 273 variants (1.7%), insertions or deletions without frameshifts occurred in 226 cases (1.4%), and 482 variants (3.1%) were nonsense mutations (Additional file 1: Figure S2). Single mutations were mainly C > T transitions (63%, Additional file 1: Figure S3), corresponding to spontaneous deamination of 5-methyl cytosine. HMCLs age was not associated with a particular mutation (Fisher test, FDR > 0.05), but younger cell lines displayed a lower mutation load (*β* = 4.29, 95% CI = [1.07; 9.47]). Mutations were confirmed in 18 genes by direct sequencing of RT–PCR products as previously reported for *RAS* and *TP53* [1, 9] (Additional file 1: Table S1). Although amplification of genes was not assessed because of the high number of chromosome abnormalities across the HMCL collection, exon losses were reported as described in the “Methods” section. Main variants are presented in Lollipop Plots (Additional file 3).

### HMCLs display alterations similar to those in MM cells

**Fig. 1:**
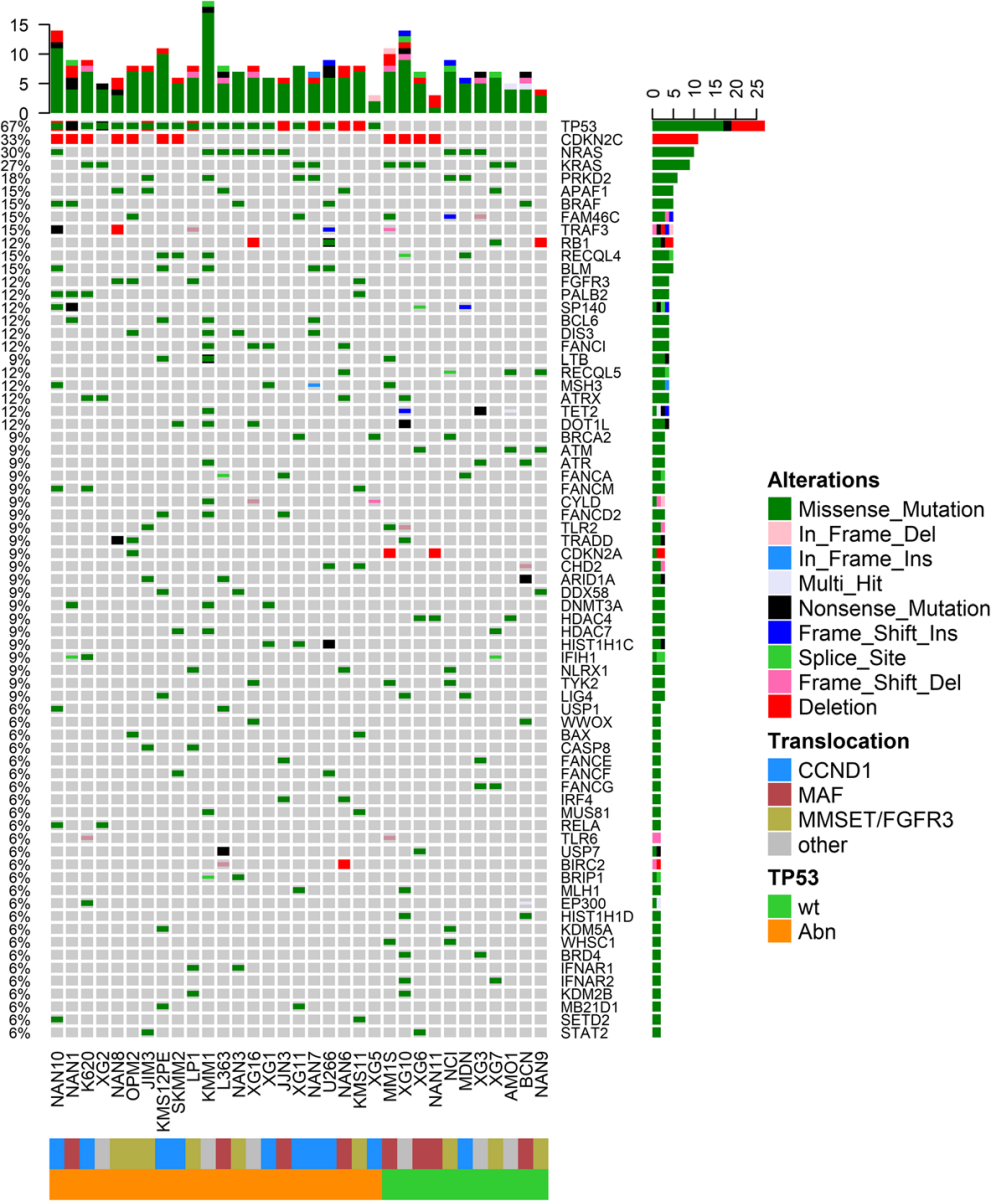
Oncoprint of the most frequently mutated and/or deleted genes in human myeloma cell lines. HMCLs were ranked according to the most frequent abnormalities. Several events affecting the same cell line (mutations and deletion) were represented in the same slot. The number of cumulative events per HMCL is indicated on the top of the graph

Figure 1 shows the most frequently altered genes across the collection and recurrent in MM [20–22]. Residues modified by mutants are provided in Additional file 1: Figure S4. Five genes, i.e., *TP53*, *KRAS, NRAS, CDKN2C* and *PRKD2* were altered in at least 21% and up to 67% of HMCLs.

**Fig. 2:**
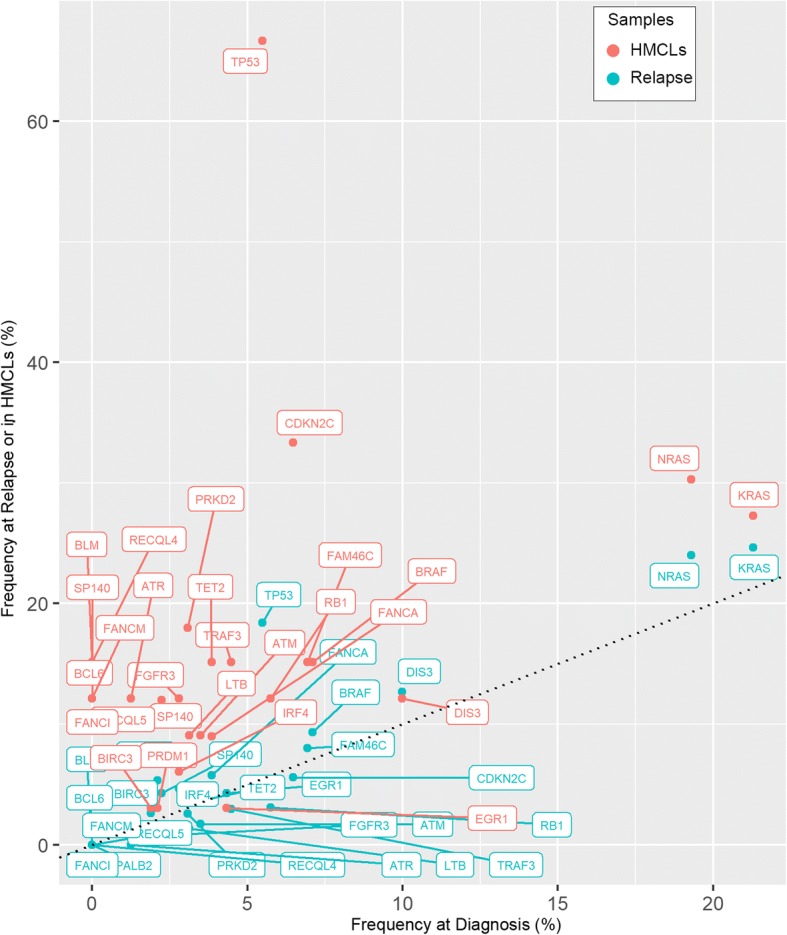
Comparison of the gene mutation/deletion frequency in human myeloma cell lines with multiple myeloma patients at diagnosis and relapse. The frequency of mutation/deletion at diagnosis (*x*-axis) was plotted against that at relapse (*y*-axis, blue). HMCL hit frequencies are represented in red dots. The dashed line represents the theoretical identical ratio between diagnosis and relapse

HMCLs shared a similar mutations rate with MM patients, either at diagnosis (DMM) or relapse (RMM) for *DIS3 (*12% in HMCLs, 10% in DMM and 13% in RMM), *PRDM1* (3%, 2%, and 5%, respectively), *BIRC3* (3%, 2%, and 3%, respectively), and *EGR1* (3%, 4%, and 4%, respectively) (Fig. 2). Of note, these very similar rates among HMCLs, DMM and RMM were in favor of early pathogenic mutations, poorly affected by subsequent treatment selection or cell culture. *KRAS* mutation rates were roughly shared between HMCLs, DMM, and RMM (21%, 24.7%, and 27%, respectively). By contrast, the *NRAS* mutation rate increased from DMM (19%) to RMM (24%) and HMCLs (30%). Similarly, *TP53* (67%), *CDKN2C* (33%), *PRKD2* (18%), *FAM46C* (15%), and *BRAF* (15%) mutation rates displayed a dramatically increased frequency in HMCLs compared with those in primary myeloma cells, either in DMM or RMM [2, 20–24] (Fig. 2). These high frequencies in the *FAM46C*, *TP53*, *BRAF*, and *NRAS* rates might be in line with either successive relapses and/or secondary plasma cell leukemia (PCL), from which HMCLs are mostly derived [25–29].

WES revealed that HMCLs displayed frequent mutations in Fanconi anemia genes (*PALB2* [12%], *FANCI* [12%], *FANCA* [9%], *FANCD2* [9%], *BRCA2* [9%]) as well as in helicases (such as *RECQL4*, 15%, and BLM, 15%) and epigenetic modifiers (e.g., *TET2*, 15% and *SETD2*, 6%). FANC family genes were recently reported to be mutated mostly in patients at relapse [24, 30, 31], suggesting that these mutations did not occur in vitro in continuously replicating cells but in vivo. Mutations in epigenetic modifiers were recently described as being more frequent at relapse [20, 22, 24, 32], such as histone methyl-transferases (6.9% vs 17%, in DMM and RMM, respectively) and DNA methylation modifiers (1.9% vs 8.3*%,* respectively*)*. On the other hand, genes involved in apoptotic pathways (extrinsic, intrinsic, execution) displayed few mutations or deletions, showing that cell death resistance was not associated with major defects in the apoptotic machinery.

These data collectively showed that HMCLs displayed mutations/deletions related to myeloma cells from patients at diagnosis (*KRAS*, *DIS3*, *EGR1*, *PRDM1* and *BIRC3*) and relapse/progression to PCL (*TP53*, *NRAS*, *BRAF*, *FANC* genes).

We further analyzed mutually exclusive and co-occurring mutations/deletions, as well as their associations with IgH translocation (Additional file 1: Figure S5). Abnormalities in the Ras/MAPK pathway were mutually exclusive: *KRAS*, *NRAS*, *HRAS*, *FGFR3* (and to a lesser extent *BRAF*) displayed mutually exclusive mutations (*p* < 0.05), and *FGFR3* mutations were exclusively found in t(4;14) HMCLs overexpressing *FGFR3* (*p* = 0.003, Additional file 1: Figure S5). *TP53* hits were mutually exclusive to mutations in *ATM* (*p* = 0.03), as previously reported in all B cell malignancies [33]. By contrast, several co-occurring mutations were found in DNA damage/repair/epigenetic modifiers for instance, in *BLM* and *FANCD2*, *RECQL5*, and *ATM*, *HDAC7*, and *DOT1L* (*p* < 0.05). *CDKN2A* deletion was found in HMCLs with *CDKN2C* mutations/deletions. *RECQL4* and *BLM* mutations were significantly associated with t(11;14), *p* = 0.03 and *p* = 0.002, respectively.

To provide a comprehensive landscape of mutations/deletions, we next performed global analysis of altered pathways based on the whole data of the mutated genes.

### HMCLs harbor the signatures of dysregulation in Rho GTPase, the cell cycle, and DNA replication

Gene Ontology (GO) enrichment analysis showed that most of the dysregulated biological processes were related to Rho GTPase signal transduction, Cell cycle/DNA replication and DNA damage (check-points before replication, DNA repair, DNA unwinding) (Additional file 1: Figure S6A). GO molecular functions such as helicase activity, nuclease activity, and Rho GTPase activity were also highly enriched (Additional file 1: Figure S6B-C).

Reactome Pathway Enrichment analysis revealed oncogenic MAPK signaling. After relaxing the *q* value at 0.1, pathways involved in DNA repair, p53 regulation of activity, and DNA double helix-modifying pathways were highlighted, as well as defects in the SUMOylation of DNA replication proteins, DNA damage response and repair proteins, cell cycle regulation by p53, resolution of D-loop structures through Holliday junction intermediates, and DNA repair (Additional file 1: Figure S6D).

Among cancer-associated pathways, the main KEGG-enriched pathways were the Fanconi anemia pathway (*q* value = 0.006), cell cycle (*q* = 0.01), prostate cancer (*q* = 0.02), chronic myeloid leukemia (*q* = 0.03), non-small cell lung cancer (*q* = 0.02), bladder cancer (*q* value = 0.02), and hepatocellular carcinoma (*q* = 0.02) (Fig. 6e). Non-homologous end joining (*q* = 0.04), platinum resistance (*q* = 0.04), base-excision-repair (*q* = 0.04), and mismatch-repair (*q* = 0.04) were also enriched in KEGG pathways. The prostate cancer, bladder cancer, non-small lung cancer, and non-cancer-related pathways revealed by KEGG enrichment analysis were mostly due to the high *RAS/BRAF* mutation rates. The hepatocellular carcinoma signature was also enriched by Wnt signaling mutations, while breast cancer signature displayed Notch, Wnt, and PI3K altered signaling.

Additional file 1: Figure S7 summarizes hits in the most dysregulated pathways. While pathway dysregulations were globally well balanced among the recurrent translocation subgroups, genes encoding helicases were more frequently encountered in t(11;14) cell lines (*p* = 0.006). On the other hand, intrinsic apoptosis mutants were more frequent in t(4;14), 66% vs 12% in non t(4;14) cell lines (*p* = 0.004).

### The extrinsic, intrinsic, and executive pathways of apoptosis are mostly unaltered in HMCLs

**Fig. 3:**
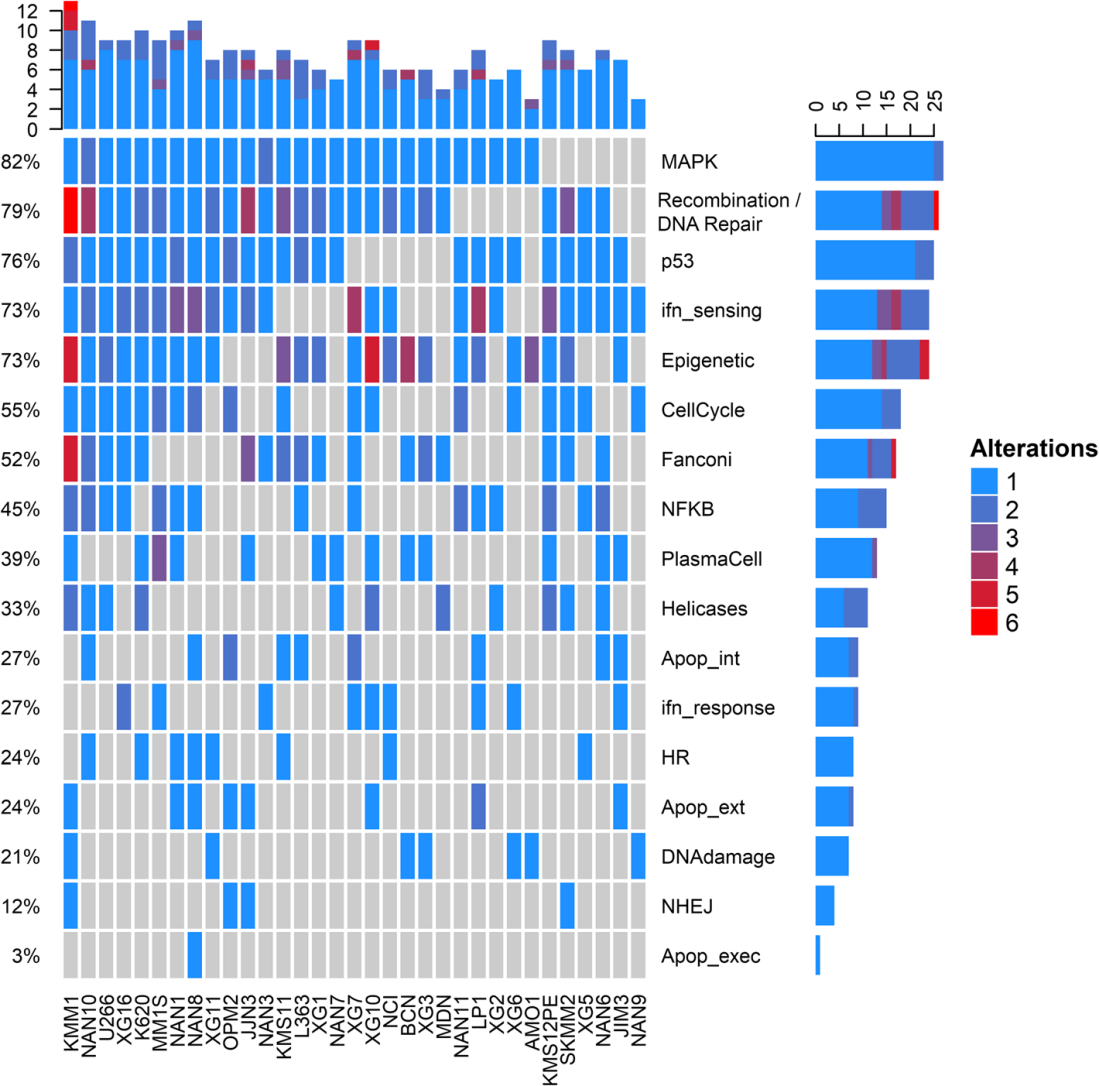
Oncoprint of altered pathways in human myeloma cell lines. Oncoprint of frequently altered pathways was performed as described in the “Methods” section. Oncoprint was performed with ComplexHeatmap R-package

Thirteen HMCLs displayed one or several mutations in the apoptotic pathway (extrinsic, intrinsic, and executive), which were heterozygous (except in LP1 that displayed a bi-allelic *BCL2L11/BIM* deletion) (Fig. 3). To assess the impact of mutations, we analyzed the cell death response through either the extrinsic, i.e., response to Fas/Trail-R agonist receptors (CH11, mapatumumab, or lexatumumab [9]) or the intrinsic pathway of apoptosis, i.e., response to BH3-mimetics (ABT-199, A-1210477, or A-1155463) [10, 11, 34, 35] (Additional file 1: Table S2). Pathway hit scores were calculated according to the number of hits in each pathway. No correlation could be drawn between the sensitivity to Trail-R agonists or Fas ligands and extrinsic apoptosis hits. Similarly, ABT-199/-737 responses did not correlate with intrinsic apoptosis hits. Moreover, intrinsic pathway hits were not associated with BH3-profiling, i.e., cytochrome C release in response to BIM peptide (Additional file 1: Table S2), confirming that the heterozygous mutations did not affect the upstream apoptotic responses (Additional file 1: Figure S8).

### HMCLs with *RAS* mutation are highly sensitive to trametinib

**Fig. 4:**
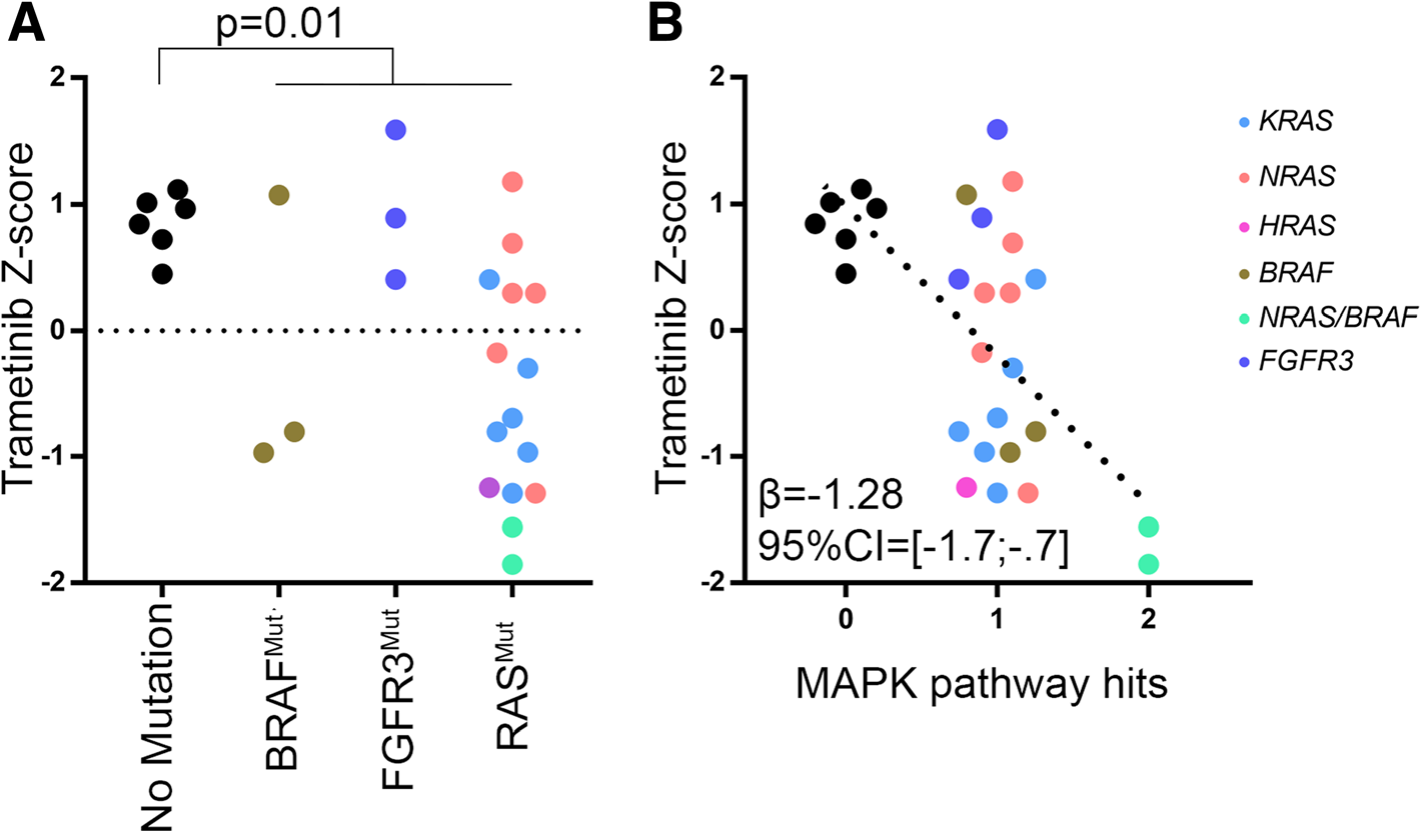
The sensitivity to trametinib is associated with *RAS* mutation. **a** Cells were cultured for 4 days with increasing concentrations of trametinib, and the sensitivity was determined by the area under the curve using the MTT assay and expressed as *z*-score. Analysis was performed as a function of mutations in the MAPK pathway (Mann–Whitney test). **b** Trametinib response associated with dysregulation in the MAPK pathway. Robust linear regression is displayed; regression line was drawn according to coefficients obtained after 5000 bootstrapped replicates. Points were jittered for clarity

Eighty-two percent of HMCLs displayed at least one variant of the MAPK pathway, with 60% of HMCLs bearing a *K-/H-/N-RAS* variant (Fig. 3 and Additional file 1: Figure S9). *FGFR3* mutations were present in 12% of HMCLs but in 50% of t(4;14) HMCLs with *FGFR3* overexpression (KMS11, LP1, OMP2). Five HMCLs expressed a *BRAF* mutation with BCN displaying six non-silent mutations in the PKc-like domain (without evidence of a frameshift). *BRAF* mutations mostly occurred in the PKc-like domain (four of five mutants), outside of the V600 codon. Two samples displayed both *NRAS* and *BRAF* mutations (NAN10 and NAN3). As shown in Fig. 4a, *RAS*-mutated HMCLs displayed hypersensitivity to the MEK-1/2 inhibitor trametinib (Mann–Whitney test, *p* = 0.002), while the three *FGFR3-*mutated HMCLs did not display significant sensitivity. Two HMCLs with *BRAF* mutations (without concomitant *RAS* mutation) out of three displayed hypersensitivity to trametinib. Of note, the two *BRAF*^*mut*^ HMCLs displaying high sensitivity to trametinib had variants around V600. Finally, the MAPK pathway hit score was associated with an increased sensitivity to trametinib (*β*_1_ = − 1.17, 95% CI = [− 1.41; − 0.61]), which was mainly related to *RAS* mutations (Fig. 4b).

### Abnormalities in cell cycle genes do not favor the response to CDK inhibition

**Fig. 5:**
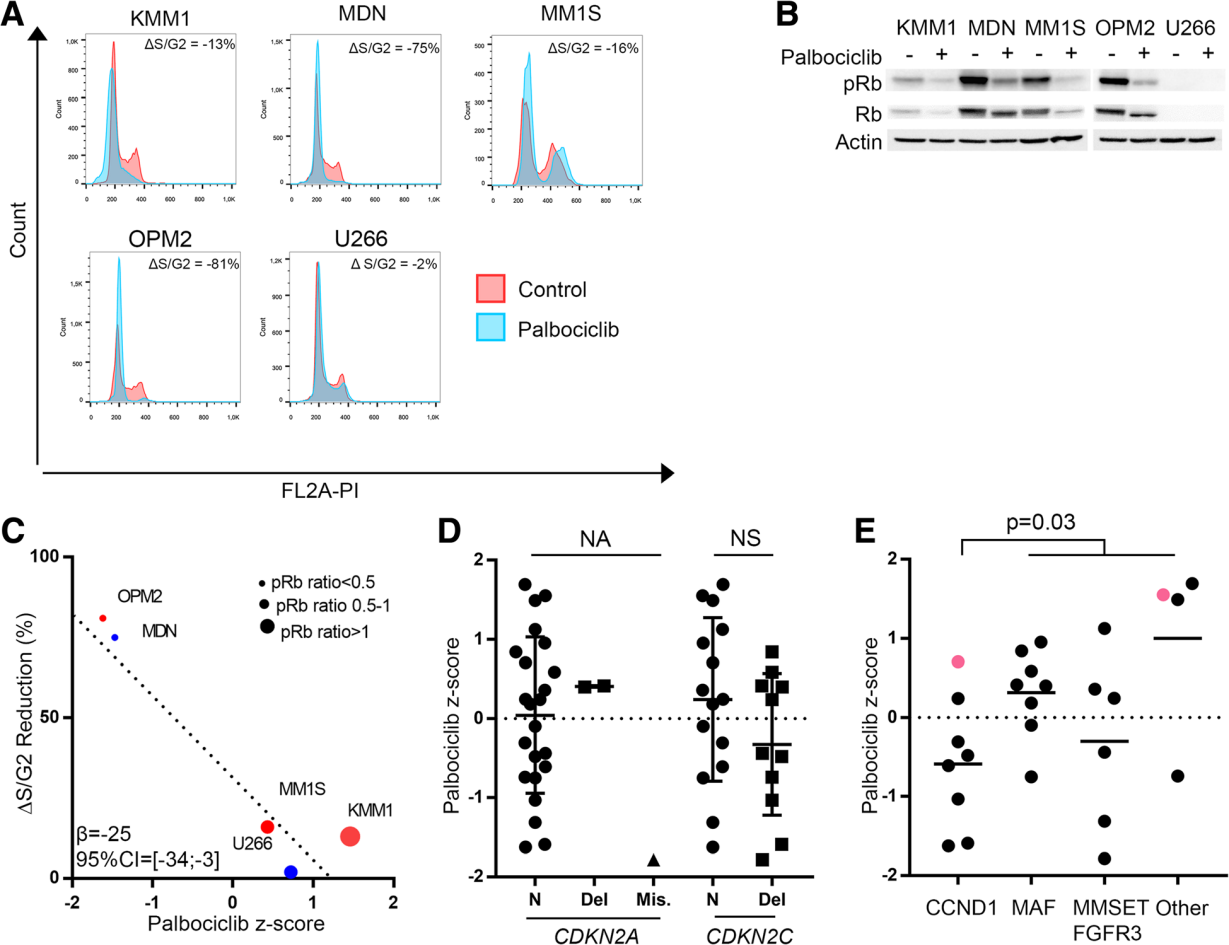
The sensitivity to palbociclib is associated with the lack of *RB1* deletion/mutation in CCND1+ human myeloma cell lines. **a** Cells were cultured for 24 h with palbociclib (500 nM); cell cycle modifications (**a**) and Rb phosphorylation (**b**) were assessed by propidium iodide staining and Western blotting, respectively. For HMCLs harboring a functional RB1, mean phase S/G2 reduction was 26% (95% CI = 16–34%) (paired *t* test, *p* = 0.03) and mean (pRb/Rb reduction was 63% (95% CI = 42–91%) (paired *t* test, *p* = .03). **c**, **d, e** Cells were cultured for 4 days with increasing concentrations of palbociclib, and sensitivity was determined by the area under the curve (AUC) using the MTT assay. **c** Correlation between S/G2 phase inhibition and palbociclib sensitivity (AUC z-score), bootstrapped robust linear regression. **d**, **e** Palbociclib sensitivity according to either the *CDKN2A*/*CDKNC* status or MM molecular classification. *RB1* abnormal HMCLs are indicated (Mann–Whitney test)

Approximately half (55%) of HMCLs showed impaired cell cycle regulation, mostly bi-allelic deletion of the *CDKN2C* locus (33% of HMCLs), *RB1* alterations (12%) or *CDKN2A* alterations (9%) (Additional file 1: Figures S3 & S5-“CellCycle”). Because deletions in *CDKN2A* or *CDKN2C*, by contrast to *RB1* deletion/mutation, were reported to favor cell cycle inhibition by the CDK4/CDK6 inhibitor palbocicib, we assessed whether altered HMCLs were sensitive to the inhibitor [36]. Palbociclib induced an inhibition of cell cycle that was correlated to pRb inhibition (*β* = − 25, 95% CI = [− 33.8; − 2.9]) (Fig. 5a–c). However, no correlation could be found between palbociclib sensitivity and the *CDKN2C* (*p =* 0.17), *CDKN2A* (*p =* 0.47) or *RB1* (*p =* 0.33) status (Mann–Whitney test, Fig. 5d). HMCLs overexpressing *CCND1* with an unaltered *RB1* showed a trend for better sensitivity than that of other HMCLs (*p* = 0.1) (Fig. 5e). Conversely, no correlation was found between palbociclib sensitivity and either the cell cycle pathway score (*β* = − 0.123, 95% CI = [− 0.89; 0.53]) or *CDK4*/*CDK6* expression levels (*β* = 0.49, 95% CI = [− 1.47; 1.97] and *β* = − 0.02, 95% CI = [− 0.46; 0.5]), respectively).

### Overactivation of NFκB by genomic alterations does not confer oversensitivity to proteasome inhibitors

**Fig. 6:**
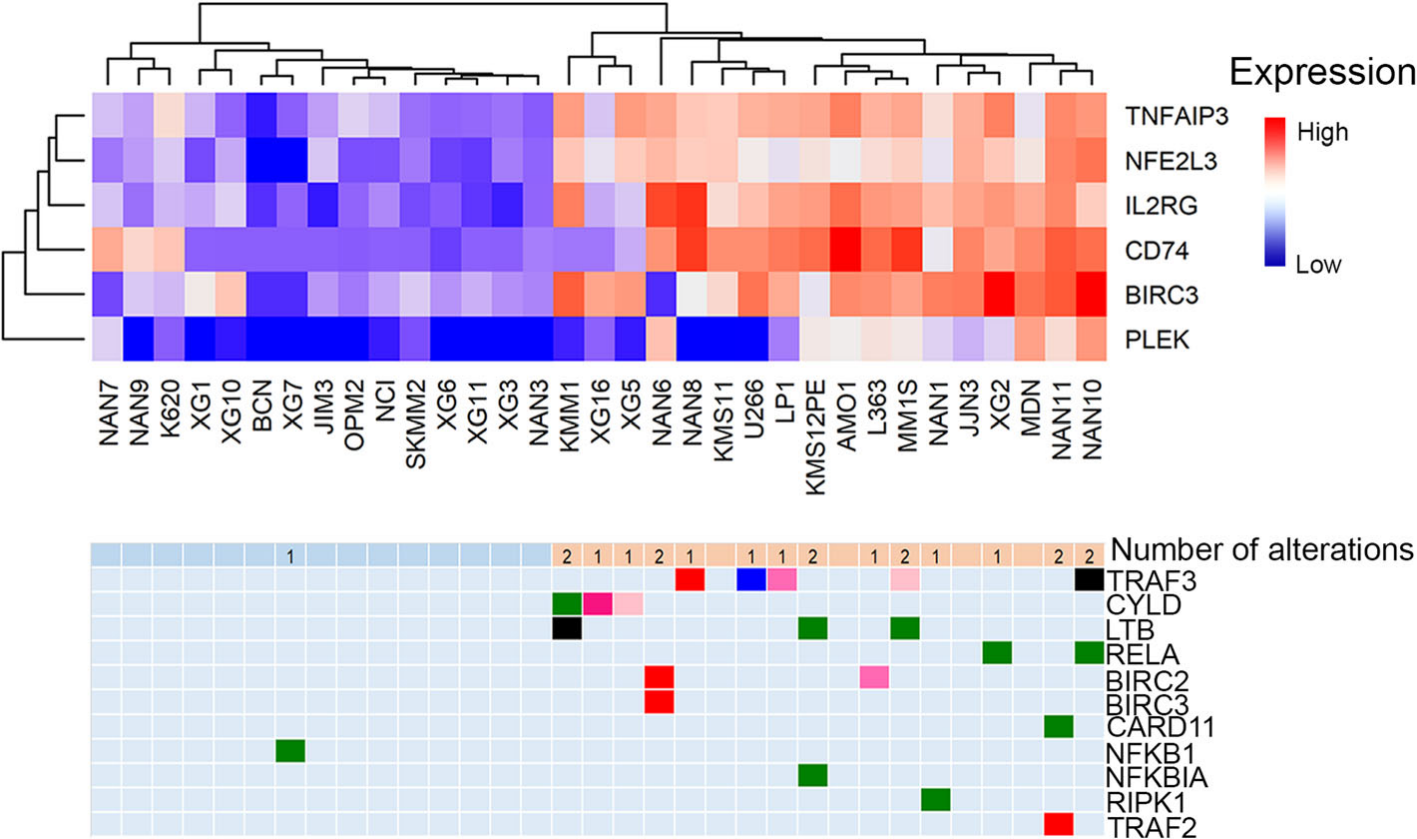
Mutations/deletions in the NFκB pathway genes are correlated with the overexpression of NFκB target genes. **a** The expression of genes significantly associated with mutation/deletion in NFκB pathway genes was identified using the limma algorithm. Clustering was performed with the most significant genes. **b** Representation of the NFκB pathway hits according to HMCL classification in **a**

The NFκB pathway was altered in 45% of HMCLs, mostly by inactivating *TRAF3* (frame-shift, non-sense mutation, insertion or deletion) or *BIRC2/BIRC3* (homozygous deletion) as previously reported in primary myeloma cells [20, 23]. Alterations in the NFκB pathway were associated with the overexpression of *NFκB* signature genes [37] (i.e., *CD74*, *TNFAIP3*, *IL2RG*, *BIRC3*, and *PLEK*), and we further identified that *NFE2L3* (a downstream target of TNF-α signaling displaying a NFκB site in the promoter) was highly expressed in samples harboring NFκB pathway hits (Fig. 6). Furthermore, we found no correlations between NFκB pathway dysregulation and sensitivity to proteasome inhibitors (*β* = 0.2, 95% CI = [− 0.25; 0.65] for bortezomib and *β* = − 0.09, 95% CI = [− 0.5; 0.16] for carfilzomib, Additional file 1: Figure S8).

### p53 and DNA damage pathways are associated with shifts in response to myeloma alkylating drugs

**Fig. 7:**
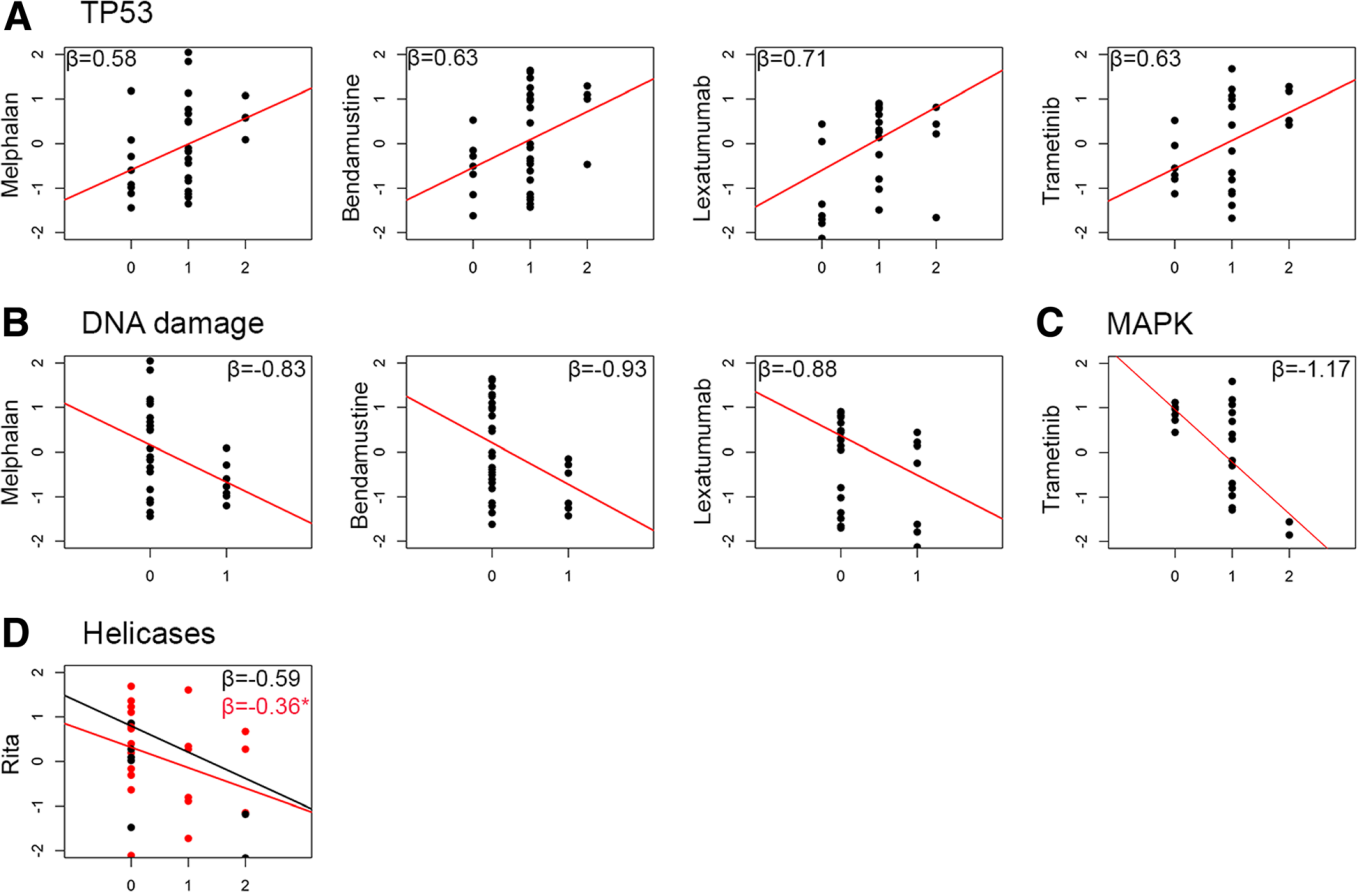
Significant associations between dysregulated pathways and drug responses. The number of hits in each pathway was plotted against the drug *z*-score. Robust linear regression is displayed; regression line was drawn according to coefficients obtained after 5000 bootstrapped replicates. Only significant associations of tested drugs with the pathways of interest are displayed. Drugs responses were detailed in Methods, as durations and assessment of response differ according to drugs pharmacodynamical specificities. **a** Drug responses associated with dysregulation in the p53 pathway. **b** Drug responses associated with dysregulation in the DNA damage pathway. **c** Drug responses associated with dysregulation in the MAPK pathway. **d** RITA sensitivity correlated with the helicase hit scores in *TP53*^*w*t^ HMCLs. *TP53*^*w*t^ and *TP53*^*Abn*^ HMCLs are represented by black and red dots, respectively. The lethal-dose-50 of RITA was determined as previously described [13]. Asterisk indicates not statistically significant

We previously reported that the sensitivity to alkylating drugs was impaired by p53 deficiency [12]. We further assessed whether hits in pathway(s) were associated with the response to drugs reported to be related to p53 deficiency. As shown in Fig. 7, p53 pathway alterations were associated with a lesser response to melphalan (*β* = 0.58, 95% CI = [0.08; –0.9]) and bendamustine (*β* = 0.63, 95% CI = [0.1; –1]). Of note, HMCLs harboring a high DNA damage kinase sensor score had high sensitivity to melphalan (*β* = − 0.83, 95% CI = [− 1.38; − 0.27]) and bendamustine (*β* = − 0.93, 95% CI = [− 1.52; − 0.28]), which was related to *TP53* status.

On the other hand, hits in the p53 pathway were also associated with reduced sensitivity to lexatumumab (*β* = 0.71, 95% CI = [0.01; 1.95]) and with a trend for increased sensitivity to mapatumumab (*β* = − 0.79, 95% CI = [− 1.24; − 0.08]). These correlations were related to the direct and indirect p53-mediated regulation of *TNFRSF10B* and *TNFRSF10A* expression, as previously reported [9].

Correlations between other clinically commonly used drugs (Imids, dexamethasone, and proteasome inhibitors) and pathways were not significant (Additional file 1: Figure S8).

### Deficiency in the DNA repair pathway does not predict the responses to RITA or CX-5461

We assessed whether efficacy of DNA targeting drugs could be related to specific alterations. We analyzed sensitivity to RITA and CX-5461, which induce DNA crosslinking and stabilize DNA G-quadruplex, respectively, and are known to involve DNA repair during DNA replication [13, 38, 39]. Since p53 is involved in DNA repair, we analyzed drug responses according to *TP53*^*status*^ of HMCLs. Sensitivity to RITA in *TP53*^*wt*^ HMCLs was enhanced by mutations in helicases (*β* = − 0.59, 95% CI = [− 1.04; − 0.01]) (Fig. 7). HMCL sensitivity to CX-5461 was not associated with any genes or pathway alterations (Additional file 1: Figure S8) although XG11, which displayed a homologous *BRCA2* mutation, showed the highest sensitivity to CX-5461.

## Discussion

WES was performed in 33 HMCLs, including 19 that had not been reported yet. HMCLs selected in this study were established between 1965 and 2015 in Europe, USA, or Japan and in the presence or absence of added recombinant IL6. While HMCLs’ ages spanned from ~ 55 to 3 years old, mutation load was similar among those, even if more recent HMCLs display lower mutation load. Our analysis showed that mutated genes were shared between HMCLs and primary myeloma cells, whatever the organ origin of samples that gave rise to the cell lines. Although HMCLs always emerged from patients with extra-medullary disease, no strong comparison could be made with primary or secondary PCL because of the very low number of sequenced PCLs yet, except for del17p (46% in sPCL) [29]. We thus compared mutation frequency with primary cells at diagnosis and at relapse (without any indication of medullary or extramedullary disease). Although the number of HMCLs was low as compared with patients, we nevertheless identified genes with a very high tumor load suggesting that some of them are drivers. The HMCLs mutational landscape may thus provide a panorama of mutations in refractory patients. The frequency of mutated “myeloma” genes in HMCLs was identical, lower, or higher when compared to primary cells at diagnosis or relapse [20–22]. While mutation rate in *KRAS* was similar between HMCLs and primary myeloma cells, the frequencies of *TP53* (67%), *CDKN2C* (33%), *PRKD2* (21%), *FAM46C* (15%), and *BRAF* (15%) dramatically increased compared to primary myeloma cells, either in DMM or RMM. The high *TP53* abnormality frequency (67%) in HMCLs identified by WES in our study (and confirmed by direct RT-PCR sequencing [1]) was not in good agreement with a previous WES study reporting a rate of 21% in HMCLs [40], which was highly underestimated: indeed, well-known *TP53* mutations in L-363, LP-1, and SKMM-2 (COSMIC database and p53.iarc.fr, Release = 18) were not reported in this study and at least three “HMCLs” were not of myeloma origin (ARH77, MC-CAR, CTV-1) [41, 42].

Our results clearly confirmed a major alteration in both proliferation control, with either loss of suppressor (*TP53*, *CDKN2C*, *RB1*) or acquisition of activator (*BRAF*, *RAS*) and in tumor suppression/drug response (*TP53*, *FAM46C*), as in most if not all cancers [43]. Because the loss of function of *TP53*, *FAM46C*, or *CDKN2C* are not directly targetable, drugs bypassing these proteins or exploiting their loss consequences are required. Indeed, as shown in Fig. 5, cells lacking *CDKN2C* expression were sensitive to the CDK4/6 inhibitor palbociclib, especially in the CCND1 group. This CCND1 impact was surprising because palbociclib is efficient against all CDK-CCND complexes, i.e., CDK4/CCND1, CDK4/CCND3, and CDK6/CCND2 [44]. Of note, *CCND2* myeloma cells overexpress *CDK6* and *CDK4* while *CCND1* myeloma cells overexpress *CDK4* but not *CDK6*, suggesting that CDK4 is “empty” of cyclin D in *CCND2* myeloma cells (Additional file 1: Figure S10). This free CDK4 pool might explain the low efficiency of palbociclib in *CCND2* HMCLs. Palbociclib has shown no global efficiency in MM patients without indication of their subgroup origin and their CCND1 expression [45]. However, since it is efficient (in combination) in patients with tumors overexpressing CCND1 such as mantle cell lymphoma or HR^+^ breast cancer, it might be of interest for patients with t(11;14) without Rb deficiency [46, 47]. Concerning *TP53*, we previously described p53 independent drugs, which were efficient whatever *TP53* status, such as PRIMA-1^Met^ that targets glutathione or BH3 mimetics that target anti-apoptotic proteins [6, 11]. We also reported that loss of p53 function favors measles virus replication and cell death in myeloma cells [48]. *FAM46C* was recently been shown to encode for a non-canonical poly(A) polymerase and its over expression in MM cells induced cell death [49]. *FAM46C* is a type I IFN-stimulated gene, and it might modulate virus replication such as the yellow fever virus (YFV) and the Venezuelan equine encephalitis virus (VEEV) [50]. Of note, anti-viral type I IFN pathway appeared highly impaired, suggesting defects in infection defense that might be exploited using oncolytic viruses such as measles virus [48, 51].

Concerning mutations with gain of function such as *RAS* mutations, we showed that sensitivity of 27 HMCLs to MEK1/2 inhibitor trametinib was associated to *RAS* mutations (70% of “*RAS* only” mutated HMCLs were sensitive), but not to *FGFR3* (none sensitive HMCL out of three with “*FGFR3* only” mutation). Concerning *BRAF*, four HMCLs out of five with *BRAF* mutation (and with *NRAS* mutations for two of them) were sensitive but the low number of “*BRAF* only” mutated HMCLs prevented definitive conclusions. Although all HMCLs without hit in *RAS/BRAF/FGFR3* genes were resistant to trametinib, all HMCLs with *NRAS* mutation were not sensitive since four *NRAS* mutated HMCLs were resistant. These data collectively suggest that mutation in *RAS/BRAF* genes is required but not sufficient for eliciting response to trametinib. The *BRAF/RAS* impact will be assessed in an ongoing clinical trial (NCT03091257) evaluating dabrafenib and/or trametinib in patients with relapsed and/or refractory multiple myeloma patients according to their *BRAF/RAS* mutation.

The high percentage of altered genes in DNA/chromatin repair/regulation, Fanconi pathway, and chromatin/DNA modification might be related to the frequency in relapsing patients [32]. Because of the lack of specific drugs, we could not directly assess the functionality/vulnerability of these pathways, which require a deep investigation. Of note, mutations in Fanconi genes were recently reported in patients at relapse, suggesting that drug escape might involve this pathway. HCLMs exhibiting such “BRCAness” will be a good model for assessing efficiency of drugs like USP1 and/or PARP inhibitors [23, 31, 52].

On the other hand, no major alteration was found in apoptosis pathway, either extrinsic/intrinsic or executive, showing that resistance to cell death was rather upstream of the mitochondria. In good agreement with the low number of alterations in apoptosis pathway, HMCLs were highly primed for death as shown by their BH3 profiling and their high response rate to BH3 mimetics [11, 53] (Additional file 1: Table S2). Considering the huge difference between cell responses to DNA damaging drugs and BH3 mimetics, loss of response was not on the mitochondrial side, and BH3 mimetics appear thus of major interest to target MM cells whatever their genomic alterations or responses to classical myeloma drugs.

## Conclusions

In summary, WES suggests that HMCLs harbor enriched mutations and defects in cell cycle, p53, recombination/DNA repair, NFκB, and epigenetic genes. Importantly, some very early pathogenic events such as IgH translocations and MAPK pathway mutants are stable over time and are not enriched by in vitro long-term culture, thus making HMCLs a reliable drug screening model for refractory patients at diagnosis or relapse. What is more, detection at diagnosis of mutations/deletions in genes associated with progression and HMCLs (i.e., *CDKN2C*, *FAM46C*, *TRAF3*, *PRKD2*) might identify particularly aggressive sub-clones warranting adapted treatment strategies and surveillance. WES results suggest that in addition to target apoptosis using BH3 mimetics and the antiviral deficiency using oncolytic viruses, targeting DNA damage, recombination/DNA repair, and epigenetic modifiers should be further investigated and might offer significant options for high-risk and refractory patients, including extramedullary diseases.

## Additional files

Additional file 1: Additionnal Analyses and Plots. (DOCX 17568 kb)

Additional file 2: MAF File for Sequence variants. (XLSX 16253 kb)

Additional file 3: Lollipop plots of the main sequence variants. (PDF 2025 kb)

## Abbreviations

DMM: Multiple myeloma at diagnosis
HMCL: Human myeloma cell lines
PCL: Plasma cell leukemia
pPCL: Primary PCL
RMM: Relapsing multiple myeloma
SNP: Single nucleotide polymorphism
sPCL: Secondary PCL
WES: Whole-exon sequencing
