# Supplementary material for: Whole-exon sequencing of human myeloma cell lines shows mutations related to myeloma patients at relapse with major hits in the DNA regulation and repair pathways
Source: J Hematol Oncol. 2018 Dec 13;11:137. doi: 10.1186/s13045-018-0679-0 (PMC6293660; doi:10.1186/s13045-018-0679-0)
Supplement: Supplementary file 1 — Additionnal Analyses and Plots. (DOCX 17568 kb) [file 13045_2018_679_MOESM1_ESM.docx]

#### Supplementary Methods

Cell Lines

Whole exome Sequencing

Copy Number estimation from WES

Variants validations

Functional assays

Statistical Analyses

Supplementary Tables

Table S1: HMCLs characteristics

Table S2: Direct Sequencing Primers

Table S3: HMCLs’ main characteristics and drug screenings

Supplementary Figures

Figure S1: SNP analysis of 33 HMCLs

Figure S2: Mutants Classification

Figure S3: Genomic Signatures

Figure S4: Lollipop Plots

Figure S5: Co-occurrence plot (Somatic Interaction plot)

Figure S6: GO, Reactome and Kegg pathway enrichments

Figure S7: Chord Diagram of pathway hits relative to recurrent translocation subgroup

Figure S8: Drugs responses according to pathway hits

Figure S9: Major Hits classified in their respective pathways

Figure S10: CDK4/6 expression according to CCND1 expression

Supplementary Methods

## Cell Lines

Gene expression profile of HMCLs has been previously published^1^. Expression levels were processed from CEL files by GCRMA^2^ and further normalized (inter-batch) by quantile-normalization^3,4^.

## Whole Exome Sequencing

DNA was extracted from cell lines and sheared, degradation was controlled by migration on agarose gel (1%), purity was assessed by NANODROP (260nm/280nm ratio between 1.7-2 and 260nm/230nm ratio superior to 1.4). Double stranded DNA was dosed by PicoGreen and 3µg of DNA was further processed. After sequencing, for sakes of parallelization, samples were processed individually. Reads were aligned to the GRCh37 human reference genome using BWA (v0.7.10-r789)^5^. Duplicated reads were marked by Picard tool (v1.119), indels were realigned around capture (+/-500bp) and base quality recalibration was finally performed (Genome Analysis Toolkit, (GATK, v3.2.2).

Copy Number Variation (CNV) calling was performed with two techniques: ExomeCOPY using Hidden Markov Model ^6^ and CANOES ^7^ which used a negative binomial regression. Briefly, read counts were obtained by bedtools on a bed list of ensemble canonical exon regions. Only regions with a mapping quality >20 were retained. GC content was assessed by GATK (GCContentByInterval) on the intervals. CANOES was executed with a probability of CNV event of 1e-6. Exome Copy was executed with the following parameters: goto.cnv=1e-3, expected CNVs (S): n=0-4, init.phi=”counts”, goto.normal=1/20. Results were validated by visual inspection of BAM read depth in Integrative Genomics Viewer^8^.


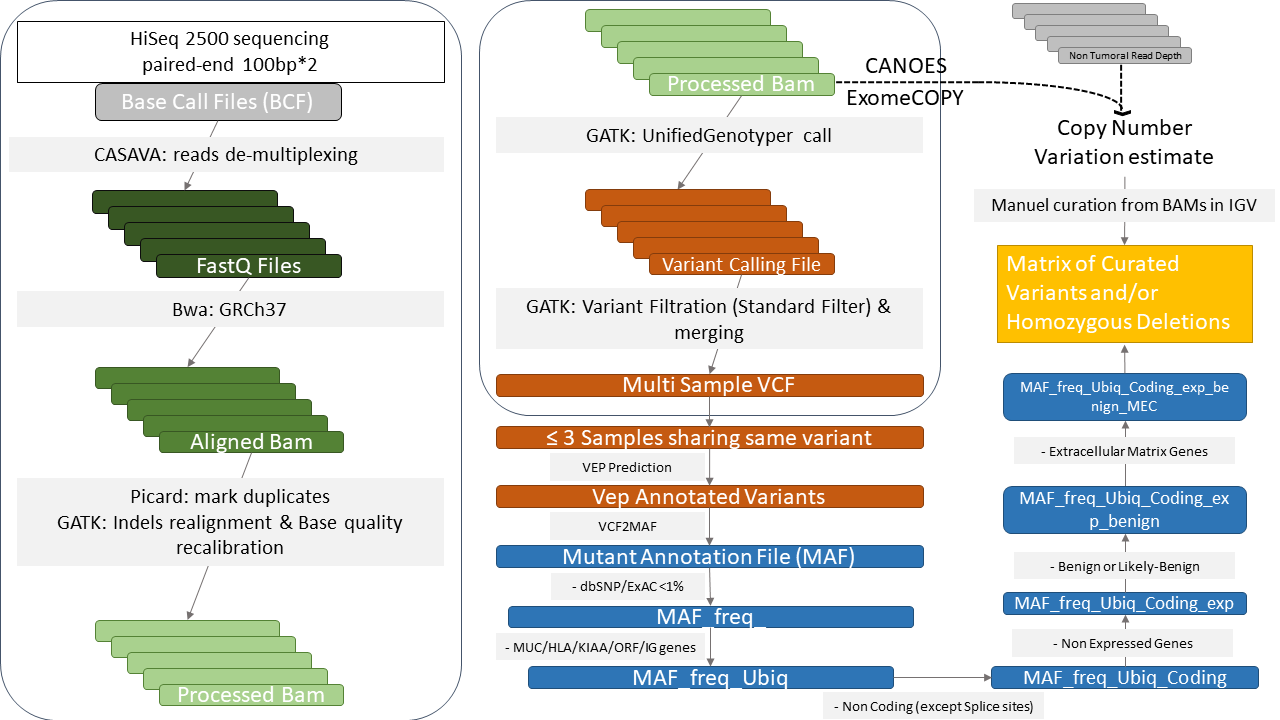


## Functional assays

Cell count and viability were measured using MMT (3-(4,5-dimethylthiazol-2-yl)-2,5-diphenyltetrazolium bromide, SIGMA) assay. Briefly, cells were incubated in 96-well microplates (triplicate wells) for 48 hours (Bortezomib, Carfilzomib, CX4615), 72 hours (Pomalidomide, Bortezomib, Carfilzomib) or 120 hours (Trametinig, Palbociclib) before MTT reaction. Wells were submitted to lysate and absorbance was measured at 550nm. Results were expressed as the percentage of control wells. All experiments were replicated 4 times. Response to Melphalan, Bendamustine, FAS and TRAIL-R agonist antibodies, PRIMA-1^Met^, RITA, nutlin3a, dexamethasone, ABT-737 and ABT-199 were previously reported^9^.

## Statistical Analyses

For Reactome, KEGG and GO annotations, only genes with at least 2 mutants after filtering were considered. MAF manipulation was performed by maftools packages^10^, oncoprints, heatmaps and circular diagrams were generated by ComplexHeatmap package. Area Under the Curve was estimated with Graphpad Prism v7.0 for Palbociclib (0-1µM), CX5461(0-1µM) and Trametinib (0-25nM). Pathway scores were calculated as the sum of all genes in the considered pathway. Regarding low number of samples, regression between scores and drug responses were calculated by robust linear regression using a M estimator (rlm, MASS package). Coefficients were further bootstrapped by Boot function (car package), with 5000 replicates (seed= 22062016) and considered significant when 95% Confidence Interval (95%CI) didn’t overlap with zero, only β_1_ coefficients are presented in the text. If matrix was singular for rlm regression, a neglectable amount of noise was added to pathway hits scores (noise from a normal distribution, with mean of zero and variance of 1e-5). Patients microarray data were obtained from NIH Gene Expression Omnibus under accession number GSE2658^11^, as raw data were not provided, MAS5 were simply normalized by quantiles and log2 transformed.

Supplementary Tables

| **HMCL Name** | **IL-6 dependence^1^** | **Year^2^** | **Disease^3^** | **Patient sample^4^** | **Gender** | **Isotype** | **t(14q32 or 22q11 ;)** | **Target genes** | **Number of genes mutated/deleted** |
| --- | --- | --- | --- | --- | --- | --- | --- | --- | --- |
|  |  |  |  |  |  |  |  |  |  |
| U266 | - | 1965 | MM | PB | M | El | t(11;14) | *CCND1* | *427* |
| L363 | - | 1977 | PCL | PE | F | NS | t(20;22) | *MAF-B* | *429* |
| MM1S | - | 1980 | PCL | PB | F | Al | t(14;16) | *c-MAF* | *520* |
| KMM1 | - | 1980 | MM | SC | M | l | t(6;14) | *CCND3* | *843* |
| AMO1 | - | 1981 | PCT (D) | AF | F | Ak | t(12;14) | unknown | 473 |
| NCI-H929 | - | 1984 | MM | PE | F | Ak | t(4;14) | *MMSET/FGFR3* | *412* |
| OPM2 | - | 1985 | MM | PB | F | Gl | t(4;14) | *MMSET/FGFR3* | *496* |
| LP1 | - | 1986 | MM | PB | F | Gl | t(4;14) | *MMSET/FGFR3* | *369* |
| Karpas620 | - | 1987 | P-PCL(D) | PB | F | Gk | t(11;14) | *CCND1* | *346* |
| KMS11 | - | 1987 | MM | PE | F | Gk | t(4;14) | *MMSET/FGFR3* | *508* |
| KMS12PE | - | 1987 | MM | PE | F | NS | t(11;14) | *CCND1* | *686* |
| JJN3 | - | 1987 | MM | PE | F | Ak | t(14;16) | *c-MAF* | *429* |
| SKMM2 | - | 1989 | PCL | PB | M | Gk | t(11;14) | *CCND1* | *389* |
| JIM3 | - | <1990 | MM | PE | F | A | t(4;14) | *MMSET* | *428* |
|  |  |  |  |  |  |  |  |  |  |
| XG1 | ++ | 1990 | MM | PB | M | Ak | t(11;14) | *CCND1* | *448* |
| XG2 | ++ | 1990 | MM | PE | F | Gl | t(12;14) | unknown | 473 |
| XG3 | ++ | 1990 | P-PCL (D) | PE | F | l | t(14 ;?) | *unknown* | *374* |
| XG5 | ++ | 1990 | MM | PB | F | l | t(11;14) | *CCND1* | *431* |
| XG6 | ++ | 1990 | MM | PB | F | Gl | t(16;22) | *c-MAF* | *565* |
| XG7 | + | 1990 | MM | PB | F | Ak | t(4;14) | *MMSET* | *399* |
| XG10 | + | >1990 | PCT | AF | F | Ak | t(14;?) | *unknown* | *490* |
| XG11 | ++ | >1990 | PCL | PB | F | l | t(11;14) | *CCND1* | *355* |
| XG16 | + | >1990 | PCL | PB | M | k | none | *none* | *374* |
| BCN | + | 1996 | MM | PB | F | Gk | t(14;16) | *c-MAF* | *503* |
| MDN | + | 1997 | PCL | PB | M | Gk | t(11;14) | *CCND1* | *385* |
|  |  |  |  |  |  |  |  |  |  |
| NAN1 | + | 2000 | MM | PE | M | Ak | t(14;16) | *c-MAF* | *430* |
| NAN3 | + | 2001 | MM | PE | F | Ak | t(4;14) | *MMSET* | *341* |
| NAN6 | + | 2005 | MM | PB | F | Ak | t(14;20) | *MAF-B* | *428* |
| NAN7 | + | 2007 | P-PCL | PB | F | k | t(11;14) | *CCND1* | *308* |
| NAN8 | + | 2010 | P-PCL (D) | PB | F | A | t(4;14) | *MMSET/FGFR3* | *483* |
| NAN9 | + | 2011 | PCL | PB | F | A | t(4;14) | *MMSET/FGFR3* | *351* |
| NAN10 | + | 2012 | PCL | PB | M | l | t(11;14) | *CCND1* | *388* |
| NAN11 | + | 2015 | P-PCL | PB | M | Gl | t(14;16) | *c-MAF* | *290* |

**Table S1. Characteristics of the 33 HMCLs.** 1: IL6 derived HMCLs, -: IL6-independent, *: autocrine stimulation, +/++: IL6-dependency, 2: year of generation of HMCL, 3: Plasma Cell disorder classification: MM: Multiple Myeloma, PCL: Plasma cell Leukemia, PCT : plasmacytoma, D : Diagnosis, P-PCL : Primary Plasma Cell Leukemia, 4: Patient sample origin: PB: Peripheral Blood, PE: Pleural Effusion, AF: Ascite Fluid, SC: Sub-Cutaneous.

| **Gene** | **Forward primer** | **Reverse primer** | **HMCLs** | | | | **AMO1** | **BCN** | **JIM3** | **JJN3** | **K620** | **KMM1** | **KMS11** | **KMS12PE** | **LP1** | **MDN** | **MM1S** | **NAN1** | **NAN10** | **NAN11** | **NAN3** | **NAN6** | **NAN8** | **NCI-H929** | **OPM2** | **U266** | **XG1** | **XG10** | **XG11** | **XG16** | **XG3** | **XG5** | **XG6** | **XG7** |
| --- | --- | --- | --- | --- | --- | --- | --- | --- | --- | --- | --- | --- | --- | --- | --- | --- | --- | --- | --- | --- | --- | --- | --- | --- | --- | --- | --- | --- | --- | --- | --- | --- | --- | --- |
| HRAS | aggagaccctgtaggaggac | CAGGATGTCCAACAGGCACG | NAN11 |  |  |  |  |  |  |  |  |  |  |  |  |  |  |  |  | h |  |  |  |  |  |  |  |  |  |  |  |  |  |  |
| BRAF | ACACTTGGTAGACGGGACTC | GAGGCGAGAATTTGGGGAAAG | NAN10 | U266 | NAN1 | BCN |  | h |  |  |  |  |  |  |  |  |  | H | h |  |  |  |  |  |  | h |  |  |  |  |  |  |  |  |
| FGFR3 | CTGTCGAGCCACCAATTTCA | GGATGAACAGGAAGAAGCCC | KMS11 |  |  |  |  |  |  |  |  |  | H |  |  |  |  |  |  |  |  |  |  |  |  |  |  |  |  |  |  |  |  |  |
| FANCA | TCAGATACTCACTCACAGCCC | ACCAACTCCTCTGCACTCAG | MDN |  |  |  |  |  |  |  |  |  |  |  |  | h |  |  |  |  |  |  |  |  |  |  |  |  |  |  |  |  |  |  |
| FANCD2 | GAAGGGGAAGGTGCTCACTC | GGAGGATGTCTTGCTGCCAT | KMM1 | KMS12 |  |  |  |  |  |  |  | h |  | h |  |  |  |  |  |  |  |  |  |  |  |  |  |  |  |  |  |  |  |  |
| FANCE | ACTGGGGTCGCTTGCTCG | ATCCCTTCGCAGCAATTCCC | XG3 |  |  |  |  |  |  |  |  |  |  |  |  |  |  |  |  |  |  |  |  |  |  |  |  |  |  |  | H |  |  |  |
| FANCG | CTTGGAGCTGACTGTCACCT | CTCAATGAGAAACTGCGGGG | XG3 | XG7 |  |  |  |  |  |  |  |  |  |  |  |  |  |  |  |  |  |  |  |  |  |  |  |  |  |  | h |  |  | H |
| FANCI | CCTGTCATCCTCTCAGTGCA | CCAGCTGAGAGTTCCTTCGA | XG1 | XG16 | NAN6 |  |  |  |  |  |  |  |  |  |  |  |  |  |  |  |  | h |  |  |  |  | h |  |  | h |  |  |  |  |
| FANCL | TCATCTCGGCTCAGGGAAGA | GAACTGGGGAGGAGGAGGTA | BCN |  |  |  |  | h |  |  |  |  |  |  |  |  |  |  |  |  |  |  |  |  |  |  |  |  |  |  |  |  |  |  |
| FANCM-PCR1 | AAAGACCTTTATTGCCGCCG | CCTGTCATTTCGGCCATGTG | K620 |  |  |  |  |  |  |  | h |  |  |  |  |  |  |  |  |  |  |  |  |  |  |  |  |  |  |  |  |  |  |  |
| FANCM-PCR2 | TGCGTAGTCCAATGATGAACA | CCTCCTCACTTGAATCATCTGGT | KMS12PE |  |  |  |  |  |  |  |  |  |  | H |  |  |  |  |  |  |  |  |  |  |  |  |  |  |  |  |  |  |  |  |
| ATM-PCR1 | GGCTATTCAGTGTGCGAGAC | ATGTCGCTGTTGGGGTAGAA | AMO1 |  |  |  | H |  |  |  |  |  |  |  |  |  |  |  |  |  |  |  |  |  |  |  |  |  |  |  |  |  |  |  |
| ATM-PCR2 | TGATGCACTTCCATTGACAAGAC | AGGACCCACTTCTCCCAAGC | XG6 |  |  |  |  |  |  |  |  |  |  |  |  |  |  |  |  |  |  |  |  |  |  |  |  |  |  |  |  |  | h |  |
| ATR-PCR1 | ACTCTCAGCCAACCTCCGTG | TCATGACCACTGGCCATTCC | KMS11 |  |  |  |  |  |  |  |  |  | h |  |  |  |  |  |  |  |  |  |  |  |  |  |  |  |  |  |  |  |  |  |
| ATR-PCR2 | TTCCCTTGAATACAGTGGCCT | GACAGTCCTTGAAAGTACGGC | BCN |  |  |  |  | h |  |  |  |  |  |  |  |  |  |  |  |  |  |  |  |  |  |  |  |  |  |  |  |  |  |  |
| ATR-PCR3 | CAGCATCTGTCTCTGGAGCA | TCTTGTTTTCGCACGTCAGCA | KMM1 |  |  |  |  |  |  |  |  | h |  |  |  |  |  |  |  |  |  |  |  |  |  |  |  |  |  |  |  |  |  |  |
| BRCA1 | TGCAGTGTGGGAGATCAAGAATT | ACACAGGGGATCAGCATTCA | NAN8 |  |  |  |  |  |  |  |  |  |  |  |  |  |  |  |  |  |  |  | h |  |  |  |  |  |  |  |  |  |  |  |
| BRCA2 | CTGGCAGCTATGGAATGTGC | GAAAGGGATGACACAGCTGC | NCI-H929 |  |  |  |  |  |  |  |  |  |  |  |  |  |  |  |  |  |  |  |  | H |  |  |  |  |  |  |  |  |  |  |
| TRAF3-PCR1 | CTGCTGAAGGAGTGGAGCAA | CGCTGCTCTTCATGCTGTCT | LP1 |  |  |  |  |  |  |  |  |  |  |  | H |  |  |  |  |  |  |  |  |  |  |  |  |  |  |  |  |  |  |  |
| TRAF3-PCR2 | AGATGTGTGCCAGGGTCTAC | ACTTATCAGGGATCGGGCAG | NAN10 | MM1S | U266 |  |  |  |  |  |  |  |  |  |  |  | H |  | h |  |  |  |  |  |  | H |  |  |  |  |  |  |  |  |
| BAX | CGCGGACCCGGCGAGAG | CACAGTCCAAGGCAGCTGGG | KMS11 | OPM2 |  |  |  |  |  |  |  |  | h |  |  |  |  |  |  |  |  |  |  | h |  |  |  |  |  |  |  |  |  |  |
| BAK1 | CCGACGCTATGACTCAGAGT | ATGCTGGTAGACGTGTAGGG | XG7 |  |  |  |  |  |  |  |  |  |  |  |  |  |  |  |  |  |  |  |  |  |  |  |  |  |  |  |  |  |  | h |
| APAF1-PCR1 | GAGAAGCTCTGGAAAAGGACA | ATGGAGAAGGGCAGCAAGAT | NAN8 |  |  |  |  |  |  |  |  |  |  |  |  |  |  |  |  |  |  |  | h |  |  |  |  |  |  |  |  |  |  |  |
| APAF1-PCR2 | CAGTCCAGGTTTCAGCACAA | CCATCAAATGACCAAGAAAGC | JIM3 | L363 | NAN6 | XG7 |  |  |  |  |  |  |  |  |  |  |  |  |  |  |  |  |  |  |  |  |  |  |  |  |  |  |  |  |

**Table S2: Direct Sequencing Primers.** h: heterozygous; H: Homozygous

|  | Melphalan (LD50) | Bendamustine (LD50) | Nutlin-3a(LD50) | PRIMA-1^Met^(LD50) | Lexatumumab (CD) | Mapatumumab (CD) | CH11 (CD) | Dexamethasone (LD50) | Bortezomib (LD50) | Carfilzomib (LD50) | Pomalidomide (I) | CX-5461 (I) | Palbociclib (I) | Trametinib (I) | ABT-737 (LD50) | ABT-199 (LD50) | A-1210477 (CD) | BIM-peptide (MP) |
| --- | --- | --- | --- | --- | --- | --- | --- | --- | --- | --- | --- | --- | --- | --- | --- | --- | --- | --- |
| AMO1 | -0,29 | -0,28 | -1,34 | 0,35 | -1,62 | 0,92 | 1,22 | 0,67 | 3,04 | 2,99 | 1,47 | NA | 1.68 | 0.40 | 0,67 | 0,41 | -0,47 | 0,38 |
| BCN | -0,98 | -1,15 | -1,34 | 0,46 | 0,44 | 1,36 | 0,60 | -2,50 | -1,35 | -0,39 | -0,38 | NA | 0.20 | -0.97 | 0,47 | 0,53 | 1,35 | 0,26 |
| JIM3 | 2,05 | 0,97 | 0,70 | 4,42 | 0,87 | 1,23 | -0,03 | -0,69 | 1,28 | 0,03 | 1,47 | 0,17 | 1.12 | 1.12 | 1,44 | -0,02 | 1,32 | 0,51 |
| JJN3 | 0,77 | 1,00 | 0,70 | -0,74 | 0,87 | 1,36 | -1,43 | 0,21 | -0,15 | -0,53 | -0,68 | NA | 0.95 | 0.69 | -0,07 | 0,14 | 0,97 | 0,14 |
| K620 | 0,49 | -0,09 | 0,70 | -0,60 | 0,82 | -1,22 | 0,53 | 0,33 | 1,36 | 0,60 | NA | 0,51 | -1.55 | -0.30 | -1,41 | -2,12 | 1,58 | 0,49 |
| KMM1 | 0,09 | -0,47 | 0,70 | 1,05 | 0,22 | -0,81 | -0,68 | 0,67 | -0,10 | -0,01 | 1,47 | -0,07 | 1.48 | 1.18 | 0,82 | 0,62 | -1,20 | 0,17 |
| KMS11 | -0,18 | 1,47 | 0,70 | -0,24 | 0,48 | -1,06 | -1,68 | 0,04 | -0,45 | 2,99 | -0,22 | NA | NA | NA | -0,07 | 0,25 | 0,74 | 0,17 |
| KMS12PE | 2,05 | 1,25 | 0,70 | 1,10 | 0,31 | -1,03 | -0,90 | 0,67 | 1,69 | -0,53 | -0,41 | -0,04 | -1.00 | 0.72 | -1,41 | -1,68 | 0,74 | 0,35 |
| L363 | 0,59 | 1,10 | 0,70 | 0,13 | 0,44 | 0,41 | 0,91 | -0,97 | 0,44 | -0,53 | -0,75 | -1,09 | -0.72 | 0.30 | 0,42 | 0,41 | -0,28 | -0,13 |
| LP1 | 1,13 | -0,39 | 0,70 | -0,24 | 0,87 | 0,10 | 0,25 | 0,61 | 0,29 | 0,69 | -0,22 | NA | -1.28 | 1.59 | 0,55 | 0,69 | 1,03 | -0,58 |
| MDN | -1,44 | -1,62 | -1,43 | 0,79 | -1,70 | -1,09 | -1,33 | 0,67 | -0,94 | -1,11 | -0,87 | NA | -1.58 | -1.29 | -0,95 | -2,32 | 0,04 | 0,64 |
| MM1S | -1,07 | -0,01 | -1,34 | -1,04 | -0,80 | 0,92 | 1,09 | -1,31 | -0,15 | -0,34 | 1,47 | -0,91 | 0.40 | -0.97 | 1,44 | 0,53 | 0,55 | 0,59 |
| NAN1 | 0,58 | 1,29 | 0,70 | 0,07 | 0,82 | -1,60 | 0,35 | 0,27 | 0,64 | -0,34 | -0,87 | -0,36 | 0.84 | 1.07 | -0,07 | -0,11 | -0,63 | 0,52 |
| NAN10 | -0,84 | -1,14 | 0,70 | -0,71 | 0,91 | 0,22 | 0,13 | 0,61 | 0,06 | -0,53 | 1,47 | 2,71 | -0.46 | -1.85 | -0,79 | -0,50 | 0,11 | 0,59 |
| NAN11 | -0,34 | -0,82 | -1,45 | -1,08 | -1,49 | -0,31 | -1,61 | 0,50 | 0,23 | -0,62 | -0,87 | 0,53 | 0.42 | -1.25 | -0,26 | 0,53 | -0,28 | NA |
| NAN3 | -0,10 | -0,34 | 0,70 | -0,65 | 0,87 | 0,00 | -0,21 | -0,58 | -0,79 | -0,53 | -0,44 | 0,38 | NA | -1.56 | 0,52 | 0,62 | -0,57 | 0,38 |
| NAN6 | -1,13 | -1,21 | 0,70 | -0,04 | 0,78 | -0,31 | -0,12 | -0,18 | -1,09 | -1,28 | -0,58 | NA | -0.08 | 0.84 | -0,53 | 0,39 | -1,27 | 0,45 |
| NAN7 | -0,44 | 0,47 | 0,70 | -0,18 | 0,65 | 0,82 | -1,21 | 0,67 | -0,73 | -0,41 | 1,47 | NA | NA | NA | -1,94 | -1,57 | -0,60 | 0,52 |
| NAN8 | 0,51 | -0,45 | 0,70 | 1,07 | 0,91 | 0,92 | 1,22 | -2,61 | -0,61 | 0,48 | -0,80 | 0,02 | -0.42 | 0.89 | 0,47 | 0,44 | 0,87 | -0,03 |
| NAN9 | -0,92 | -0,28 | -1,42 | -1,04 | -1,79 | 1,39 | 1,19 | 0,67 | -1,53 | 0,48 | -0,65 | NA | NA | 0.45 | 0,29 | 1,17 | -1,30 | 0,45 |
| NCI | 0,08 | -0,51 | -1,34 | -0,57 | -1,36 | 0,85 | 1,28 | 0,38 | -0,79 | -0,53 | -0,87 | -0,32 | 0.25 | -0.18 | 0,43 | 0,62 | -1,24 | 0,51 |
| OPM2 | 1,08 | 1,00 | 0,70 | -0,43 | -1,66 | -1,22 | -0,09 | -2,83 | -1,07 | -0,79 | -0,87 | 1,12 | -1.74 | 0.40 | 0,36 | 0,62 | -1,24 | 0,54 |
| SKMM2 | -0,16 | 1,61 | 0,70 | 0,02 | 0,27 | -1,38 | -1,52 | 0,16 | 1,74 | 0,01 | -0,43 | NA | 0.25 | 1.01 | -2,24 | -2,12 | 1,22 | -2,39 |
| U266 | 1,84 | 1,64 | 0,70 | 0,13 | 0,87 | 0,29 | 1,00 | 0,33 | 0,16 | 1,98 | 1,47 | -0,11 | 0.71 | -0.80 | 1,44 | 0,80 | 1,51 | 0,12 |
| XG1 | -0,84 | -1,36 | 0,70 | 0,24 | 0,78 | -0,22 | 1,13 | 0,67 | -0,05 | -0,53 | -0,87 | NA | NA | NA | 1,44 | 0,75 | -0,18 | -0,32 |
| XG10 | -1,12 | -0,69 | -1,51 | -0,18 | 0,05 | -1,50 | -1,27 | 0,67 | 0,16 | -0,46 | NA | 0,00 | -0.71 | -0.69 | 0,47 | 0,46 | -0,85 | 0,55 |
| XG11 | -1,20 | -1,43 | 0,70 | -0,29 | 0,14 | -0,78 | -0,03 | 0,67 | 0,13 | 0,36 | 1,47 | -2,23 | -0.29 | -1.29 | -0,95 | 0,34 | 0,97 | -3,47 |
| XG16 | -1,35 | -0,61 | 0,70 | -0,75 | -1,02 | -0,69 | -0,99 | 0,55 | 0,00 | 0,57 | NA | NA | 1.54 | 0.30 | -0,95 | 0,34 | -0,76 | NA |
| XG2 | 0,67 | 0,81 | 0,70 | -0,26 | 0,87 | -0,56 | -1,02 | -0,58 | -0,10 | -0,53 | -0,75 | NA | NA | NA | 0,09 | 0,41 | -0,12 | 0,31 |
| XG3 | -0,60 | -0,15 | -1,30 | -0,90 | -2,13 | 1,36 | 0,44 | 0,44 | -1,42 | -1,11 | -0,87 | NA | NA | NA | 0,29 | 0,47 | -1,36 | 0,59 |
| XG5 | 0,67 | 1,10 | 0,70 | 0,15 | 0,14 | -0,75 | 0,91 | 0,67 | 0,64 | -0,36 | 1,47 | 0,31 | -0.59 | 0.97 | -1,57 | -2,12 | 1,48 | 0,16 |
| XG6 | -0,77 | -1,26 | -1,43 | -0,54 | -0,25 | 1,23 | 0,78 | 0,50 | 0,08 | -0,18 | -0,60 | -0,61 | 0.59 | -0.80 | 0,34 | 0,41 | -0,92 | 0,24 |
| XG7 | 1,18 | 0,53 | -1,41 | 0,52 | 0,44 | 1,17 | 1,09 | 0,67 | -0,63 | 0,46 | -0,29 | NA | NA | NA | 1,30 | 0,62 | -1,24 | -2,70 |

**Table S3: HMCLs’ main characteristics and drug screenings.** Results are expressed as z-scores of sensitivity (mean-centered). LD50: Lethal Dose 50, I: Inhibition, CD: Cell Death at fixed dosing, MP: Mitochondrial Priming

Supplementary Figures


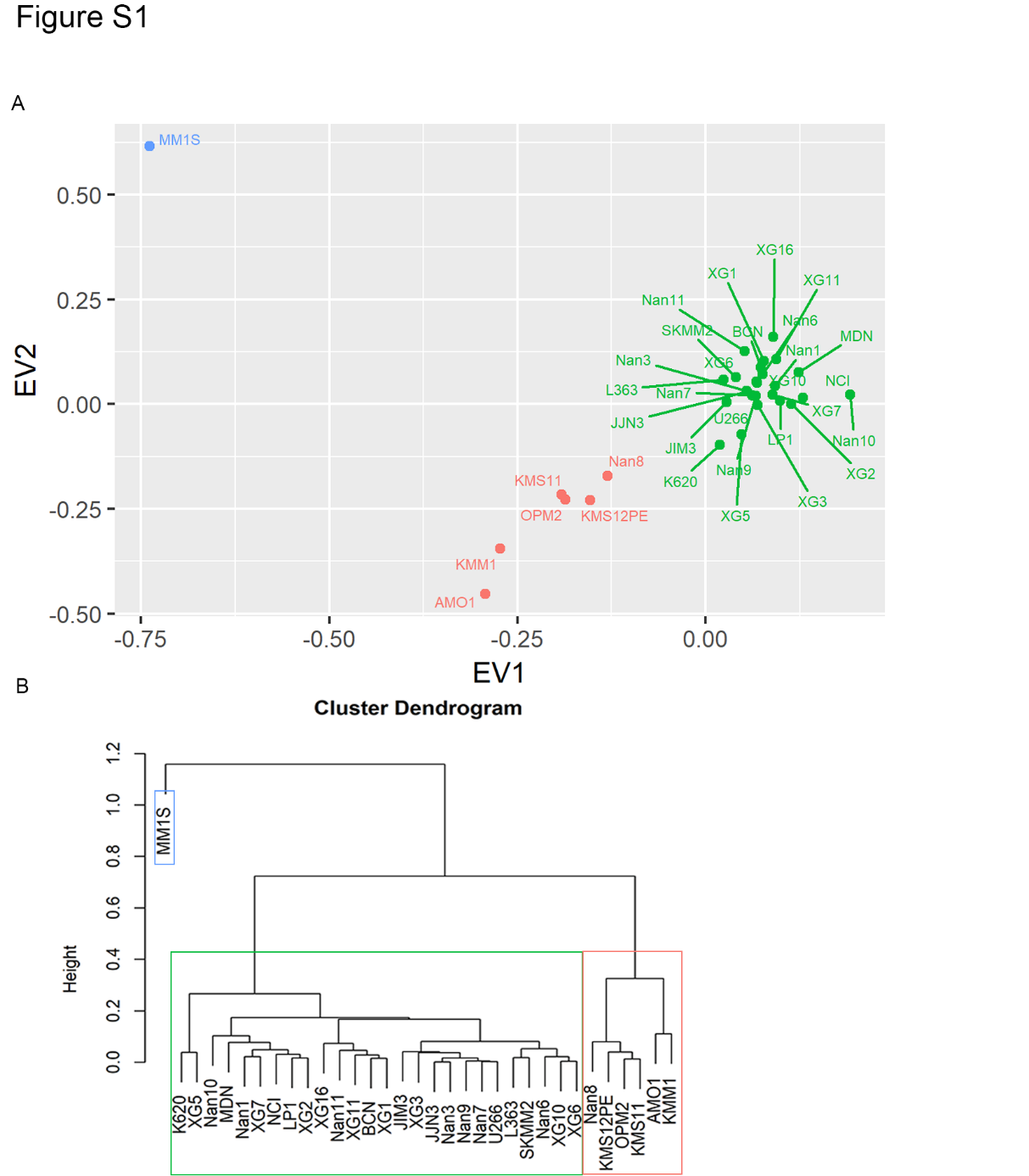


**Figure S1: SNP analysis of 33 HMCLs.** Samples SNPs (n= 609585) were analyzed by SNPRelated package. A: Principal Component Analysis was conducted on 6513 SNPs (475289 SNPs were kept after filtering of 10258 non-autosomes and 124038 monomorphic SNPs, this set of SNPs was further pruned to a reduced set of SNPs in order to limit strong influence of SNPs clusters). B: Hierarchical clustering of distance between points (PCA coordinates), groups were determined by k-means (k=3).


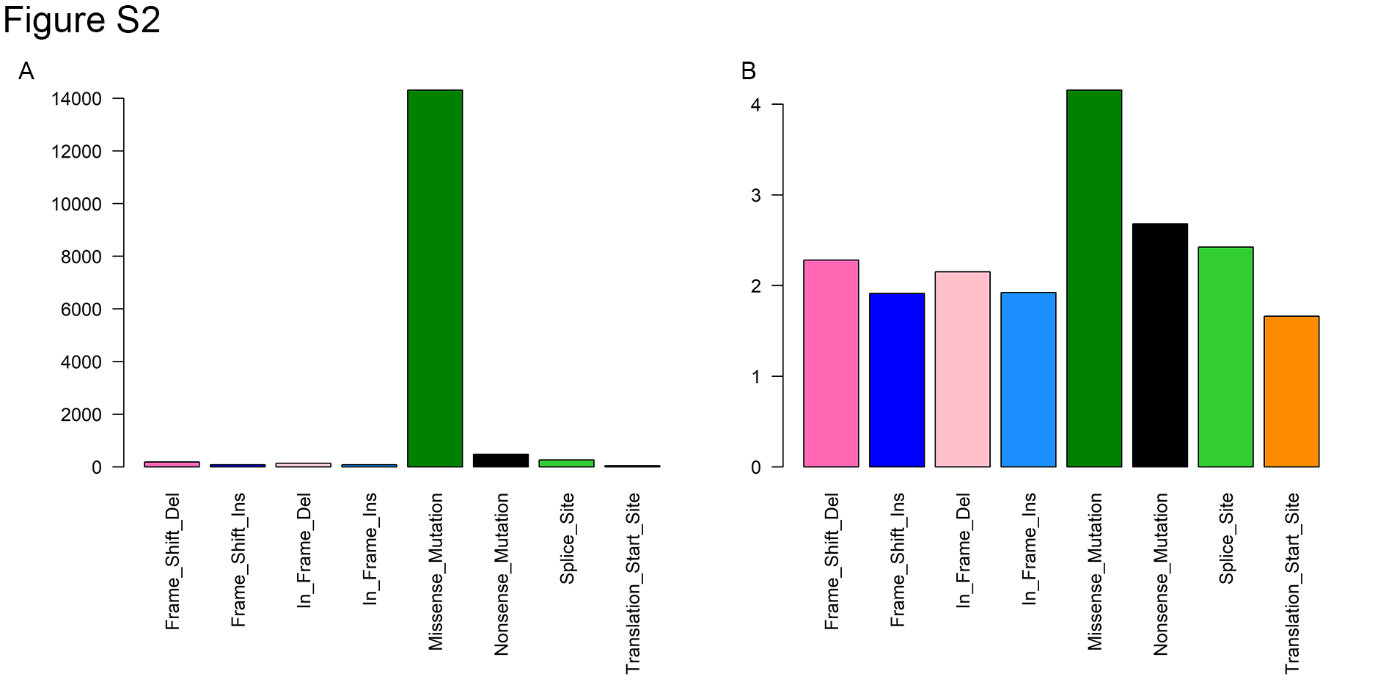


**Figure S2: Mutants Classification according to Variant Effect Predictor**. A: absolute count of abnormalities. B: Log10 transformation of absolute count of abnormalities.


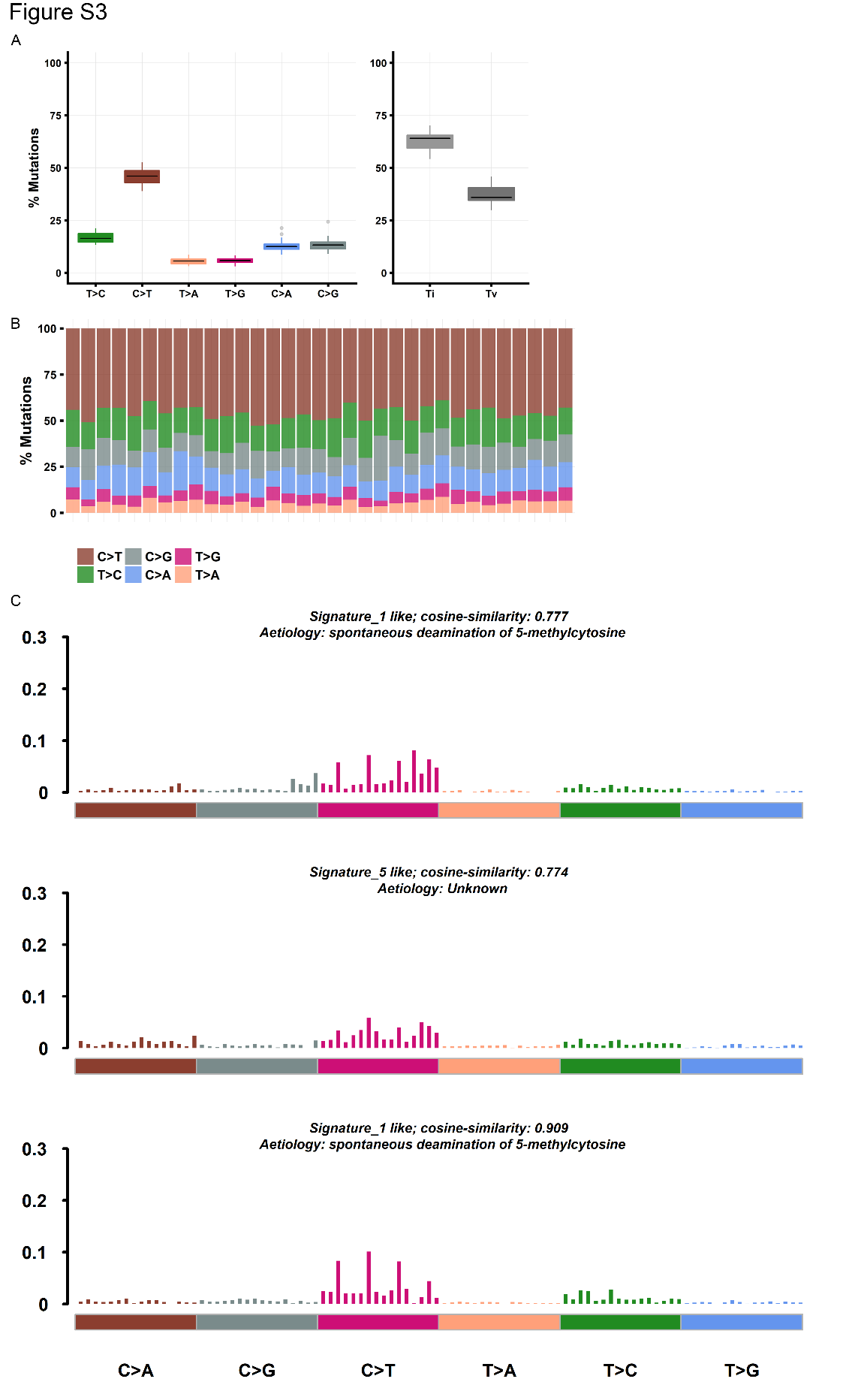


**Figure S3: Genomic signatures.** A: base change and global ratio of Transitions and Transversions. B: Base-change by HMCL. C: Mutation signature as assessed by non-negative Matrix Factorization, signature profiles were correlated with known signatures [https:/cancer.sanger.ac.uk/cosmic/signatures](https://cancer.sanger.ac.uk/cosmic/signatures)) derived from Alexandrov *et al.*^12^.

**Figure S4: Lollipop Plots.**

Lollipop plots of considered variants are available online.

Lollipop plots were drawn from maftools R package. Height of lollipop indicates occurrence of variants. Only amino-acid variants are represented.


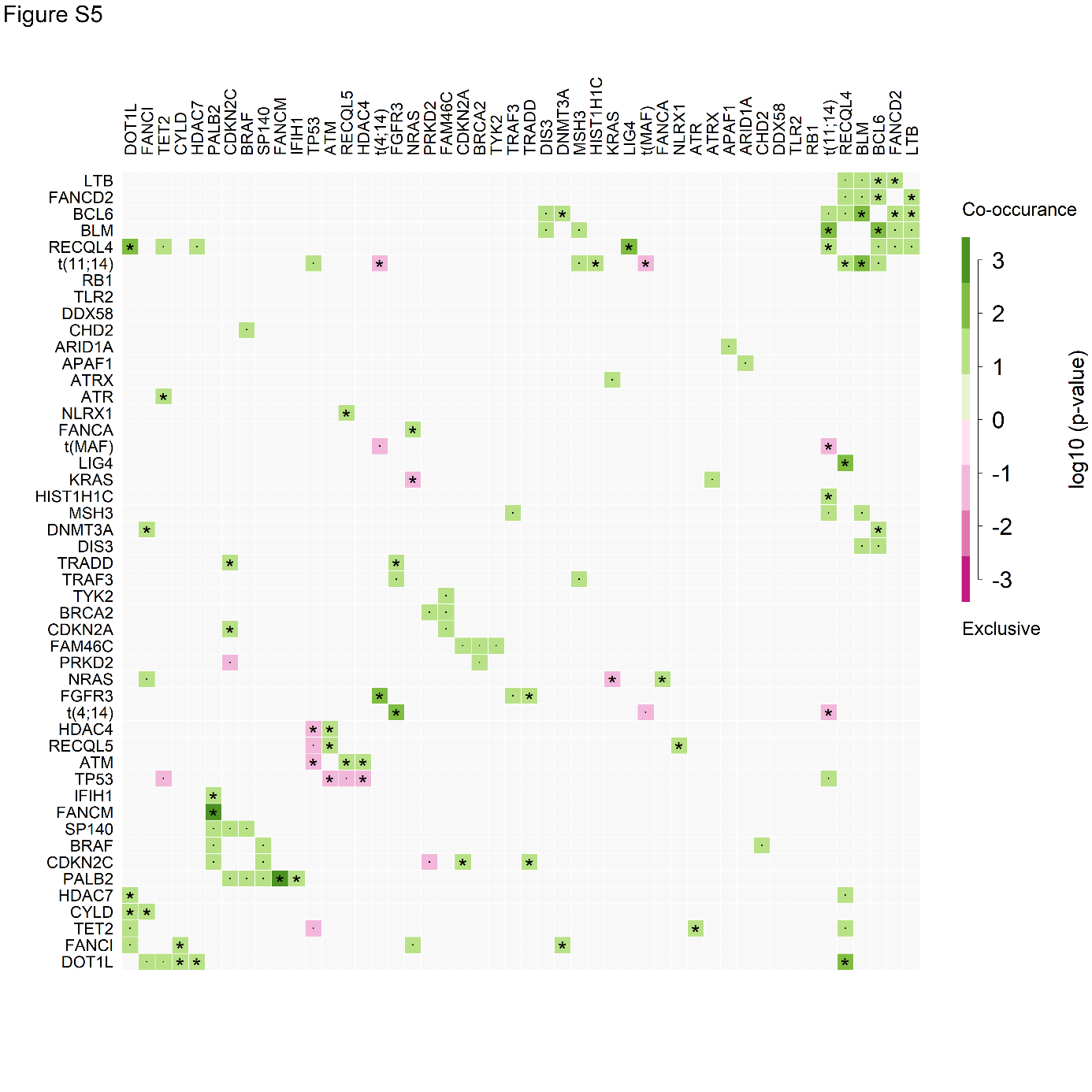


**Figure S5: Somatic Interaction Plot.** Fisher test was performed on most frequent variants (n>2). Only significant associations or exclusivity are displayed. Genes were sorted according to interactions between genes among HMCLs (adapted from Gerstung *et al.*^13^)*.*

**Figure S6: GO, Reactome and Kegg pathway enrichments.**

1. GO Biological Processes Enrichment


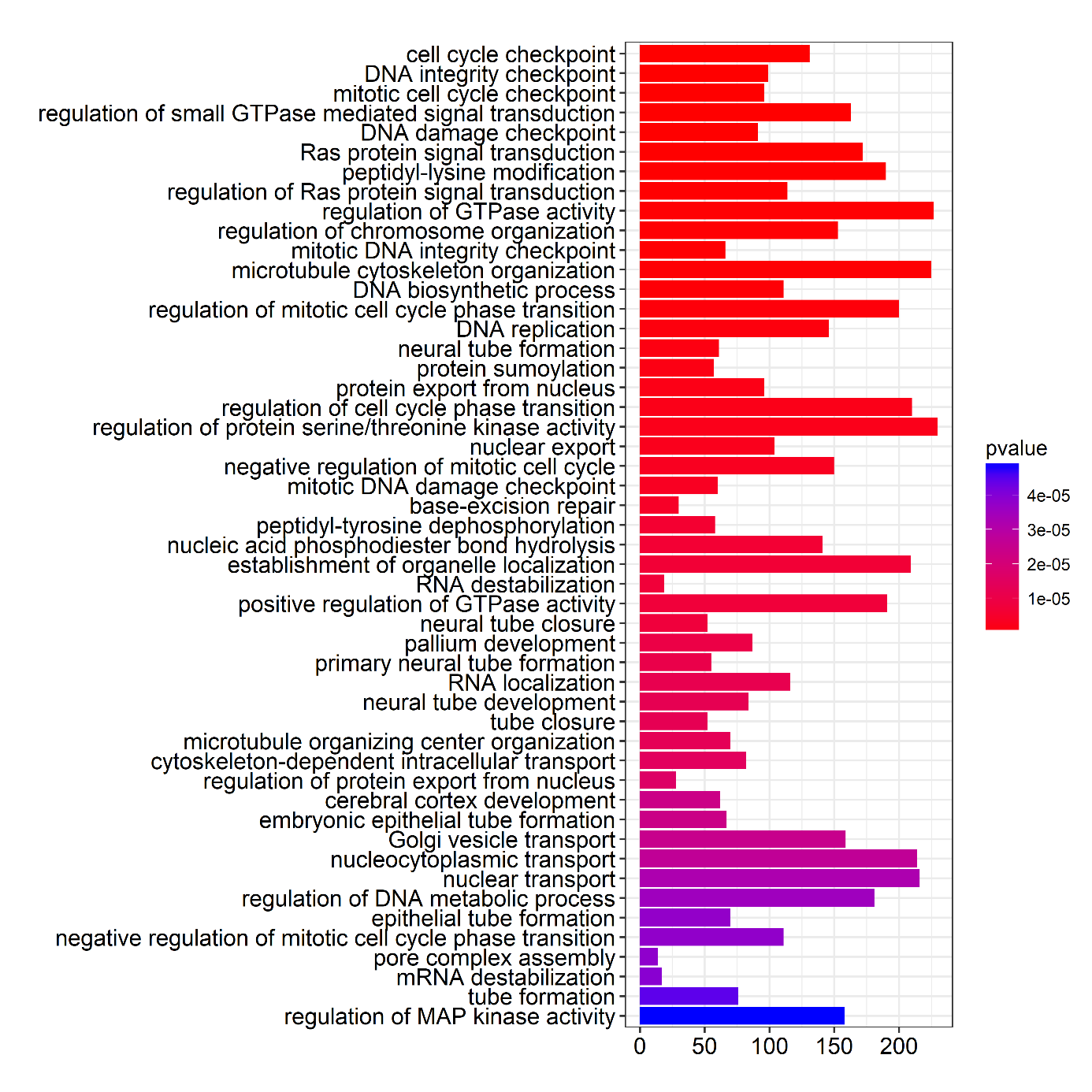


1. GO Molecular Function Processes Enrichment


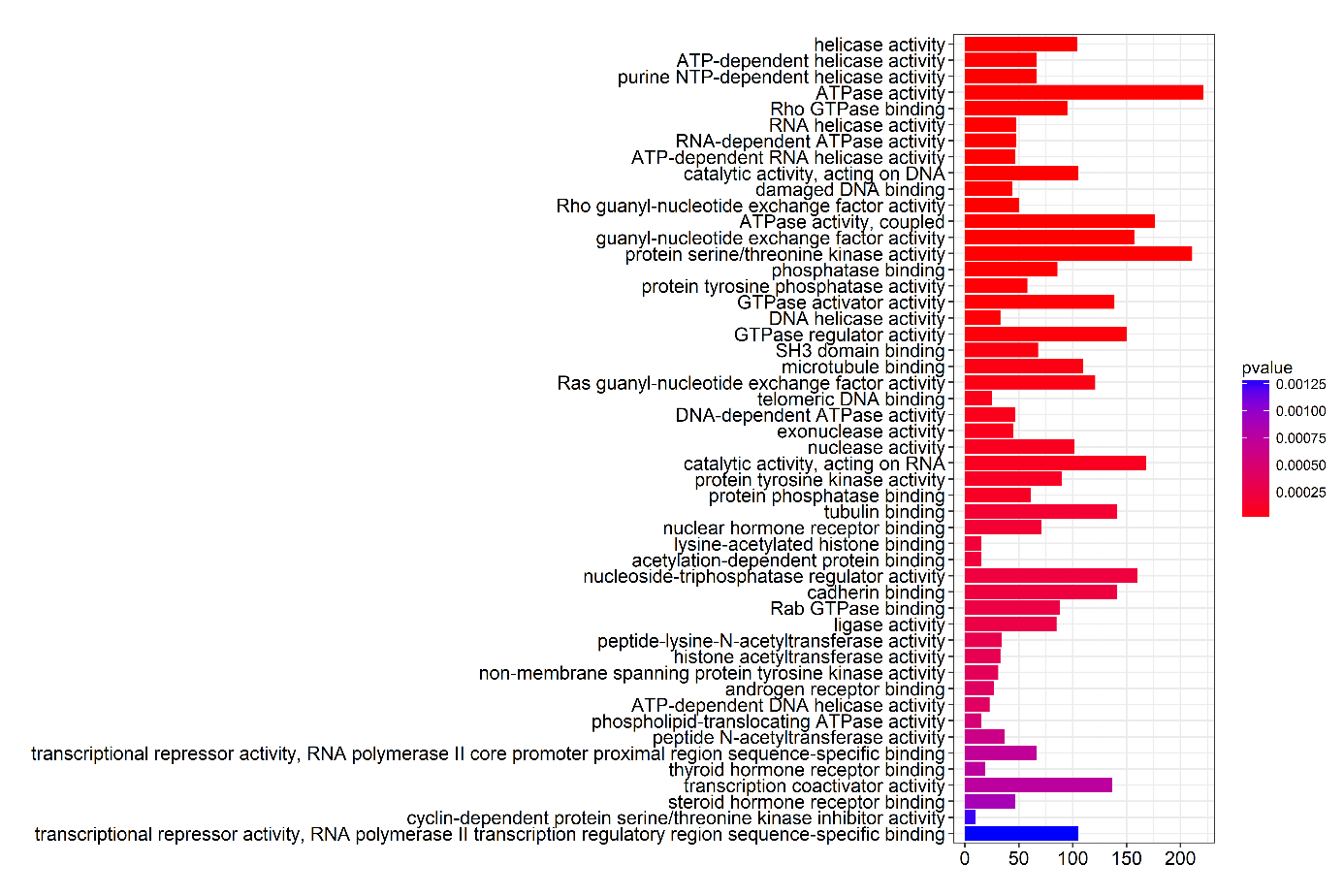


1. GO Cellular Component Enrichment


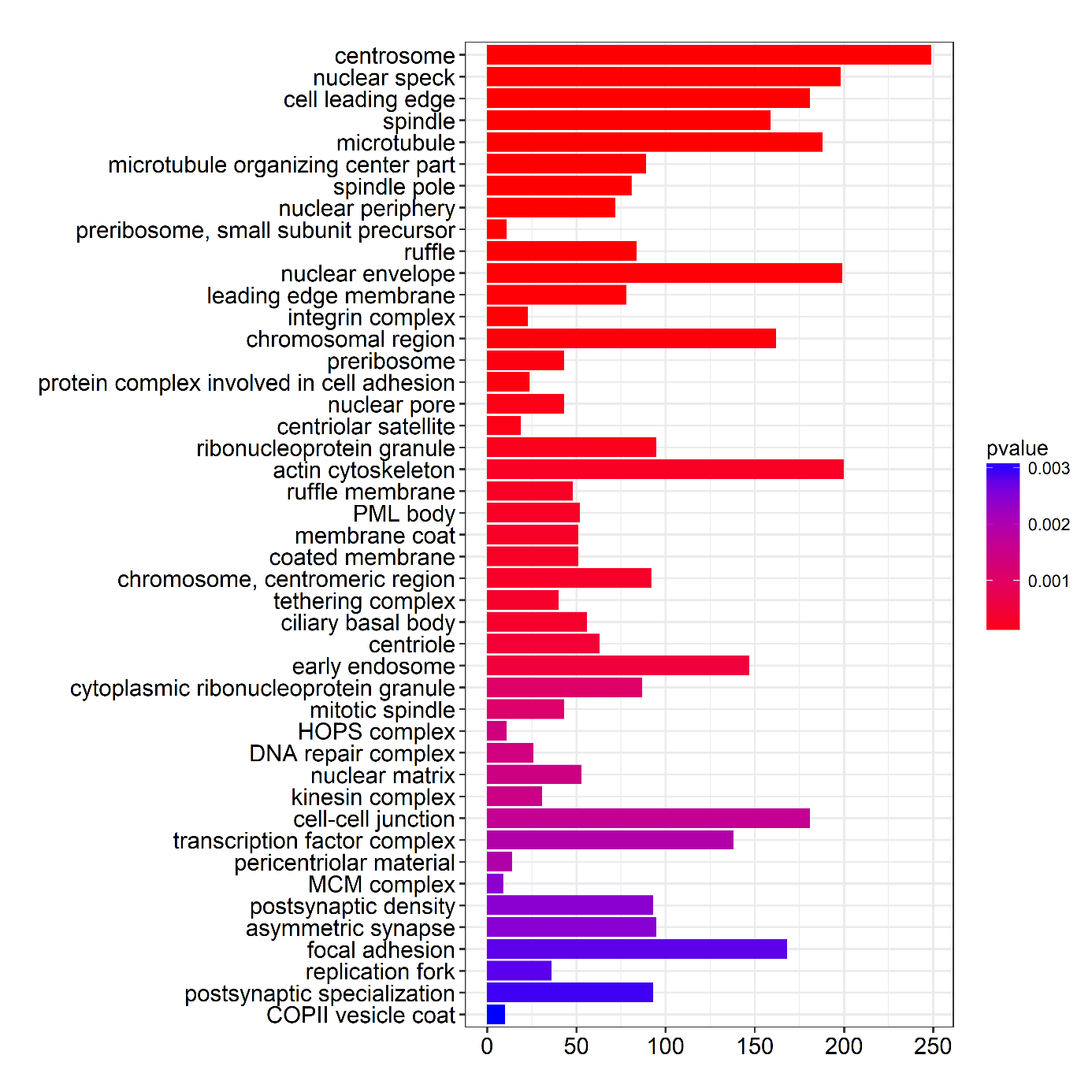


1. Reactome Pathway Enrichment


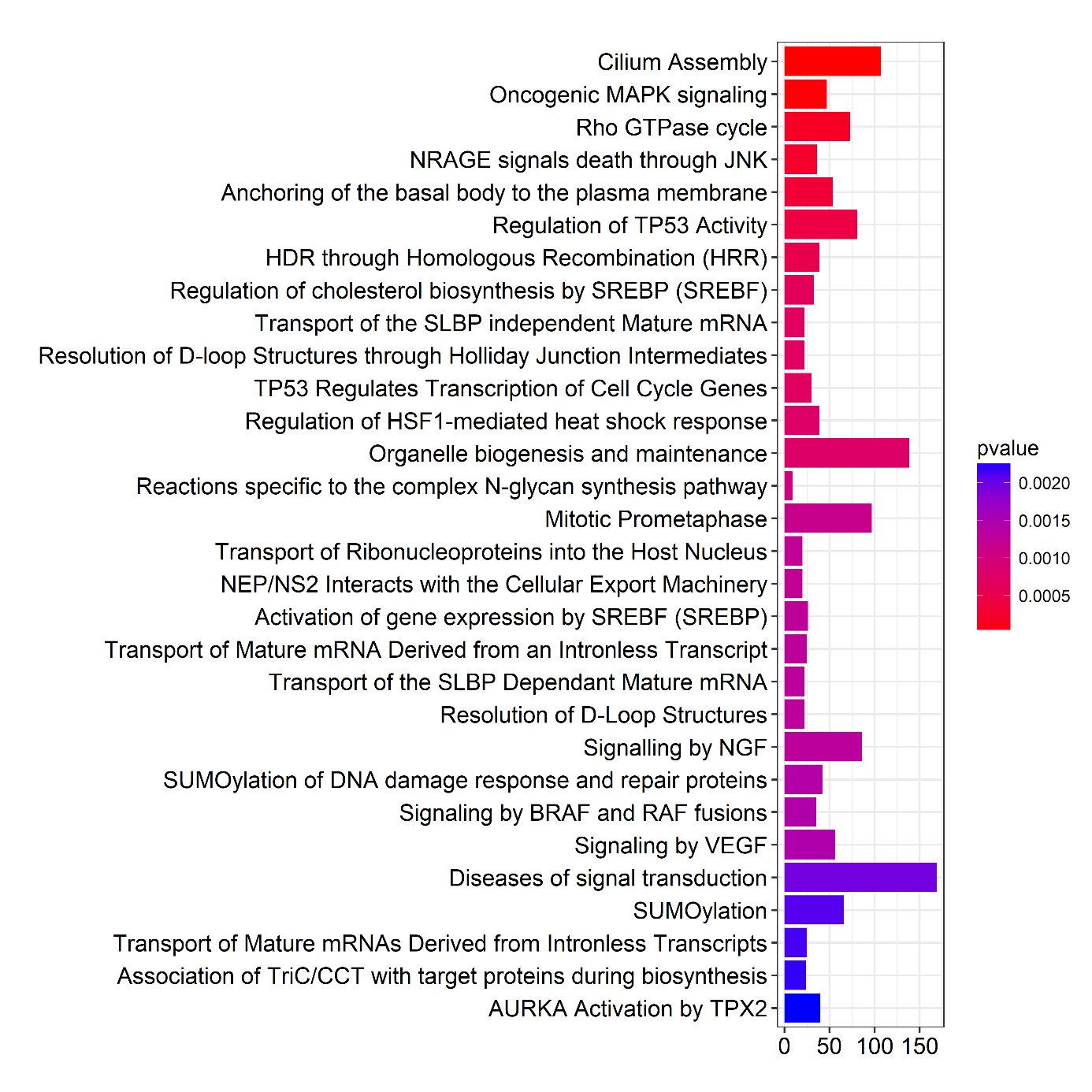


1. Kegg Enrichment


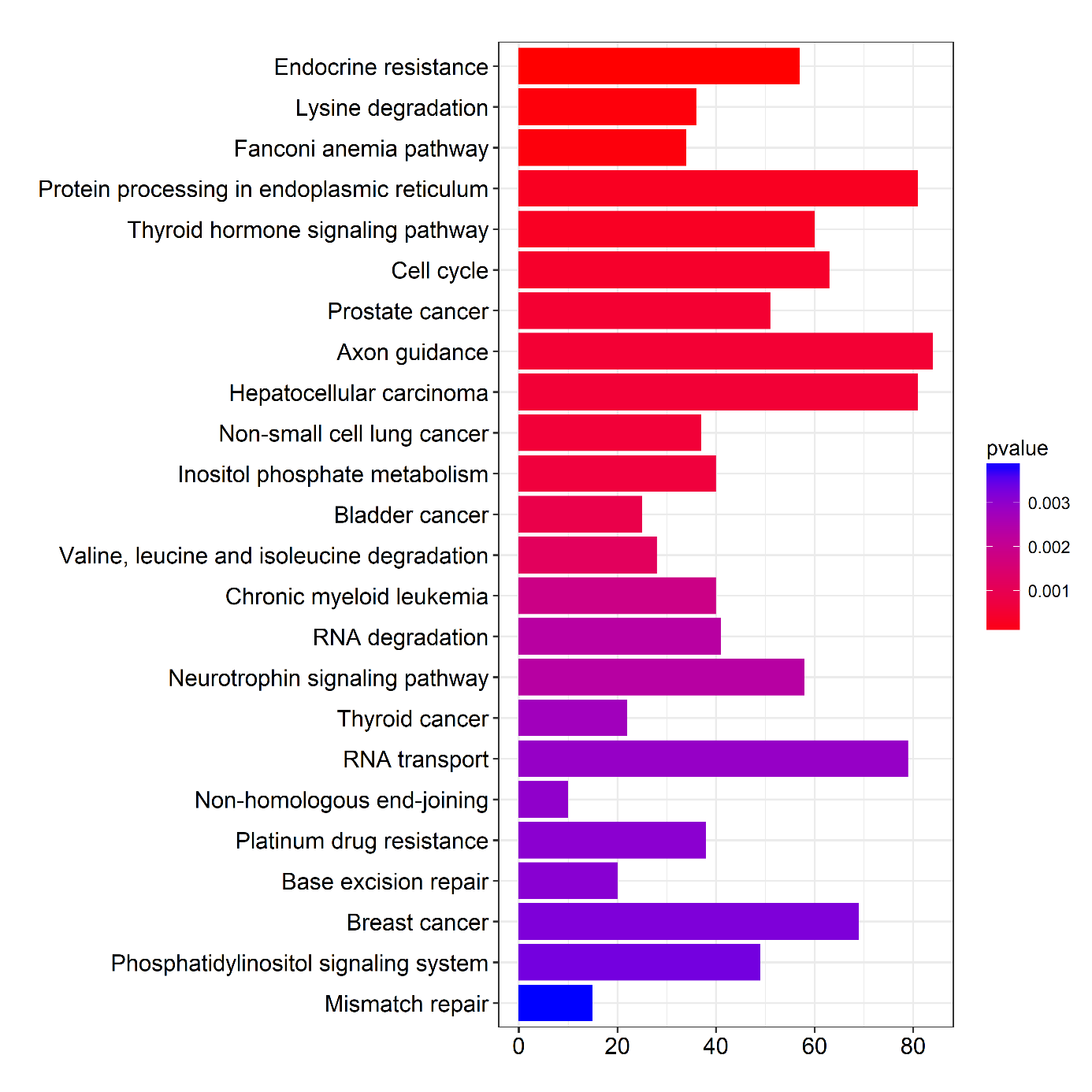


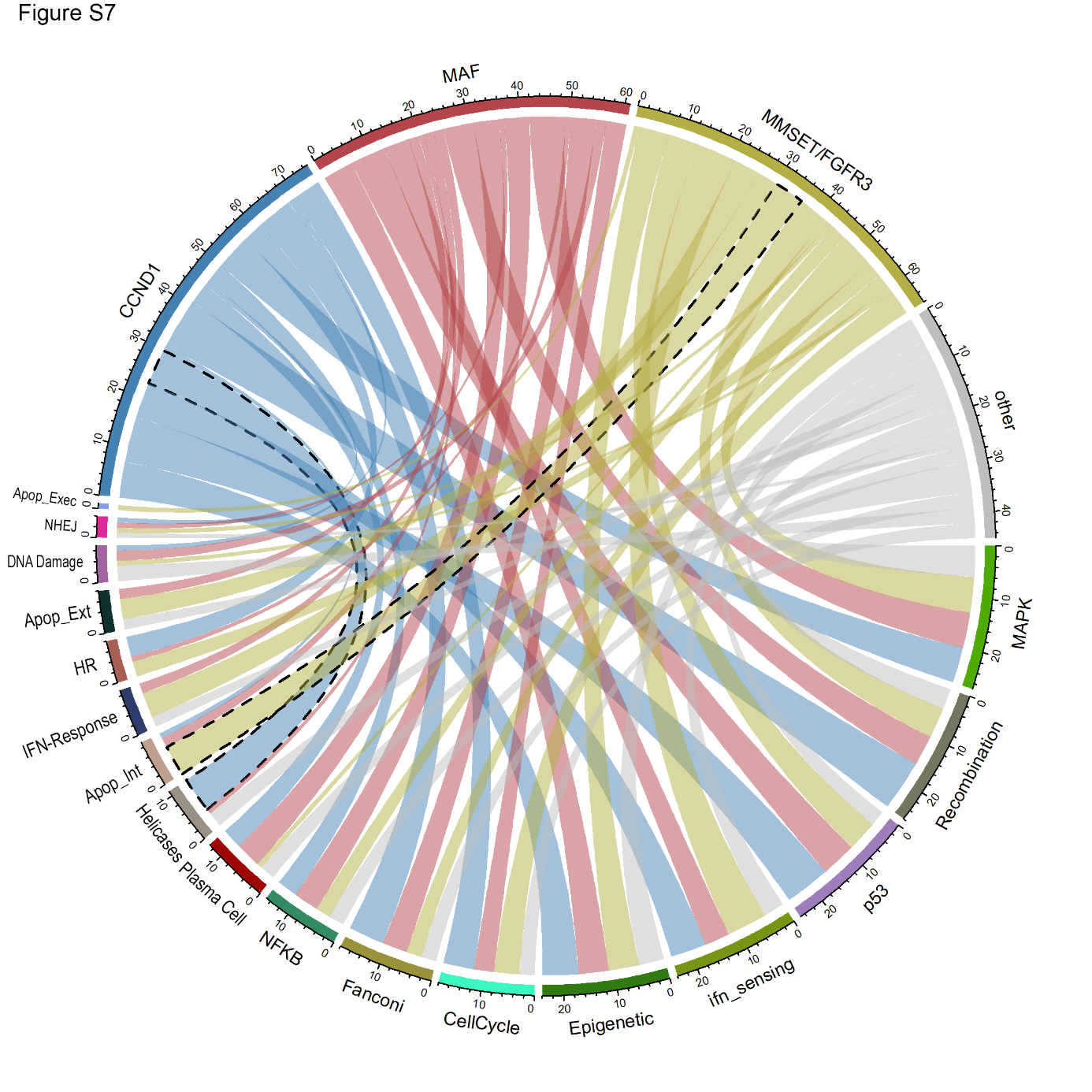


**Figure S7: Chord Diagram of pathway hits relative to recurrent translocation subgroup.** Pathway sizes are proportional to their number of hits. Significant associations are outlined in black.

**Figure S8: Drugs responses according to pathway hits.** Robust linear regression coefficient is displayed; regression line was drawn according to coefficients obtained after 5000 bootstrapped replicates. Only significant associations of tested drugs with the pathways of interest are displayed. Mel= Melphalan, benda= Bendamustine, palbo=Palbociclib, tram2= trametinib, mapa= Mapatumumab, nut= Nultin-3A, Prima= Prima-1^Met^, lexa= lexatumumab, dexa= dexamethasone, poma= pomalidomide, btz= bortezomib, cfz= carfilzomib, a11= A-1155463, a12= A-1210477.


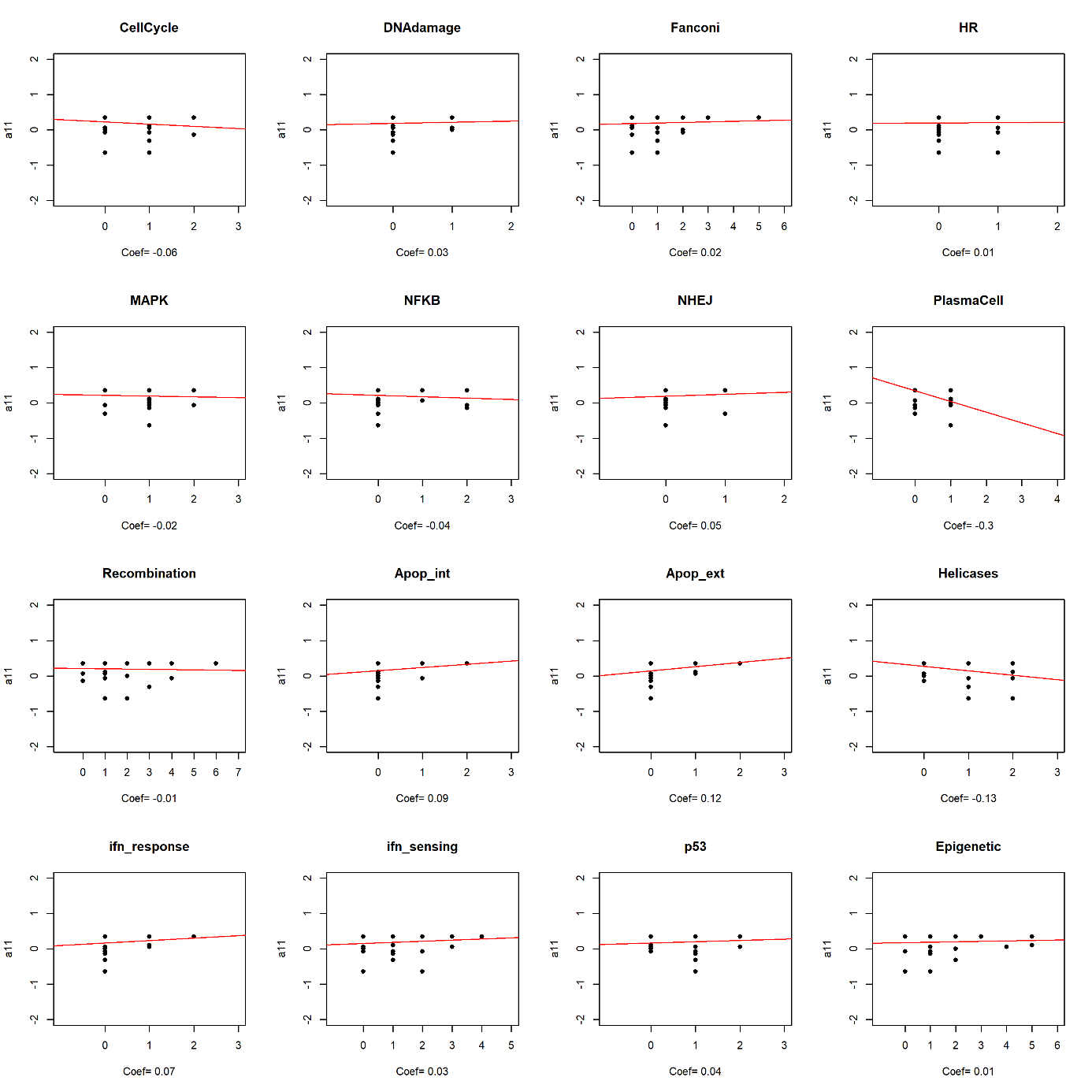


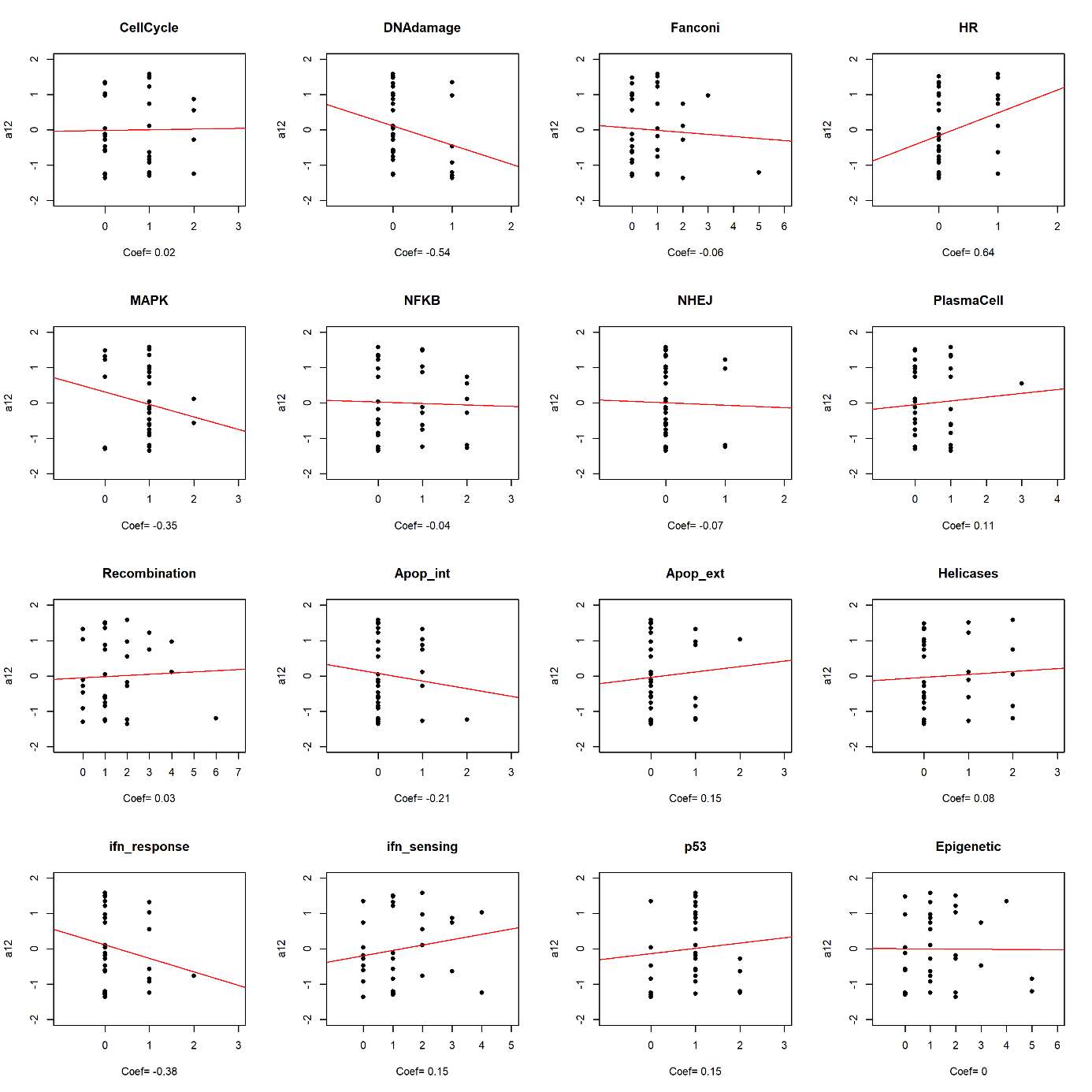


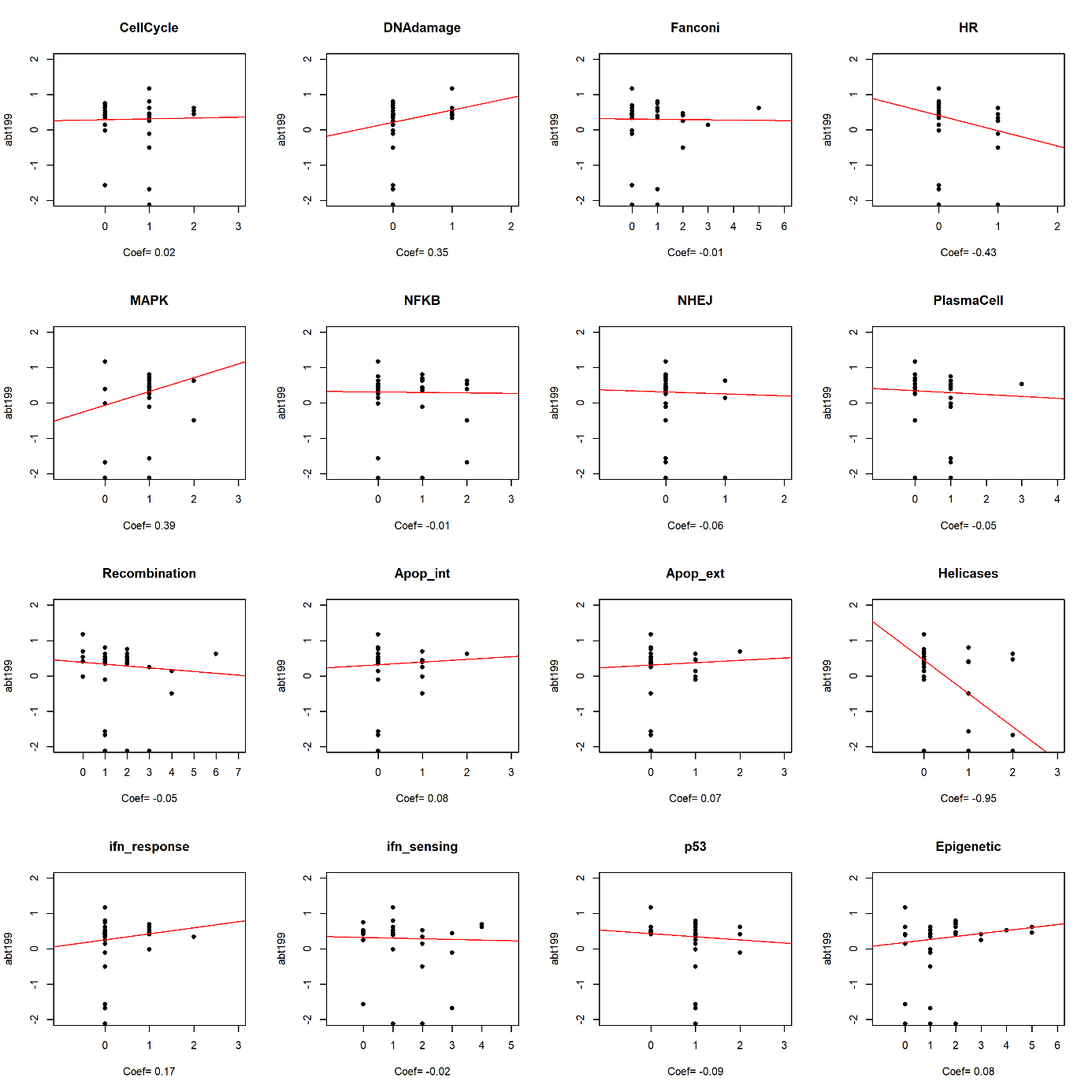


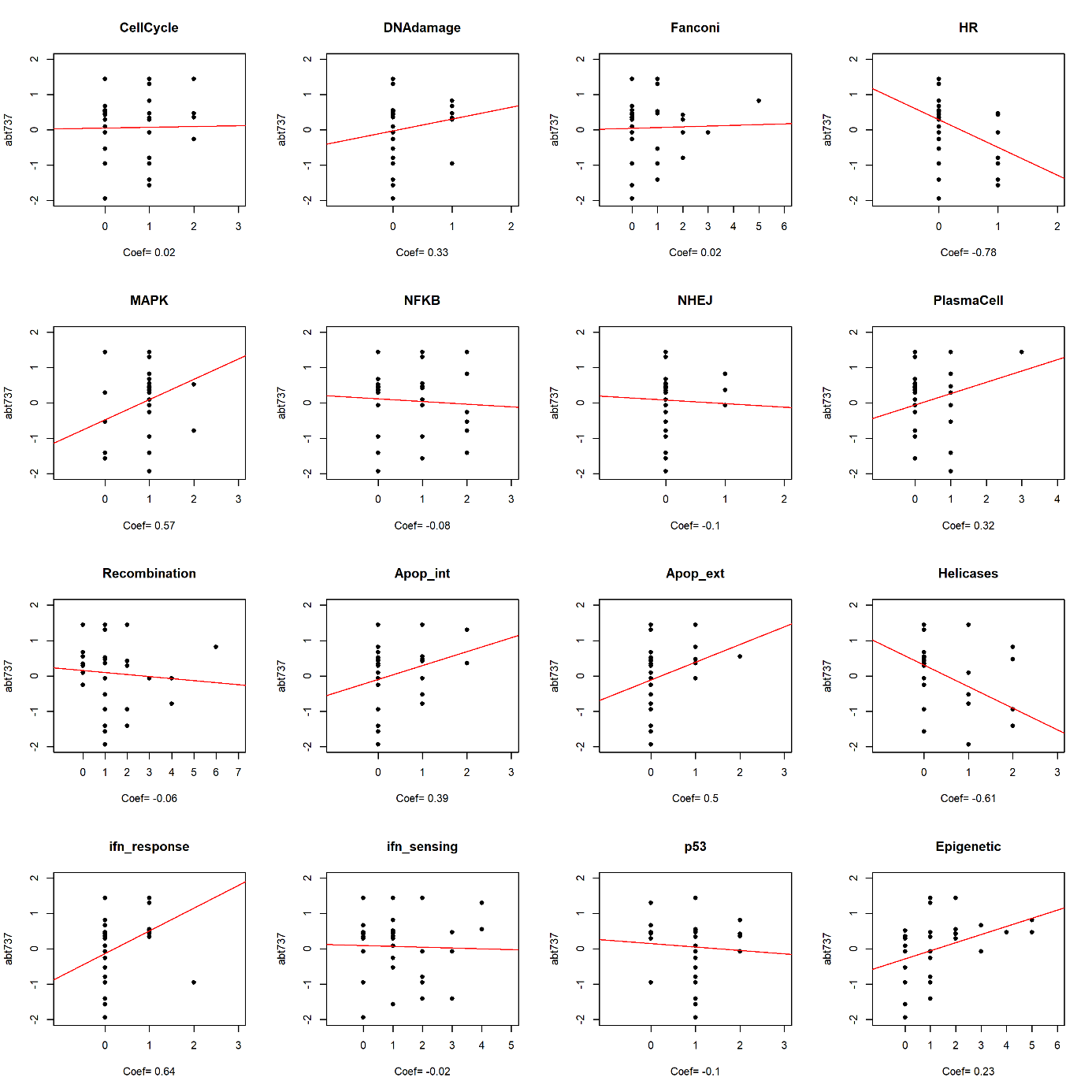


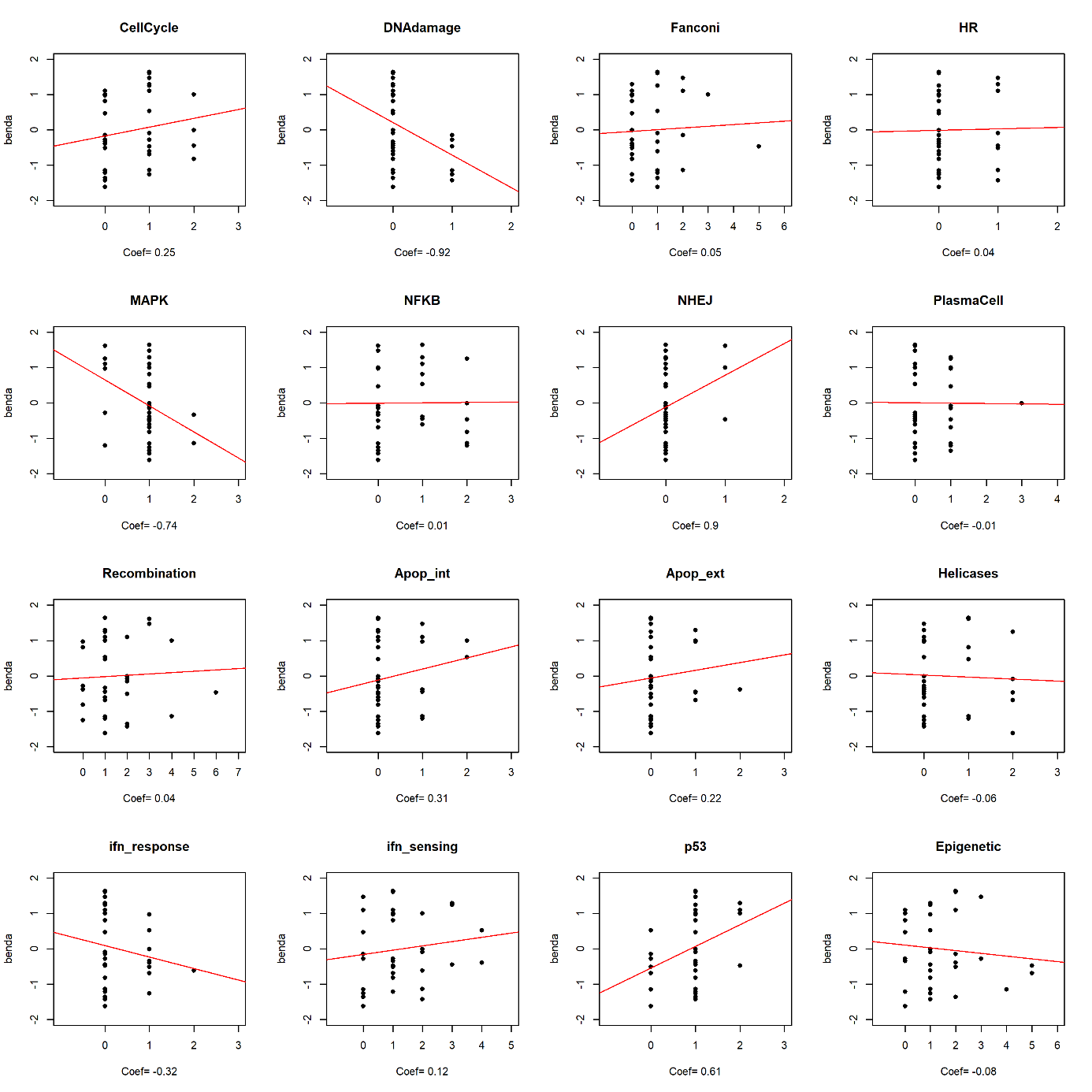


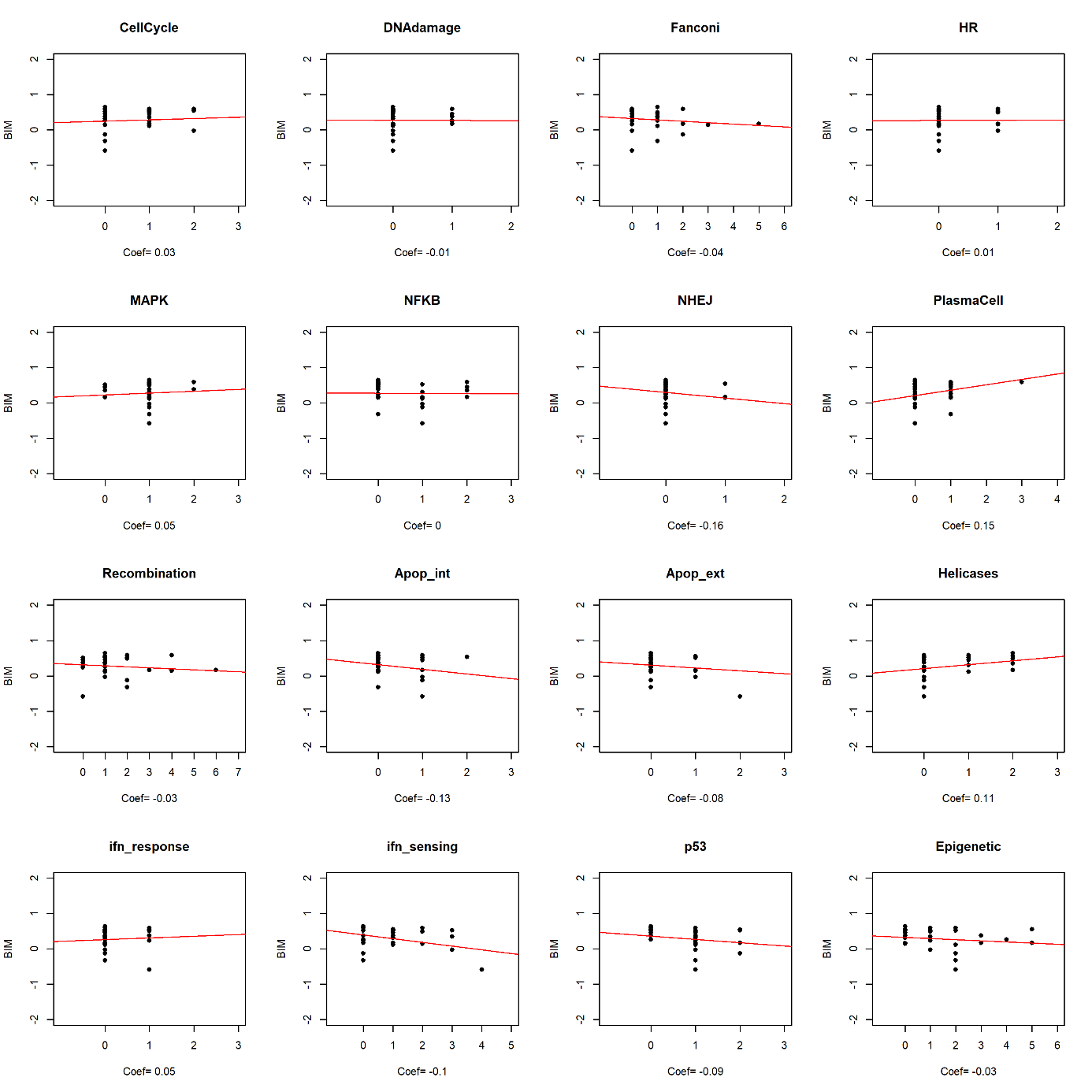


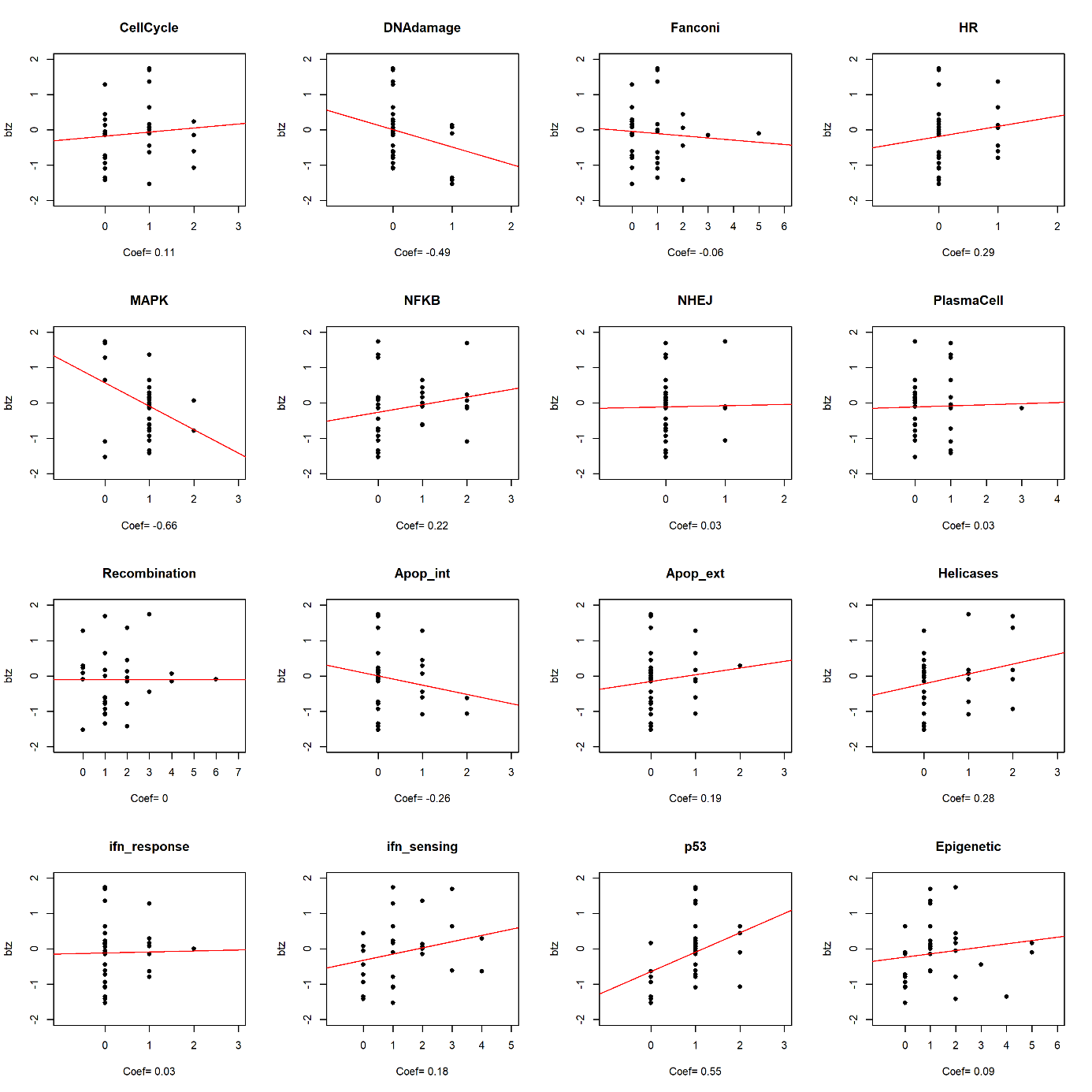


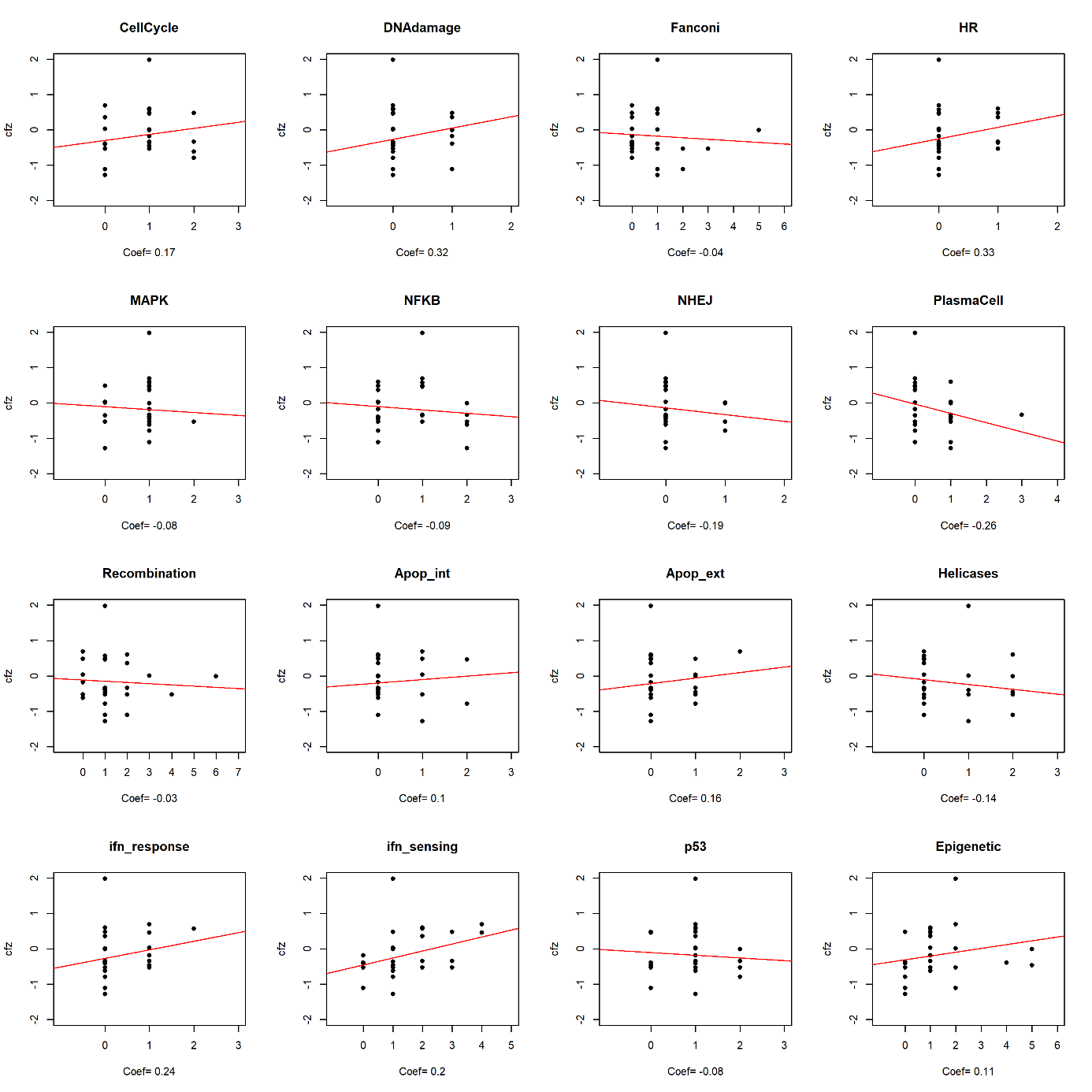


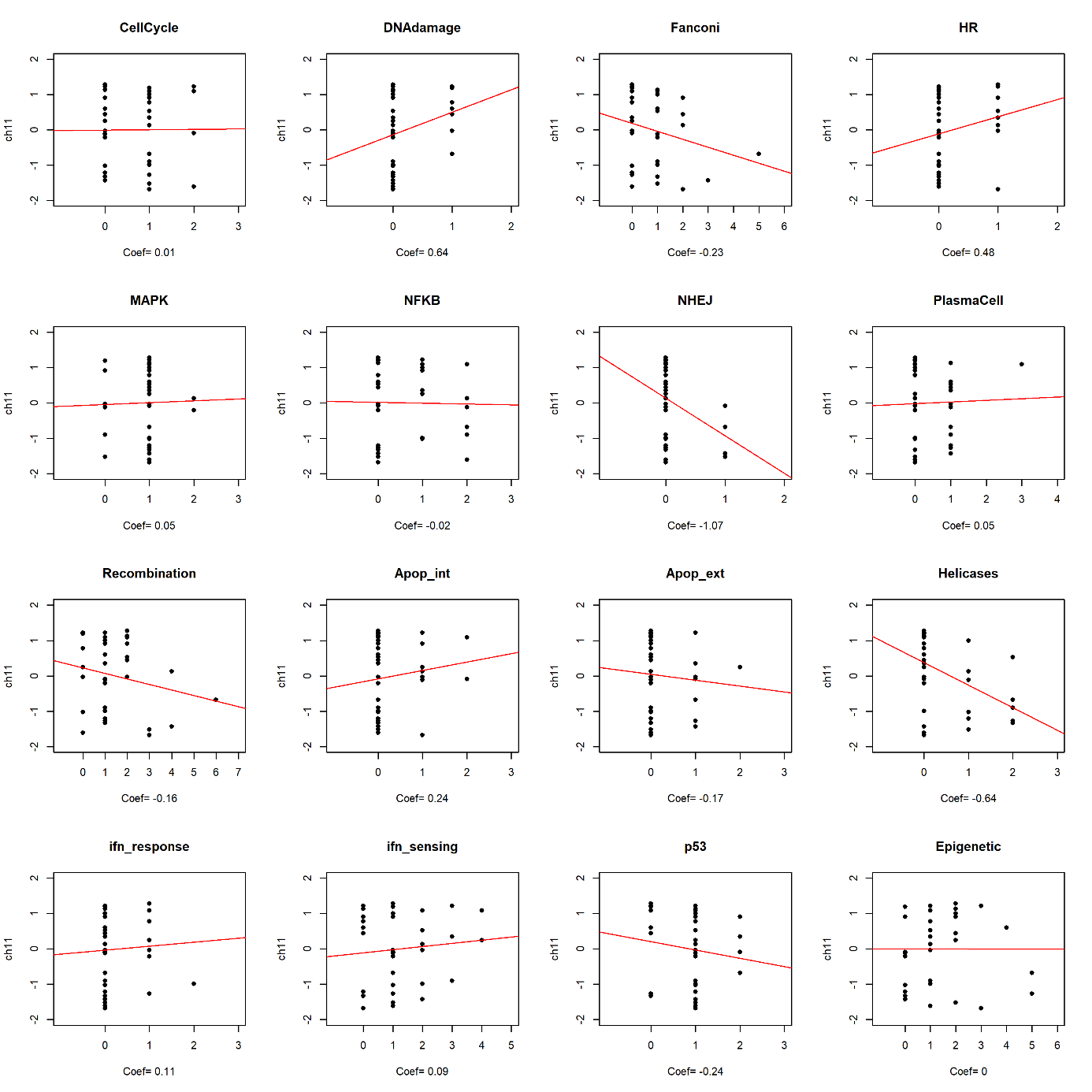


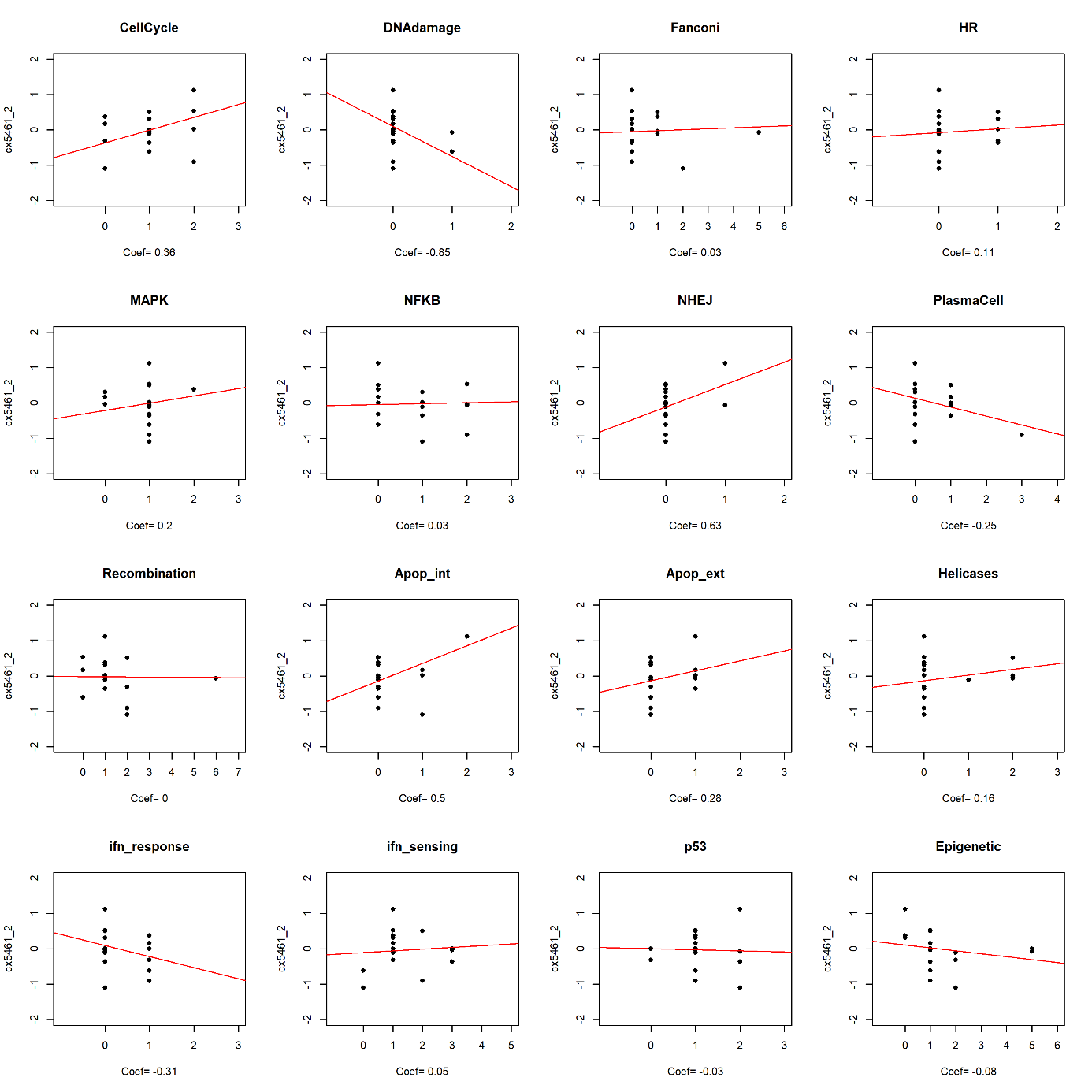


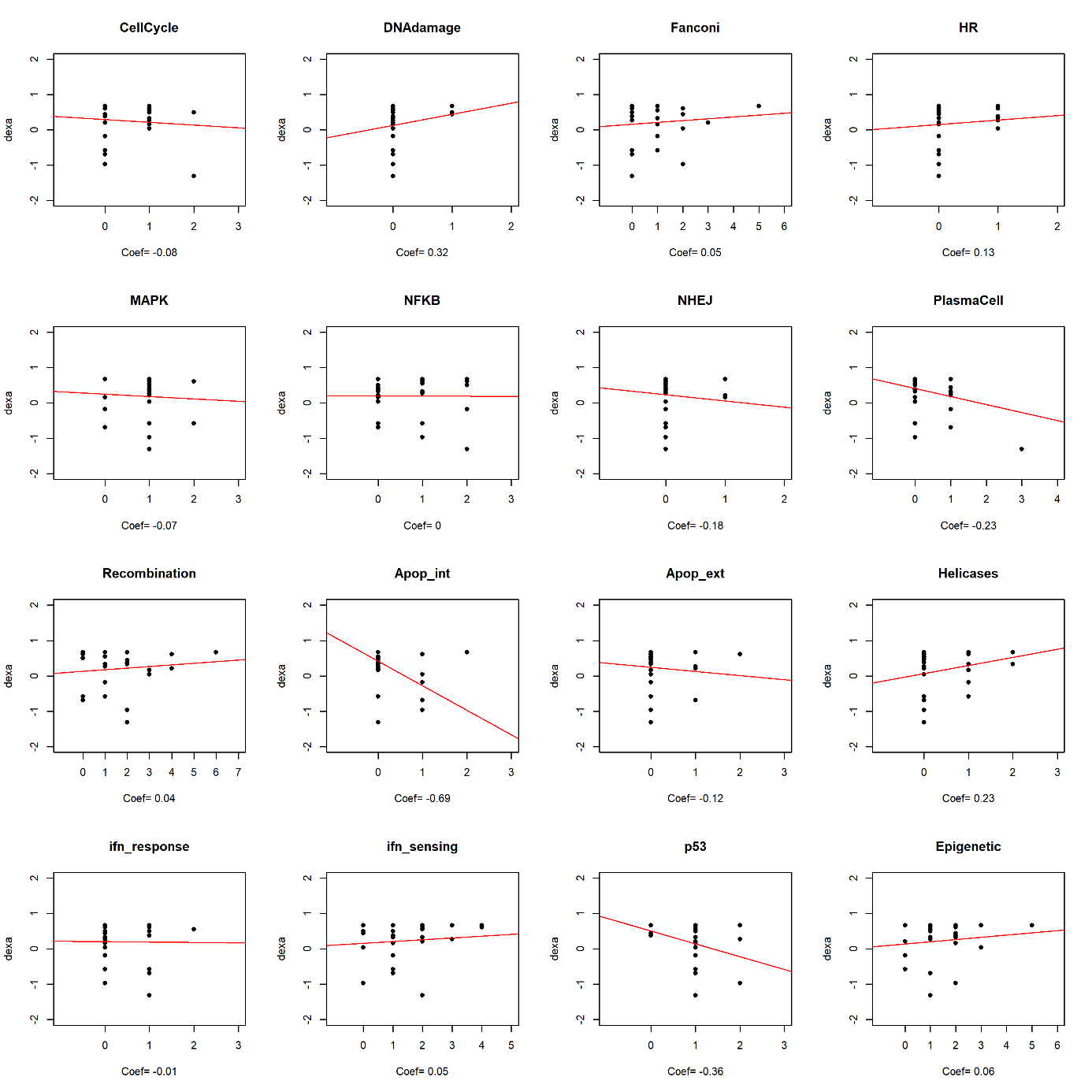


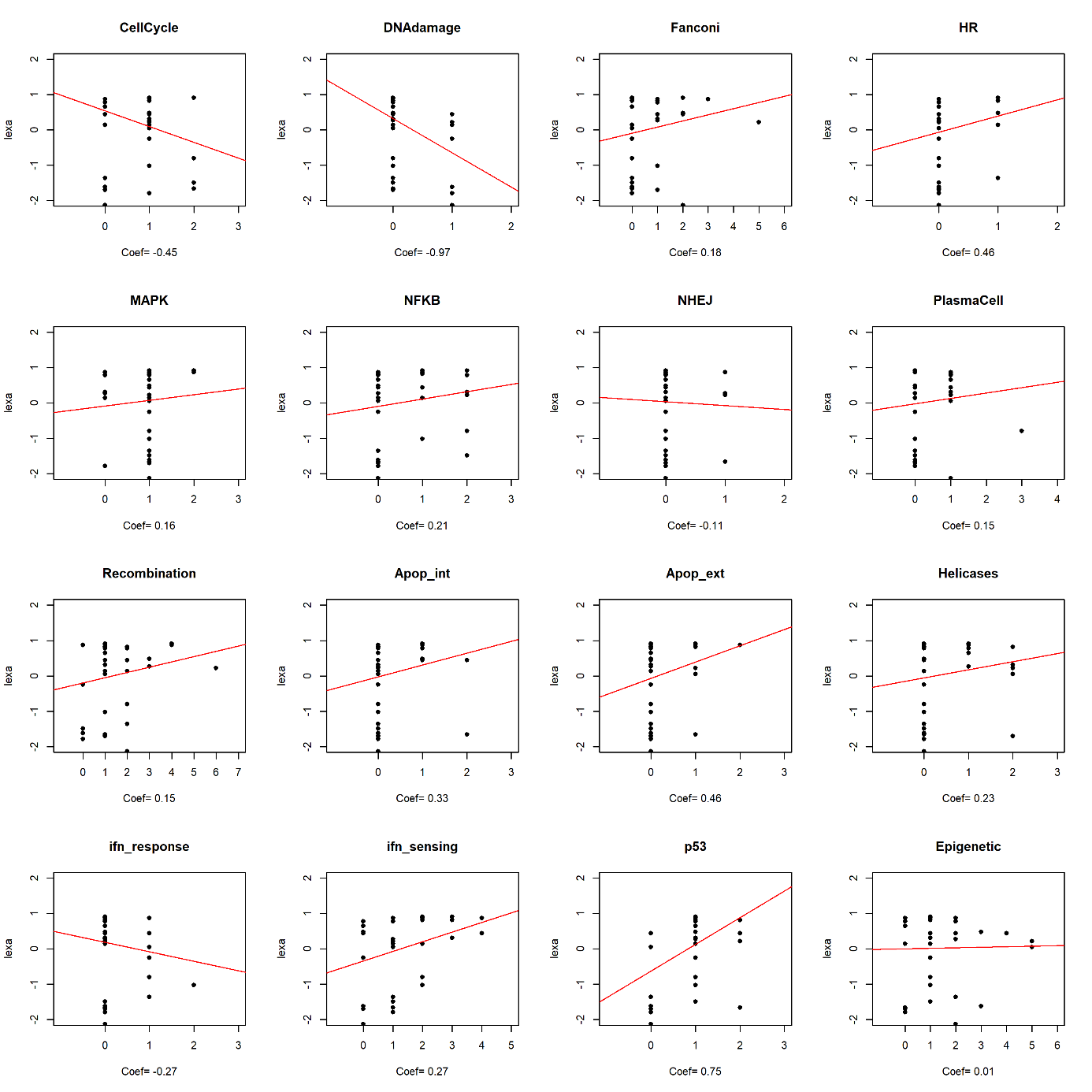


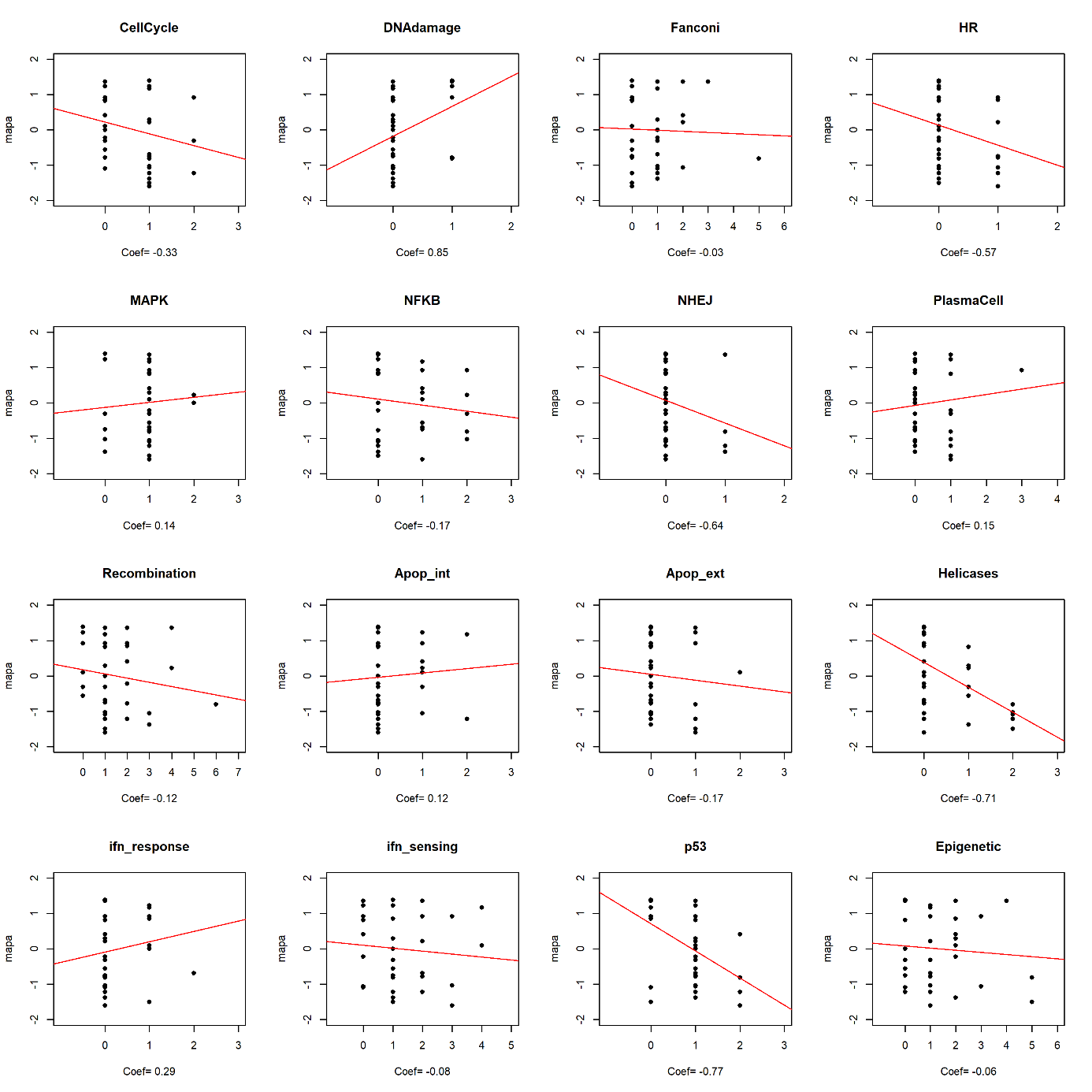


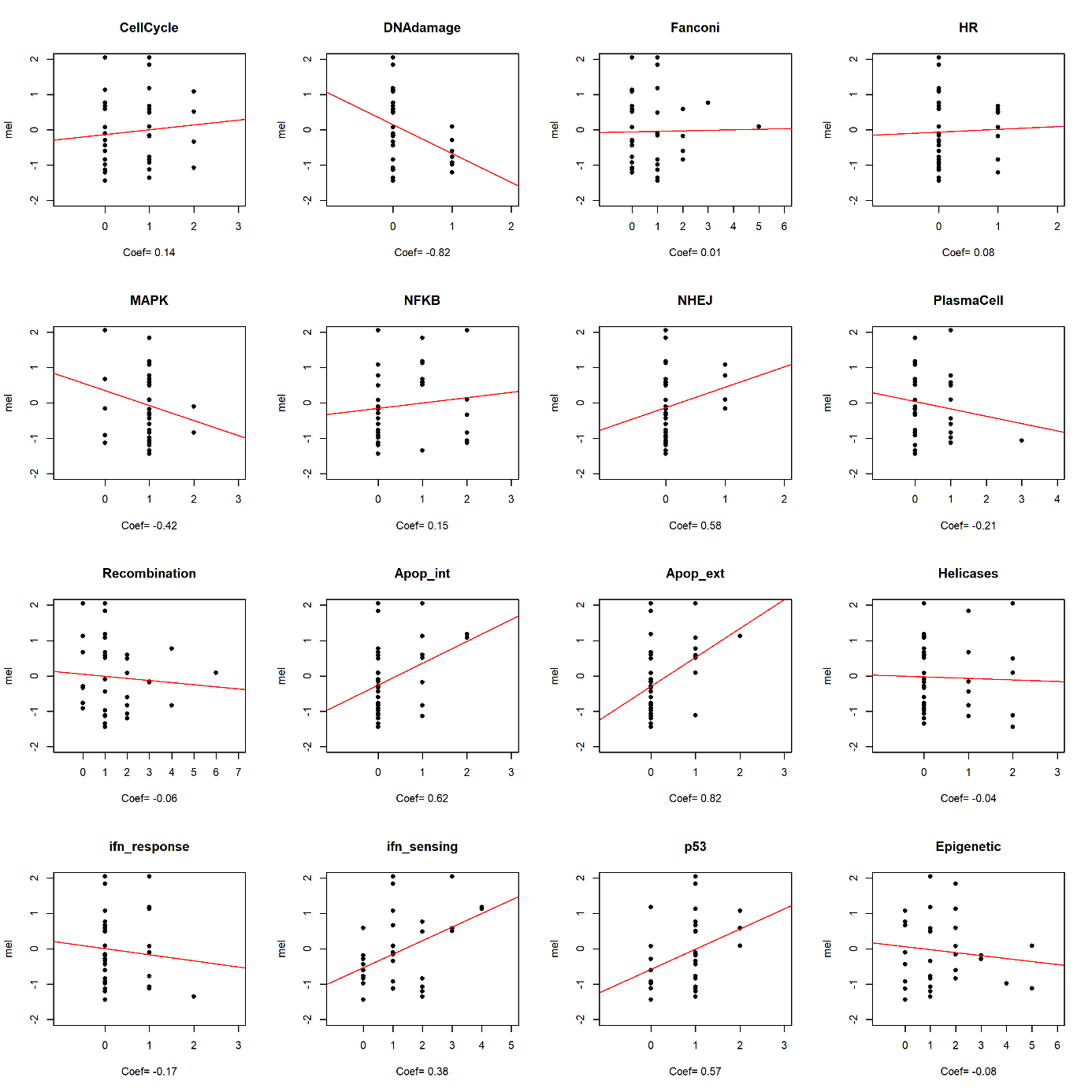


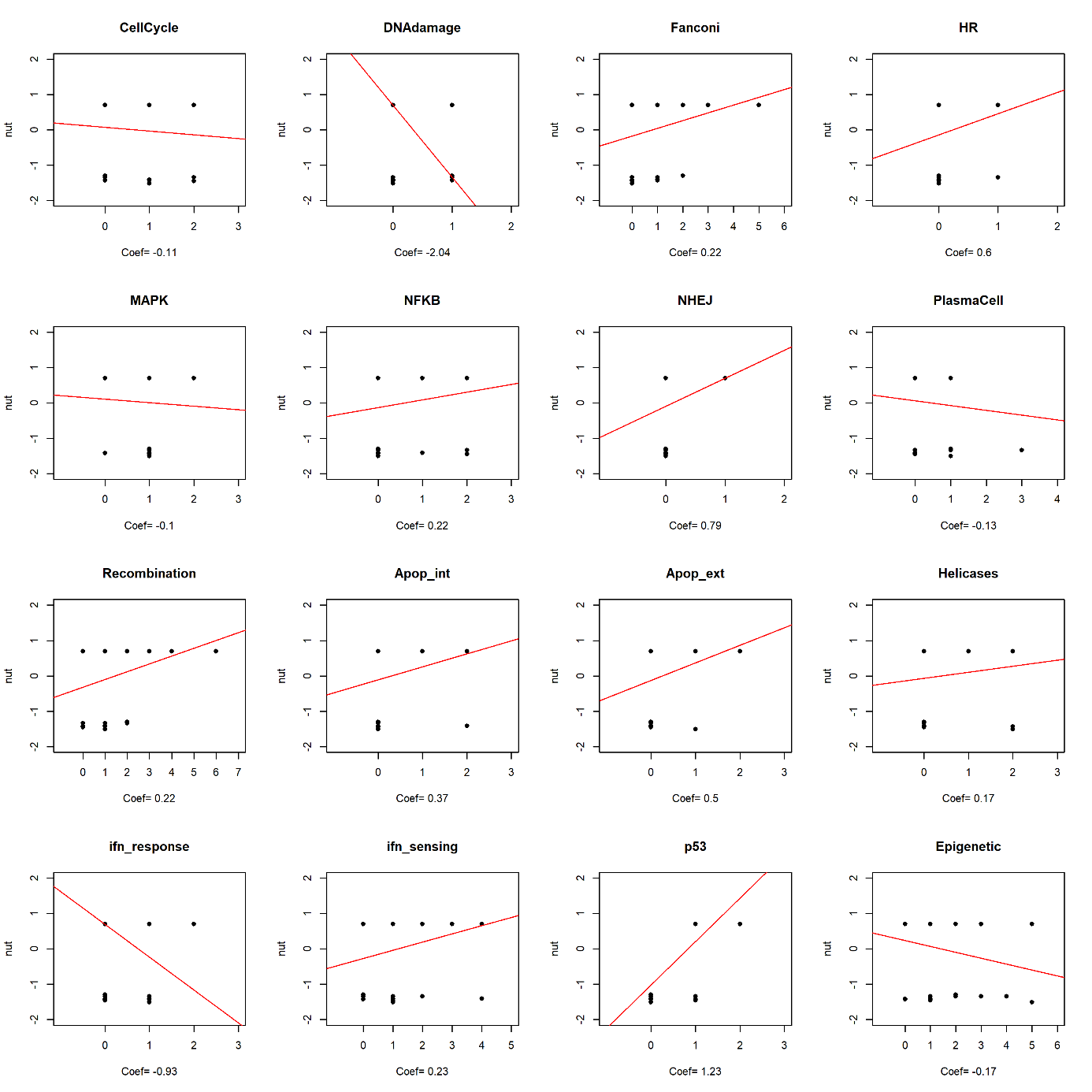


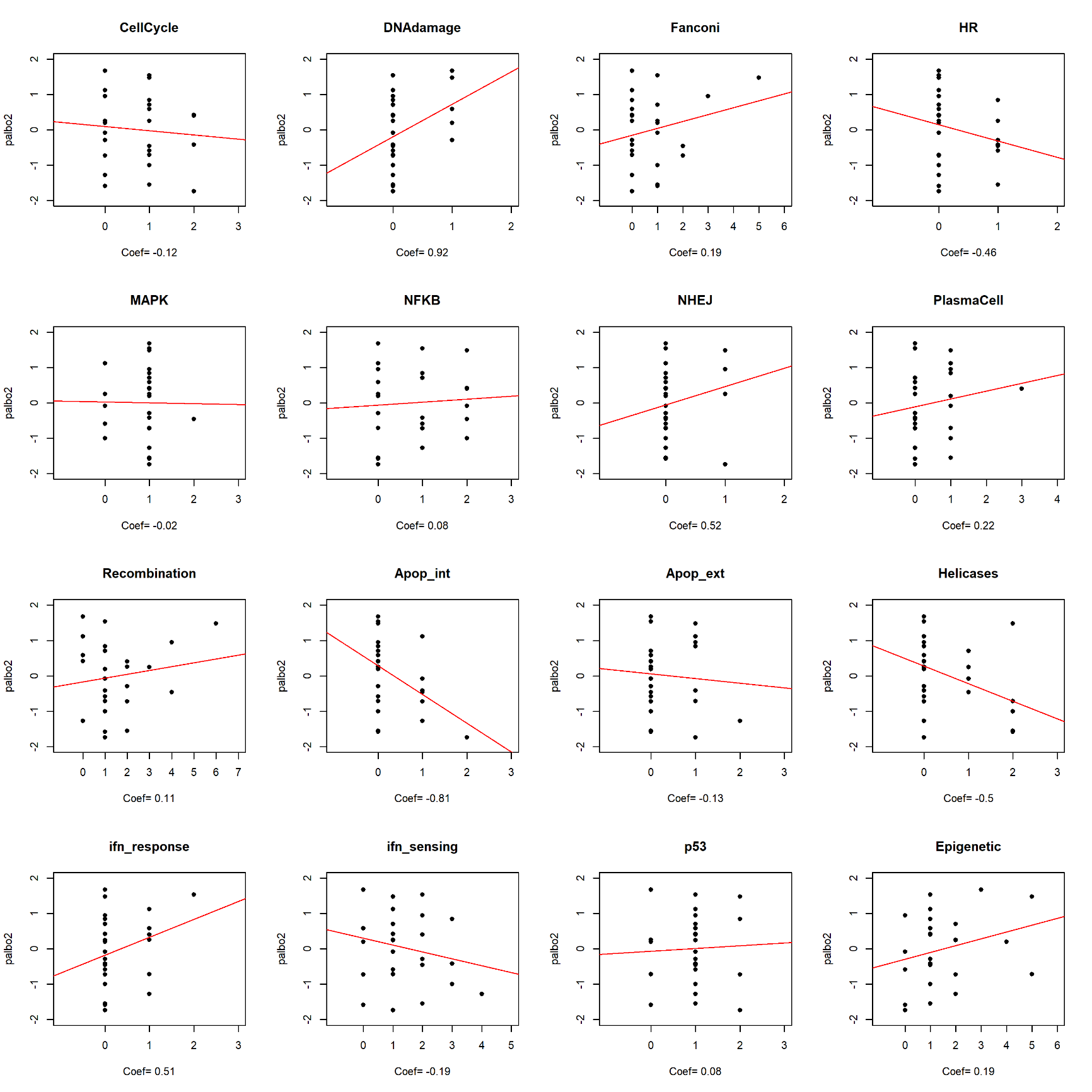


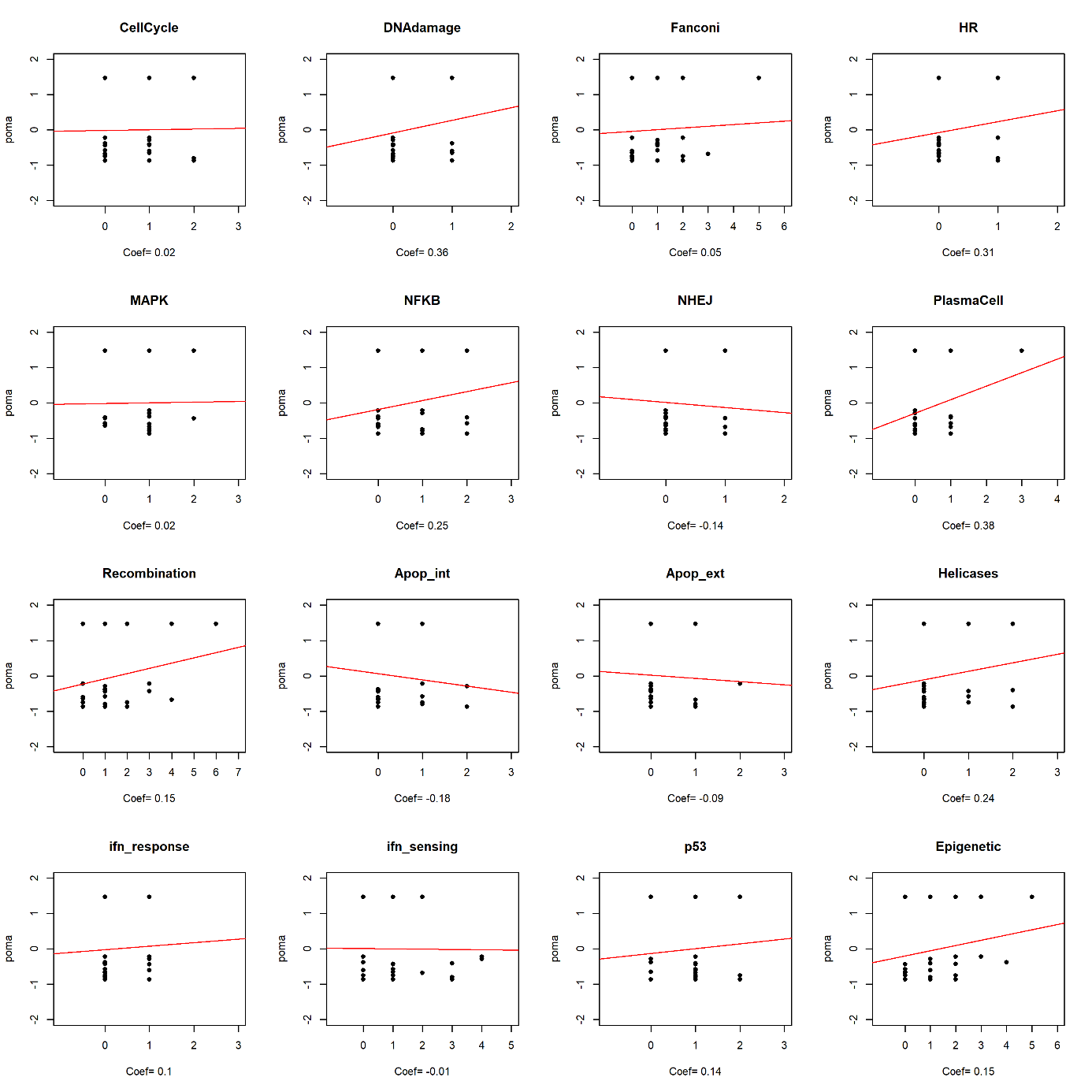


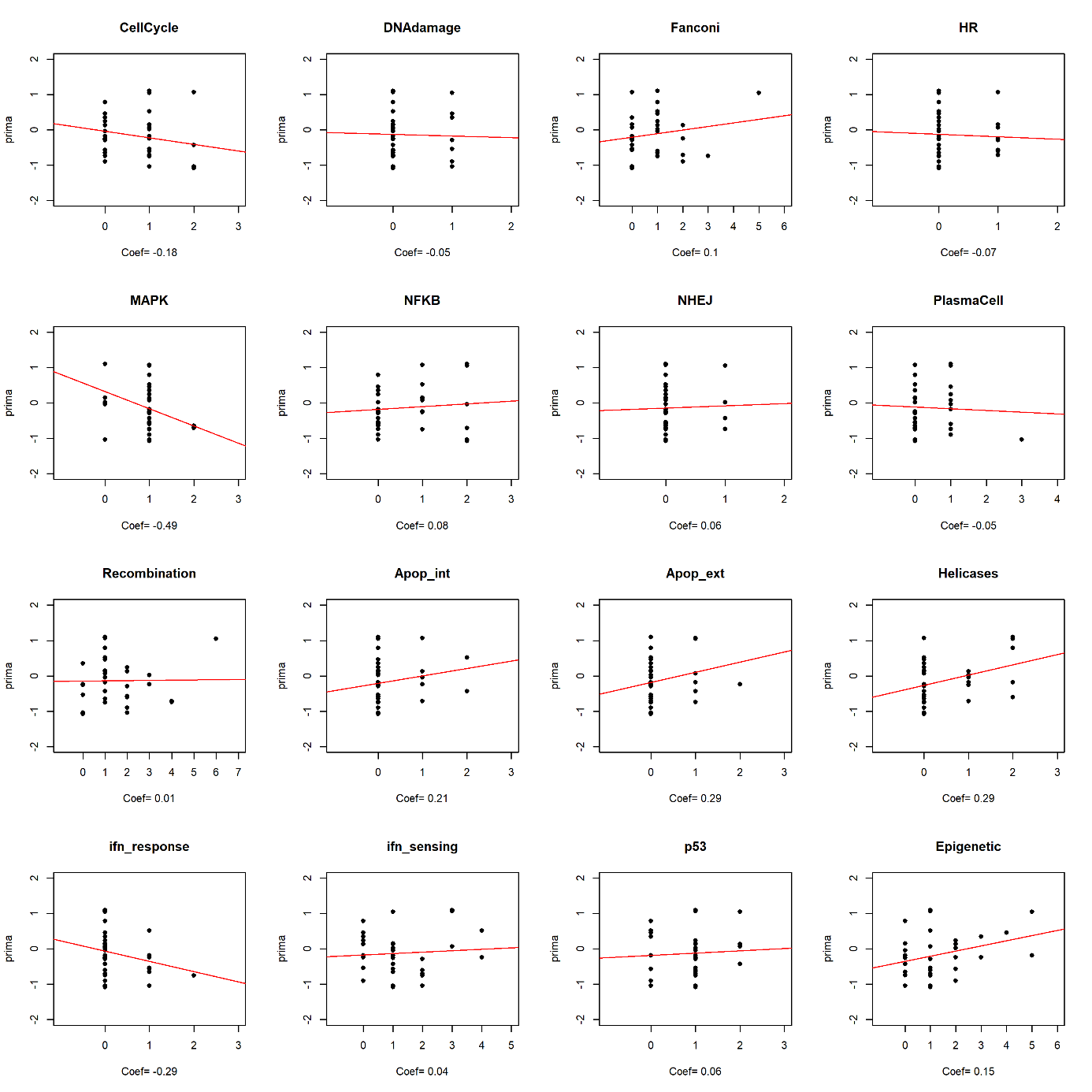


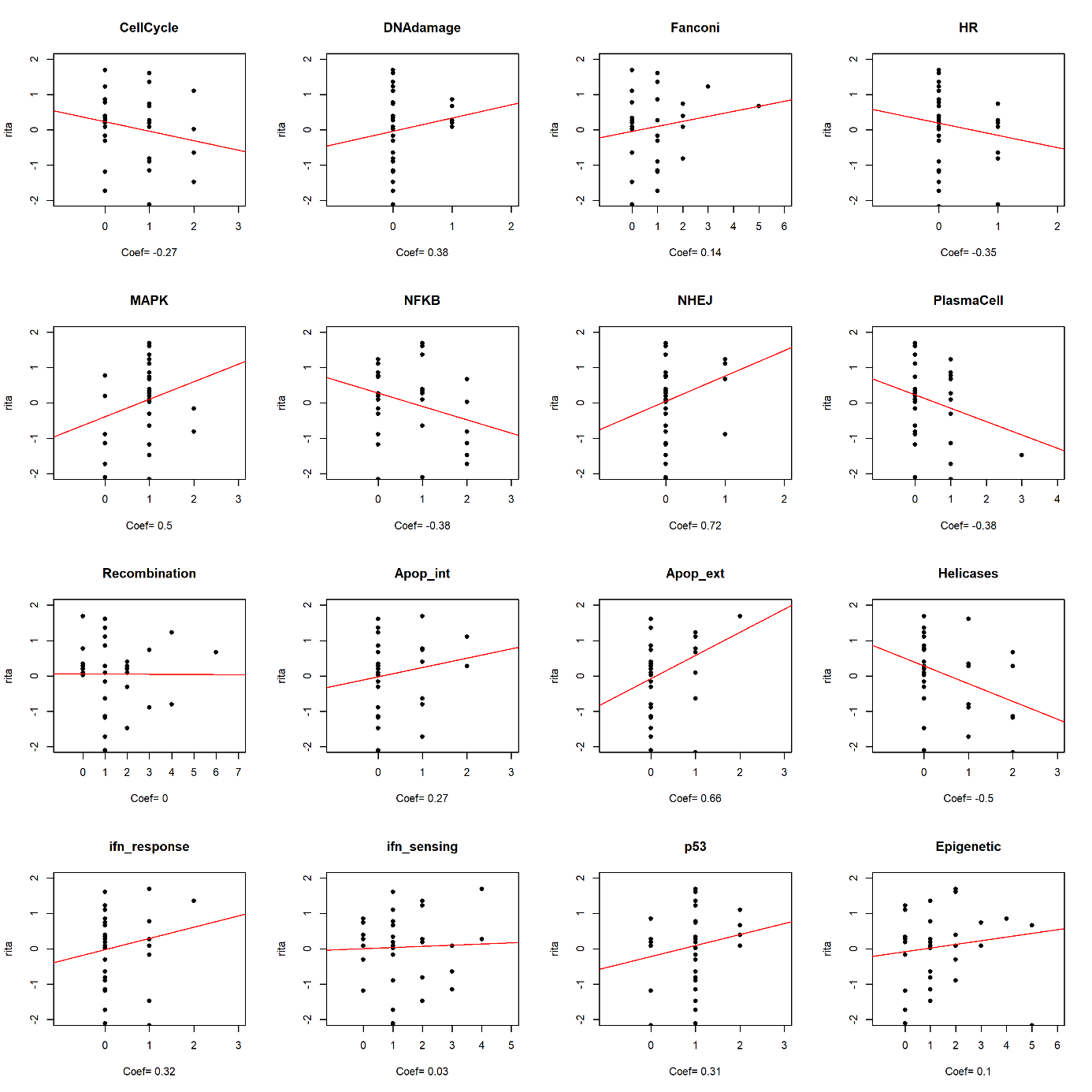


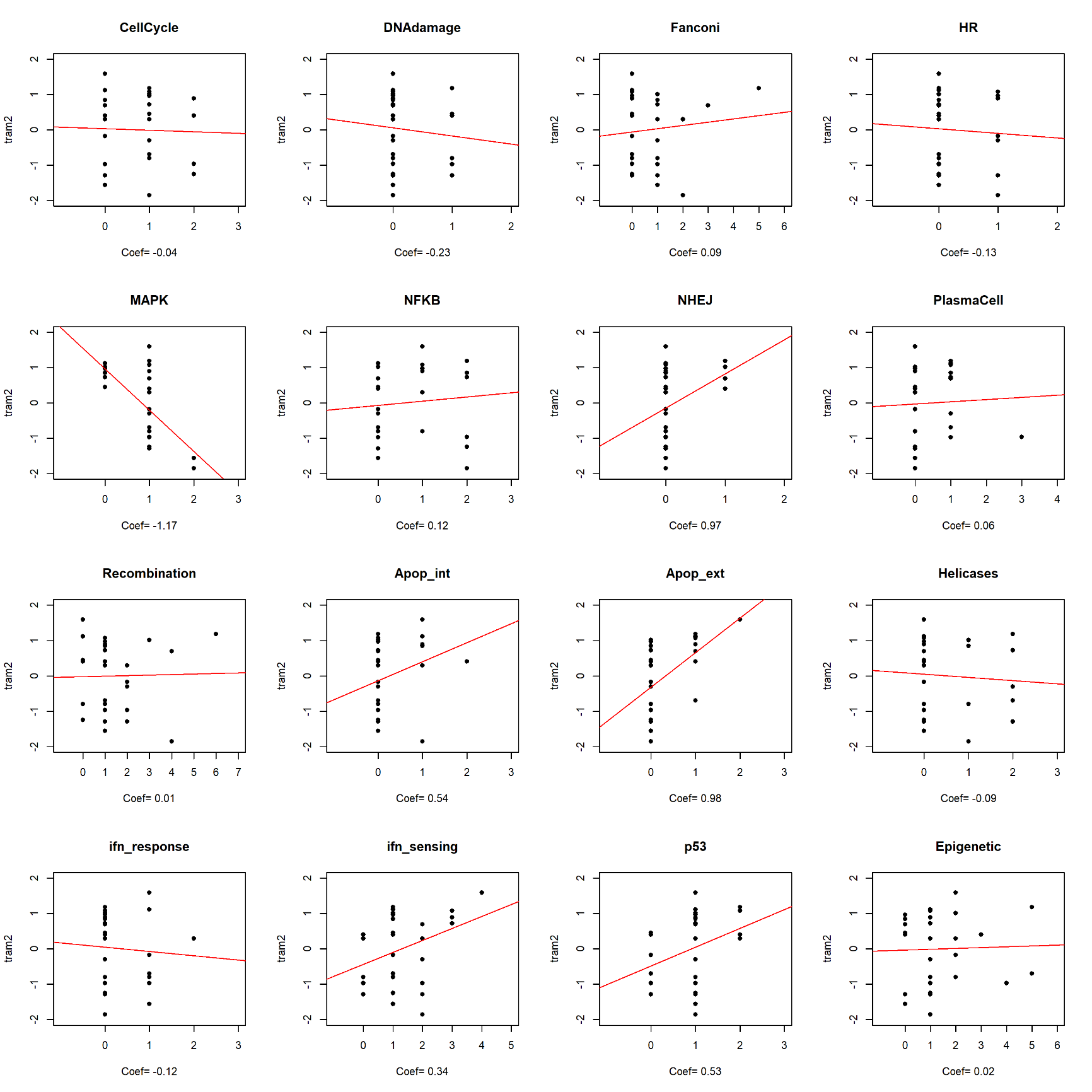


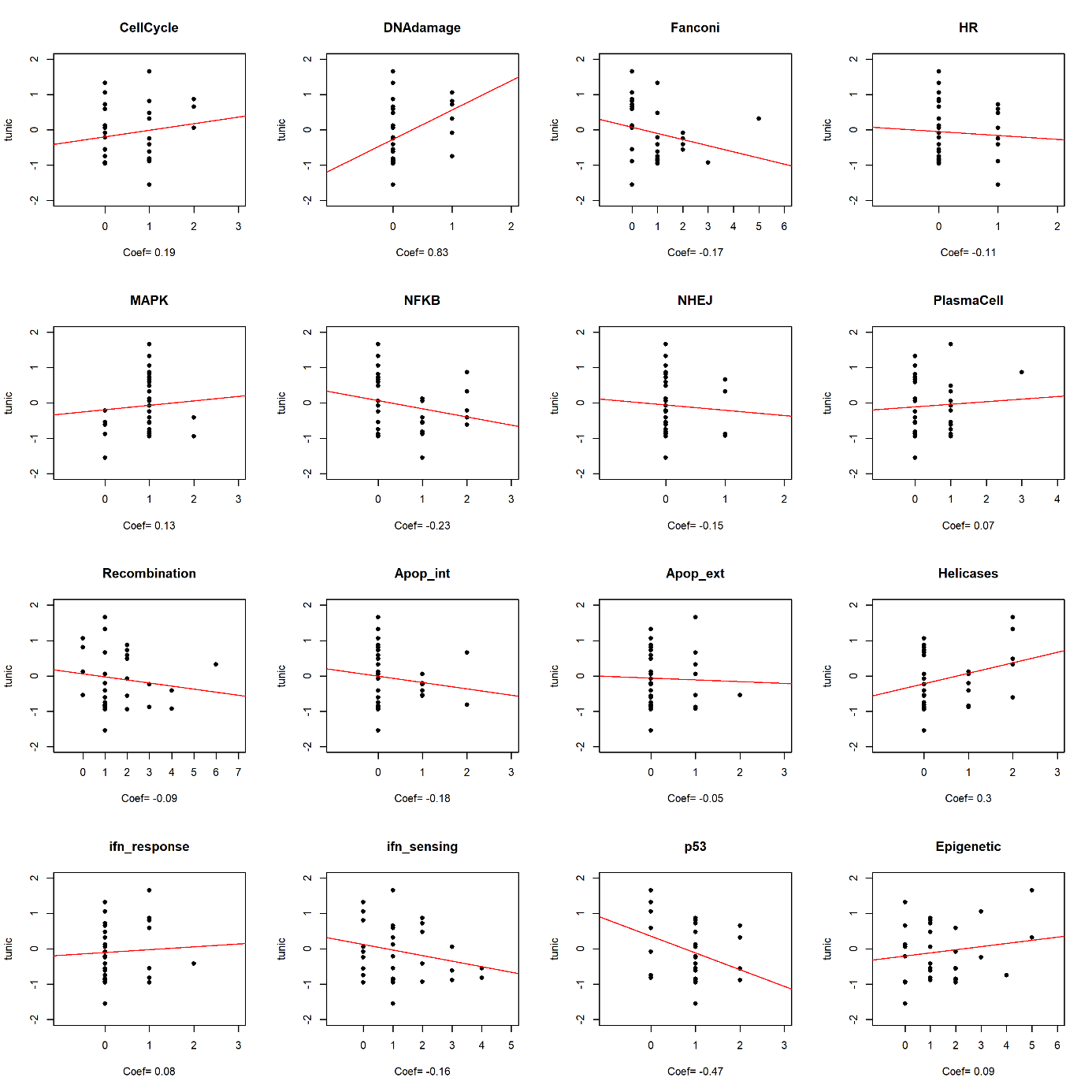


**Sup. Figure 9: Oncoprints by pathway of mostly mutated genes**
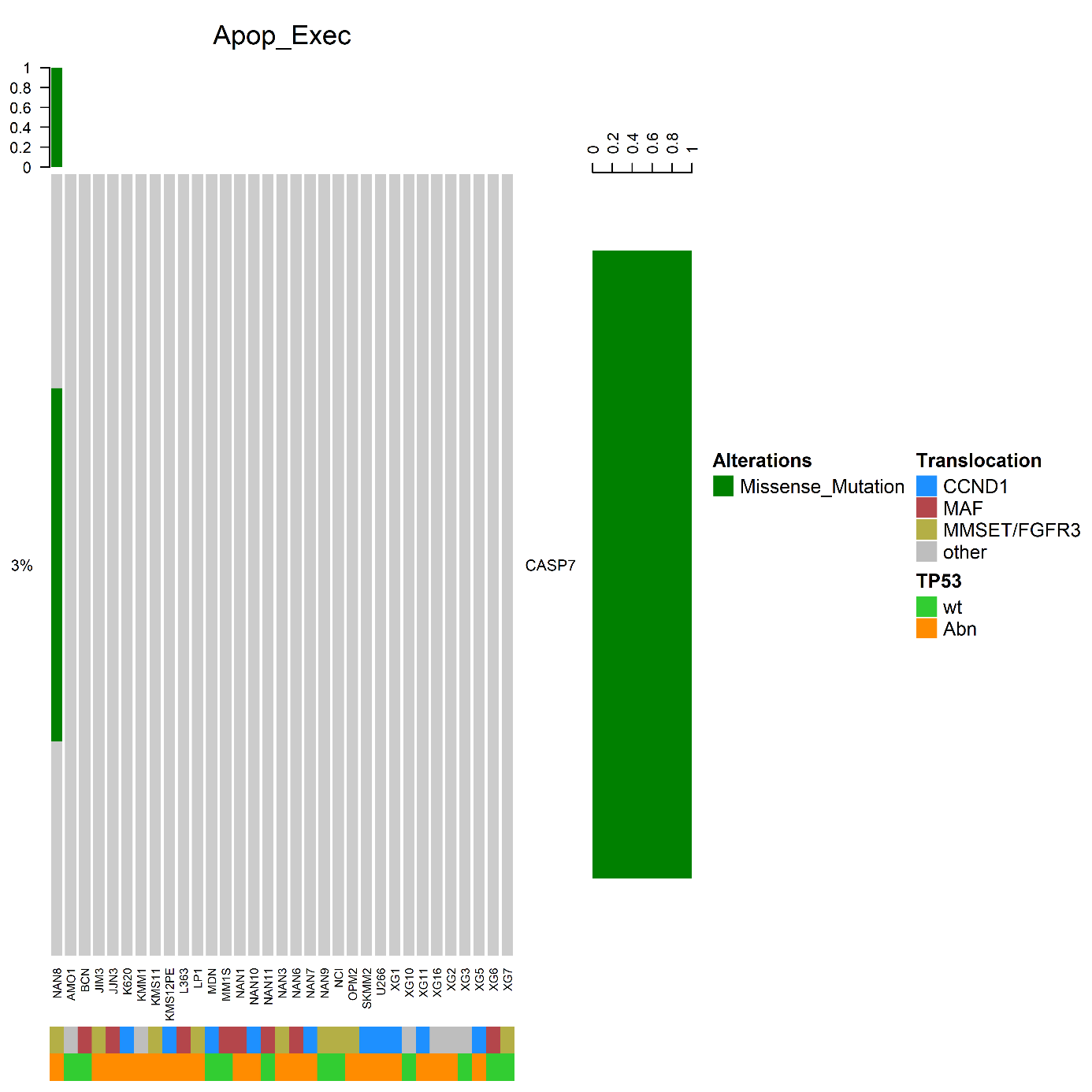

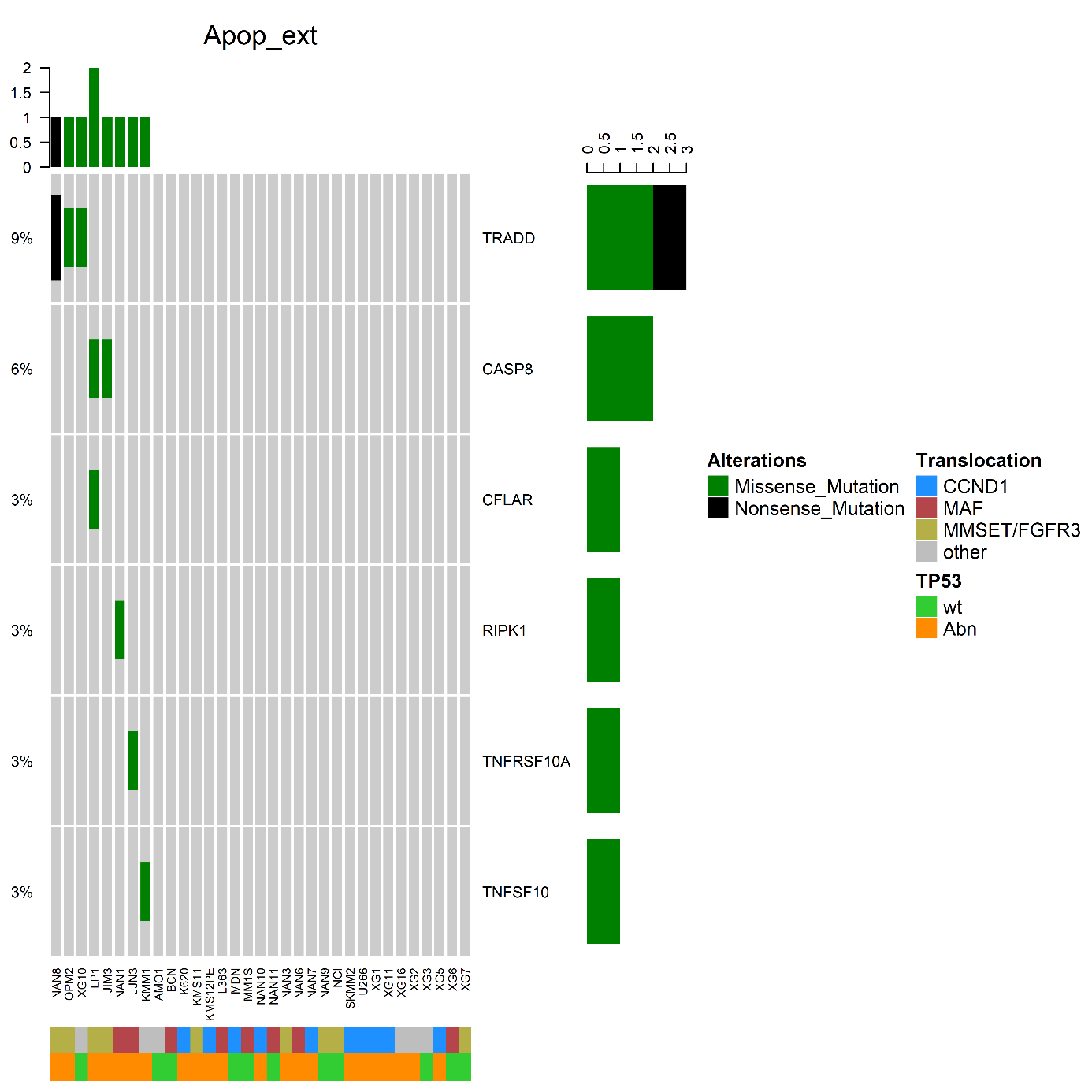

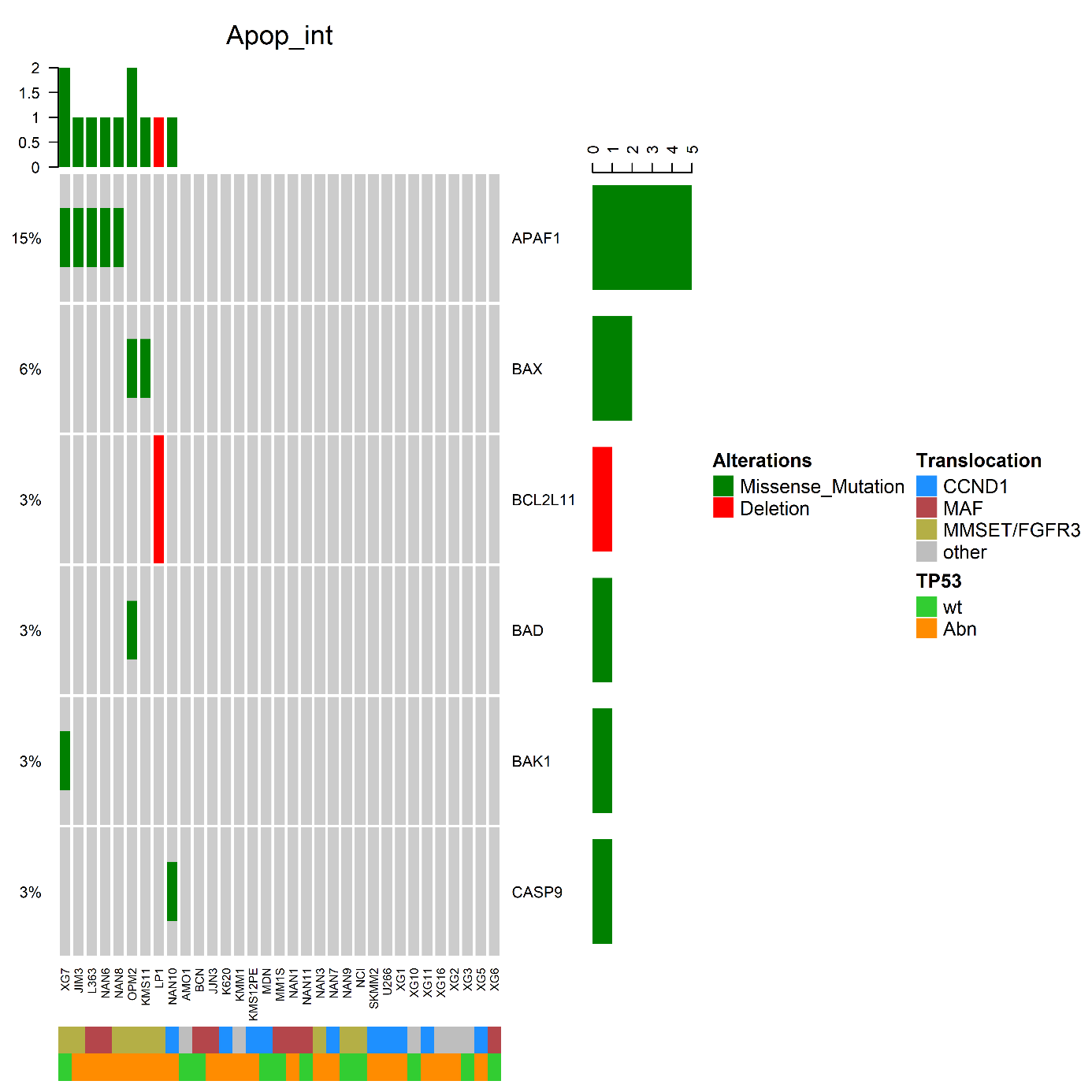

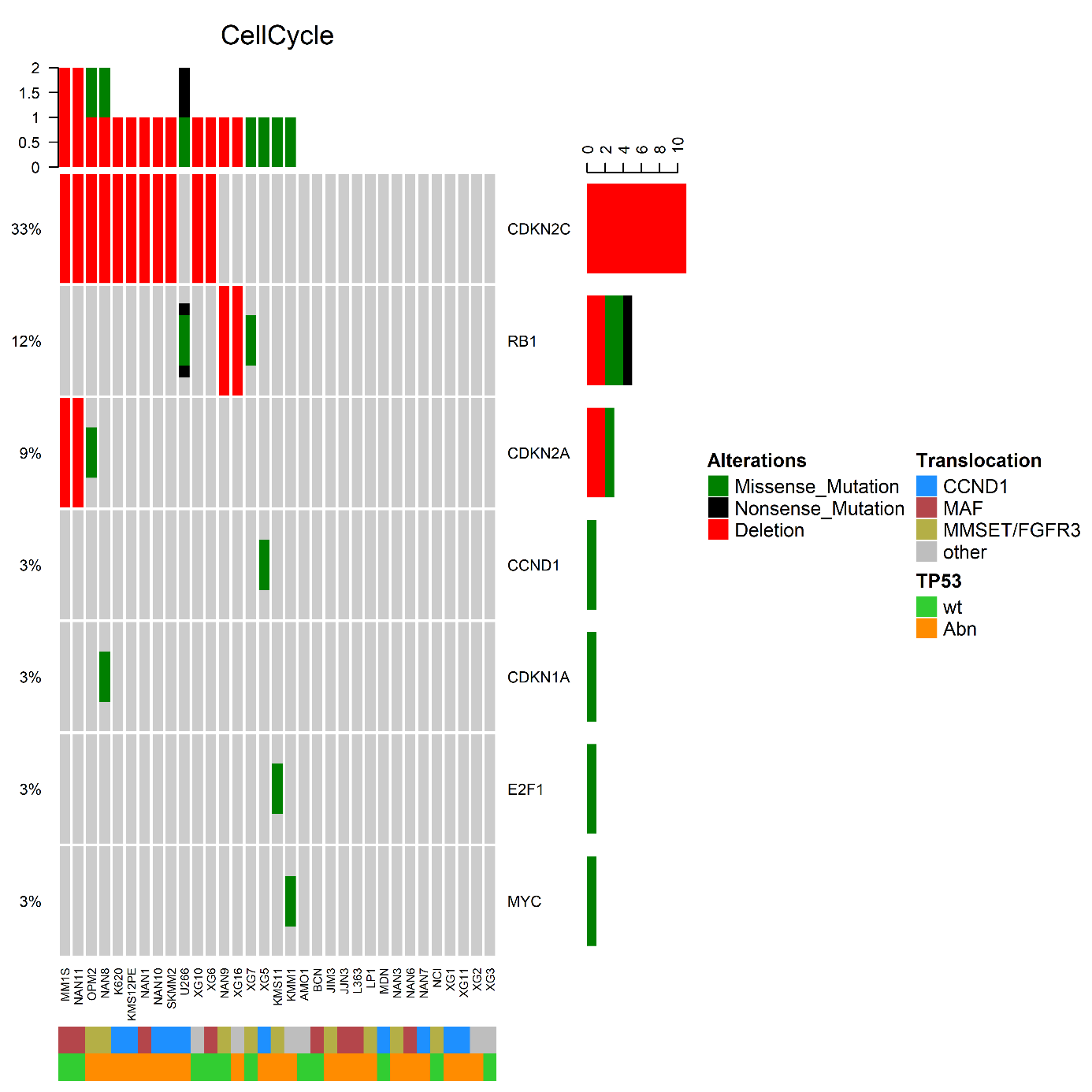

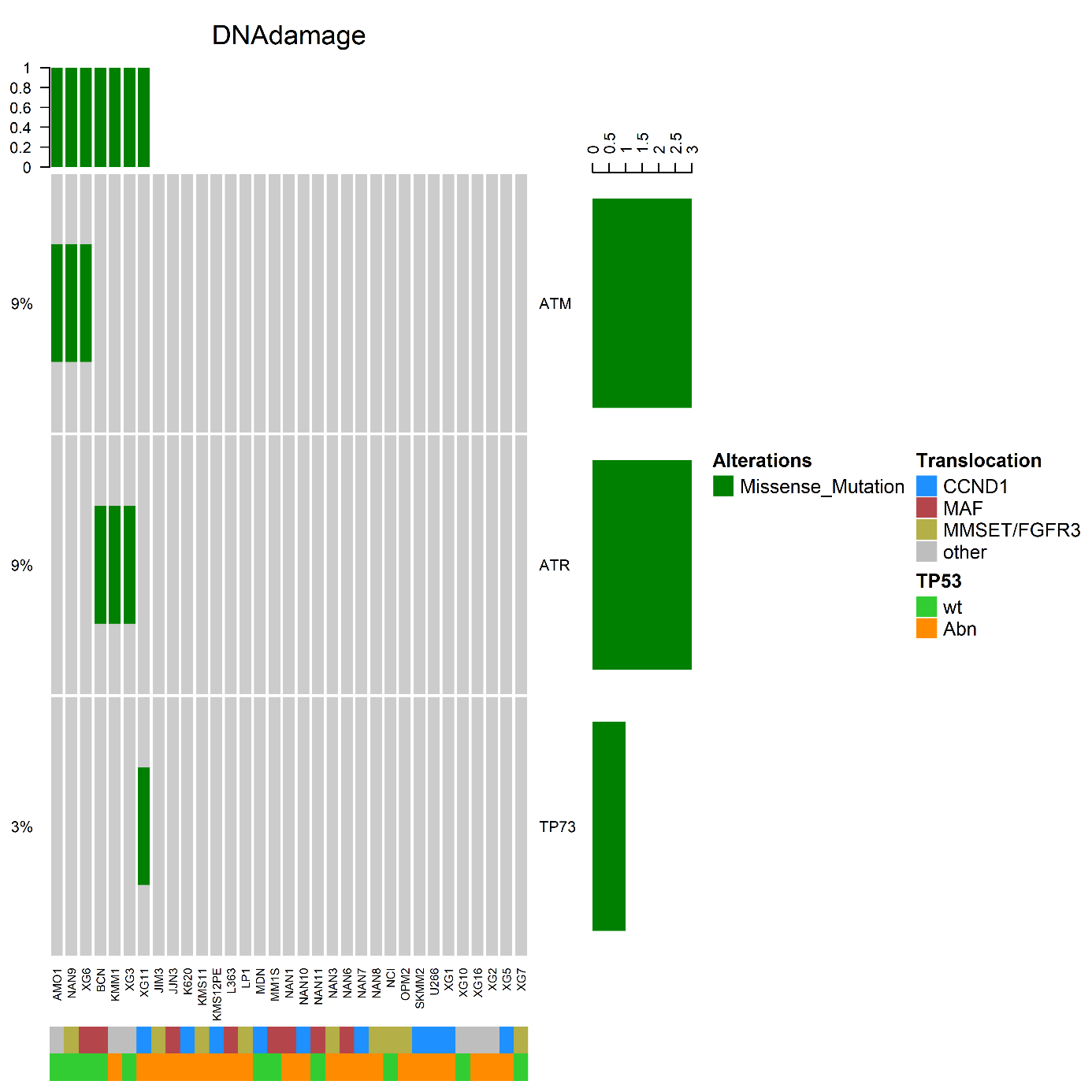

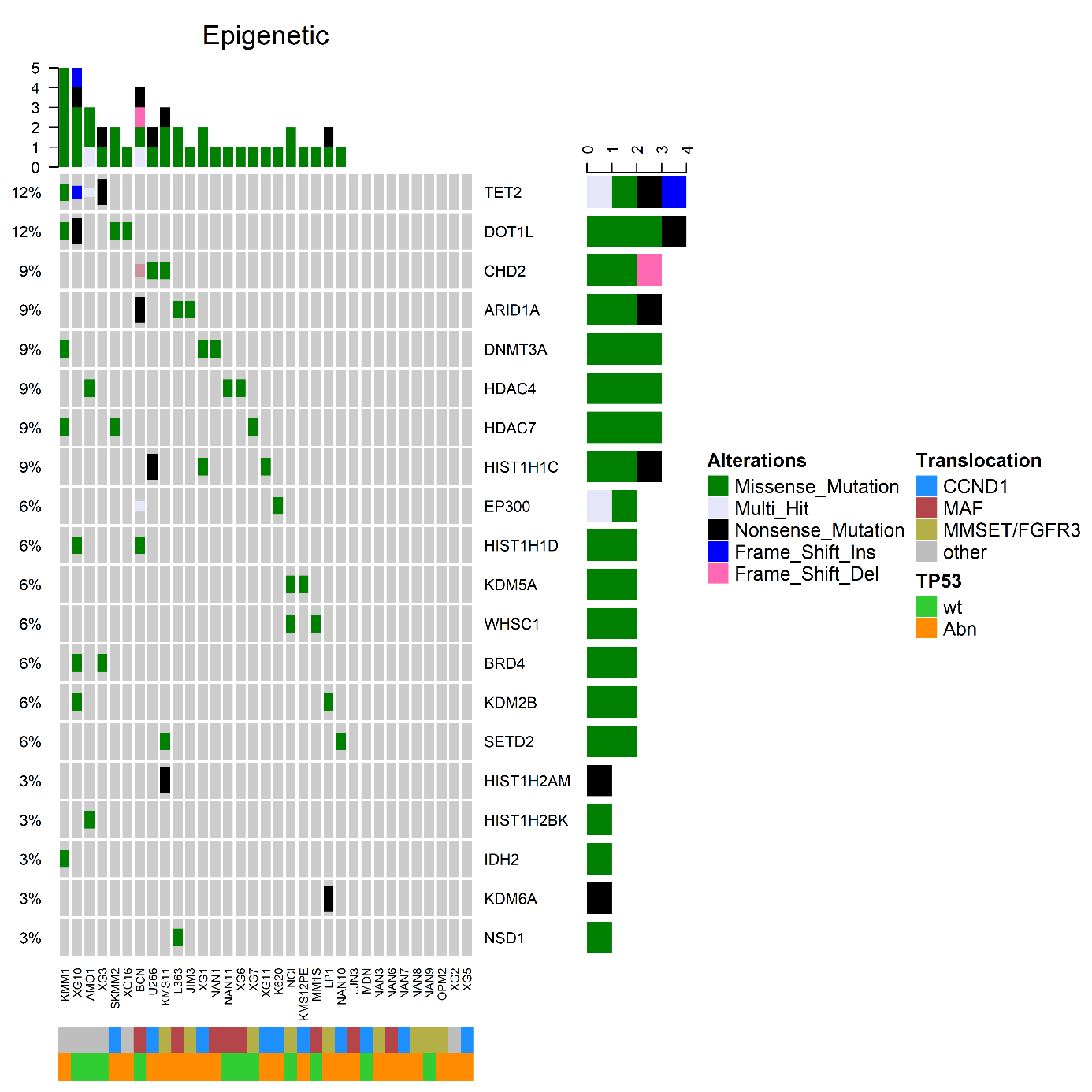

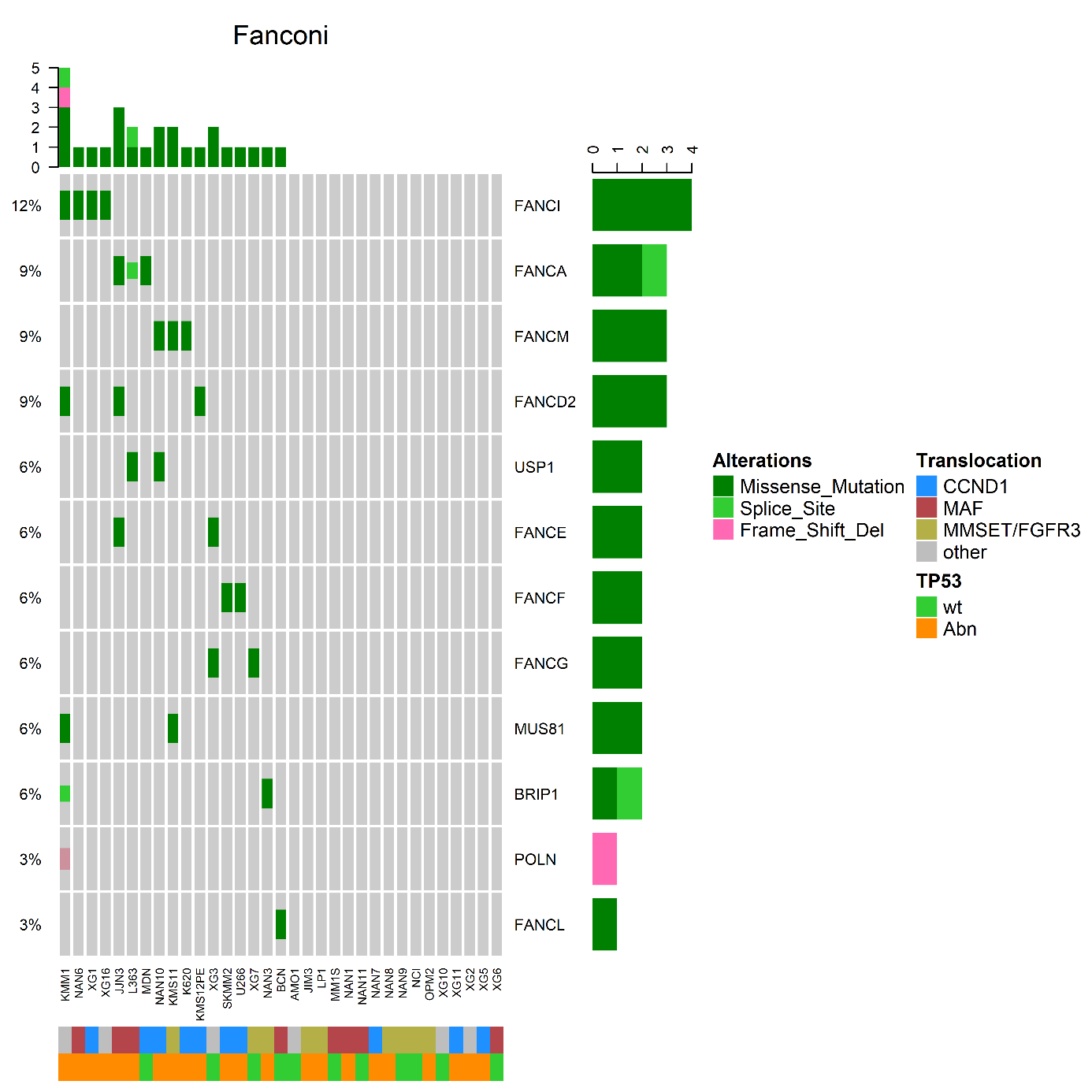

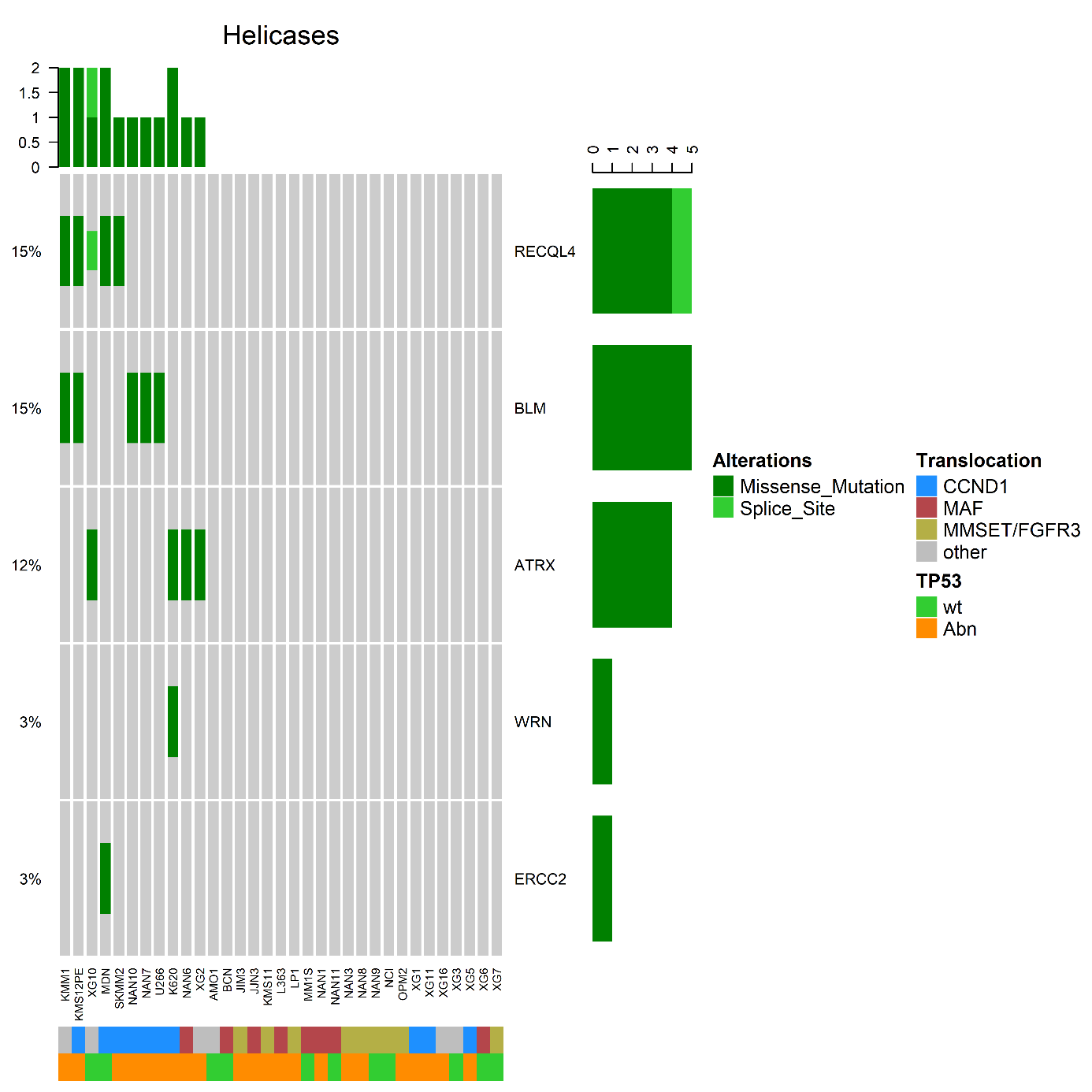

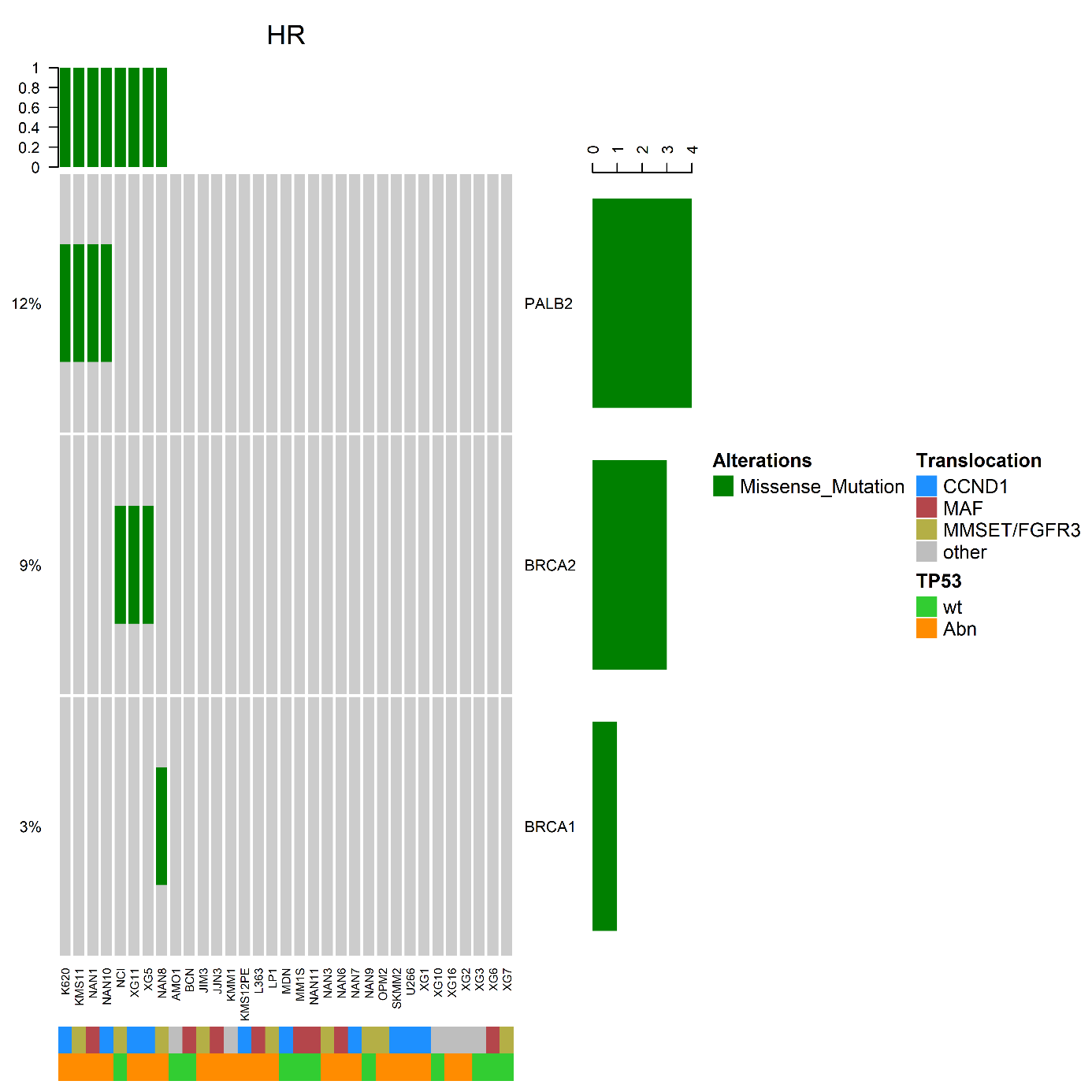

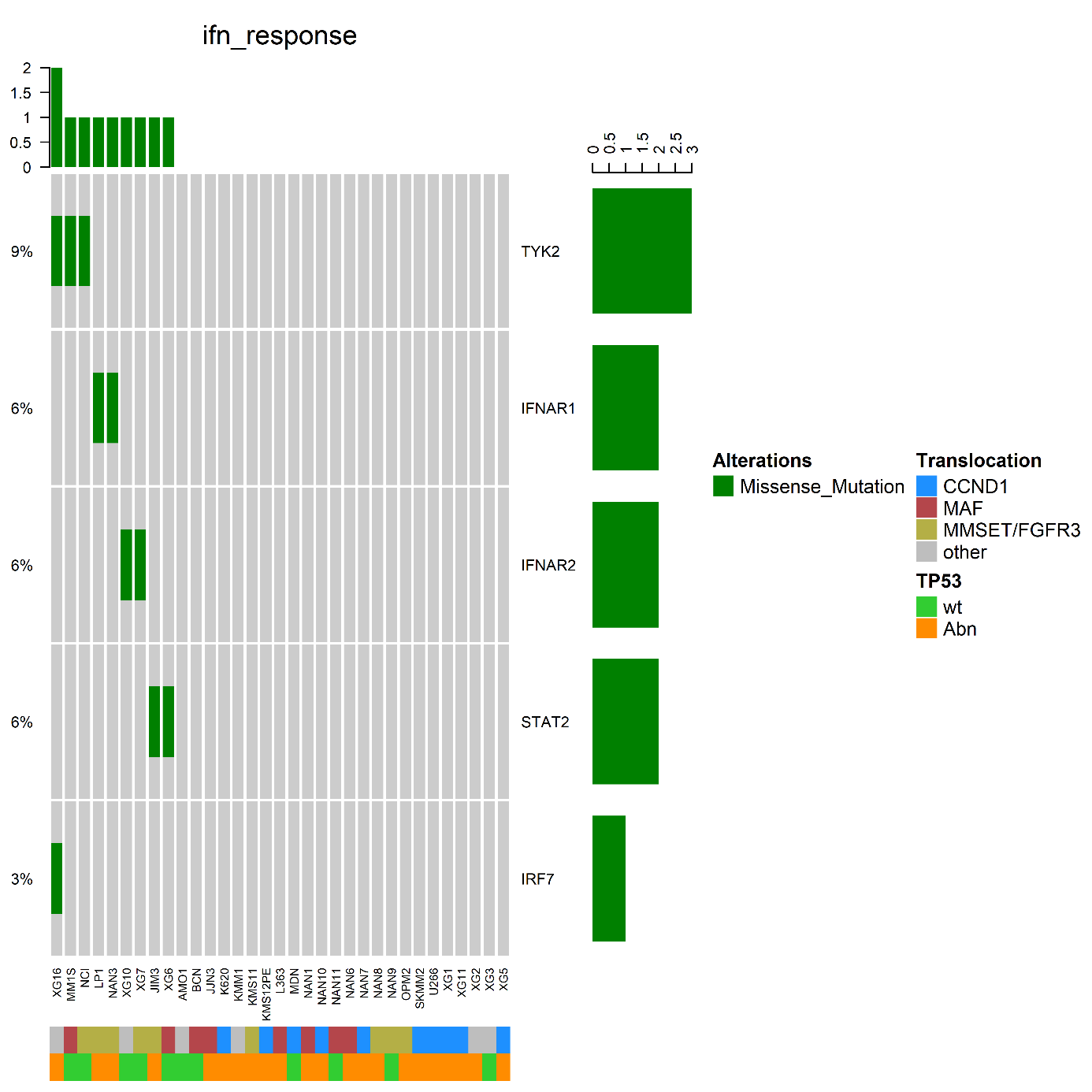

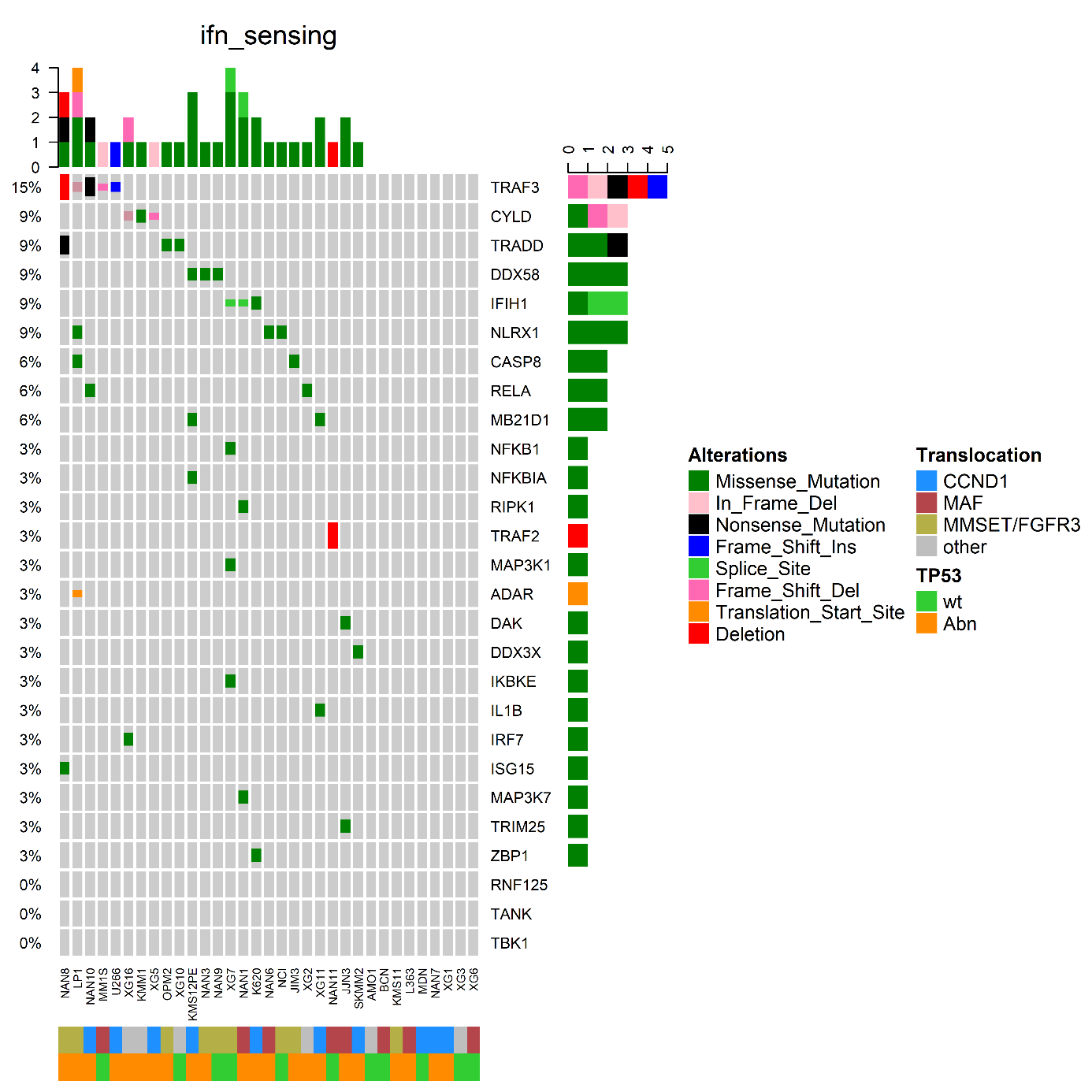

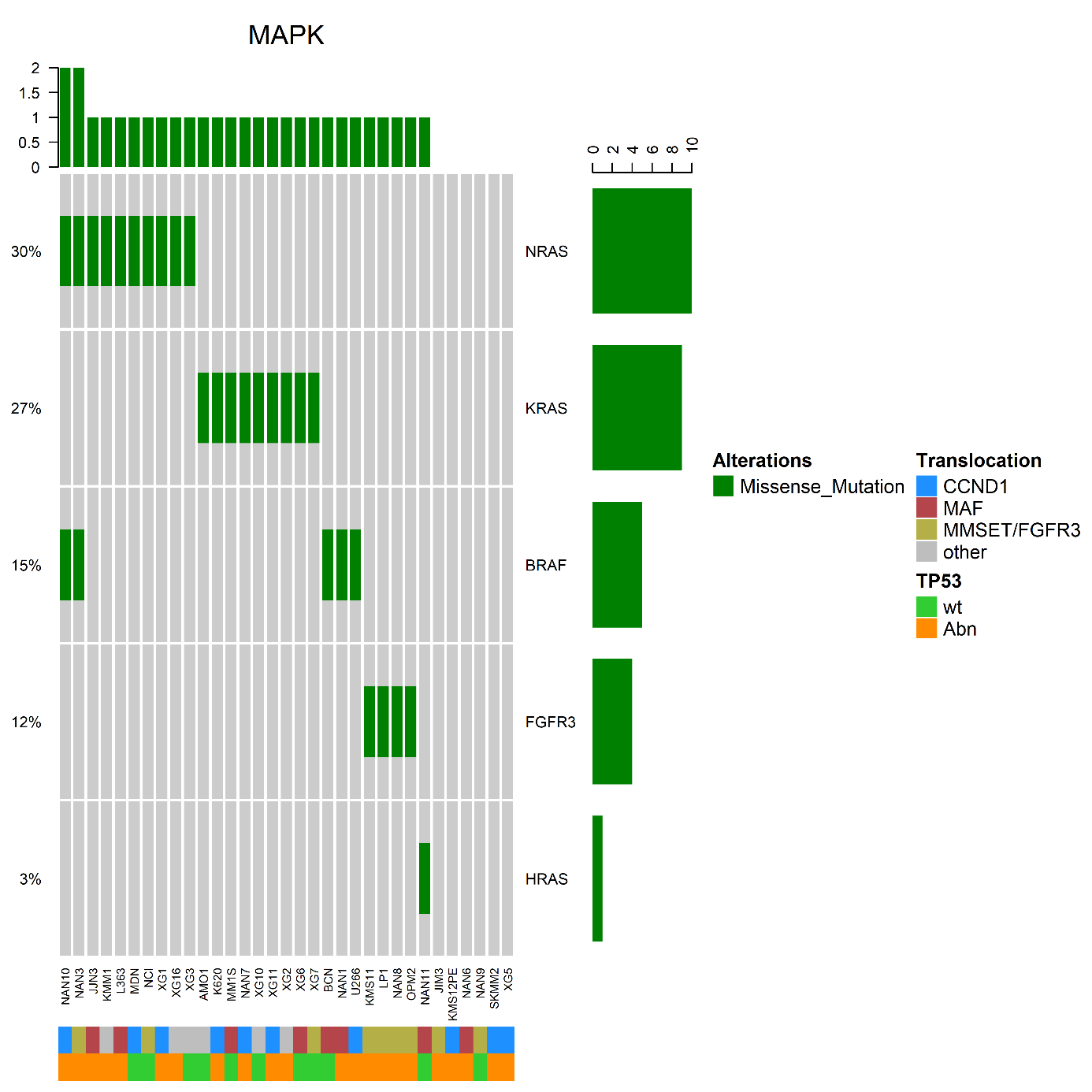

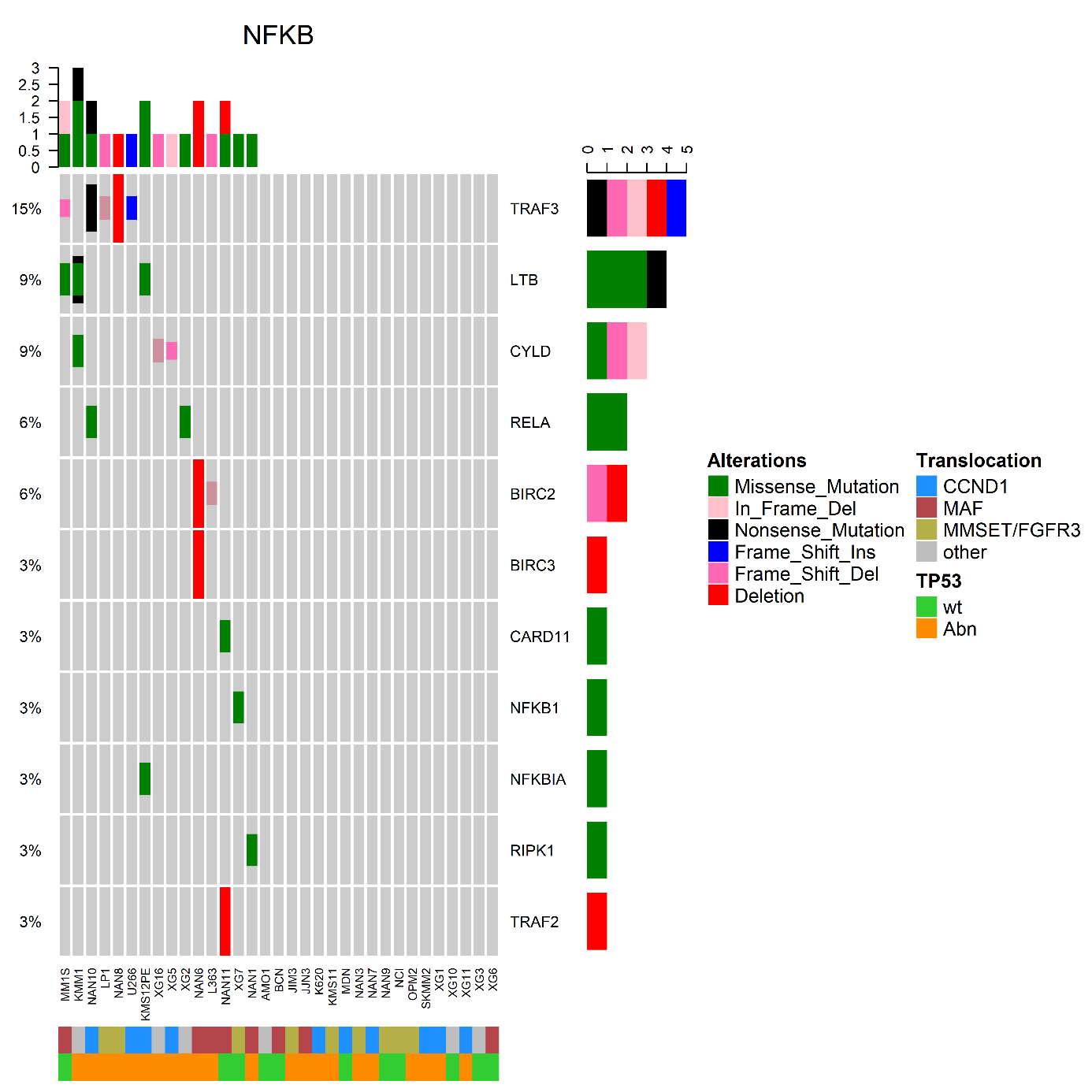

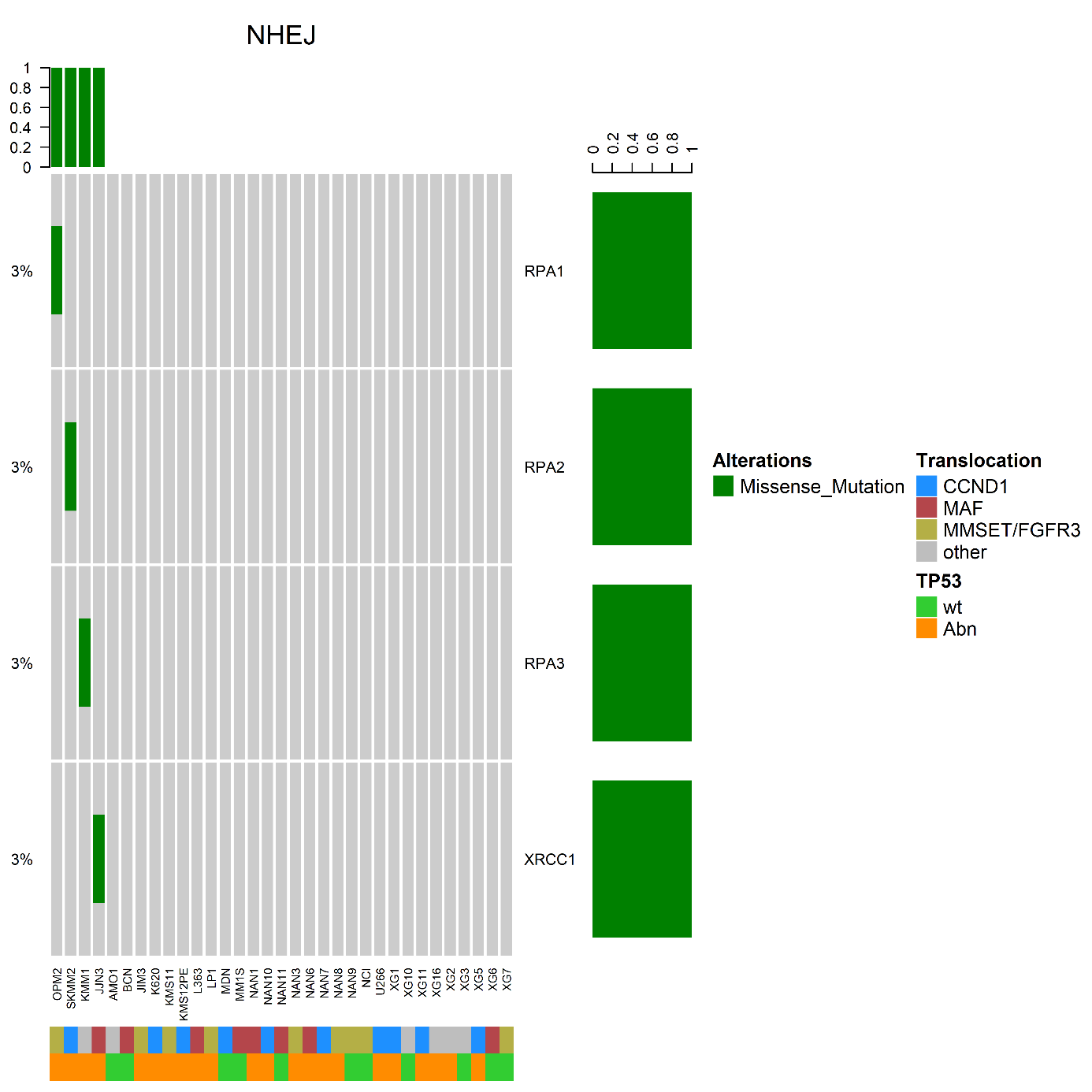

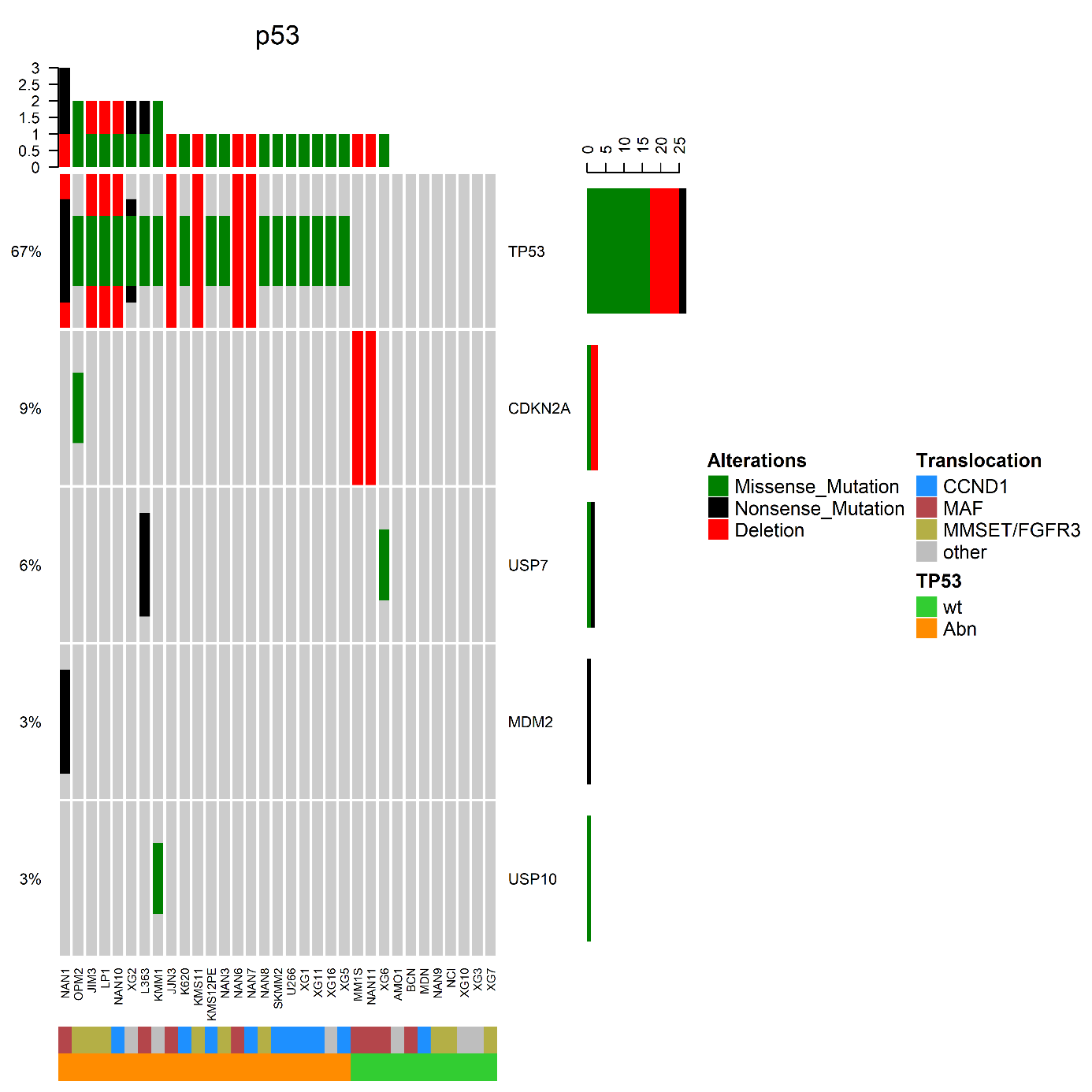

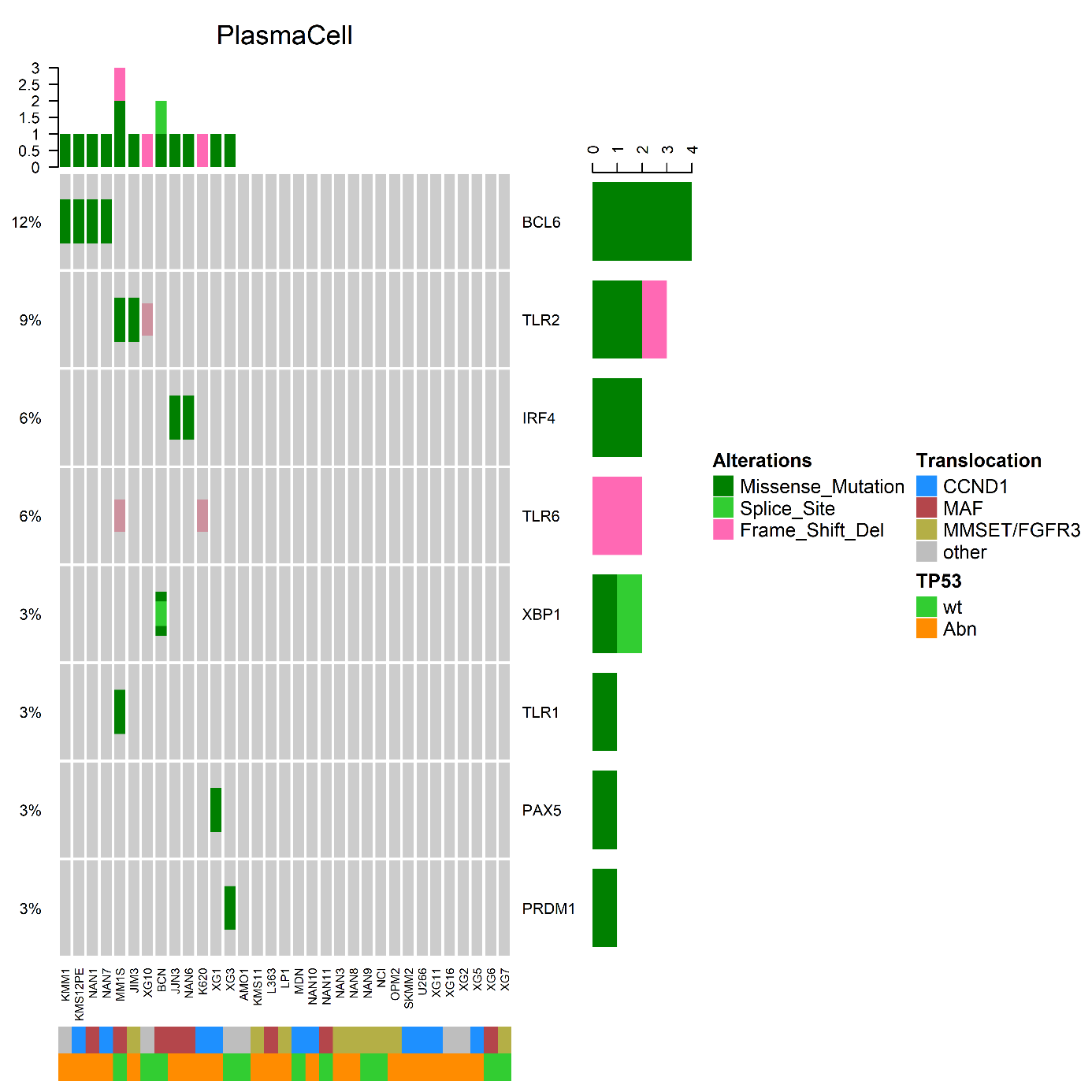

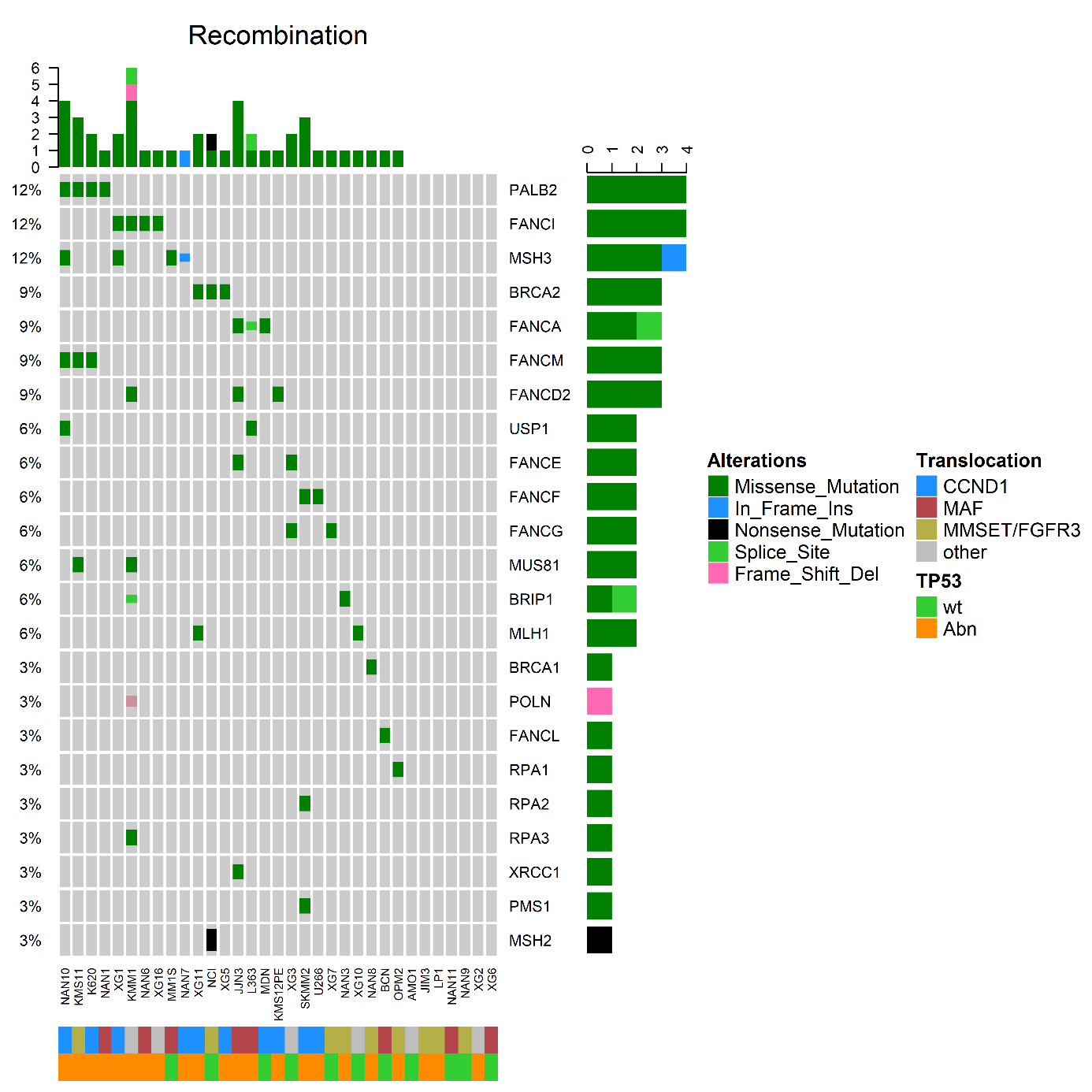

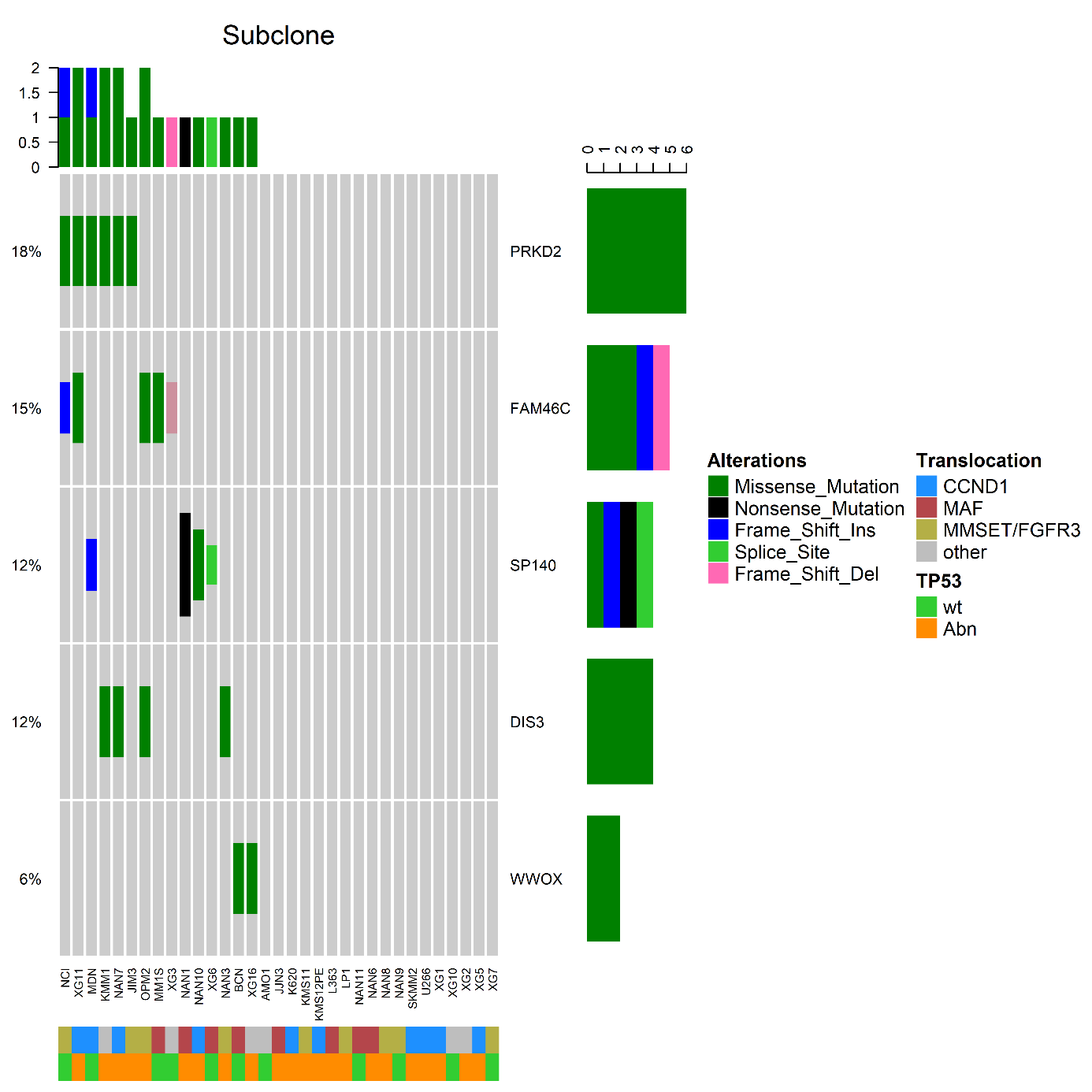


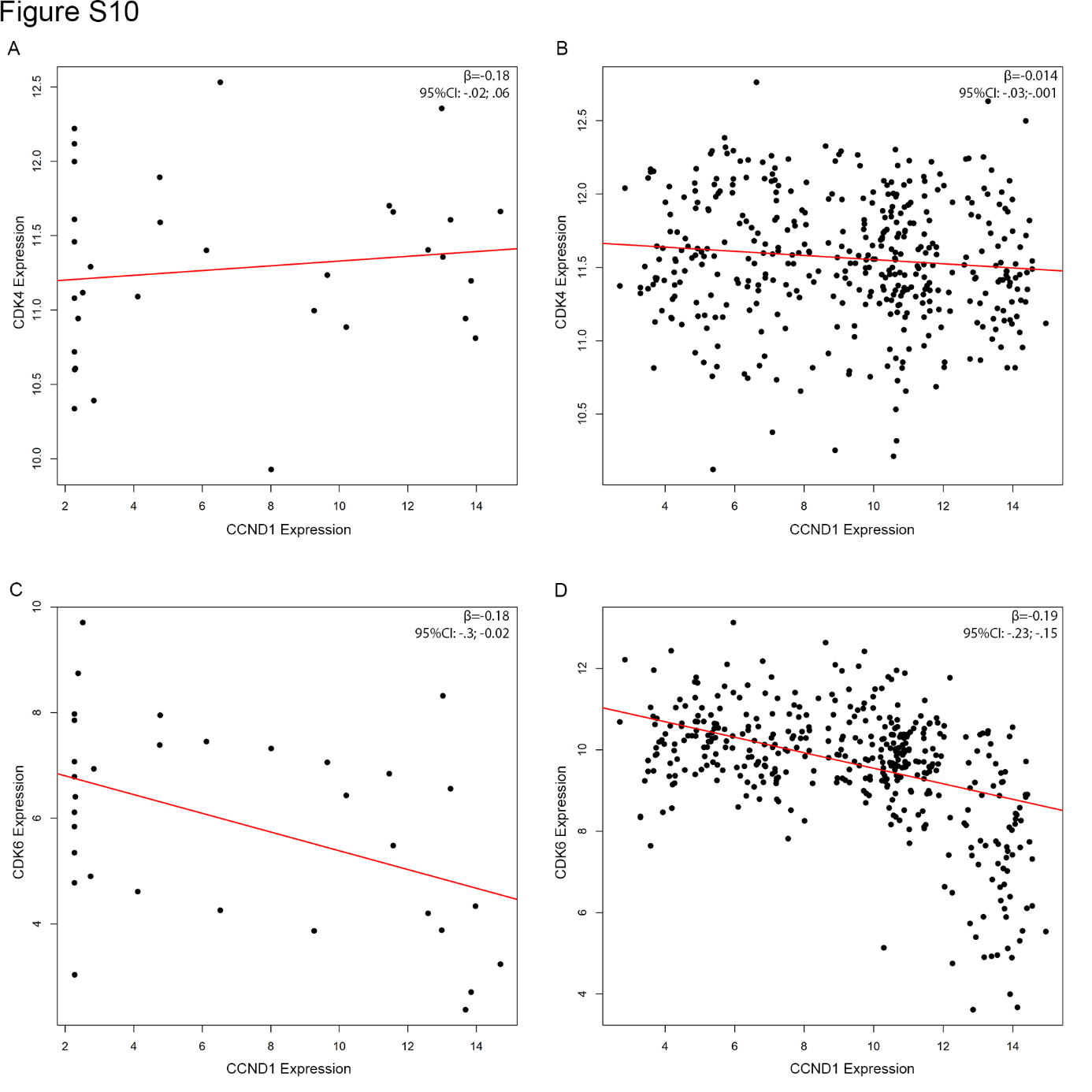


**Figure S10: CDK4 expression according to CCND1 expression in HMCLs (A) and Myeloma patients (B). CDK6 Expression according to CCND1 expression in HMCLs (C) and Myeloma patients (B).** Robust linear regression is displayed; regression line was drawn according to coefficients obtained after 5000 bootstrapped replicates. Only significant associations of tested drugs with the pathways of interest are displayed.

1 Moreaux J, Klein B, Bataille R, Descamps G, Maïga S, Hose D *et al.* A high-risk signature for patients with multiple myeloma established from the molecular classification of human myeloma cell lines. *Haematologica* 2011; **96**: 574–582.

2 Lim WK, Wang K, Lefebvre C, Califano A. Comparative analysis of microarray normalization procedures: effects on reverse engineering gene networks. *Bioinforma Oxf Engl* 2007; **23**: i282-288.

3 Ritchie ME, Phipson B, Wu D, Hu Y, Law CW, Shi W *et al.* limma powers differential expression analyses for RNA-sequencing and microarray studies. *Nucleic Acids Res* 2015; **43**: e47.

4 Smyth GK, Speed T. Normalization of cDNA microarray data. *Methods San Diego Calif* 2003; **31**: 265–273.

5 Li H, Durbin R. Fast and accurate long-read alignment with Burrows-Wheeler transform. *Bioinforma Oxf Engl* 2010; **26**: 589–595.

6 Love MI, Myšičková A, Sun R, Kalscheuer V, Vingron M, Haas SA. Modeling read counts for CNV detection in exome sequencing data. *Stat Appl Genet Mol Biol* 2011; **10**. doi:10.2202/1544-6115.1732.

7 Backenroth D, Homsy J, Murillo LR, Glessner J, Lin E, Brueckner M *et al.* CANOES: detecting rare copy number variants from whole exome sequencing data. *Nucleic Acids Res* 2014; **42**: e97.

8 Robinson JT, Thorvaldsdóttir H, Winckler W, Guttman M, Lander ES, Getz G *et al.* Integrative genomics viewer. Nat. Biotechnol. 2011. doi:10.1038/nbt.1754.

9 Surget S, Lemieux-Blanchard E, Maïga S, Descamps G, Le Gouill S, Moreau P *et al.* Bendamustine and melphalan kill myeloma cells similarly through reactive oxygen species production and activation of the p53 pathway and do not overcome resistance to each other. *Leuk Lymphoma* 2014; **55**: 2165–2173.

10 Mayakonda A, Koeffler HP. Maftools: Efficient analysis, visualization and summarization of MAF files from large-scale cohort based cancer studies. *bioRxiv* 2016; : 052662.

11 Zhan F, Huang Y, Colla S, Stewart JP, Hanamura I, Gupta S *et al.* The molecular classification of multiple myeloma. *Blood* 2006; **108**: 2020–2028.

12 Alexandrov LB, Nik-Zainal S, Wedge DC, Aparicio SAJR, Behjati S, Biankin AV *et al.* Signatures of mutational processes in human cancer. *Nature* 2013; **500**: 415–421.

13 Gerstung M, Pellagatti A, Malcovati L, Giagounidis A, Porta MGD, Jädersten M *et al.* Combining gene mutation with gene expression data improves outcome prediction in myelodysplastic syndromes. *Nat Commun* 2015; **6**: 5901.
