# Supplementary material for: Whole-exon sequencing of human myeloma cell lines shows mutations related to myeloma patients at relapse with major hits in the DNA regulation and repair pathways
Source: J Hematol Oncol. 2018 Dec 13;11:137. doi: 10.1186/s13045-018-0679-0 (PMC6293660; doi:10.1186/s13045-018-0679-0)
Supplement: Supplementary file 3 — Lollipop plots of the main sequence variants. (PDF 2025 kb) [file 13045_2018_679_MOESM3_ESM.pdf]

IFNAR2: [Somatic Mutation Rate: 6.06%]

NM_207585

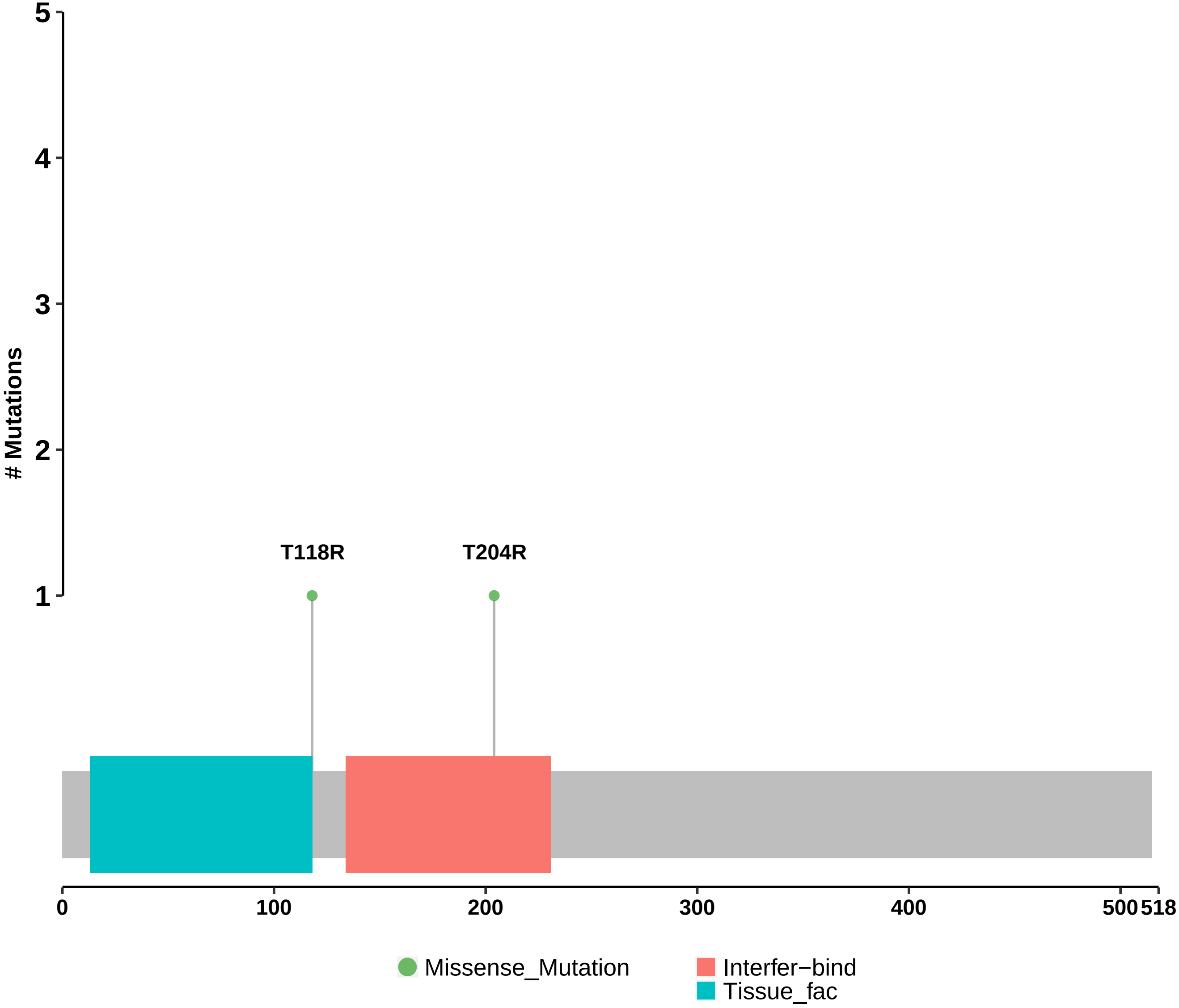

KDM2B: [Somatic Mutation Rate: 6.06%]

NM_032590

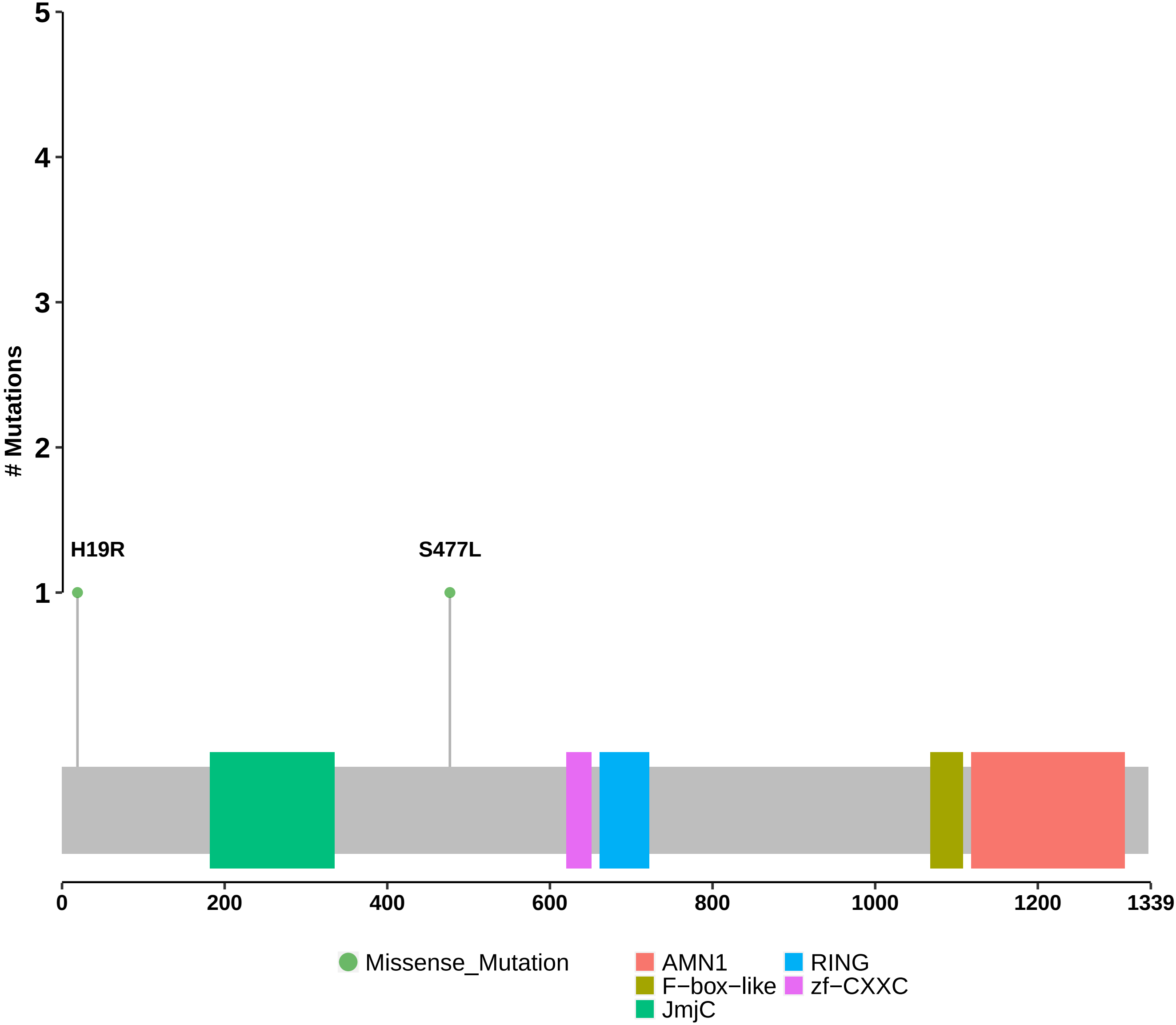

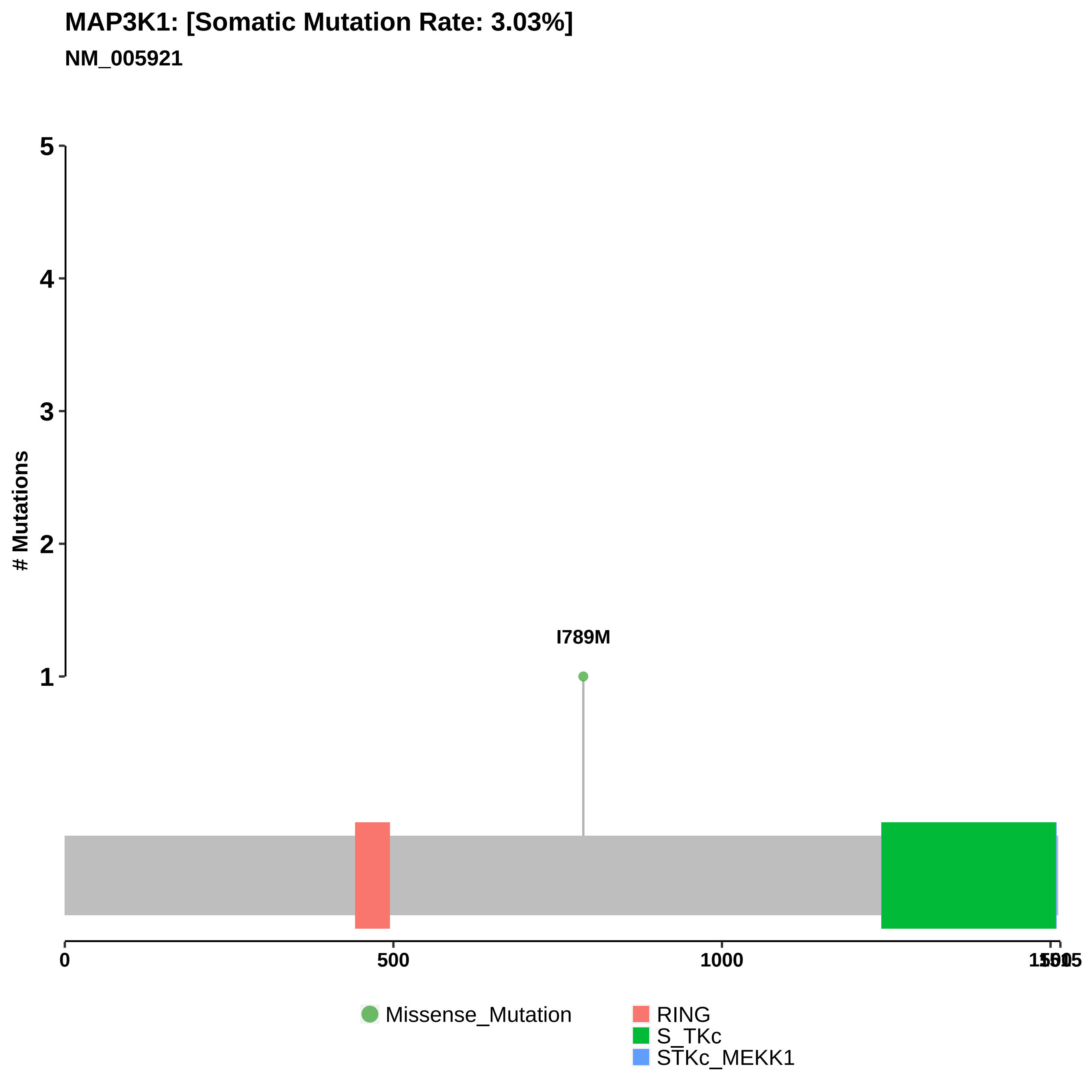
MAP3K1: [Somatic Mutation Rate: 3.03%]
NM_005921
Mutations
5
4
3
2
1
I789M
0
500
1000
1500
1515
Missense_Mutation
RING
S_TKc
STKc_MEKK1

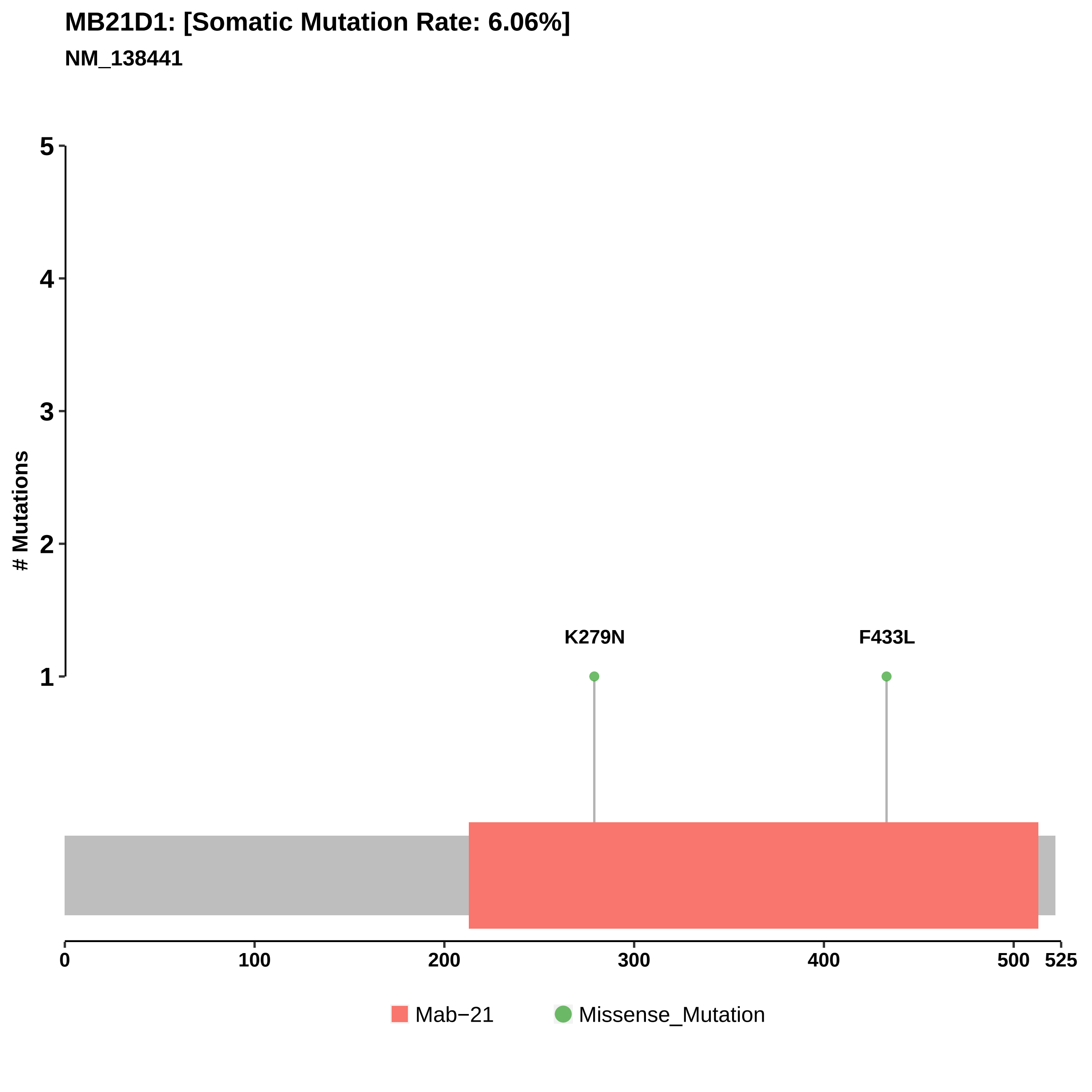
MB21D1: [Somatic Mutation Rate: 6.06%]
NM_138441
Mutations
5
4
3
2
1
K279N
F433L
0
100
200
300
400
500
525
Mab−21
Missense_Mutation

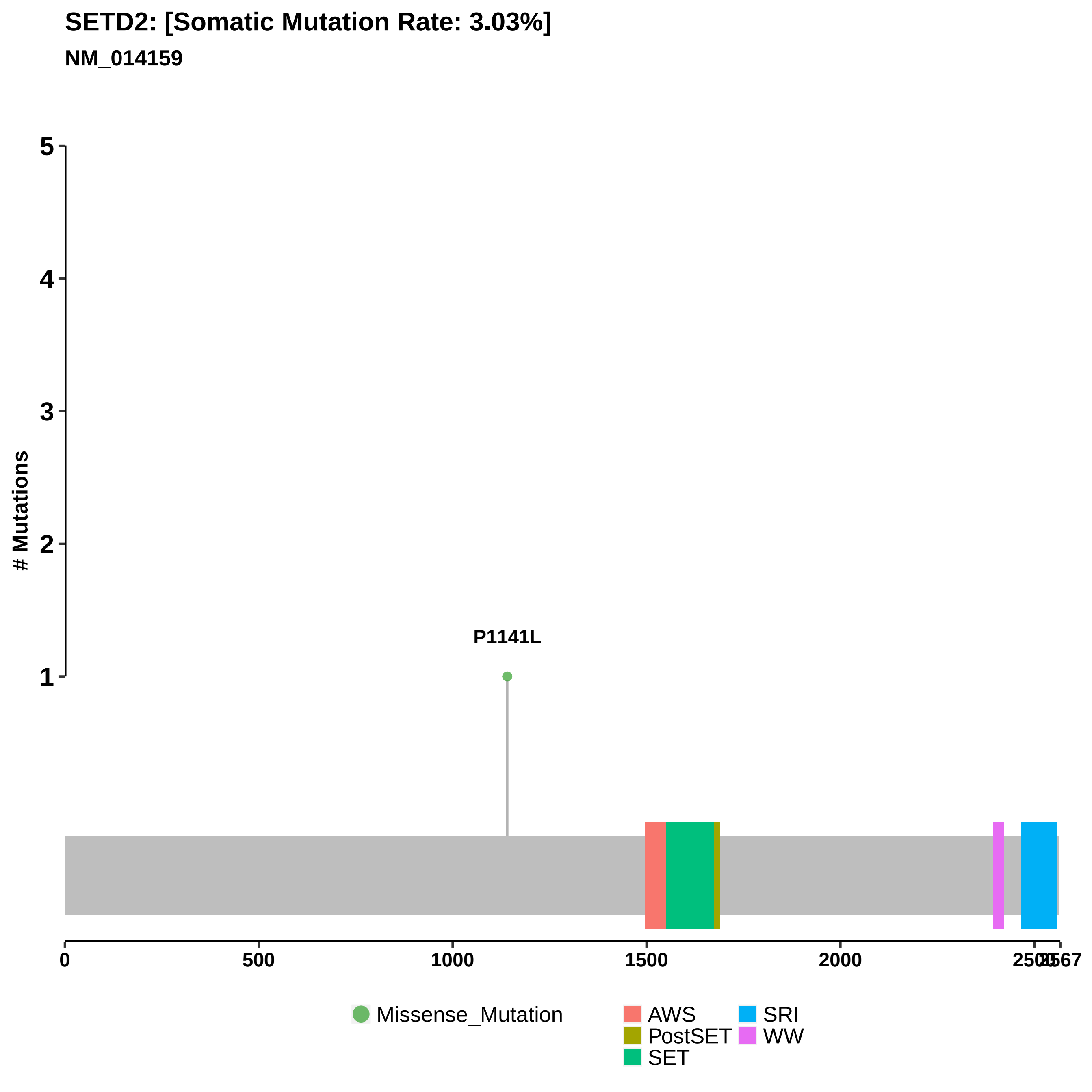
SETD2: [Somatic Mutation Rate: 3.03%]
NM_014159
Mutations
5
4
3
2
1
P1141L
0
500
1000
1500
2000
2500
2567
Missense_Mutation
AWS
PostSET
SET
SRI
WW

STAT2: [Somatic Mutation Rate: 6.06%]
NM_005419

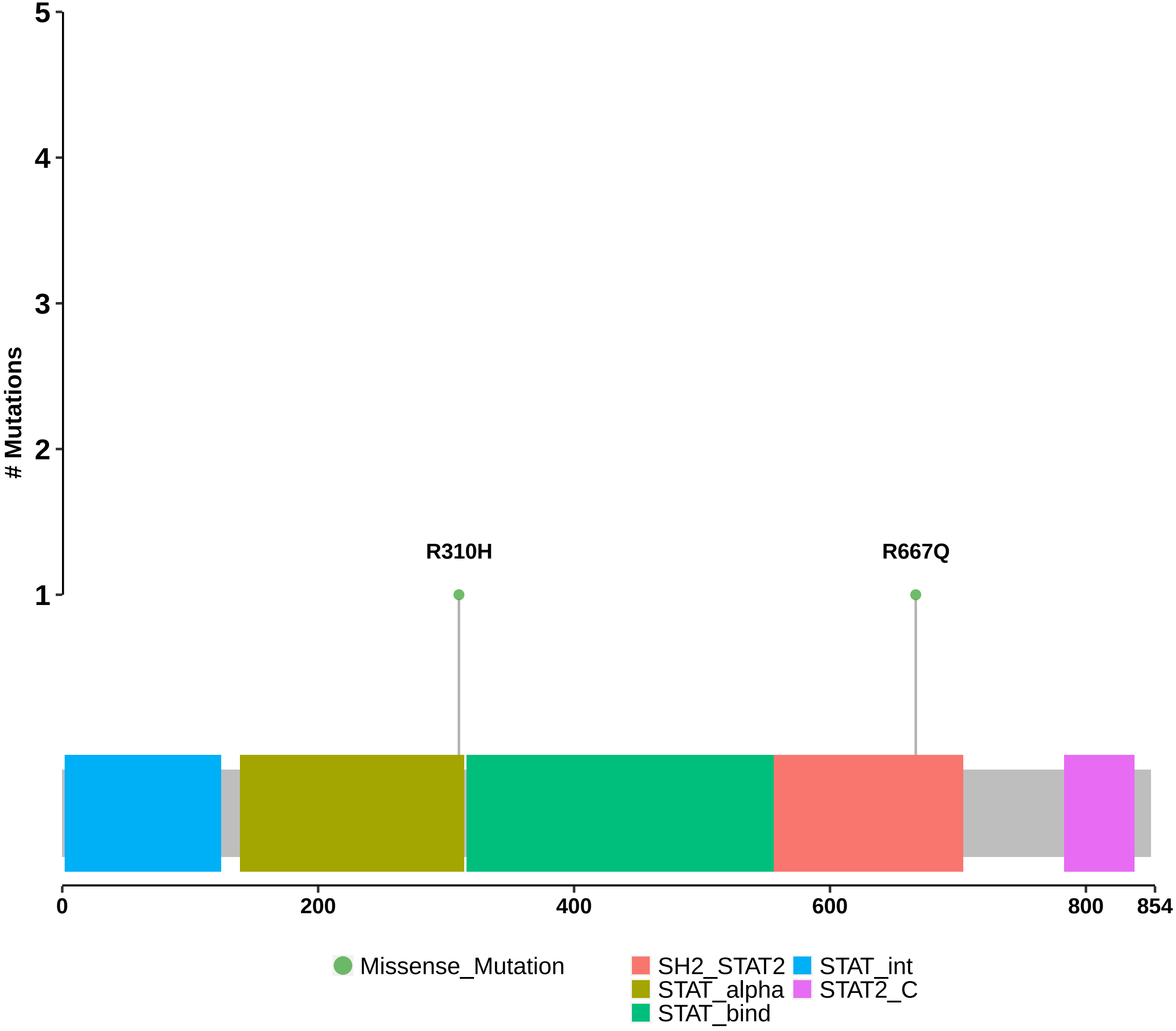

DAK: [Somatic Mutation Rate: 3.03%]

NM_015533

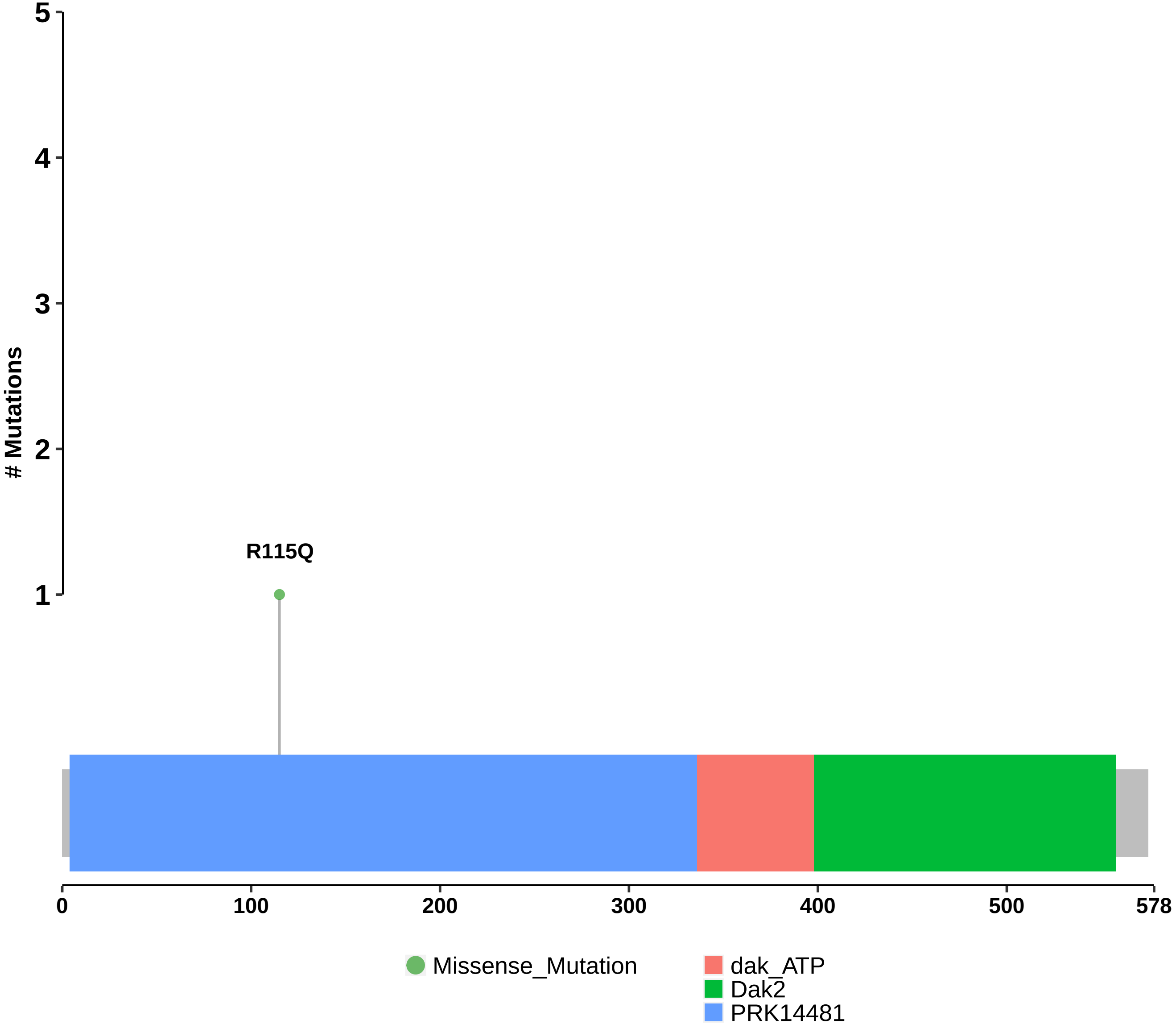

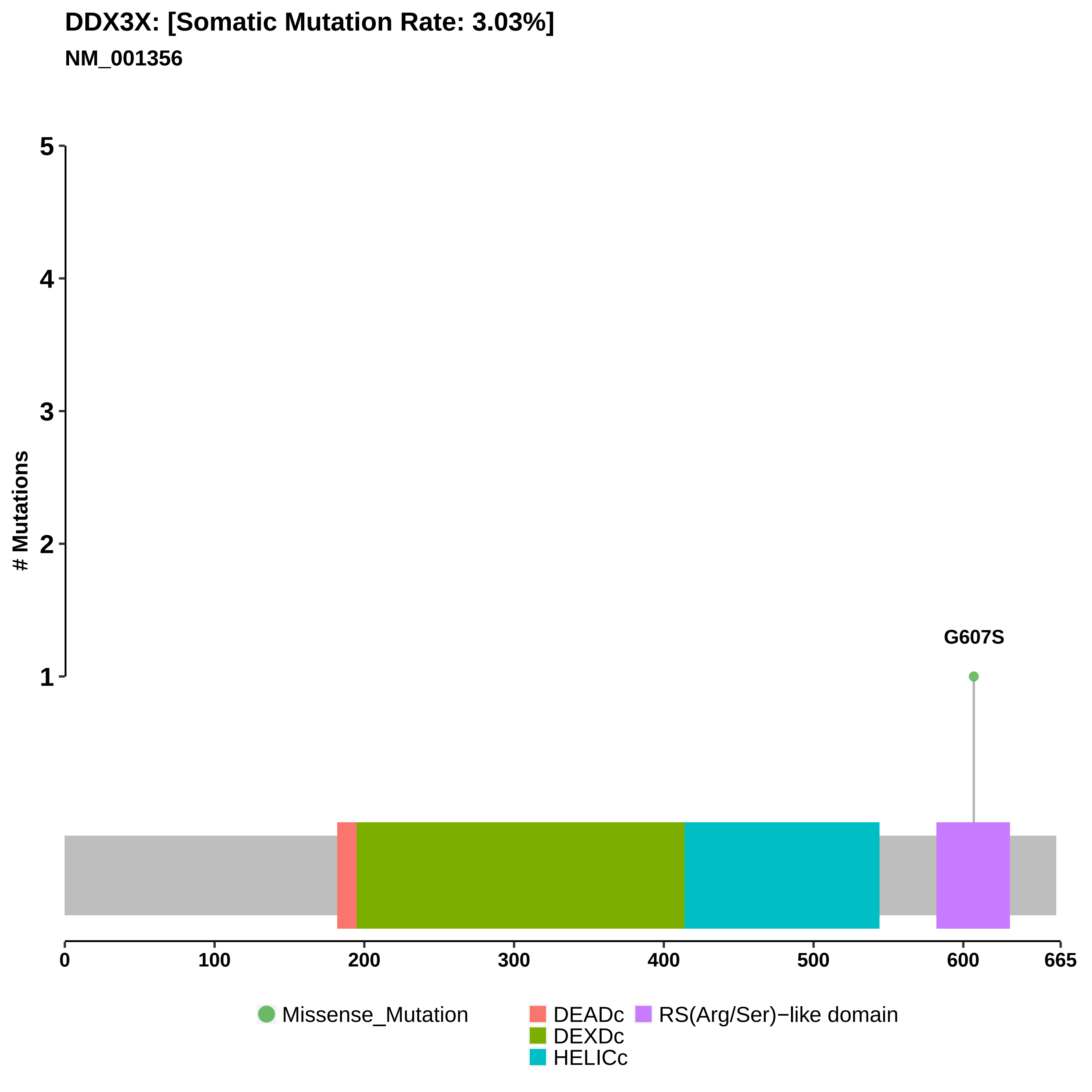
DDX3X: [Somatic Mutation Rate: 3.03%]
NM_001356
Mutations
5
4
3
2
1
G607S
0
100
200
300
400
500
600
665
Missense_Mutation
DEADc
DEXDc
HELICc
RS(Arg/Ser)−like domain

HIST1H2AM: [Somatic Mutation Rate: 3.03%]

NM_003514

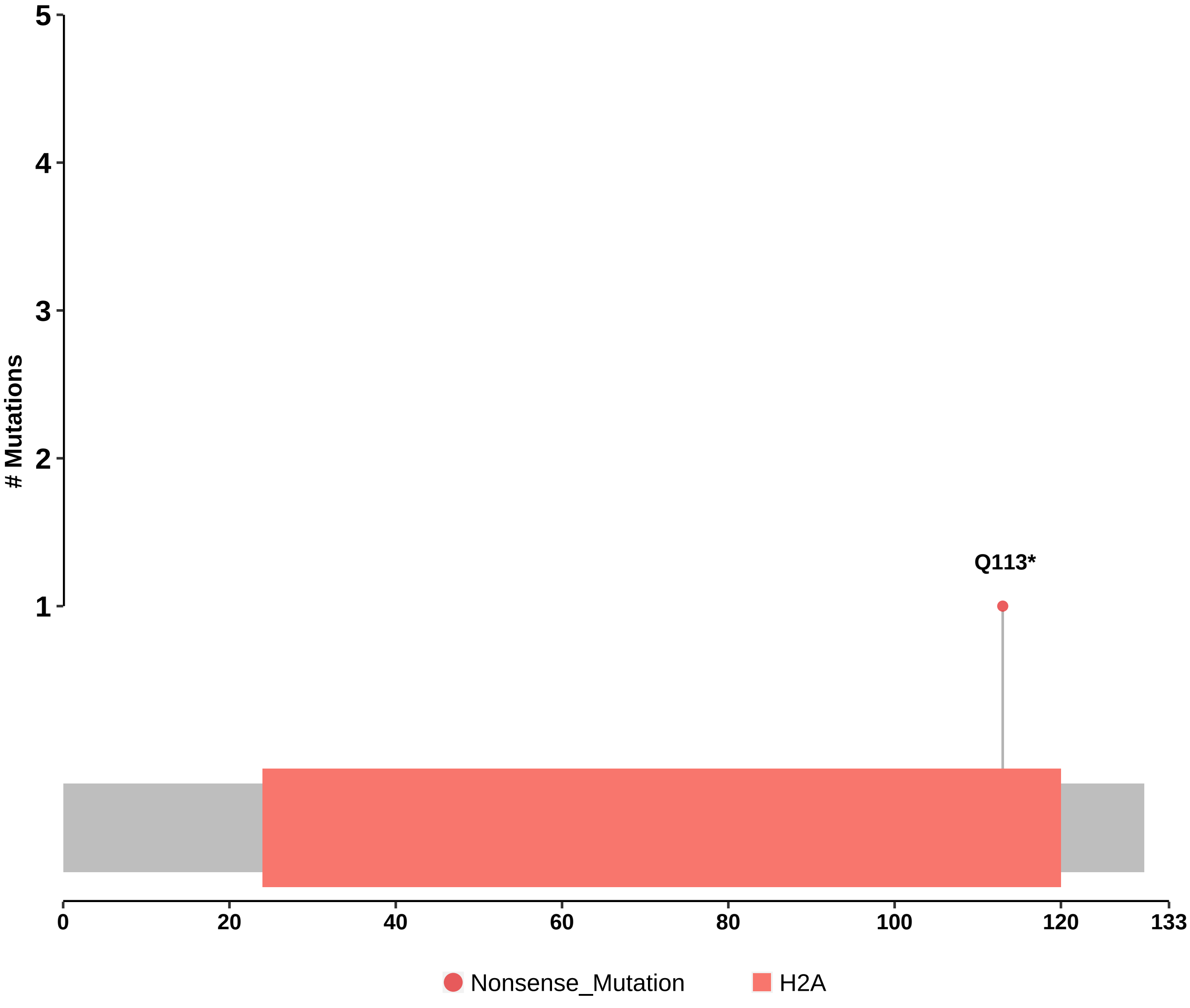

HIST1H2BK: [Somatic Mutation Rate: 3.03%]

NM_080593

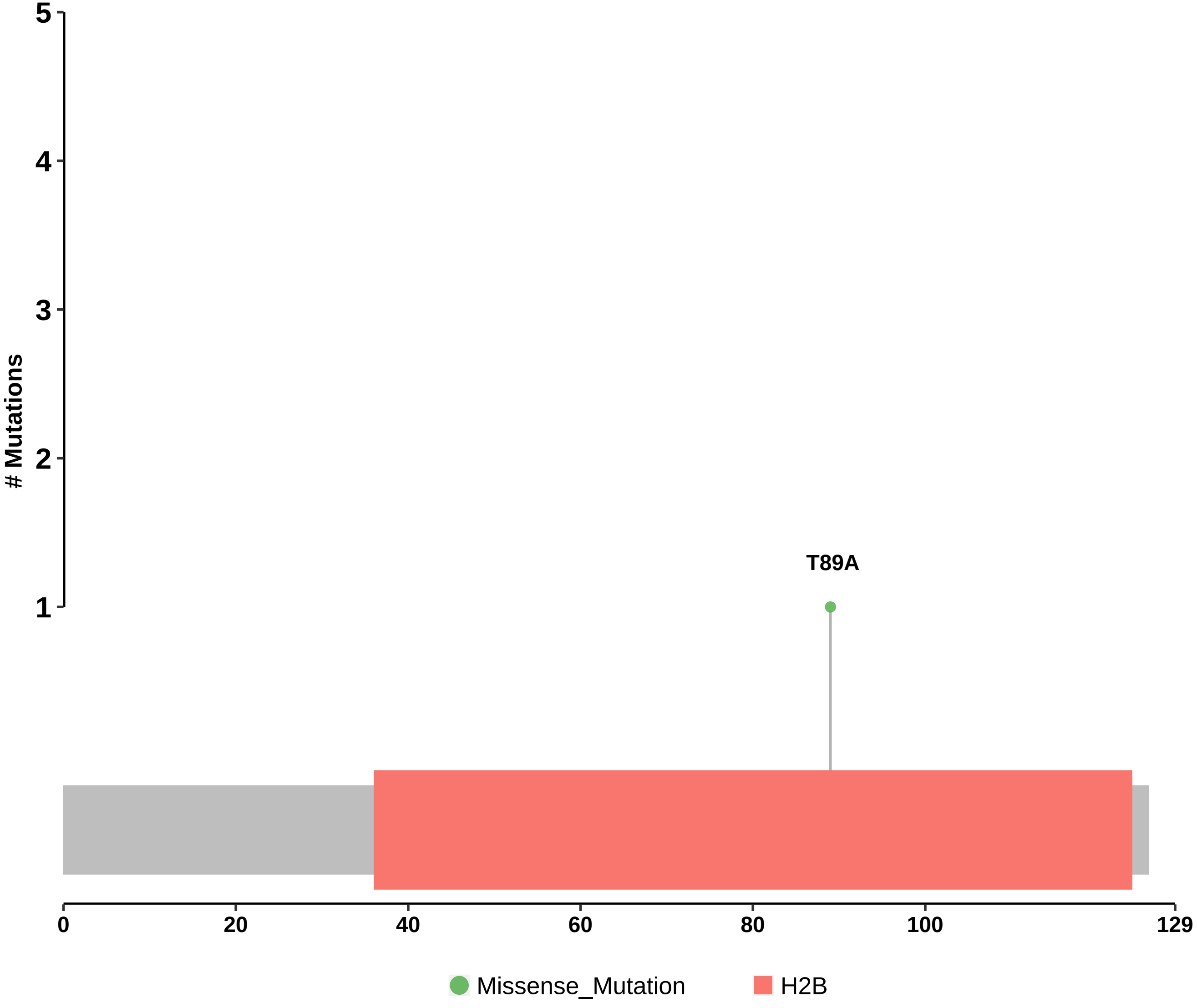

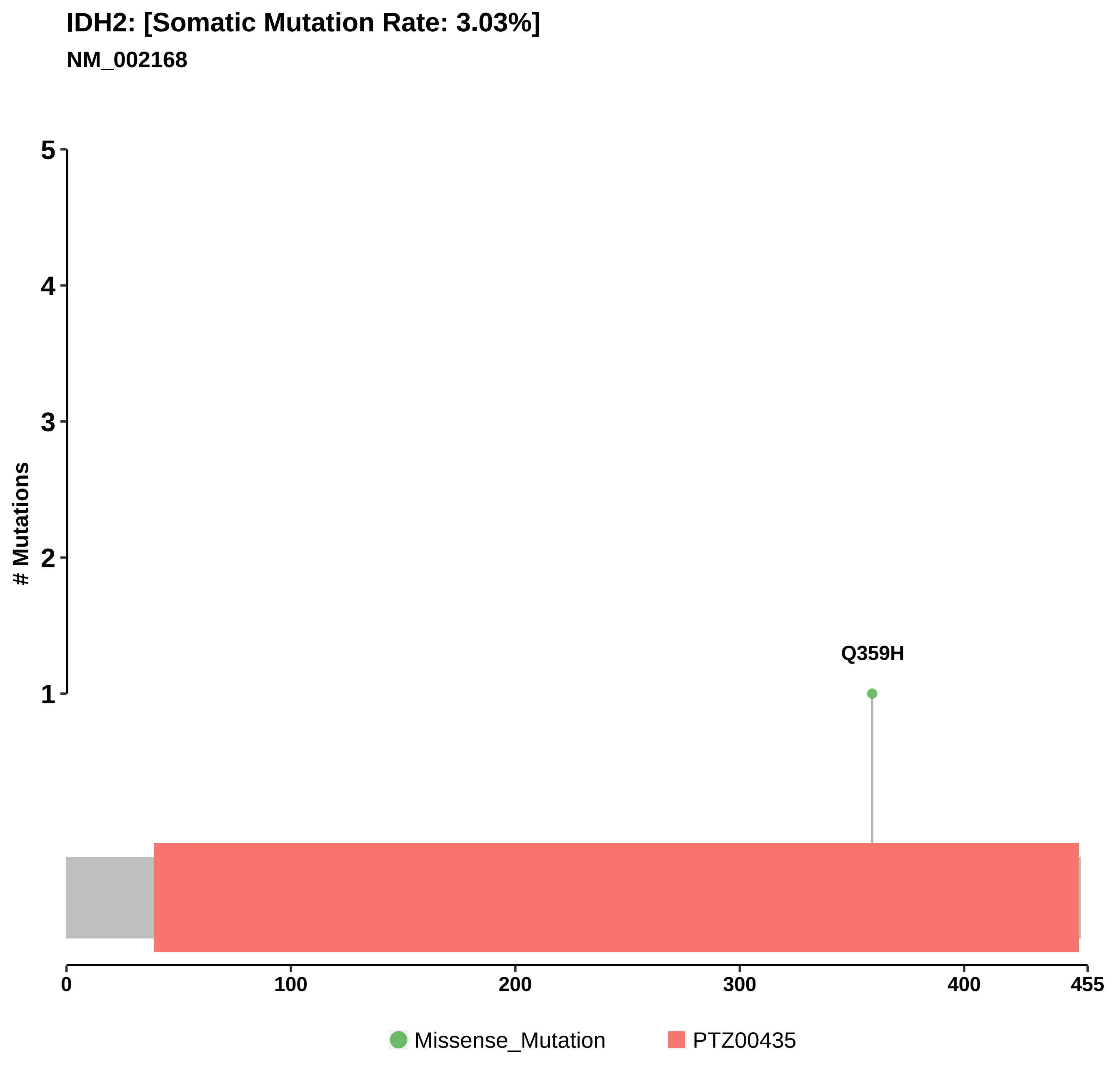
IDH2: [Somatic Mutation Rate: 3.03%]
NM_002168
Mutations
5
4
3
2
1
Q359H
0
100
200
300
400
455
Missense_Mutation
PTZ00435

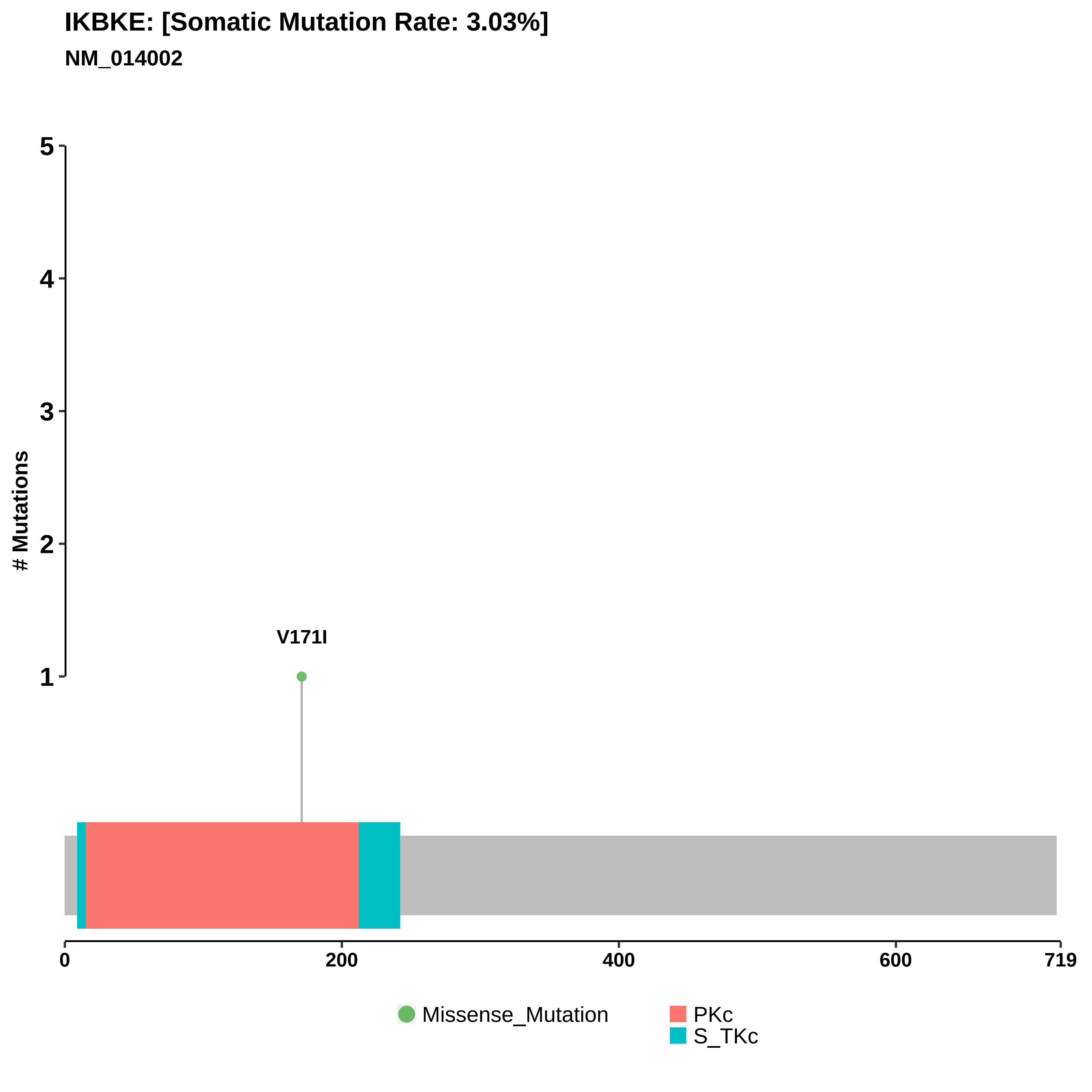
IKBKE: [Somatic Mutation Rate: 3.03%]
NM_014002
Mutations
5
4
3
2
1
V171I
0
200
400
600
719
Missense_Mutation
PKc
S_TKc

IL1B: [Somatic Mutation Rate: 3.03%]

NM_000576

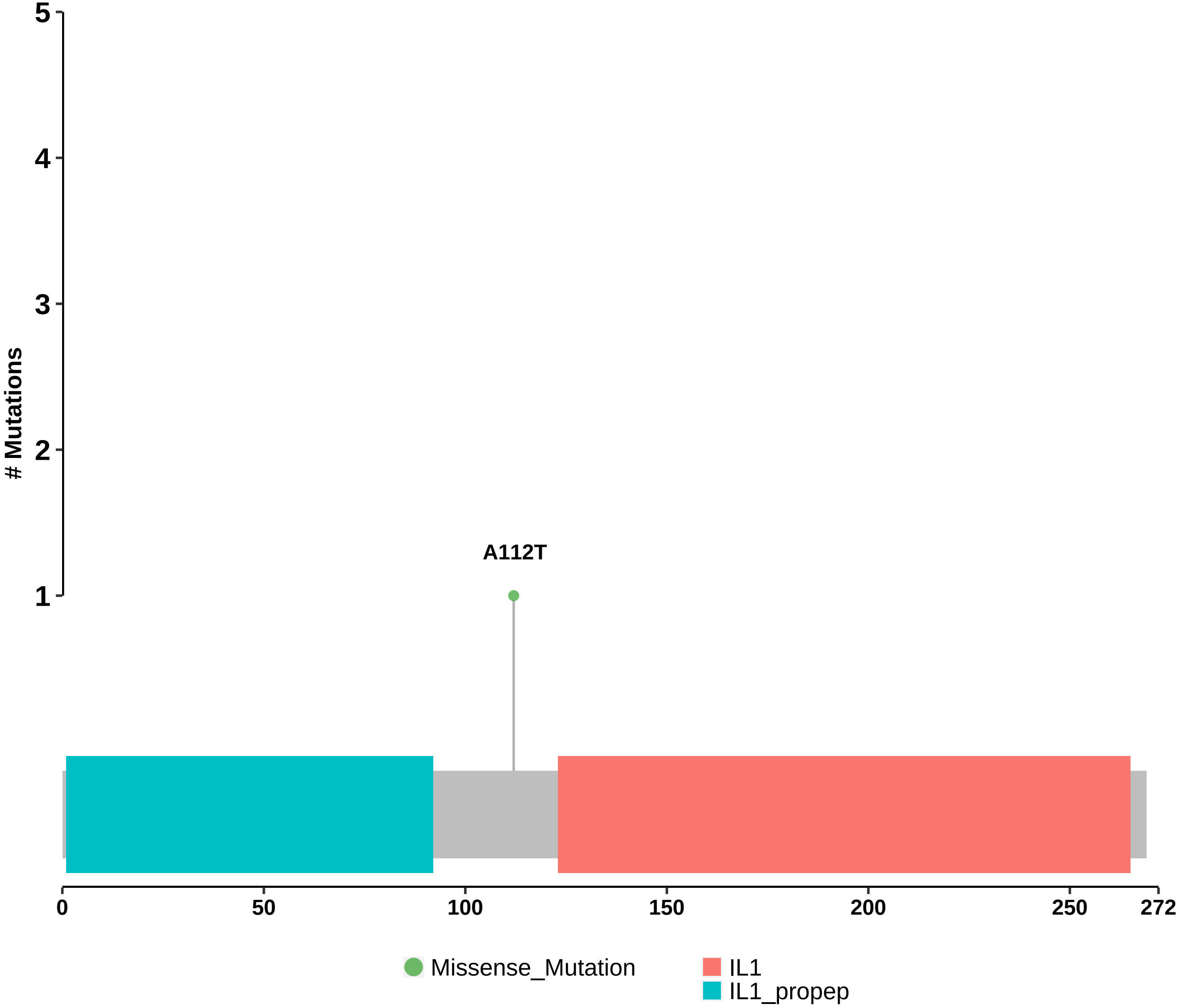

IRF7: [Somatic Mutation Rate: 3.03%]

NM_004031

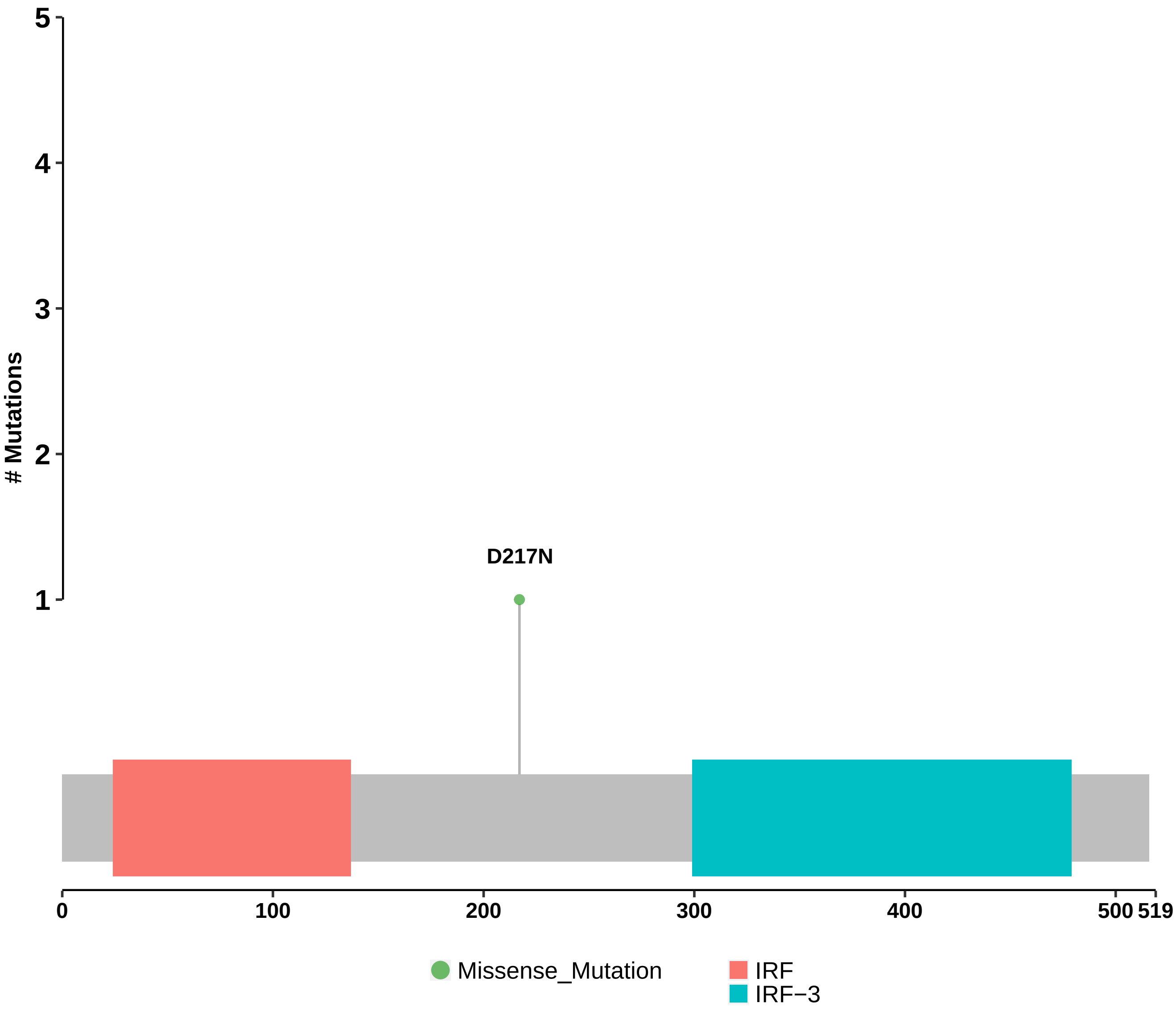

ISG15: [Somatic Mutation Rate: 3.03%]

NM_005101

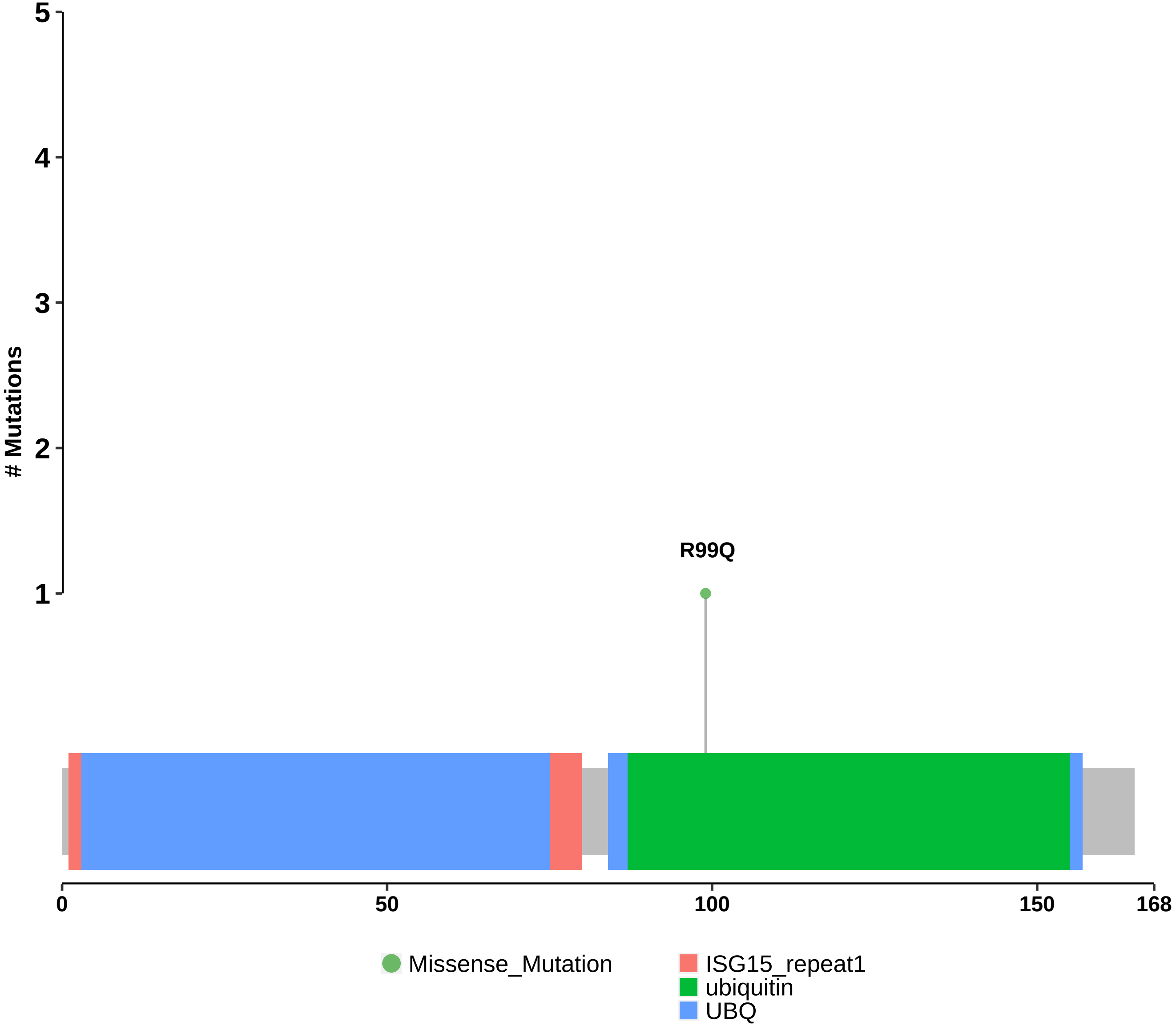

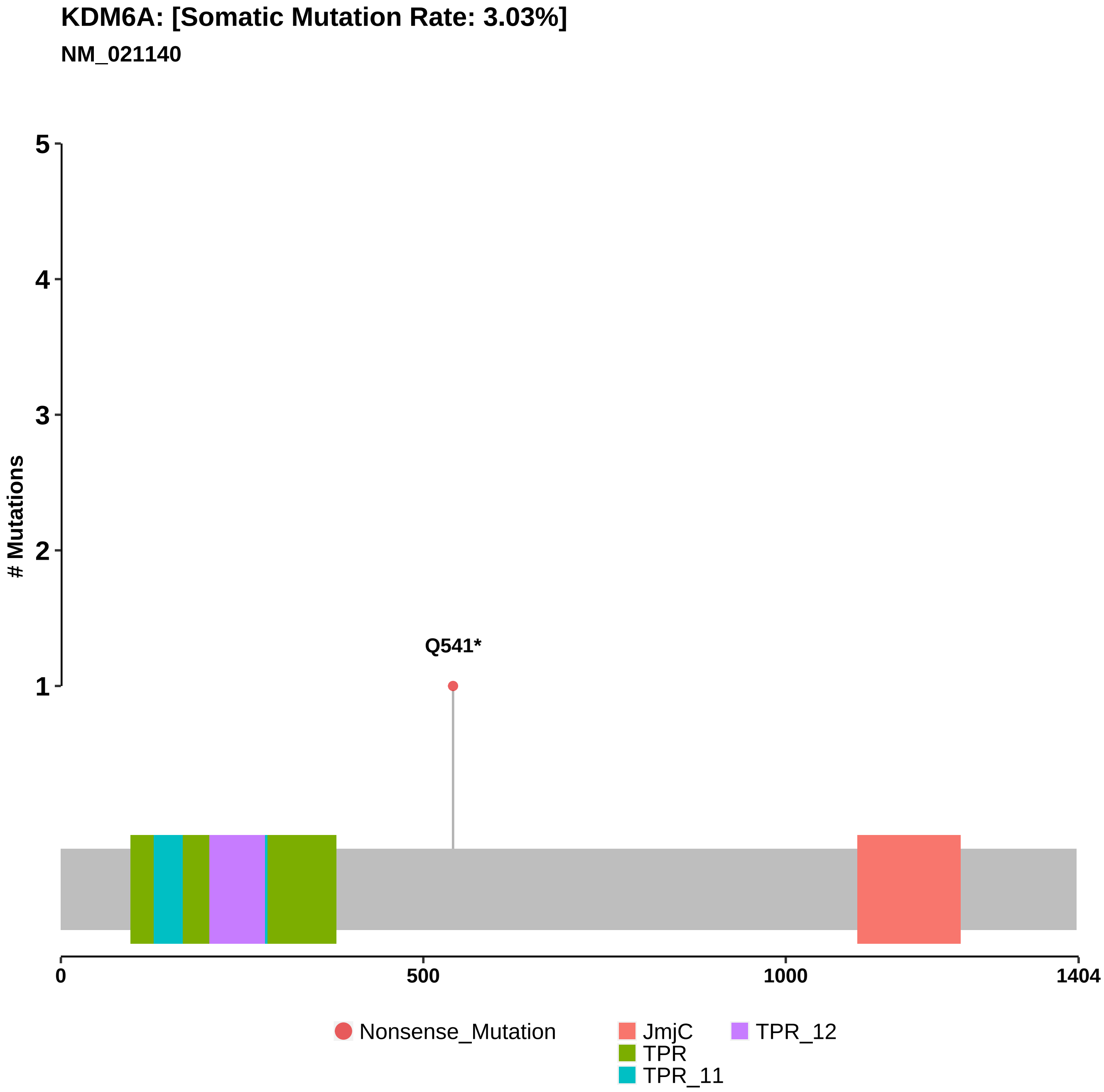
KDM6A: [Somatic Mutation Rate: 3.03%]
NM_021140
Mutations
5
4
3
2
1
Q541*
0
500
1000
1404
Nonsense_Mutation
JmjC
TPR
TPR_11
TPR_12

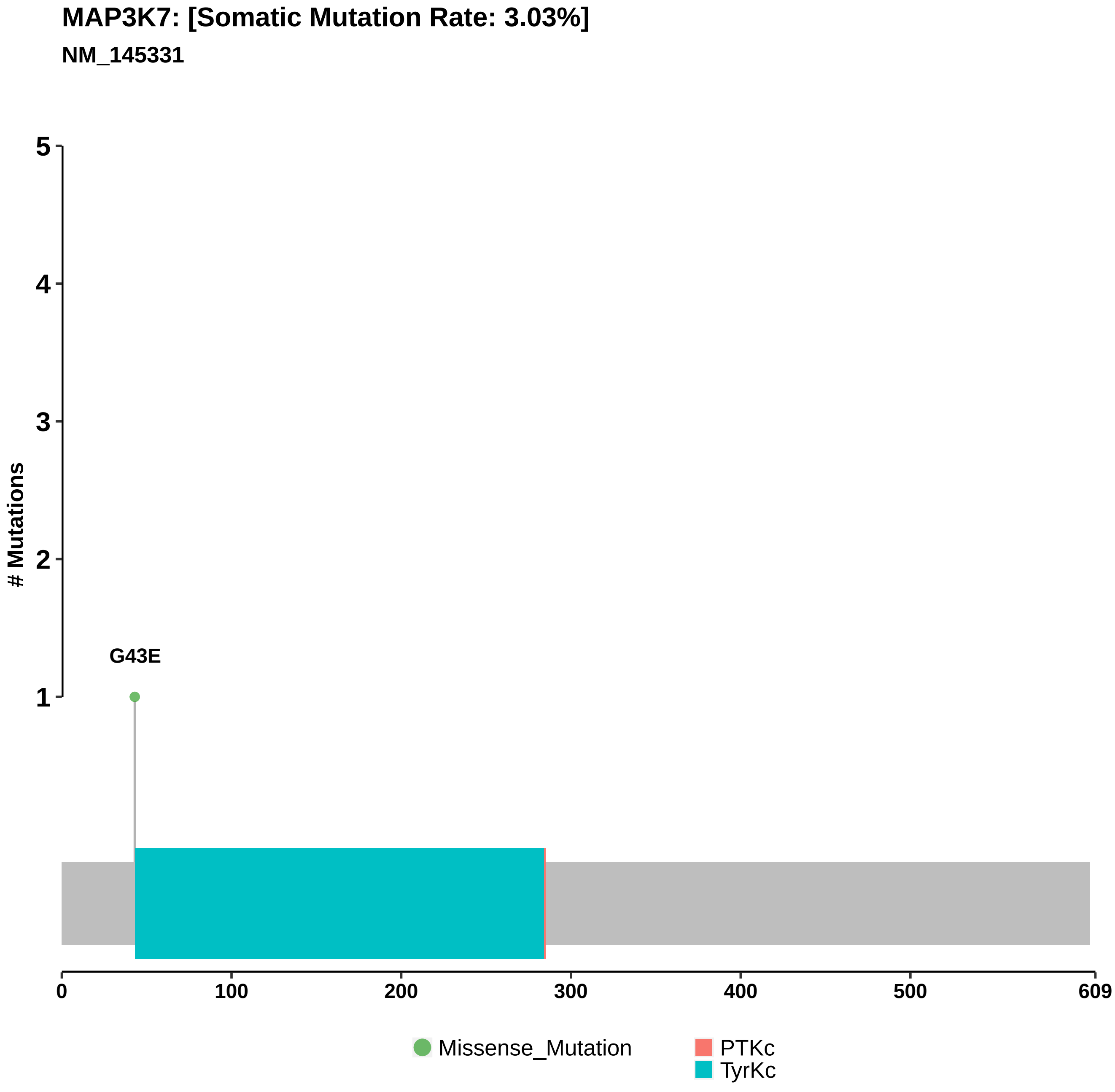
MAP3K7: [Somatic Mutation Rate: 3.03%]
NM_145331
Mutations
5
4
3
2
1
G43E
0
100
200
300
400
500
609
Missense_Mutation
PTKc
TyrKc

NSD1: [Somatic Mutation Rate: 3.03%]

NM_022455

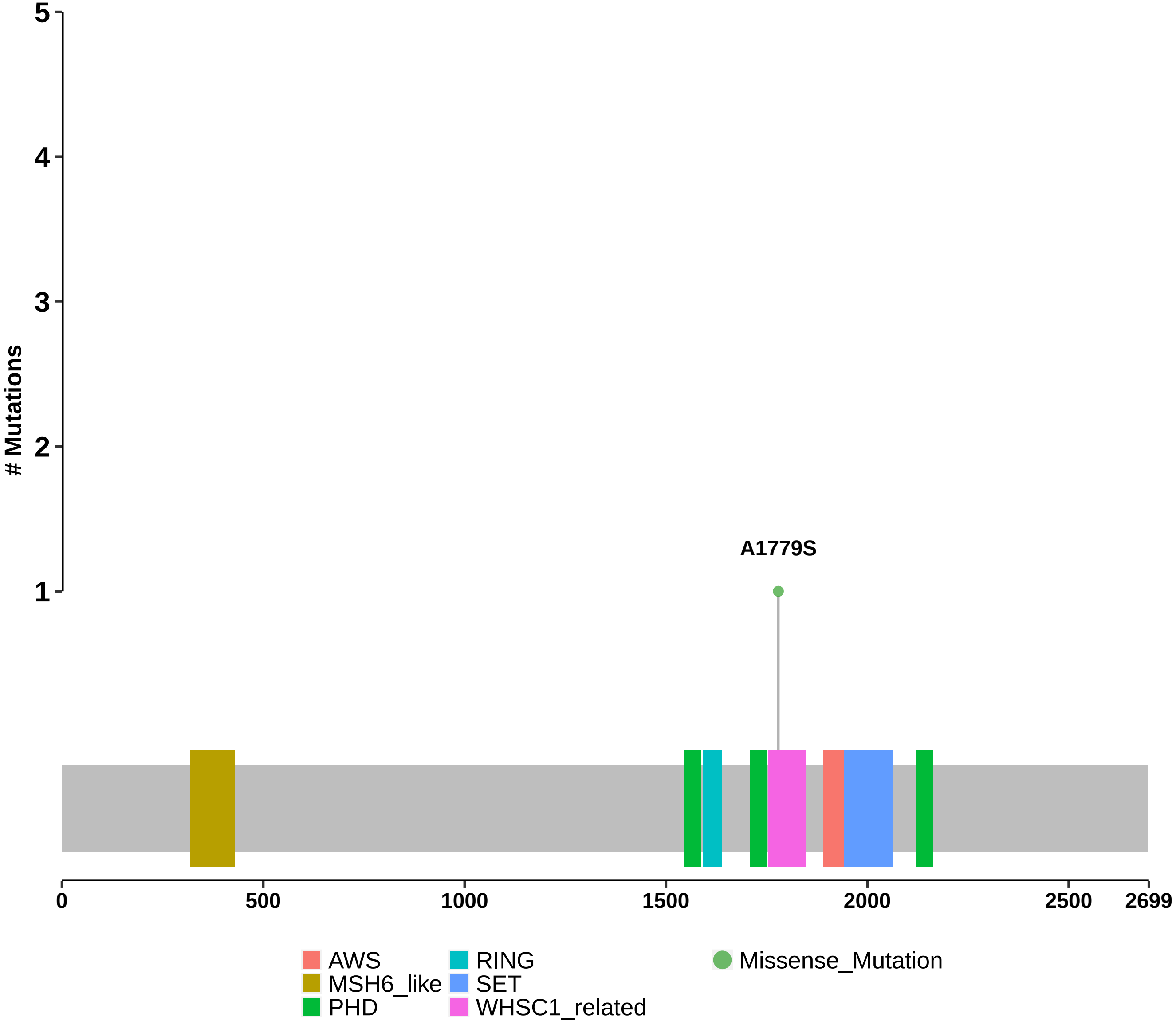

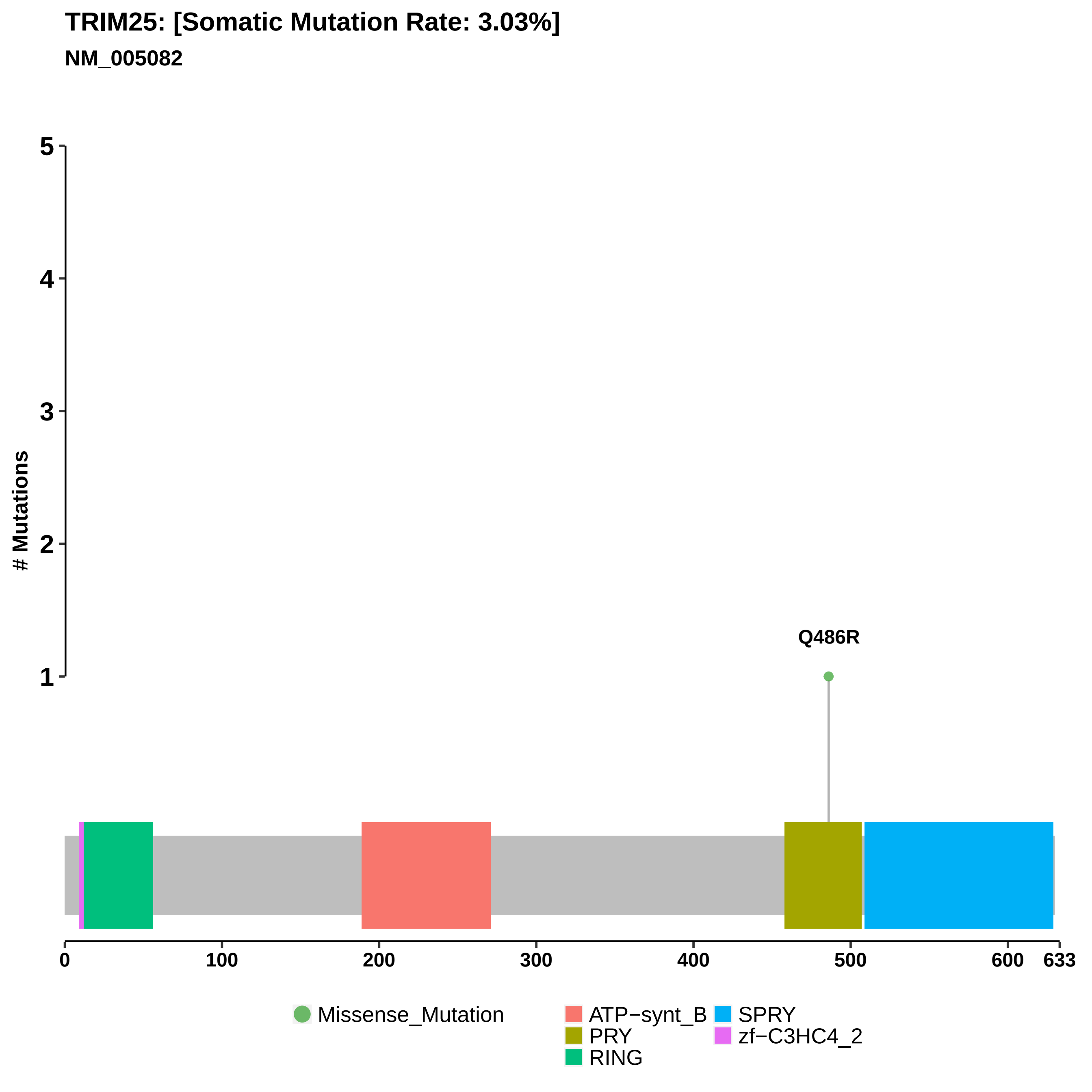
TRIM25: [Somatic Mutation Rate: 3.03%]
NM_005082
Mutations
5
4
3
2
1
Q486R
0
100
200
300
400
500
600
633
Missense_Mutation
ATP−synt_B
PRY
RING
SPRY
zf−C3HC4_2

ZBP1: [Somatic Mutation Rate: 3.03%]

NM_030776

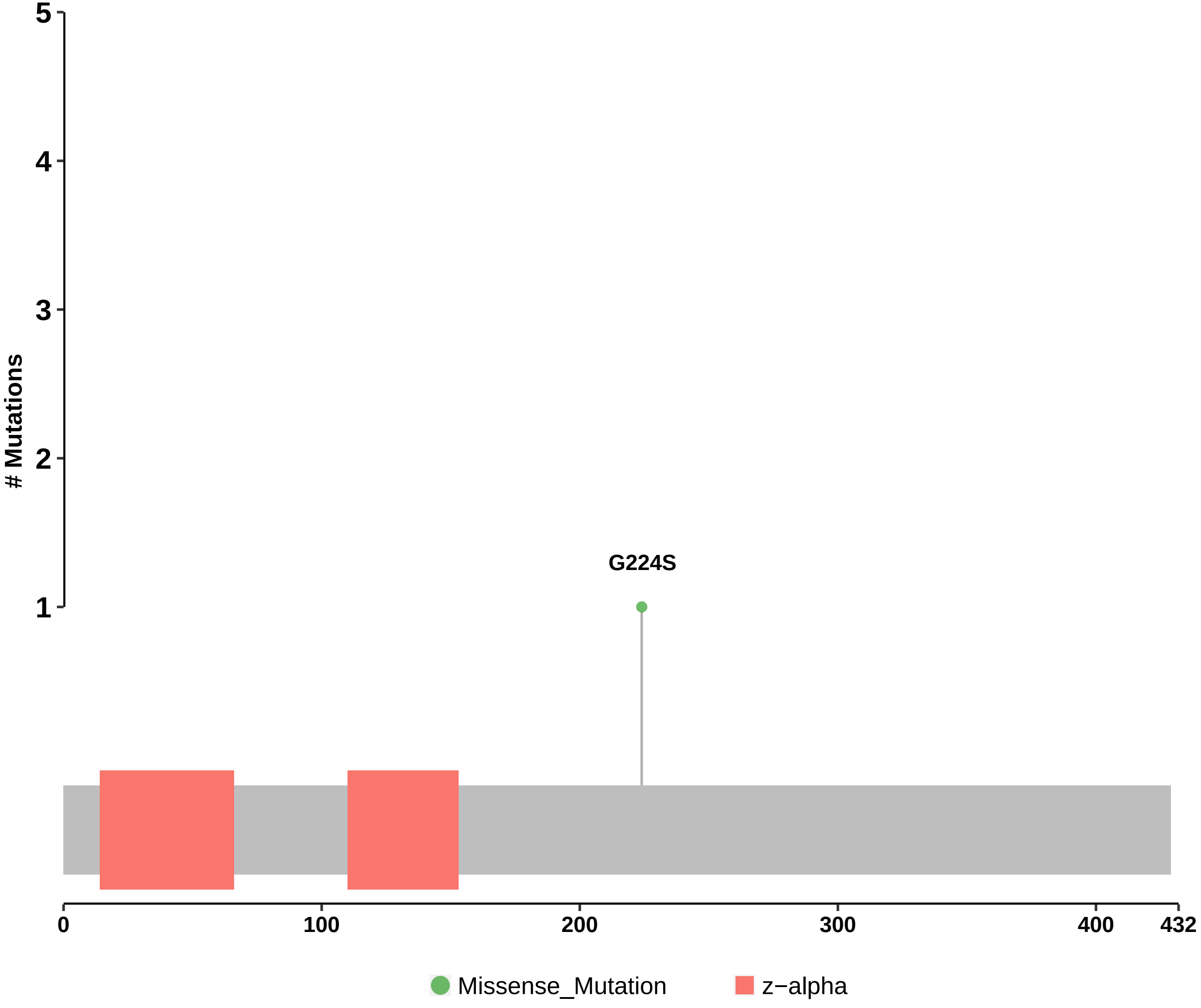

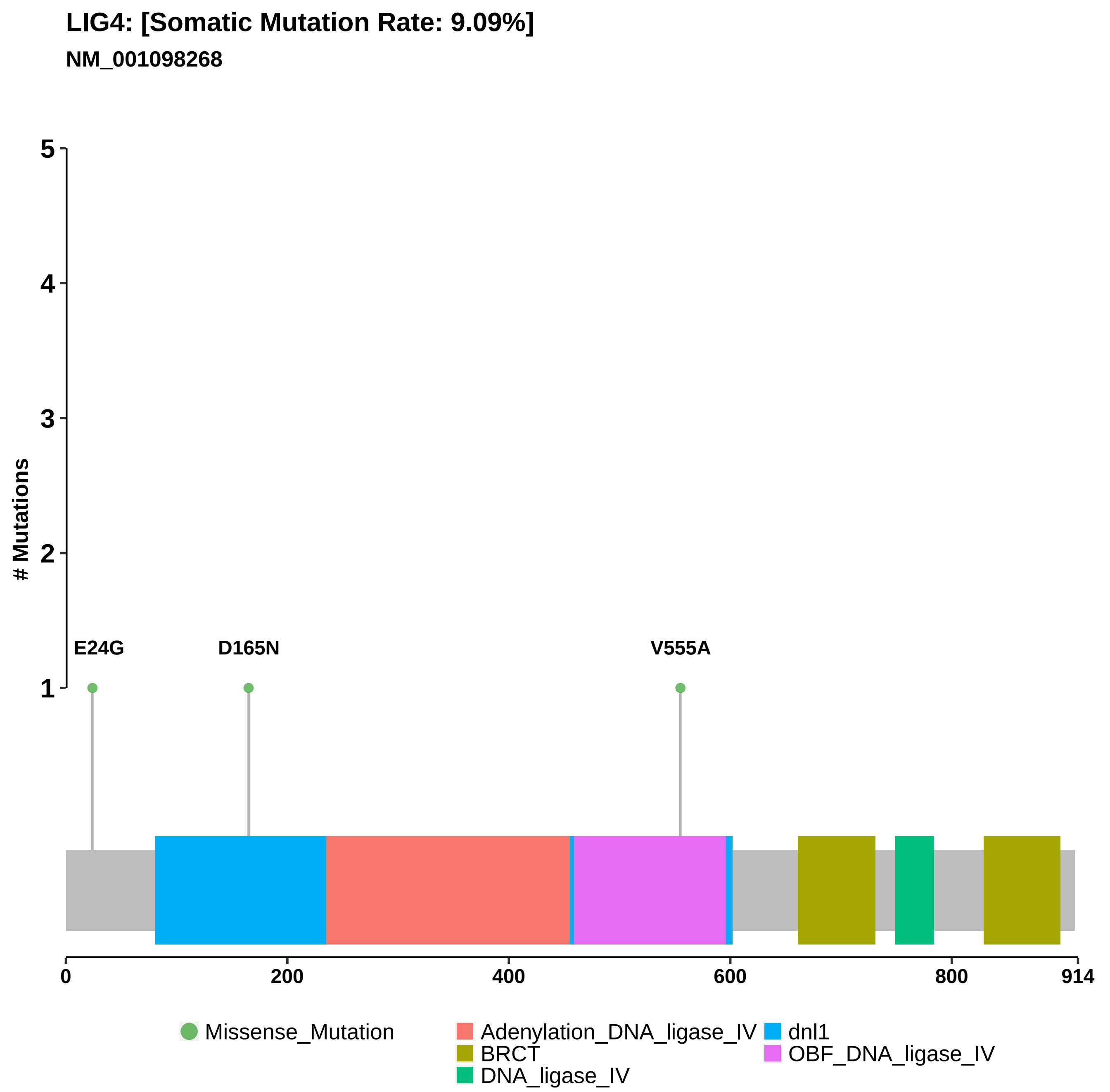

LIG4: [Somatic Mutation Rate: 9.09%]
NM_001098268
Mutations
5
4
3
2
1
E24G
D165N
V555A
0
200
400
600
800
914
Missense_Mutation
Adenylation_DNA_ligase_IV
BRCT
DNA_ligase_IV
dnl1
OBF_DNA_ligase_IV

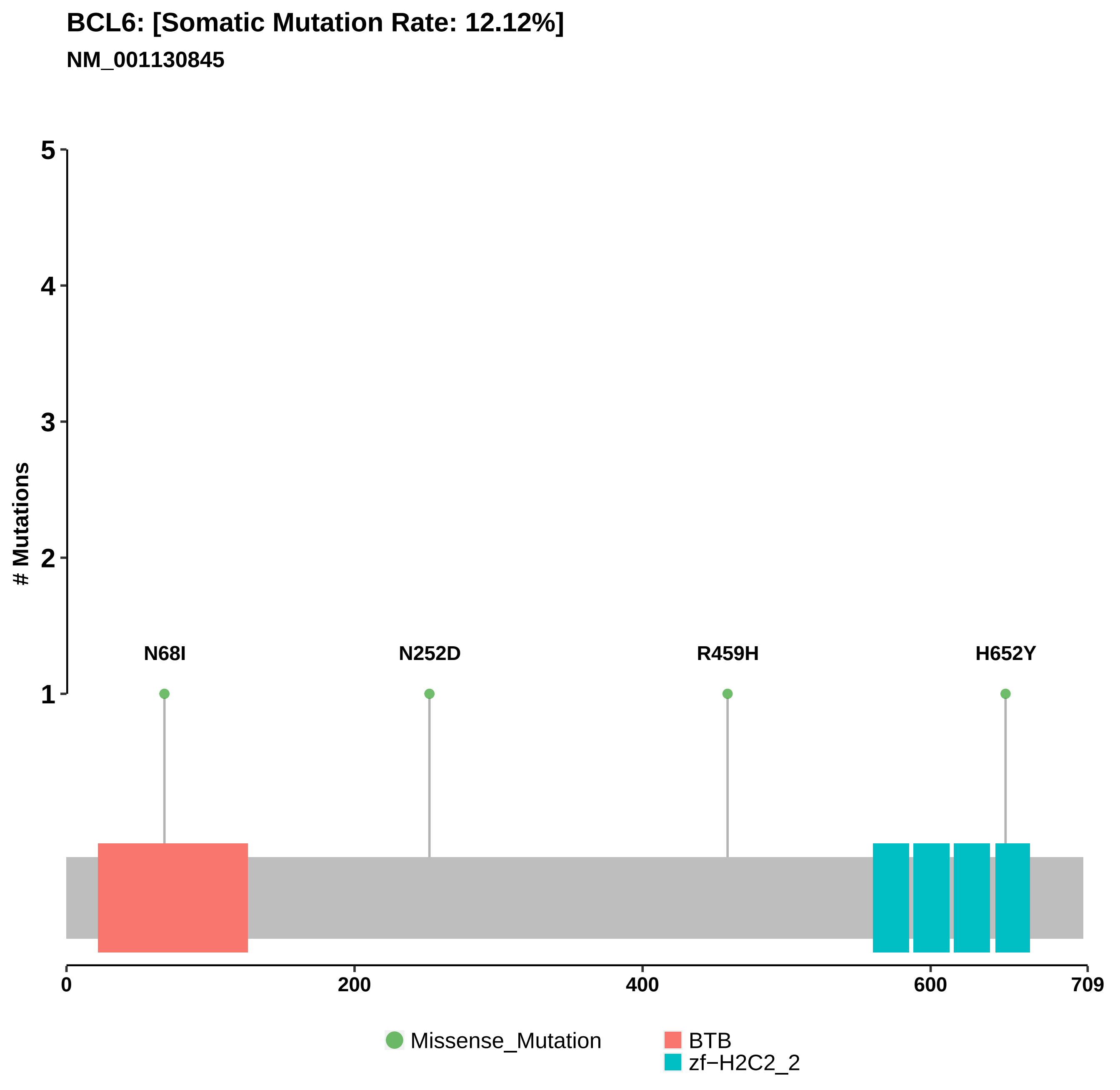

BCL6: [Somatic Mutation Rate: 12.12%]
NM_001130845
Mutations
5
4
3
2
1
N68I
N252D
R459H
H652Y
0
200
400
600
709
Missense_Mutation
BTB
zf−H2C2_2

BRAF: [Somatic Mutation Rate: 15.15%]

NM_004333

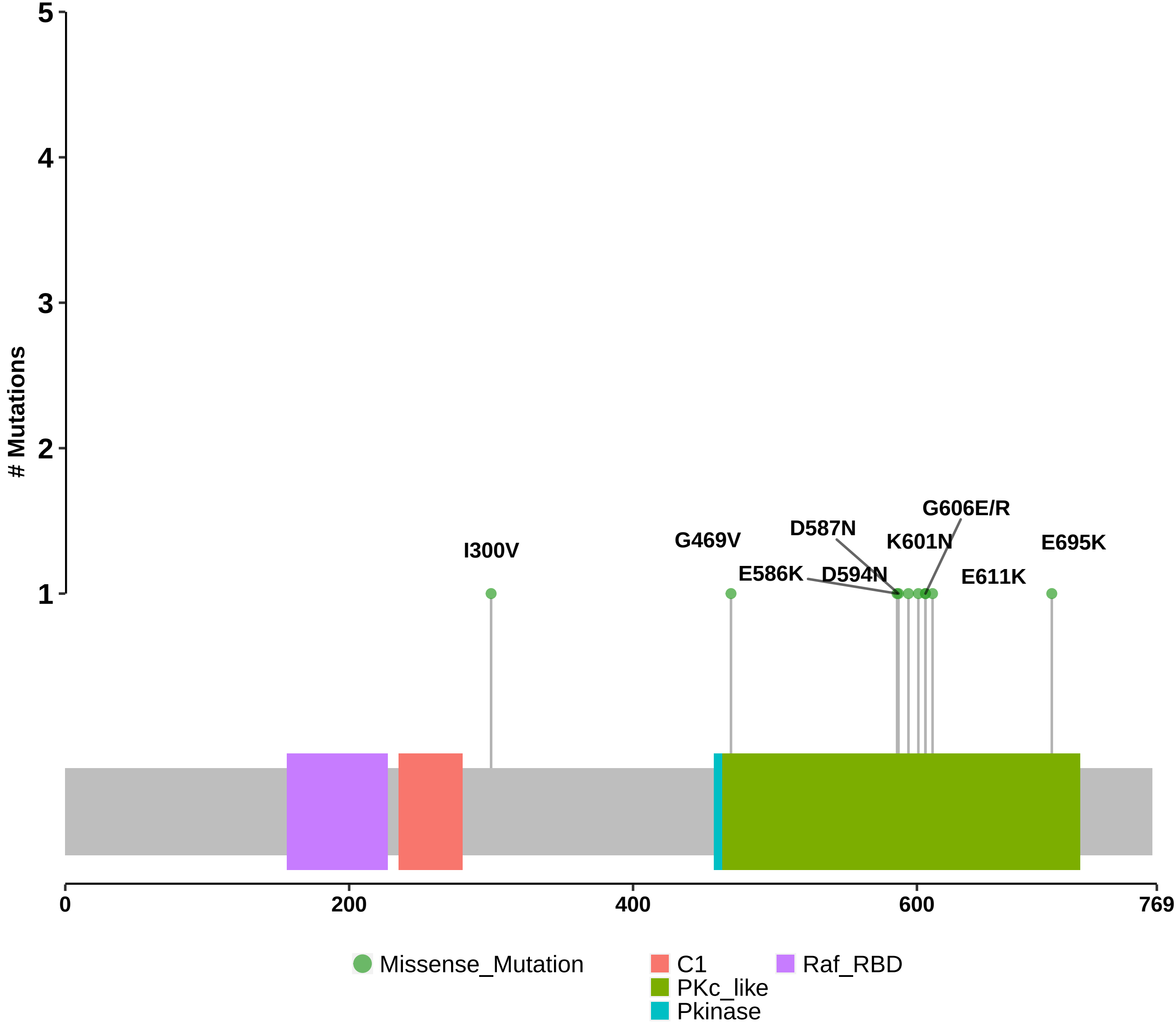

FAM46C: [Somatic Mutation Rate: 15.15%]

NM_017709

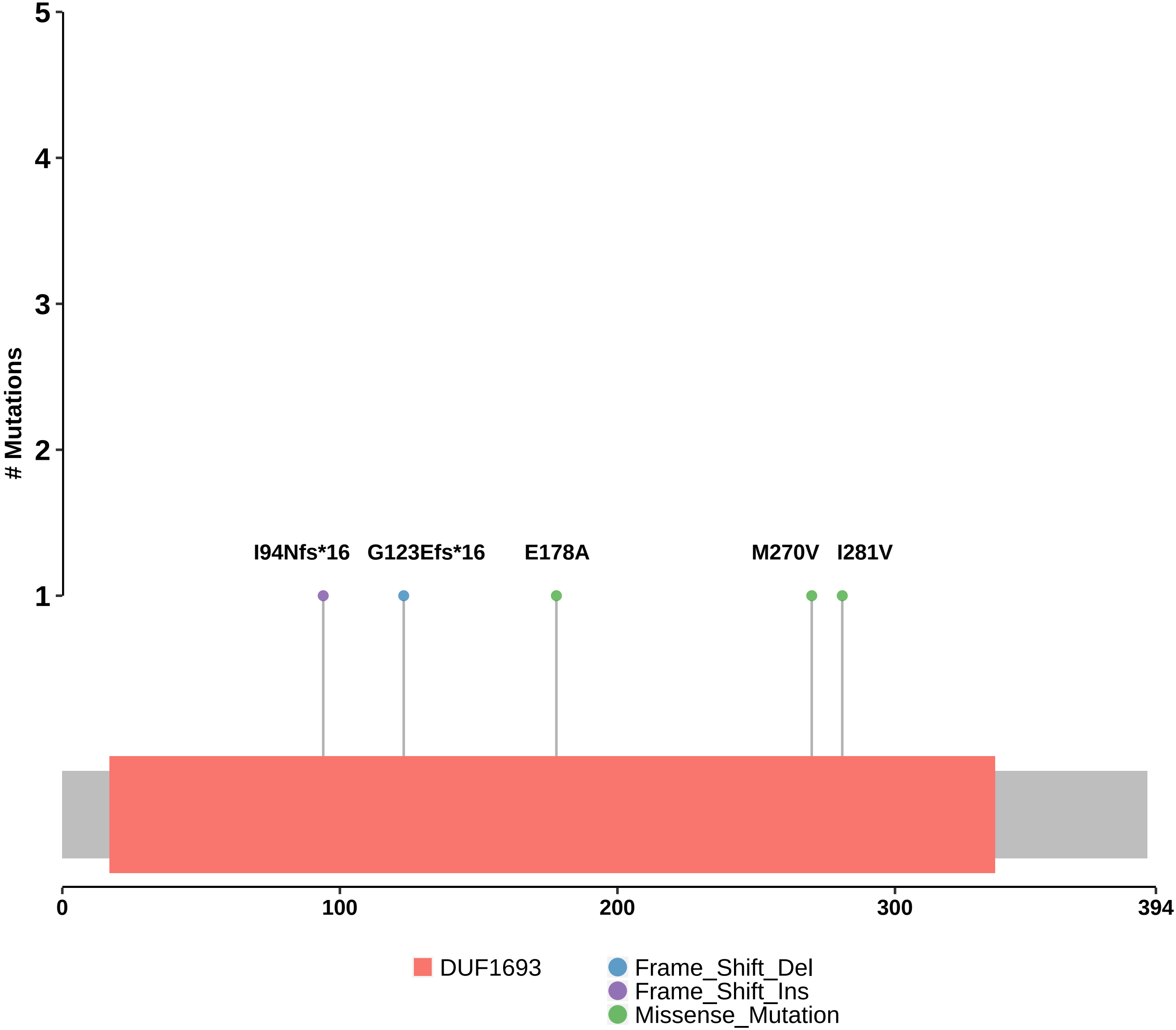

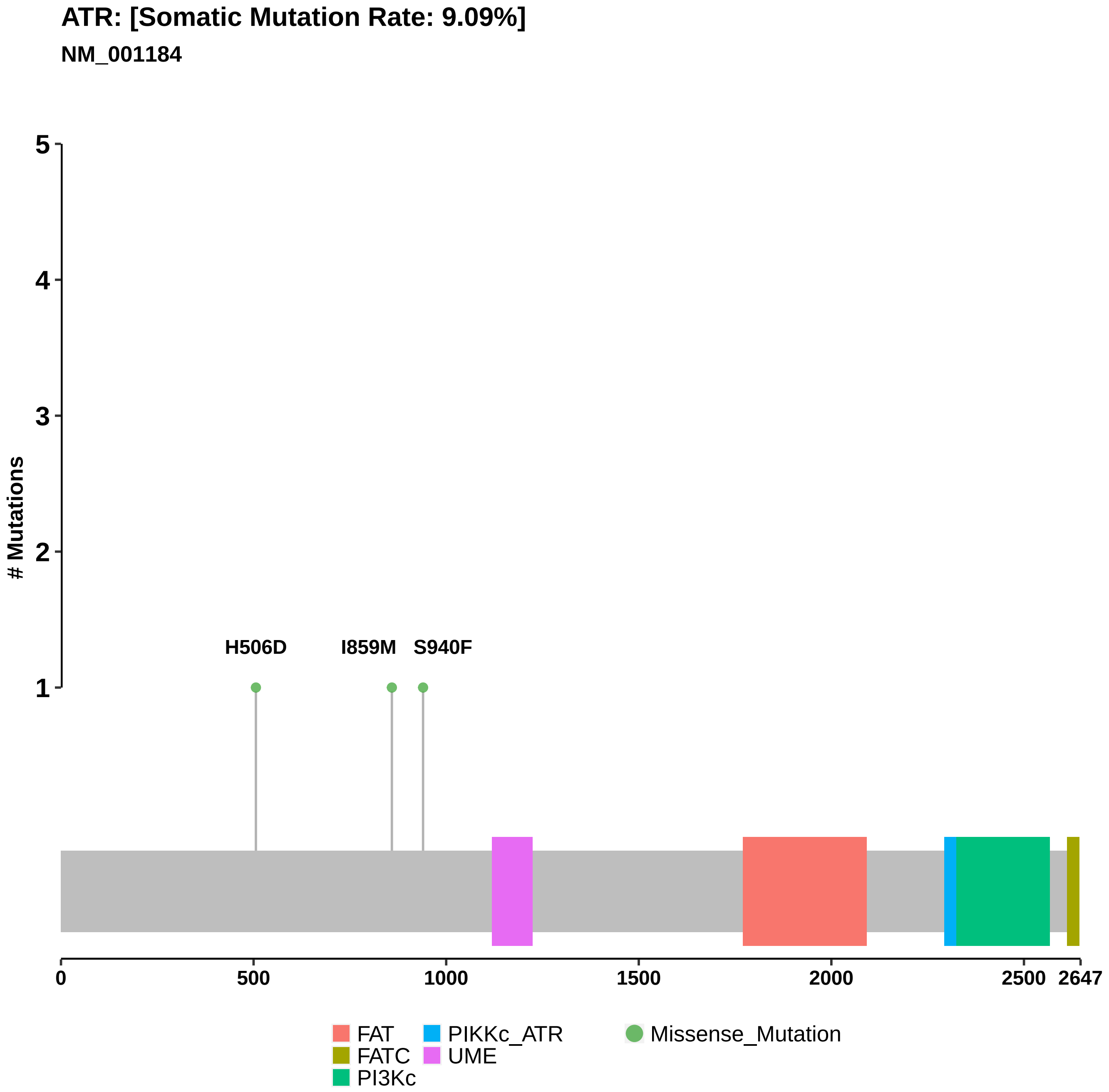
ATR: [Somatic Mutation Rate: 9.09%]
NM_001184
Mutations
5
4
3
2
1
H506D
I859M
S940F
0
500
1000
1500
2000
2500
2647
FAT
FATC
PI3Kc
PIKKc_ATR
UME
Missense_Mutation

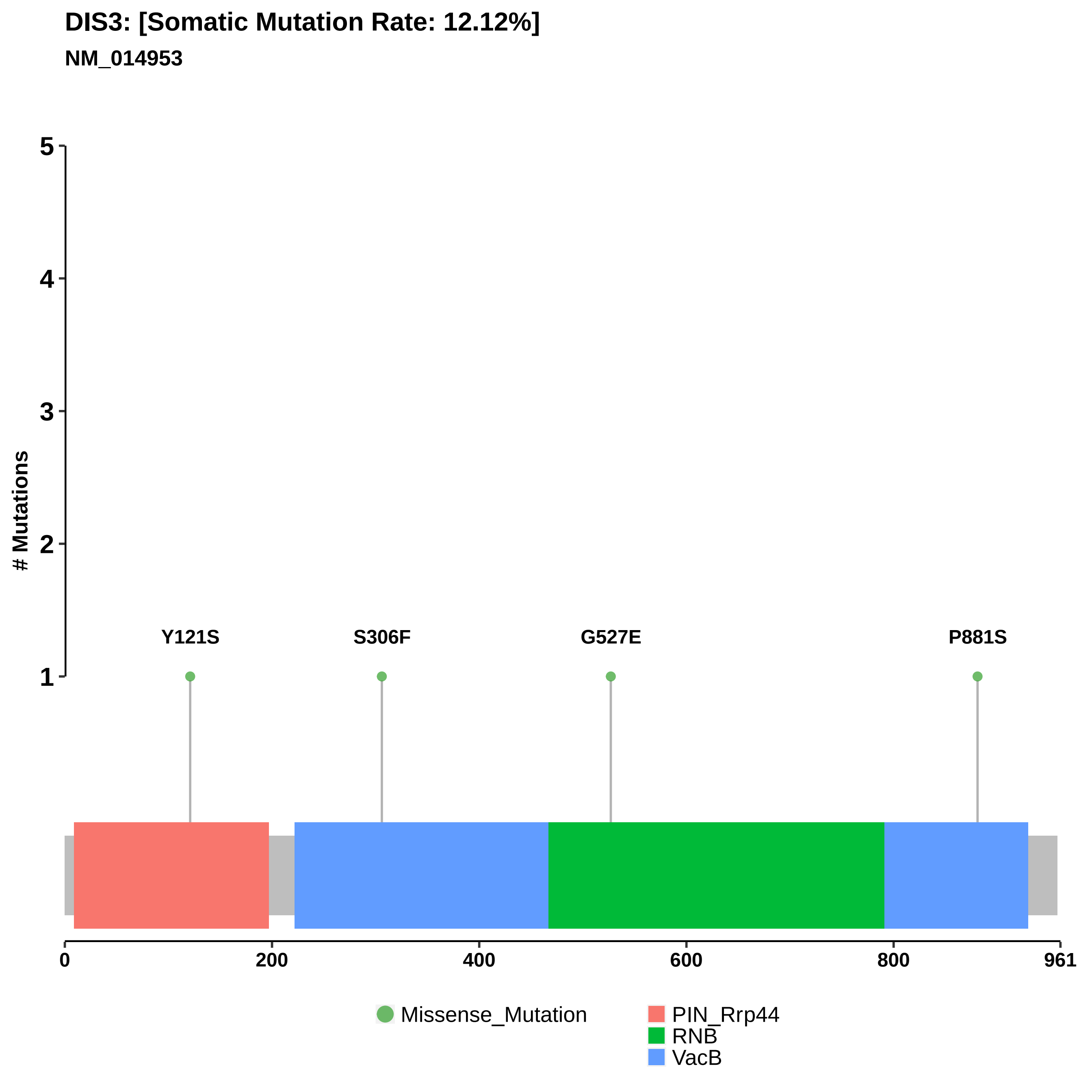
DIS3: [Somatic Mutation Rate: 12.12%]
NM_014953
Mutations
5
4
3
2
1
Y121S
S306F
G527E
P881S
0
200
400
600
800
961
Missense_Mutation
PIN_Rrp44
RNB
VacB

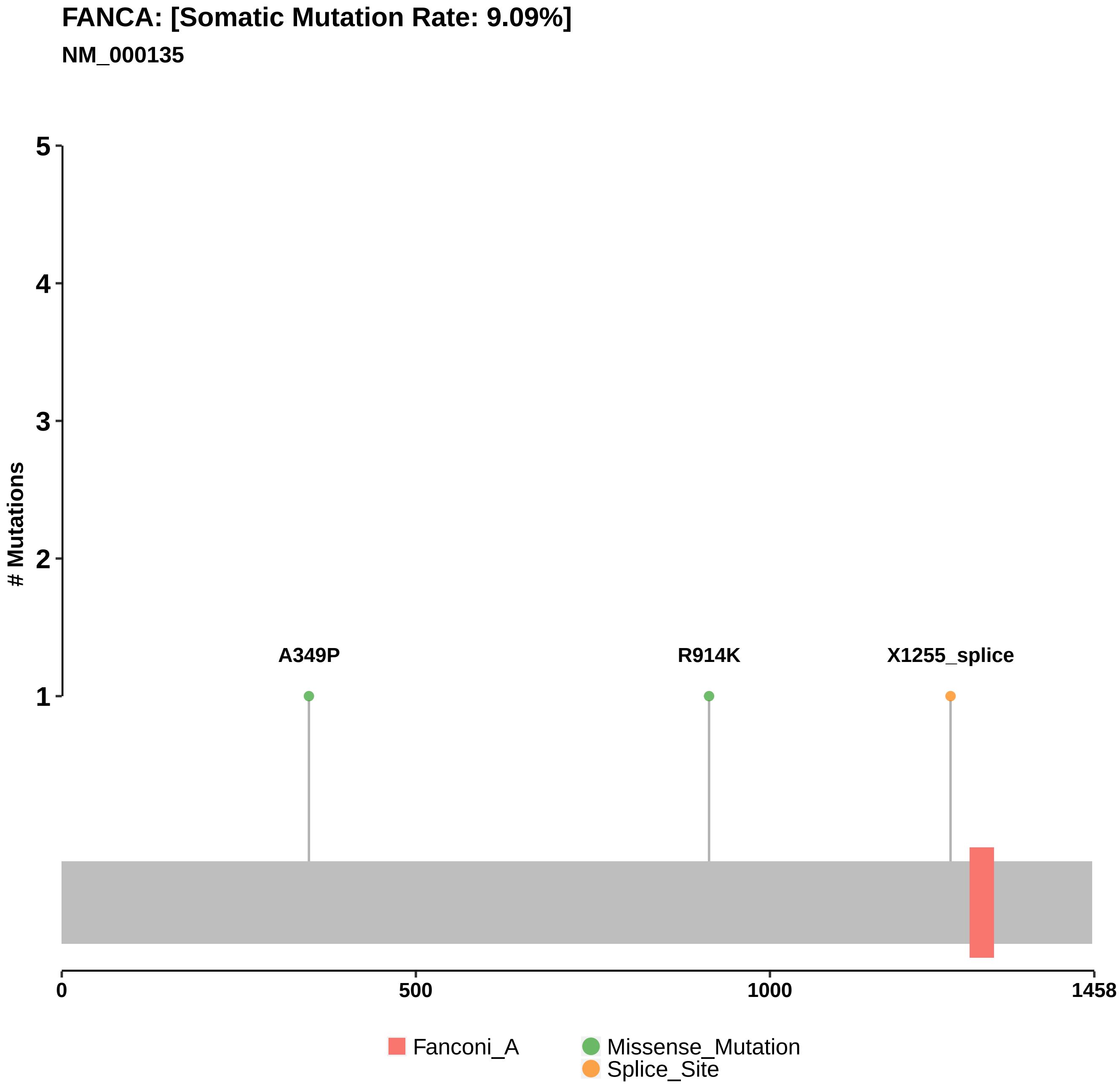
FANCA: [Somatic Mutation Rate: 9.09%]
NM_000135
Mutations
5
4
3
2
1
A349P
R914K
X1255_splice
0
500
1000
1458
Fanconi_A
Missense_Mutation
Splice_Site

FANCI: [Somatic Mutation Rate: 12.12%]

NM_001113378

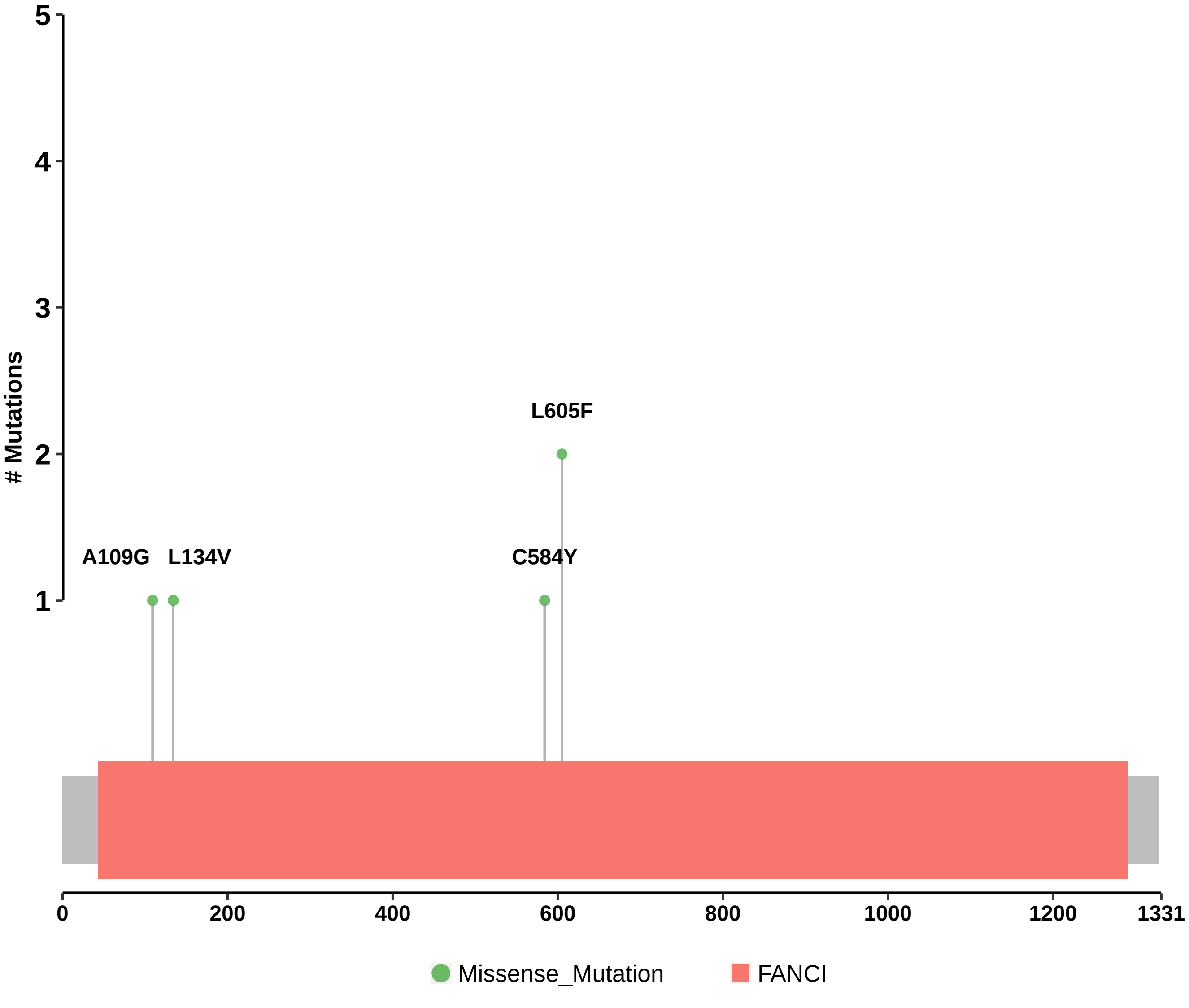

FANCM: [Somatic Mutation Rate: 9.09%]

NM_020937

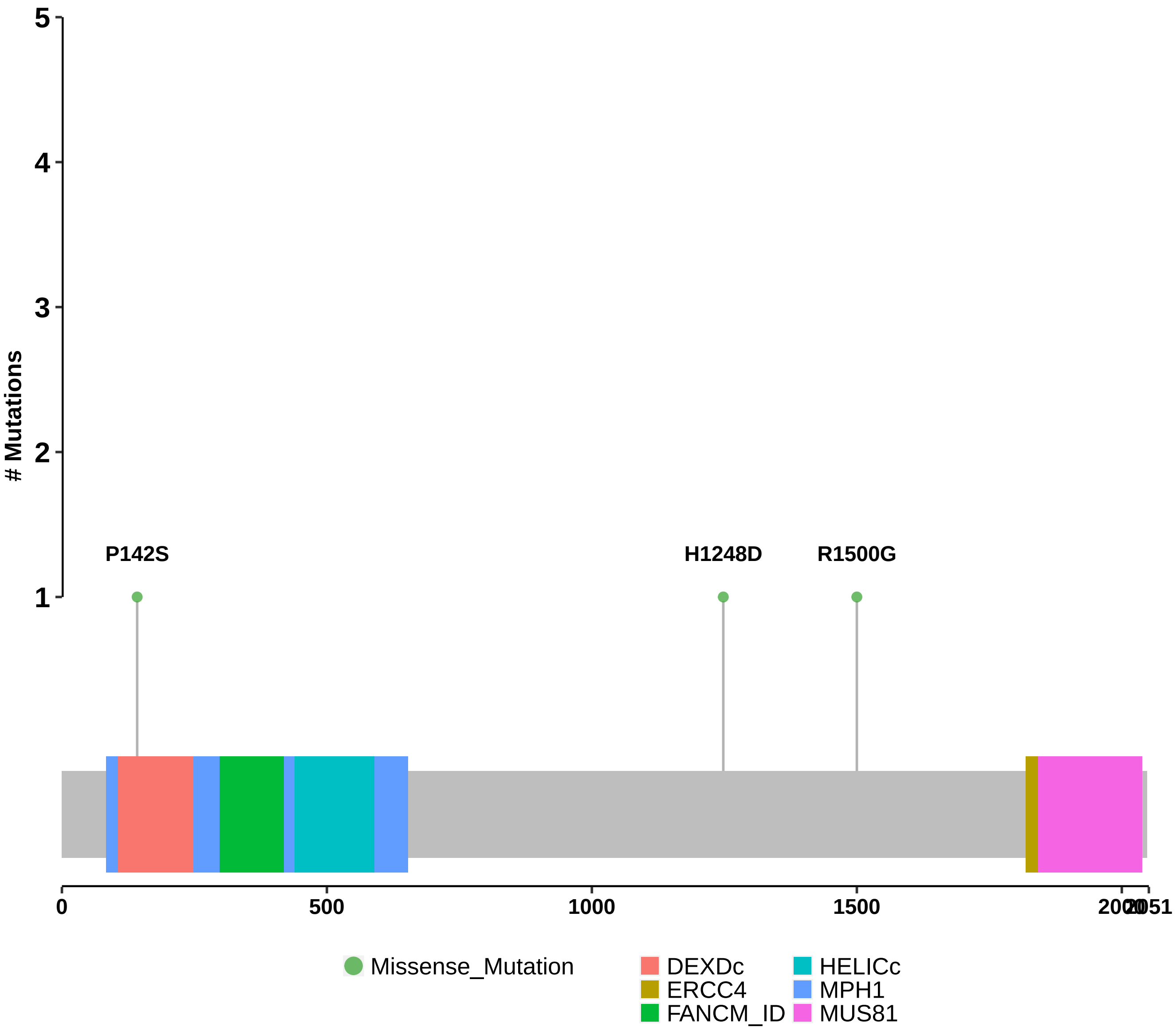

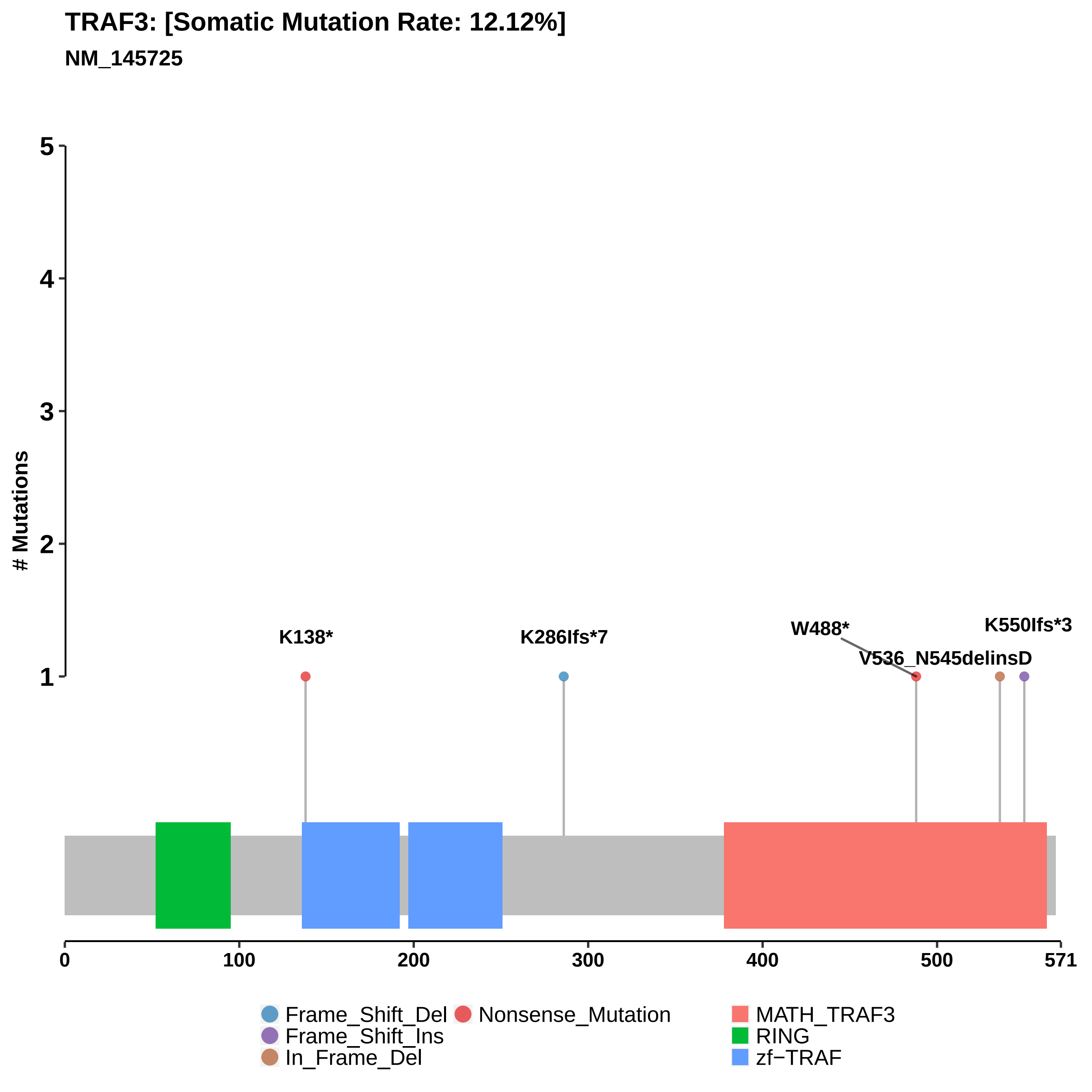
TRAF3: [Somatic Mutation Rate: 12.12%]
NM_145725
Mutations
5
4
3
2
1
K138*
K286Ifs*7
W488*
V536_N545delinsD
K550Ifs*3
0
100
200
300
400
500
571
Frame_Shift_Del
Frame_Shift_Ins
In_Frame_Del
Nonsense_Mutation
MATH_TRAF3
RING
zf−TRAF

CYLD: [Somatic Mutation Rate: 9.09%]

NM_015247

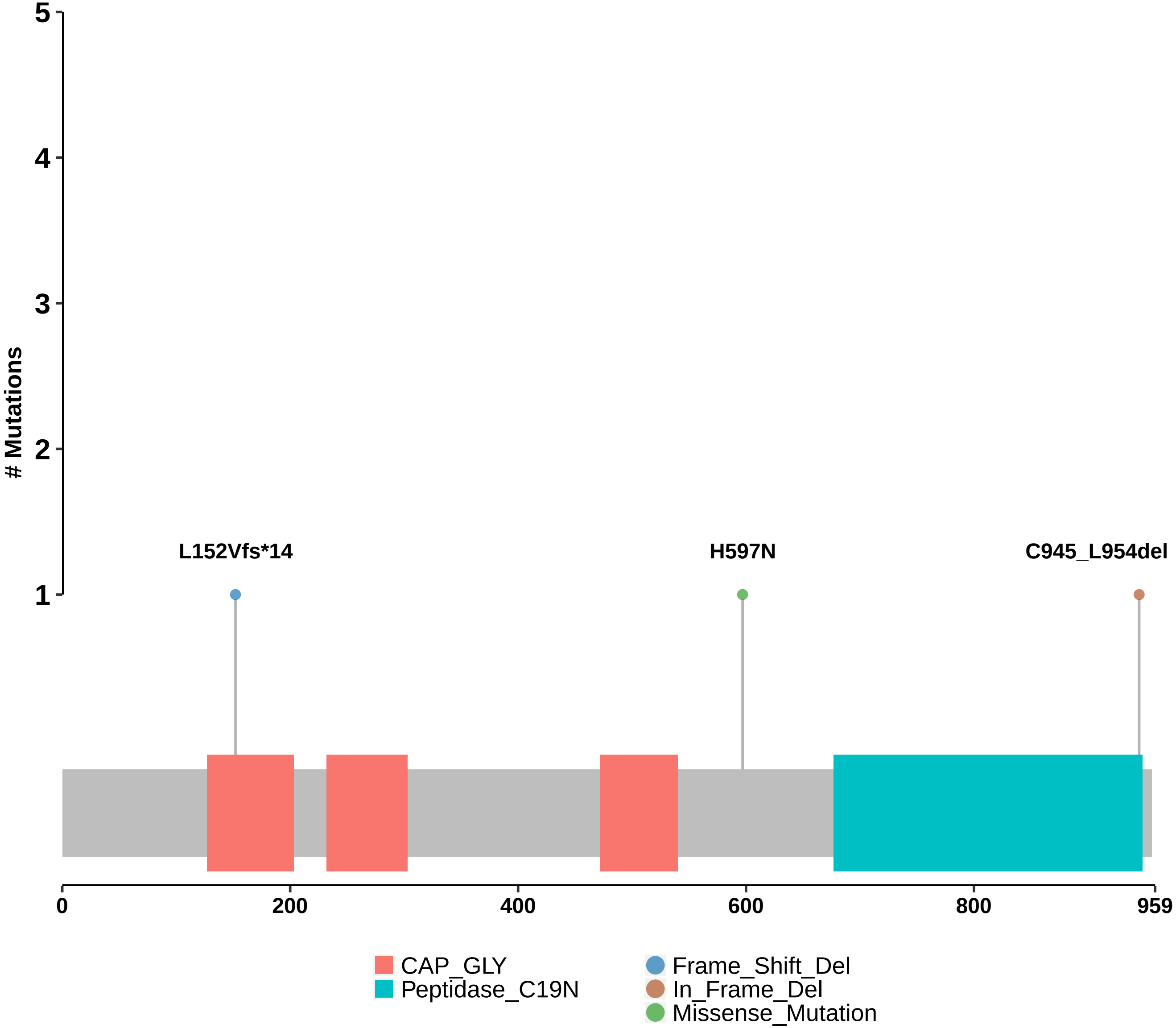

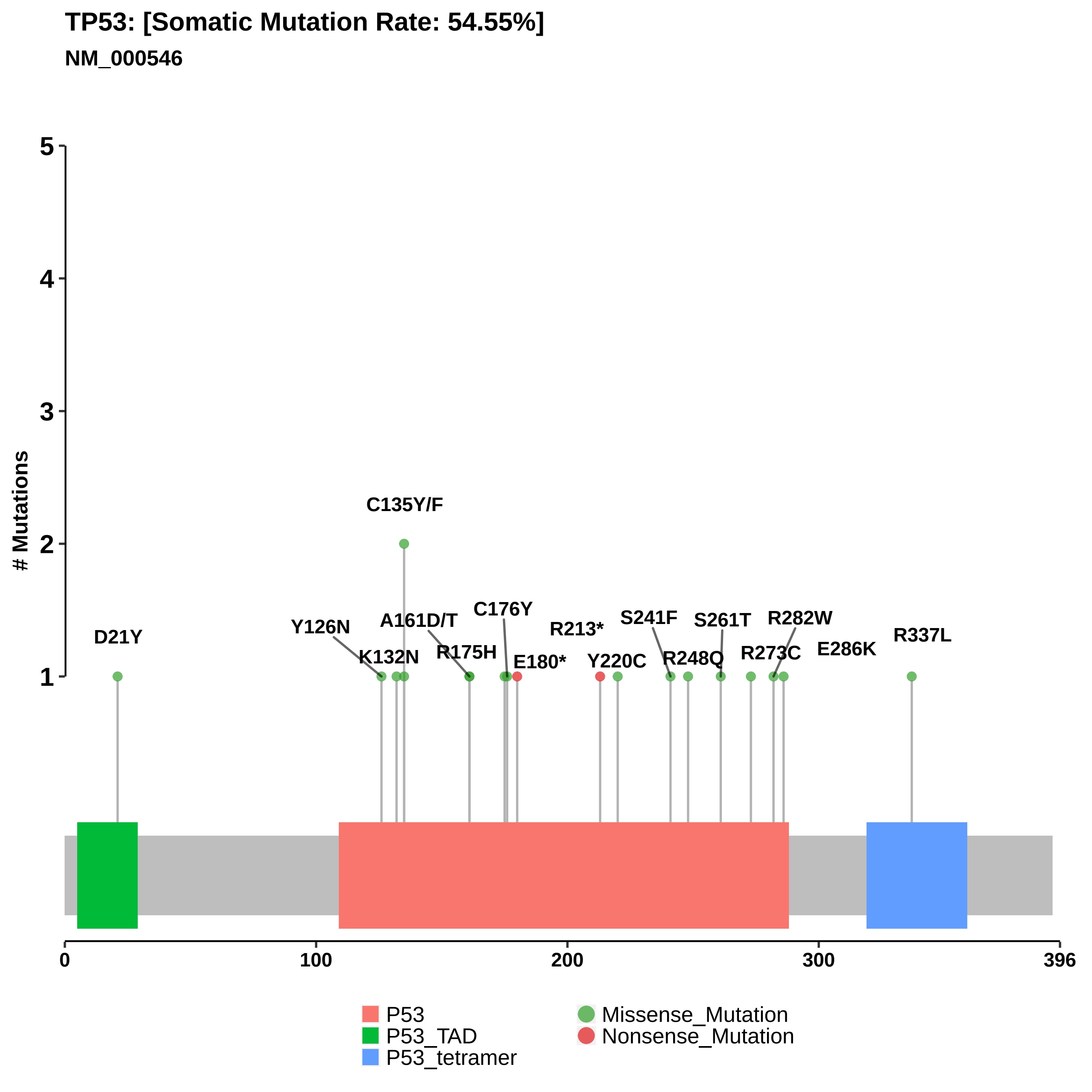
TP53: [Somatic Mutation Rate: 54.55%]
NM_000546
Mutations
5
4
3
2
1
C135Y/F
D21Y
Y126N
K132N
A161D/T
R175H
C176Y
E180*
R213*
Y220C
S241F
R248Q
S261T
R273C
R282W
E286K
R337L
0
100
200
300
396
P53
P53_TAD
P53_tetramer
Missense_Mutation
Nonsense_Mutation

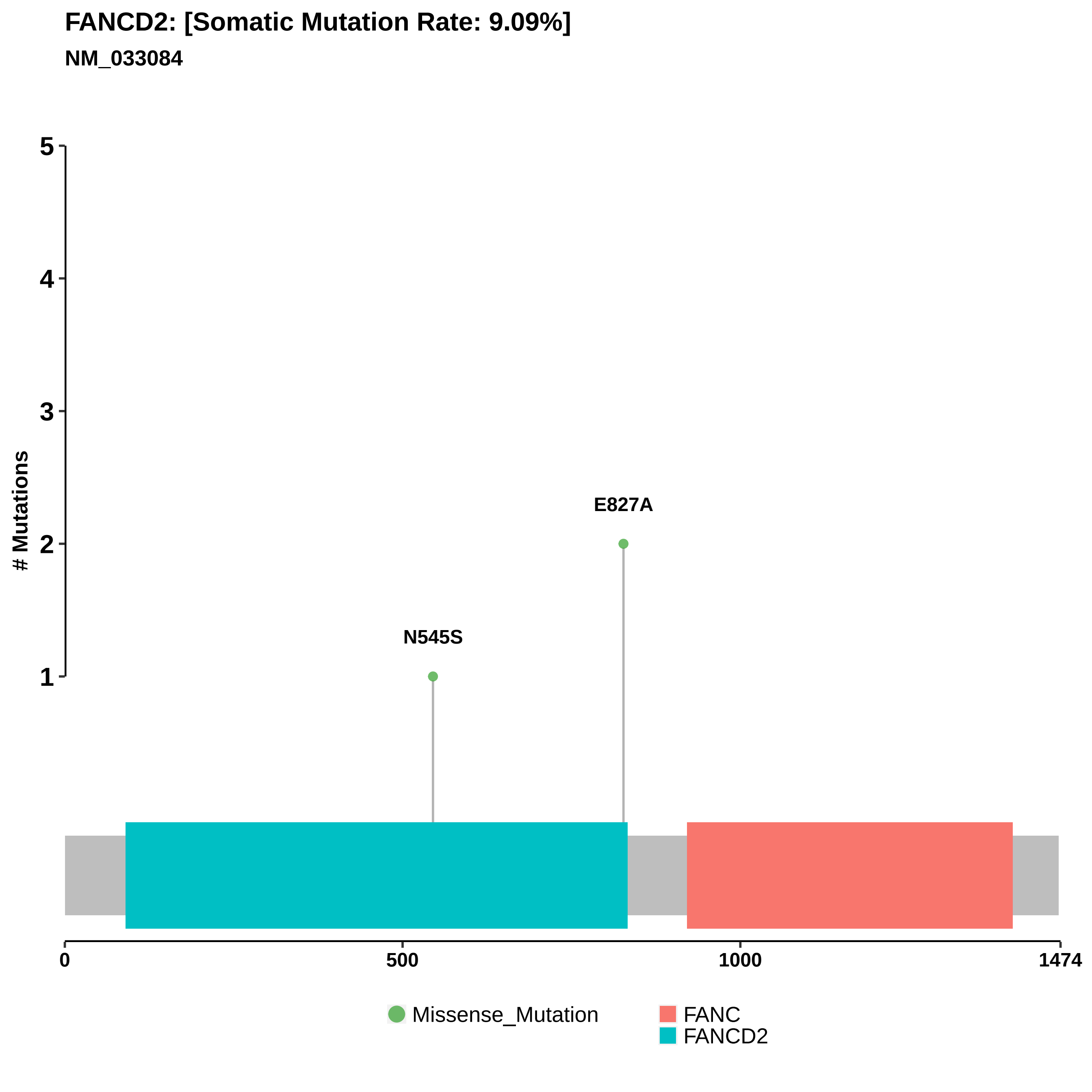

FANCD2: [Somatic Mutation Rate: 9.09%]
NM_033084
Mutations
5
4
3
2
1
E827A
N545S
0
500
1000
1474
Missense_Mutation
FANC
FANCD2

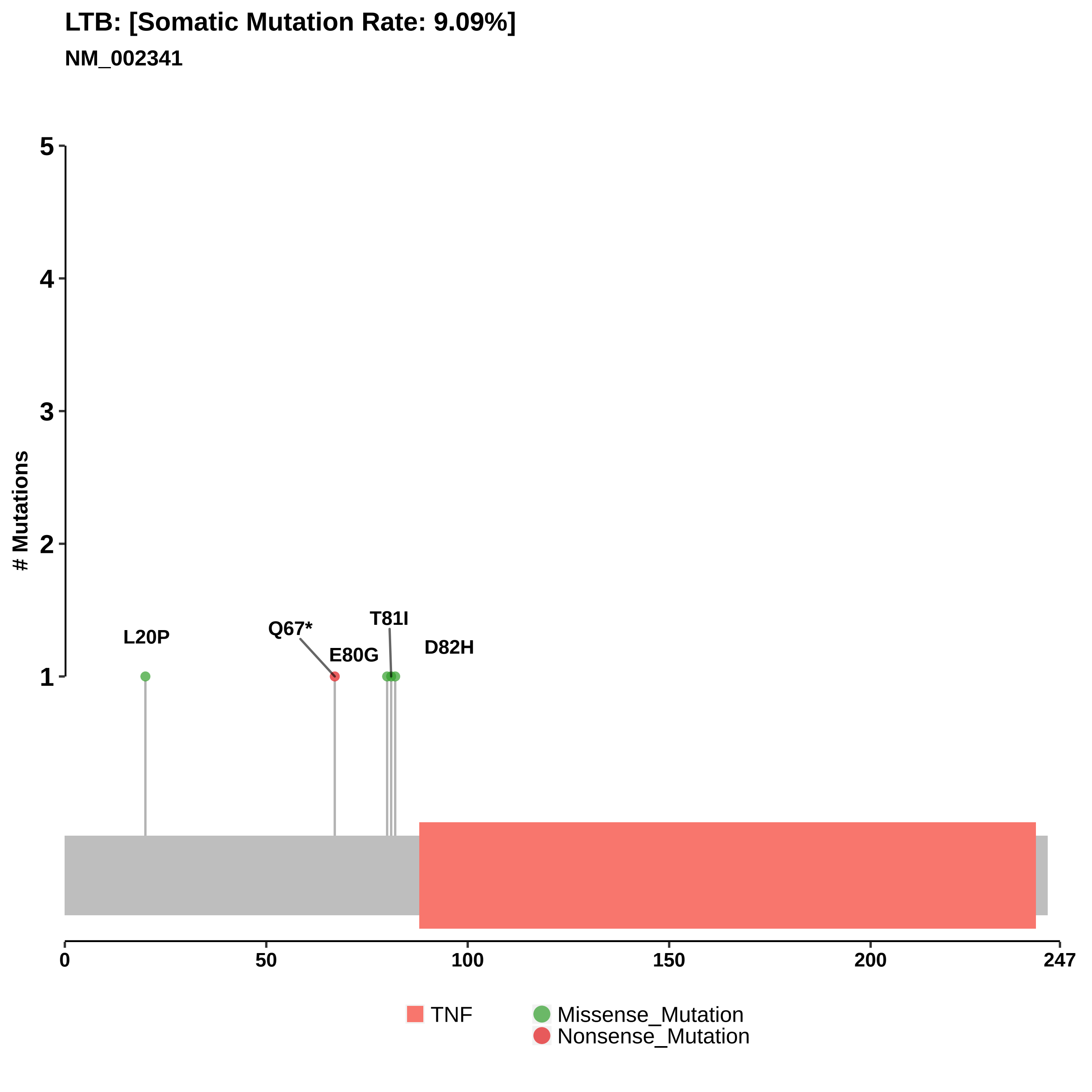
LTB: [Somatic Mutation Rate: 9.09%]
NM_002341
Mutations
5
4
3
2
1
L20P
Q67*
E80G
T81I
D82H
0
50
100
150
200
247
TNF
Missense_Mutation
Nonsense_Mutation

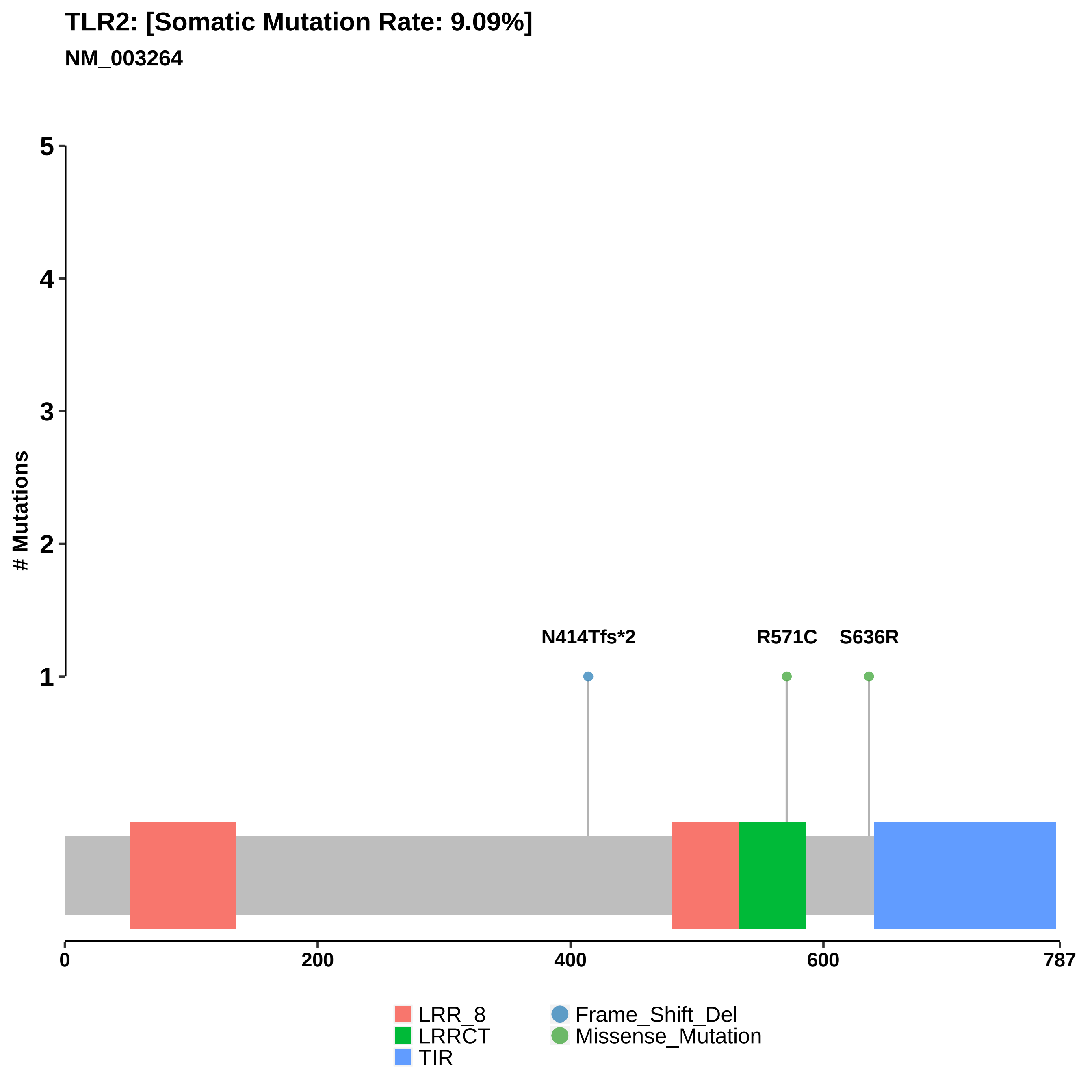
TLR2: [Somatic Mutation Rate: 9.09%]
NM_003264
Mutations
5
4
3
2
1
N414Tfs*2
R571C
S636R
0
200
400
600
787
LRR_8
LRRCT
TIR
Frame_Shift_Del
Missense_Mutation

TRADD: [Somatic Mutation Rate: 9.09%]

NM_003789

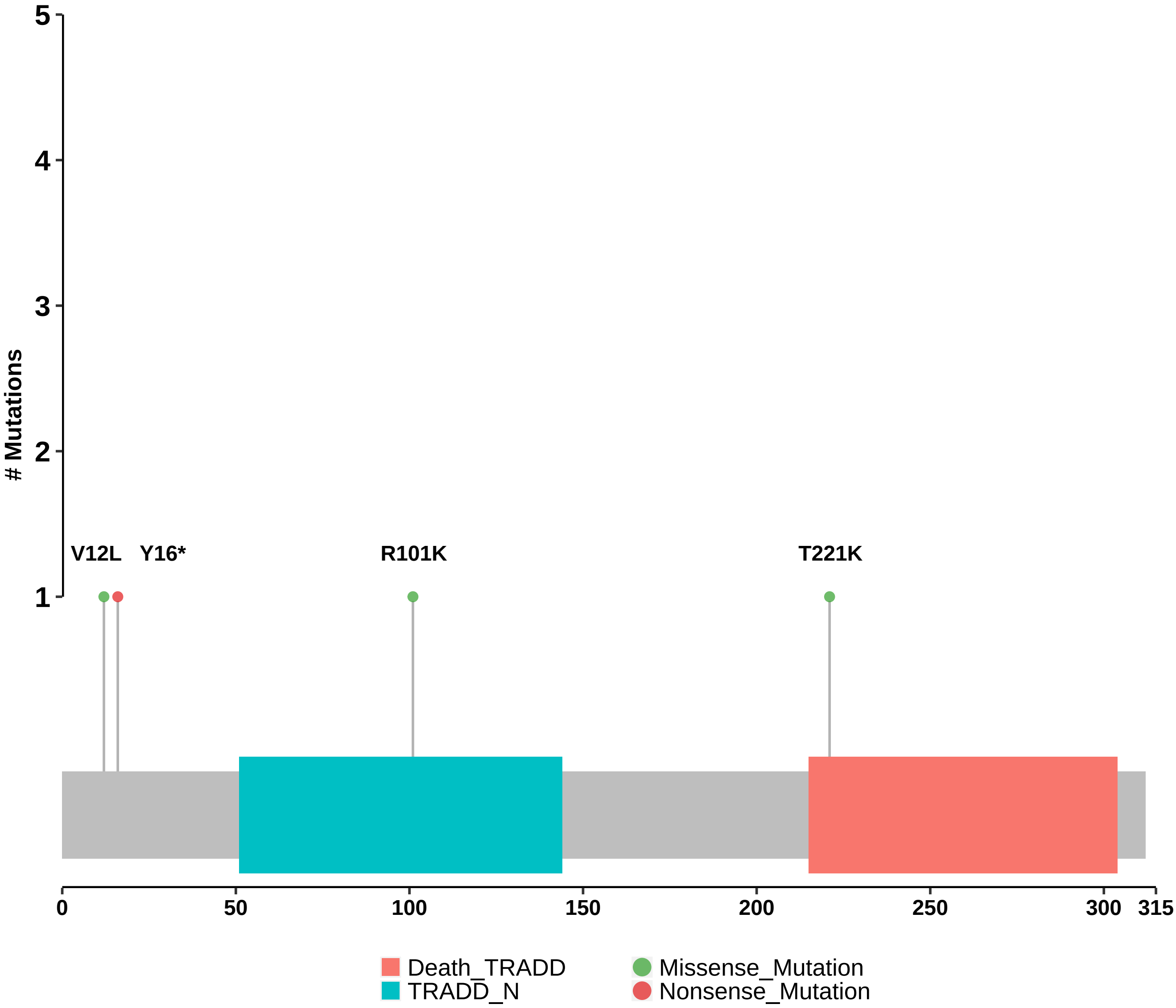

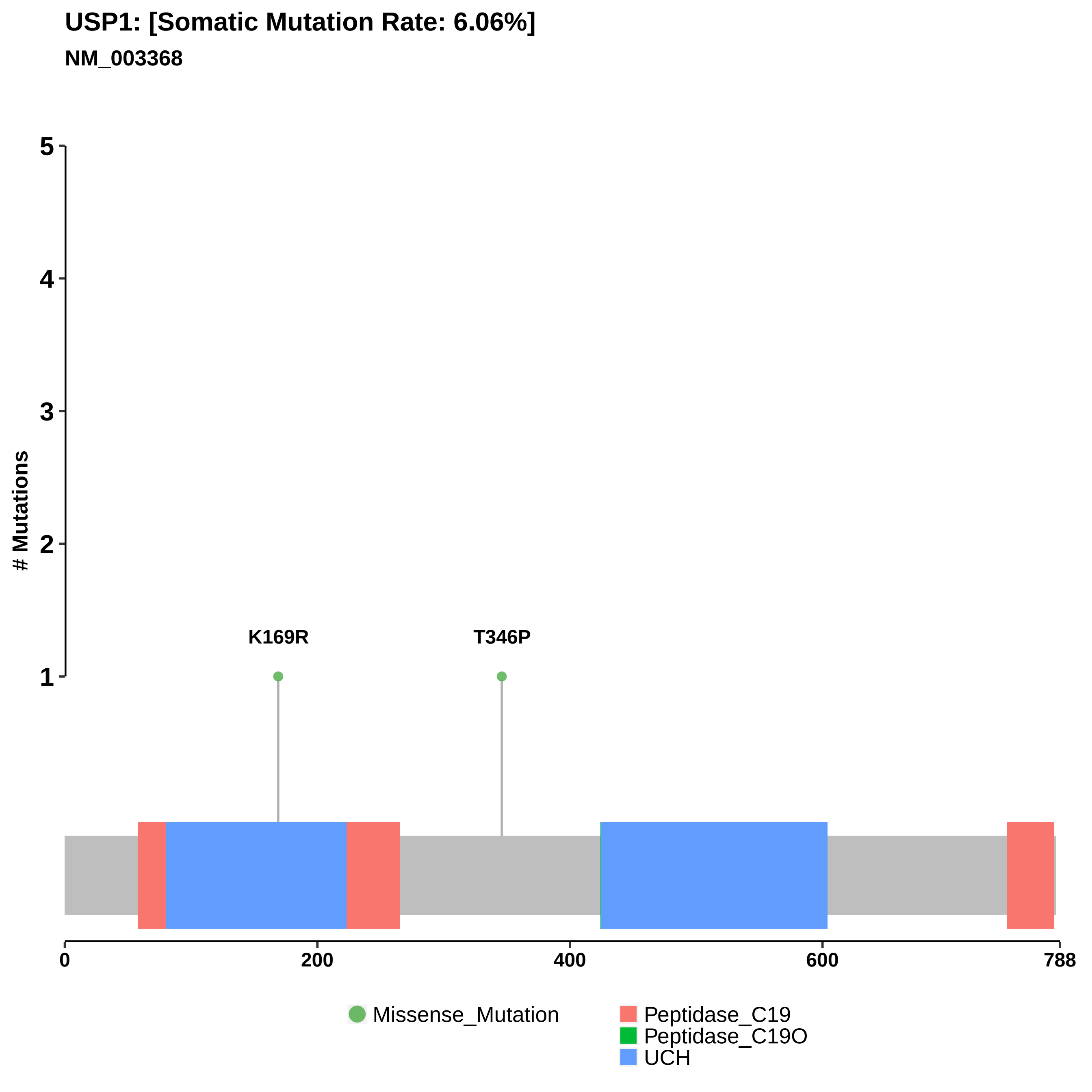
USP1: [Somatic Mutation Rate: 6.06%]
NM_003368
Mutations
5
4
3
2
1
K169R
T346P
0
200
400
600
788
Missense_Mutation
Peptidase_C19
Peptidase_C19O
UCH

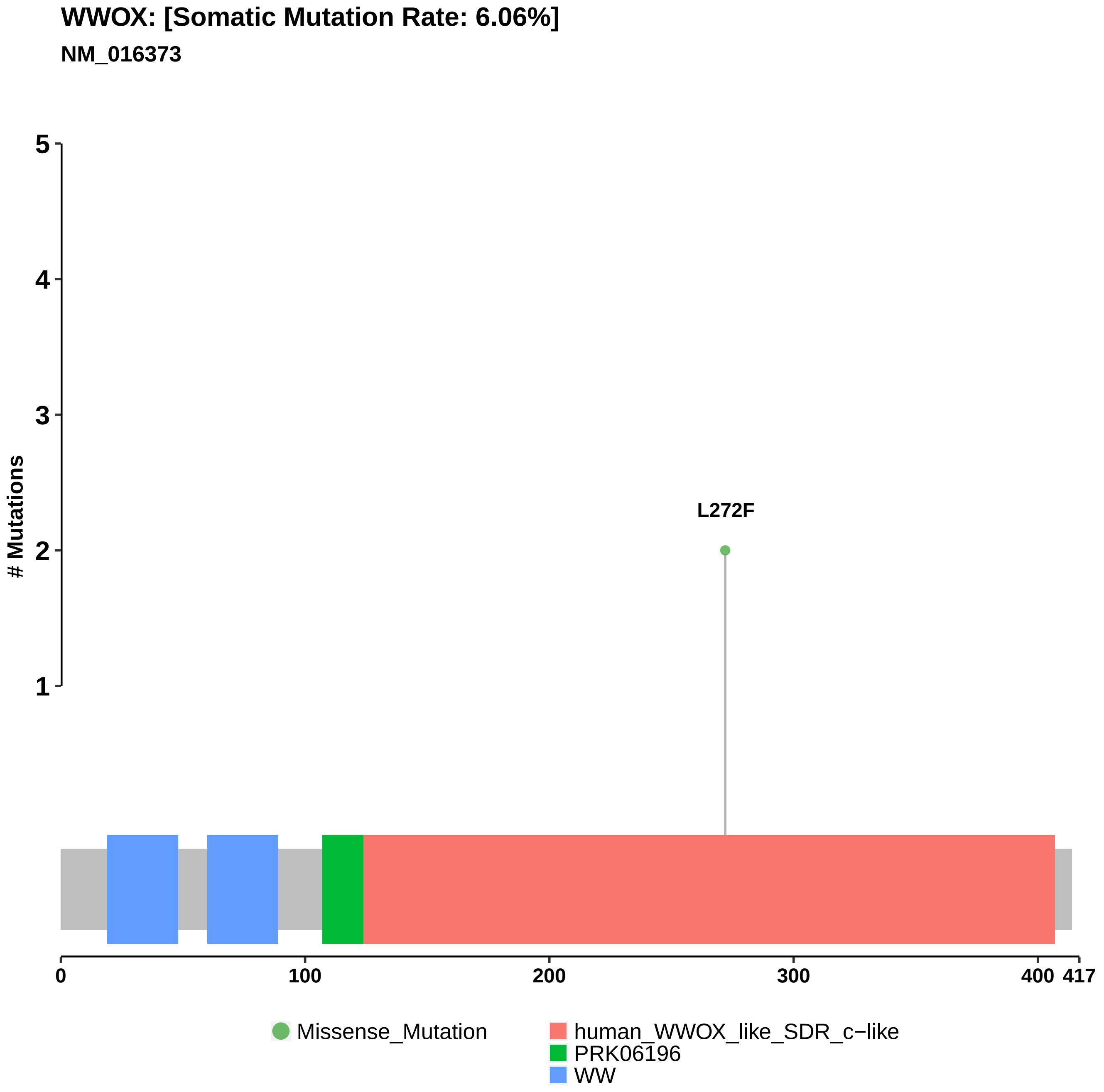
WWOX: [Somatic Mutation Rate: 6.06%]
NM_016373
Mutations
5
4
3
2
1
L272F
0
100
200
300
400
417
Missense_Mutation
human_WWOX_like_SDR_c−like
PRK06196
WW

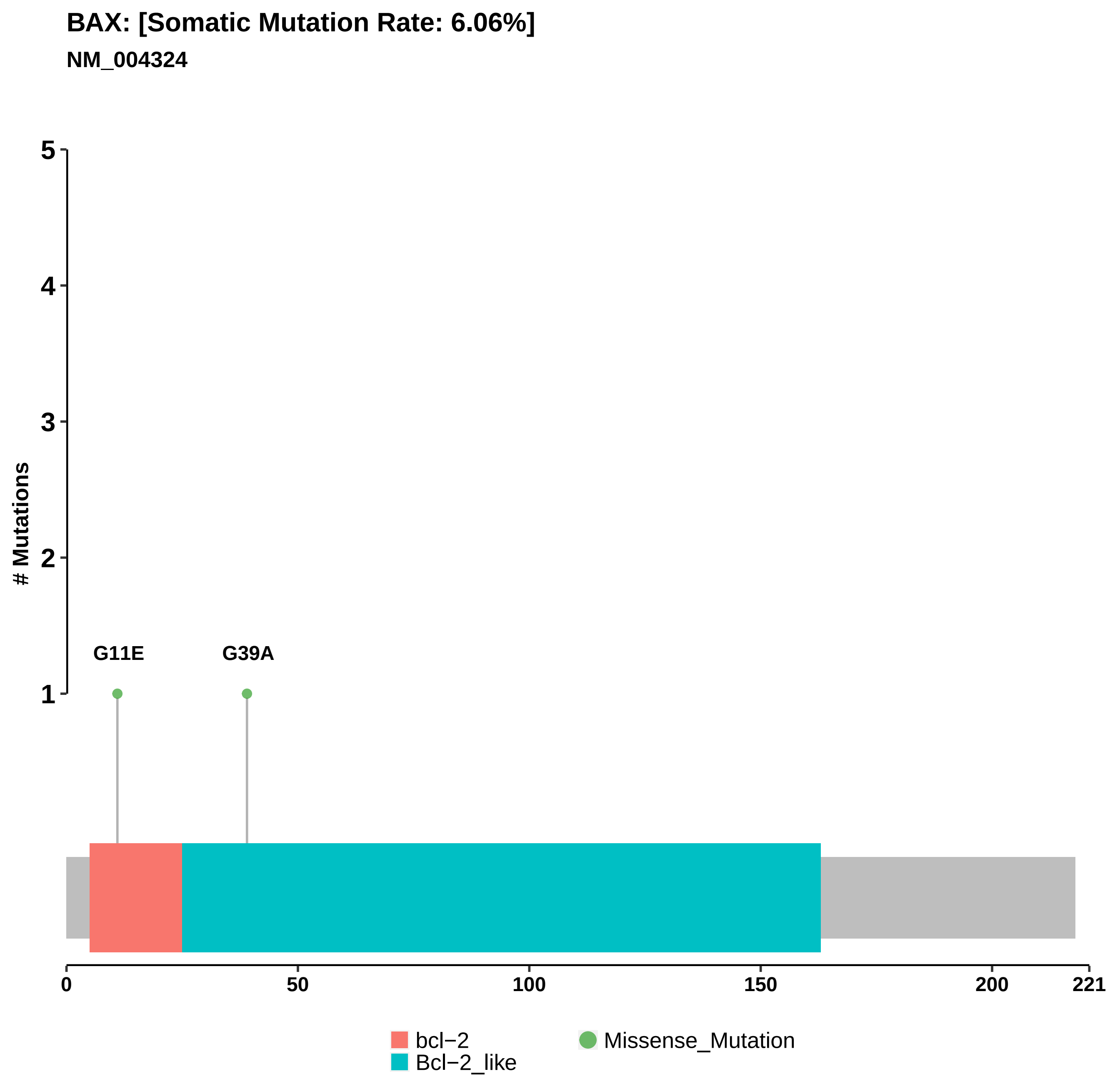
BAX: [Somatic Mutation Rate: 6.06%]
NM_004324
Mutations
5
4
3
2
1
G11E
G39A
0
50
100
150
200
221
bcl−2
Bcl−2_like
Missense_Mutation

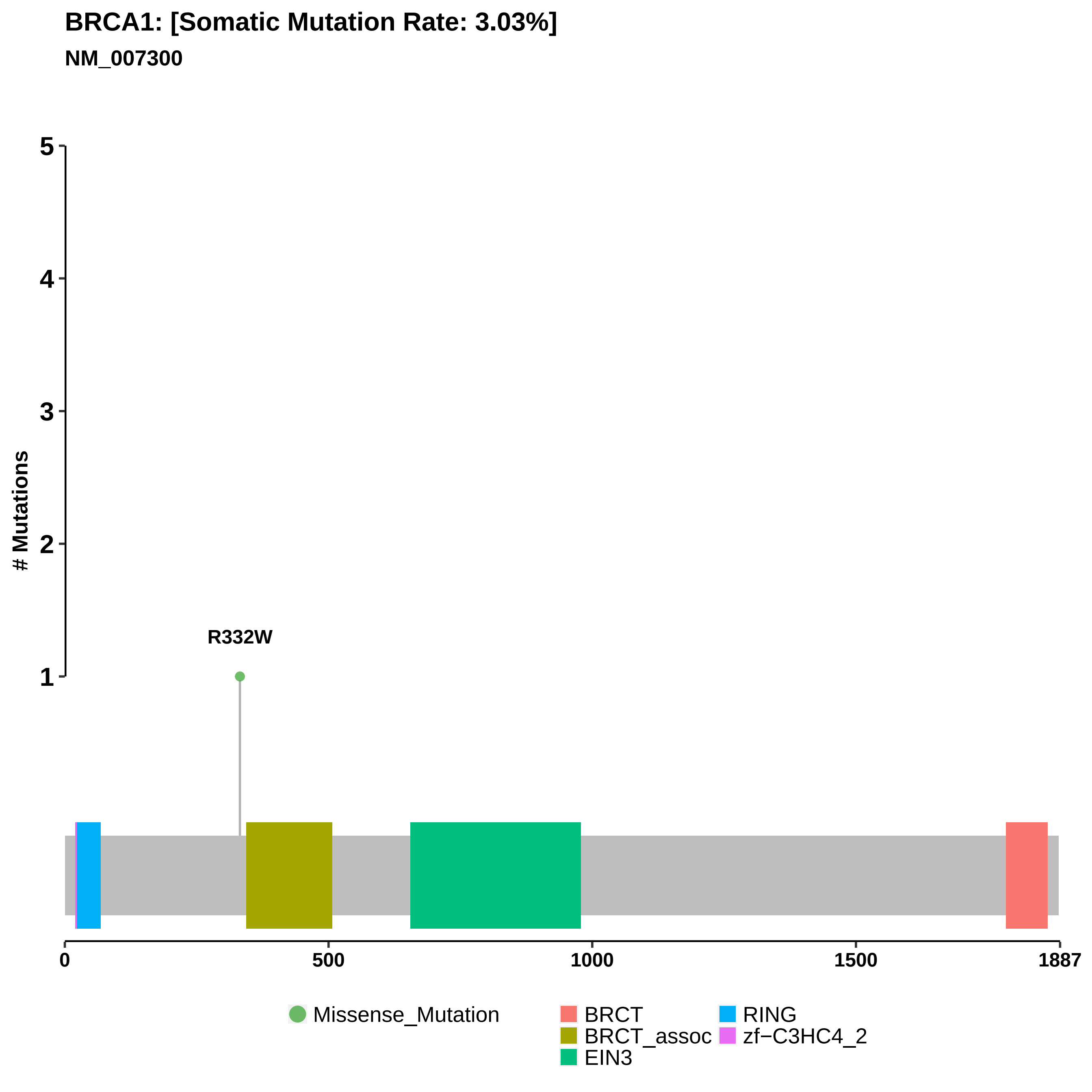
BRCA1: [Somatic Mutation Rate: 3.03%]
NM_007300
Mutations
5
4
3
2
1
R332W
0
500
1000
1500
1887
Missense_Mutation
BRCT
BRCT_assoc
EIN3
RING
zf−C3HC4_2

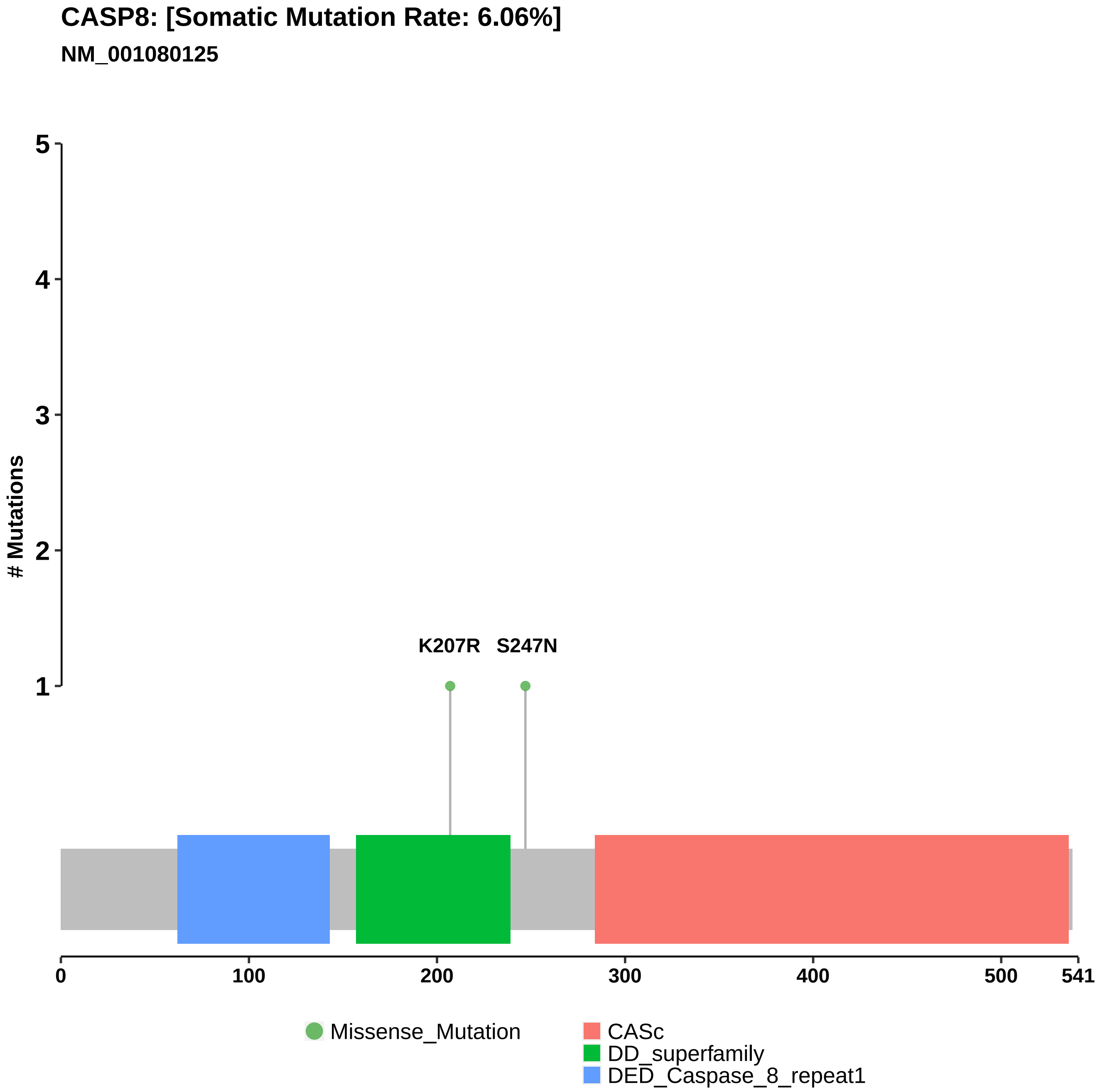
CASP8: [Somatic Mutation Rate: 6.06%]
NM_001080125
Mutations
5
4
3
2
1
K207R
S247N
0
100
200
300
400
500
541
Missense_Mutation
CASc
DD_superfamily
DED_Caspase_8_repeat1

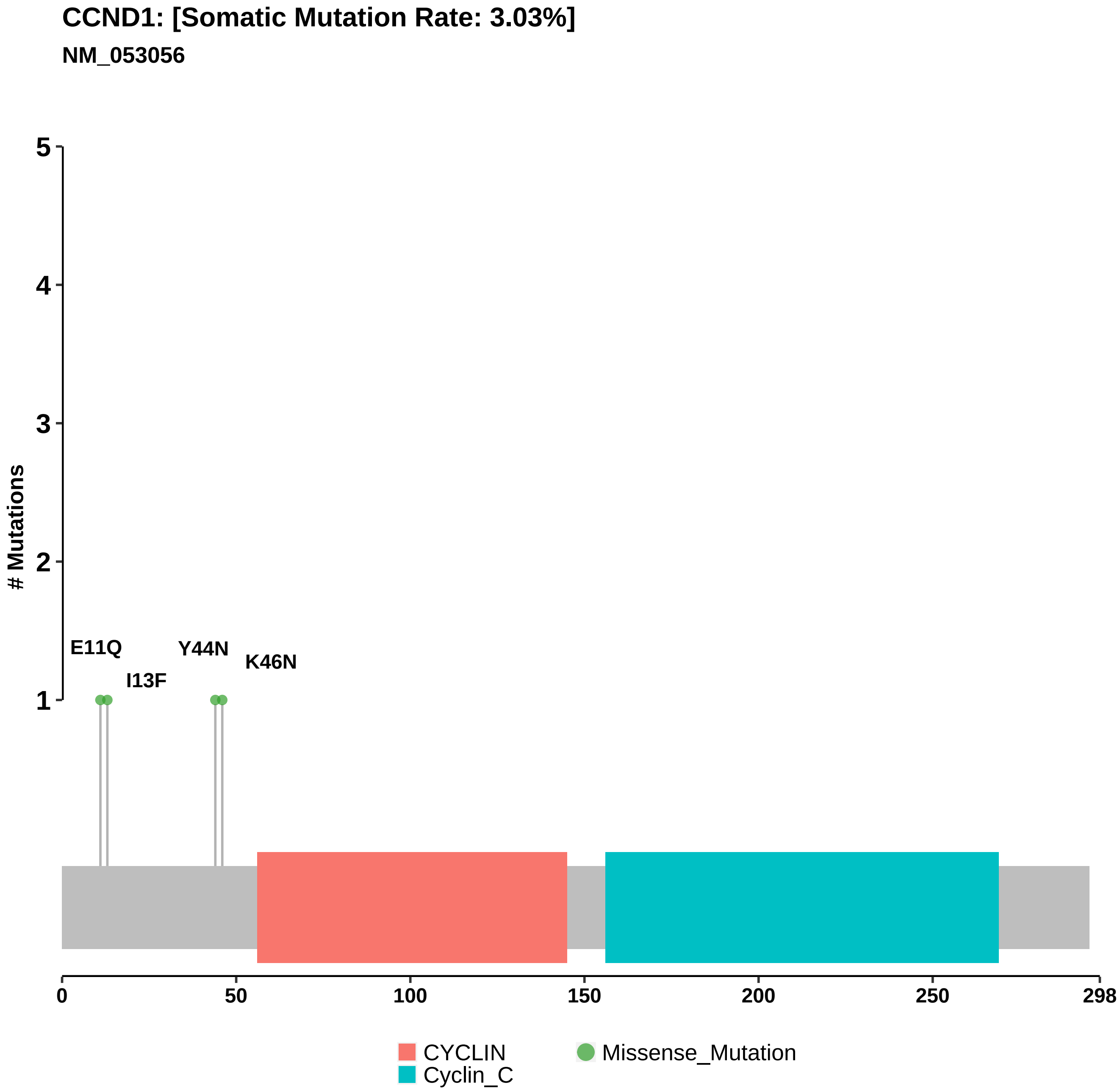
CCND1: [Somatic Mutation Rate: 3.03%]
NM_053056
Mutations
5
4
3
2
1
E11Q
I13F
Y44N
K46N
0
50
100
150
200
250
298
CYCLIN
Cyclin_C
Missense_Mutation

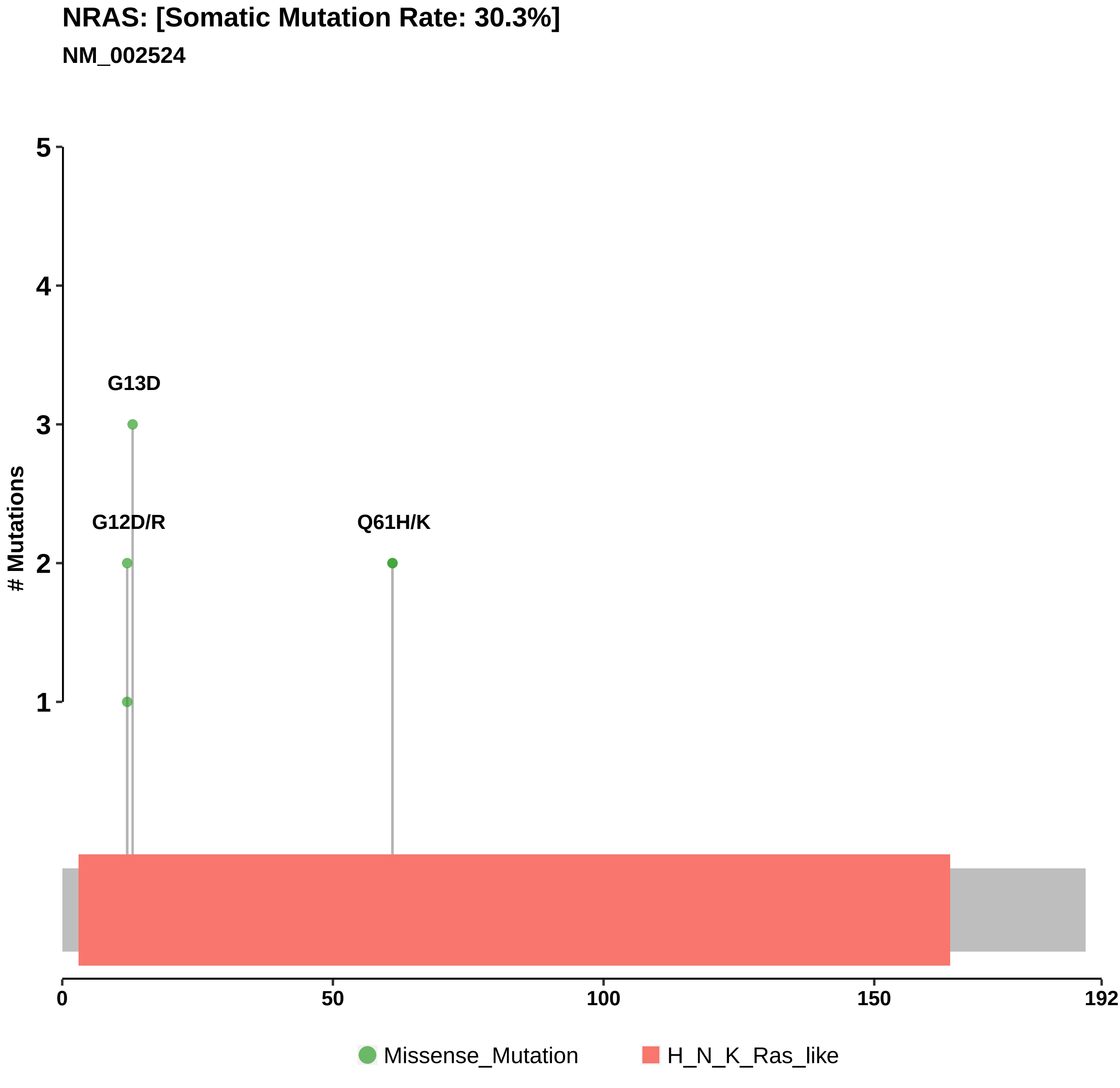

NRAS: [Somatic Mutation Rate: 30.3%]
NM_002524
Mutations
5
4
3
2
1
G13D
G12D/R
Q61H/K
0
50
100
150
192
Missense_Mutation
H_N_K_Ras_like

CDKN1A: [Somatic Mutation Rate: 3.03%]

NM_000389

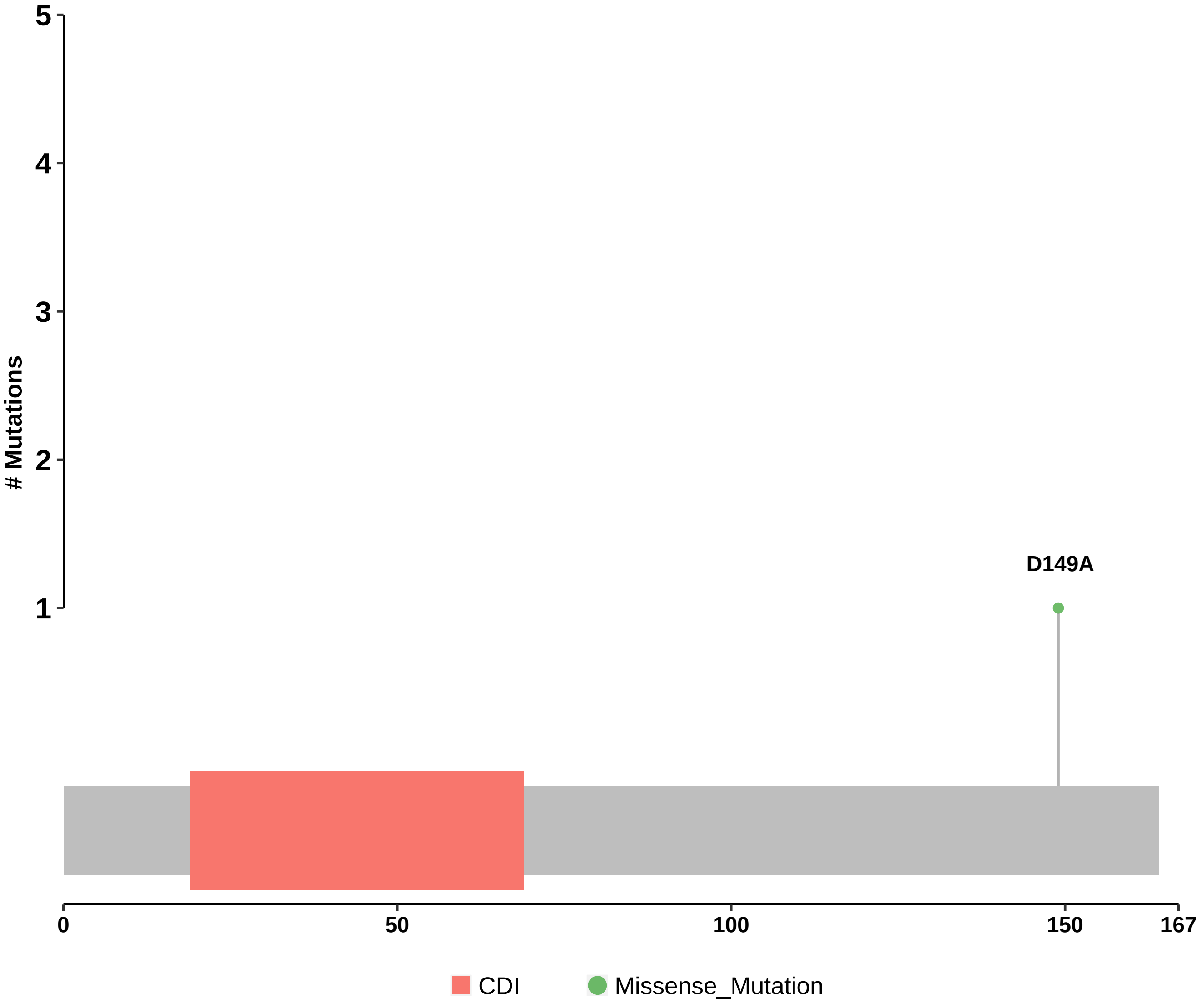

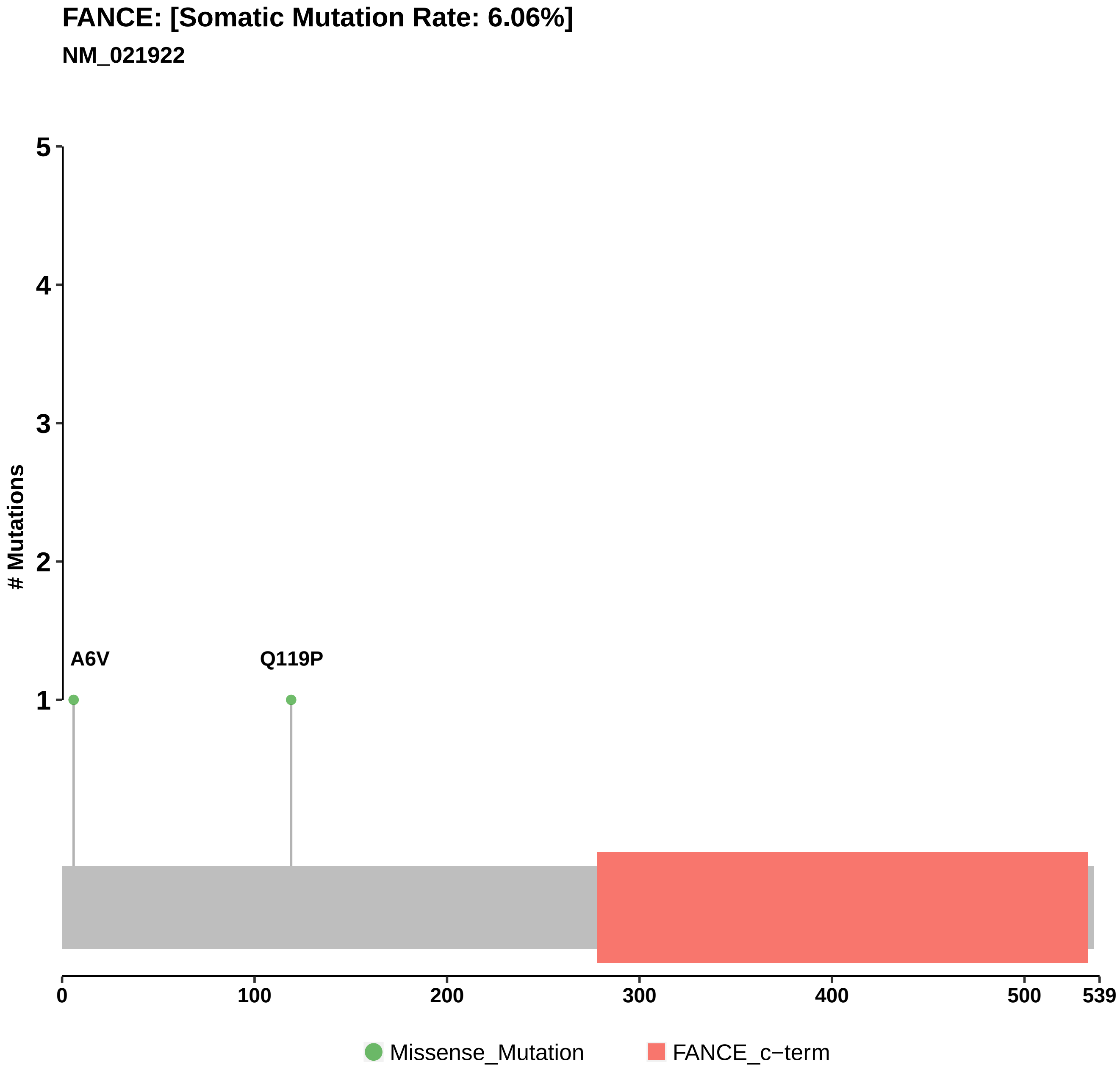

FANCE: [Somatic Mutation Rate: 6.06%]
NM_021922
Mutations
5
4
3
2
1
A6V
Q119P
0
100
200
300
400
500
539
Missense_Mutation
FANCE_c−term

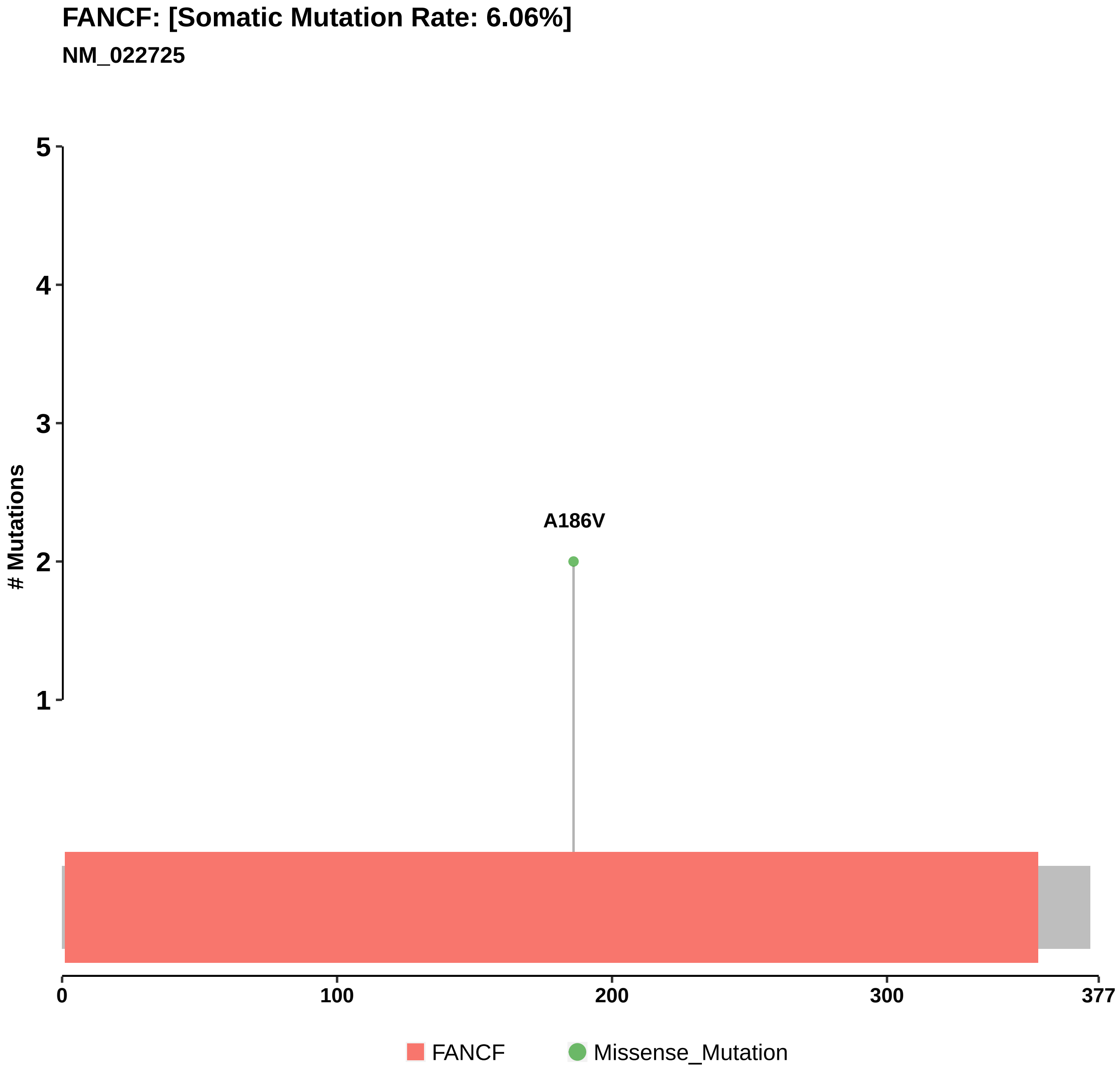
FANCF: [Somatic Mutation Rate: 6.06%]
NM_022725
Mutations
5
4
3
2
1
A186V
0
100
200
300
377
FANCF
Missense_Mutation

FANCG: [Somatic Mutation Rate: 6.06%]
NM_004629

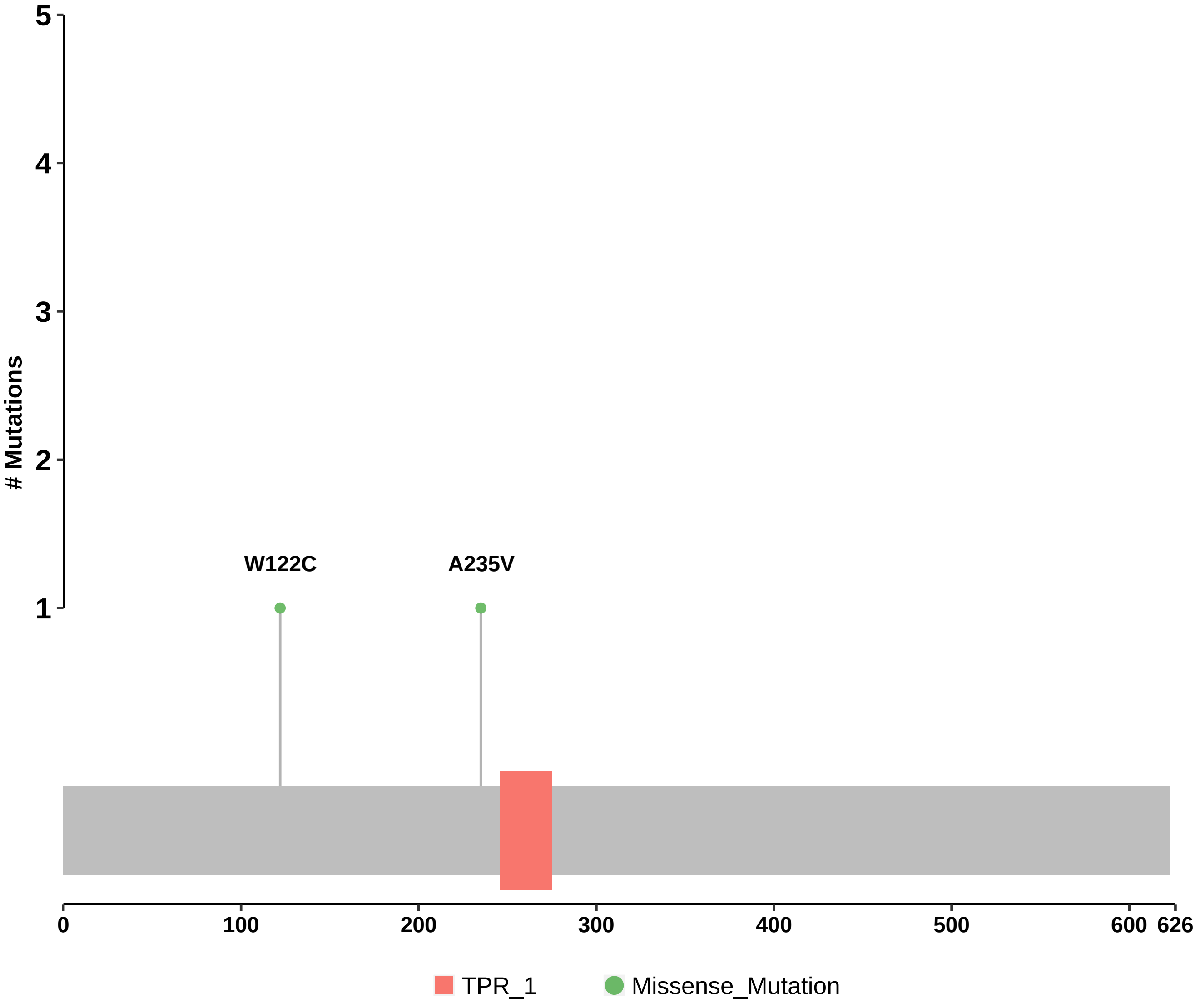

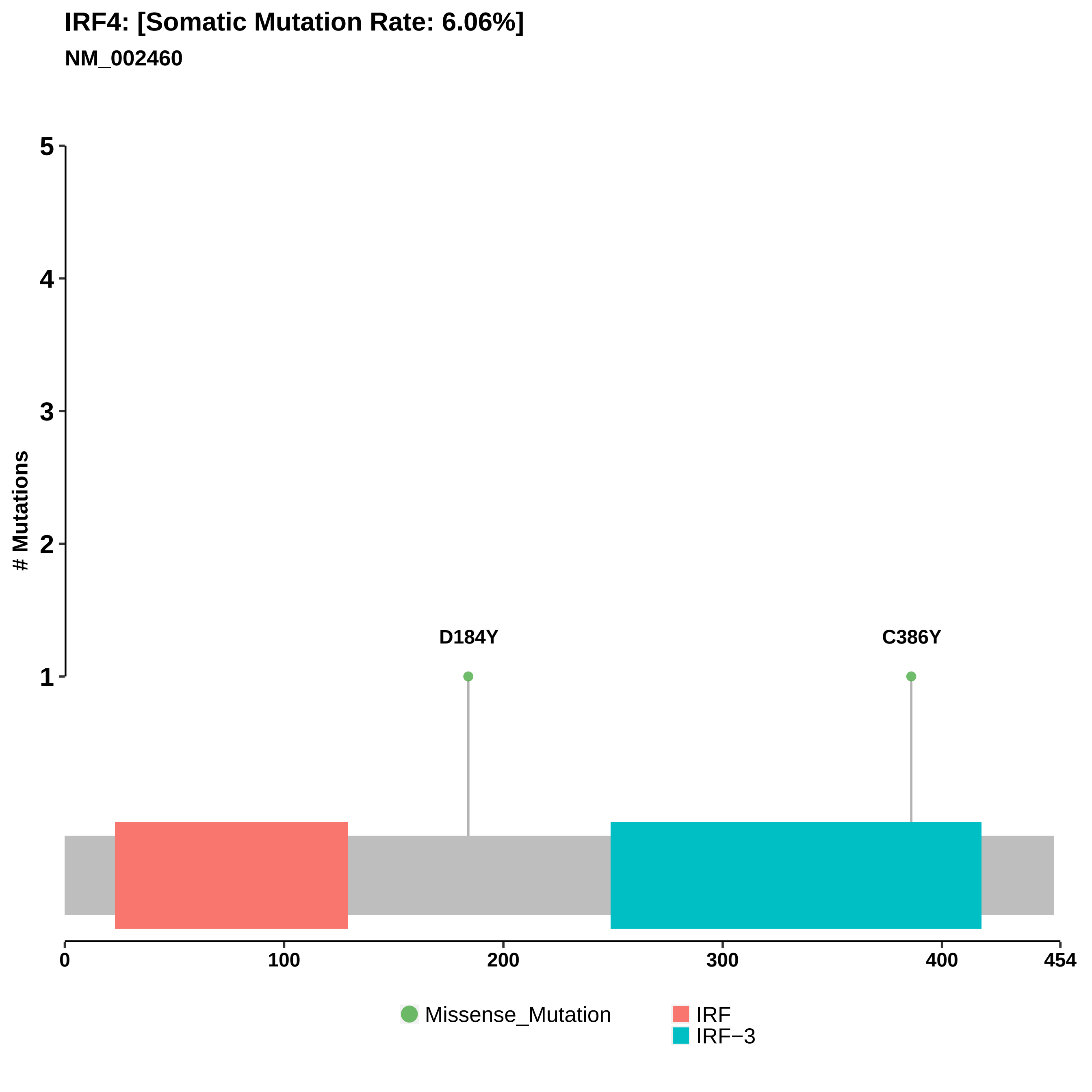

IRF4: [Somatic Mutation Rate: 6.06%]
NM_002460
Mutations
5
4
3
2
1
D184Y
C386Y
0
100
200
300
400
454
Missense_Mutation
IRF
IRF−3

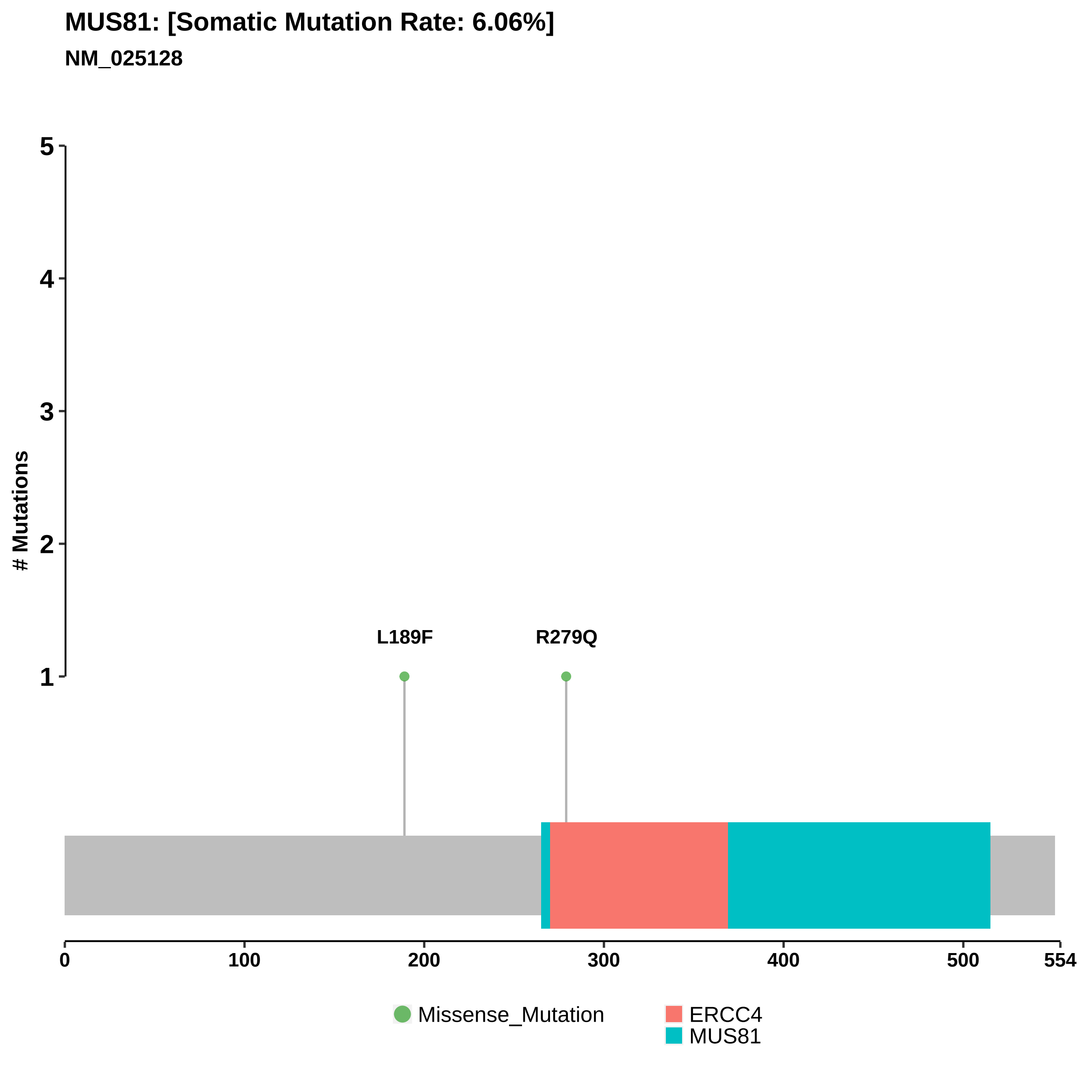
MUS81: [Somatic Mutation Rate: 6.06%]
NM_025128
Mutations
5
4
3
2
1
L189F
R279Q
0
100
200
300
400
500
554
Missense_Mutation
ERCC4
MUS81

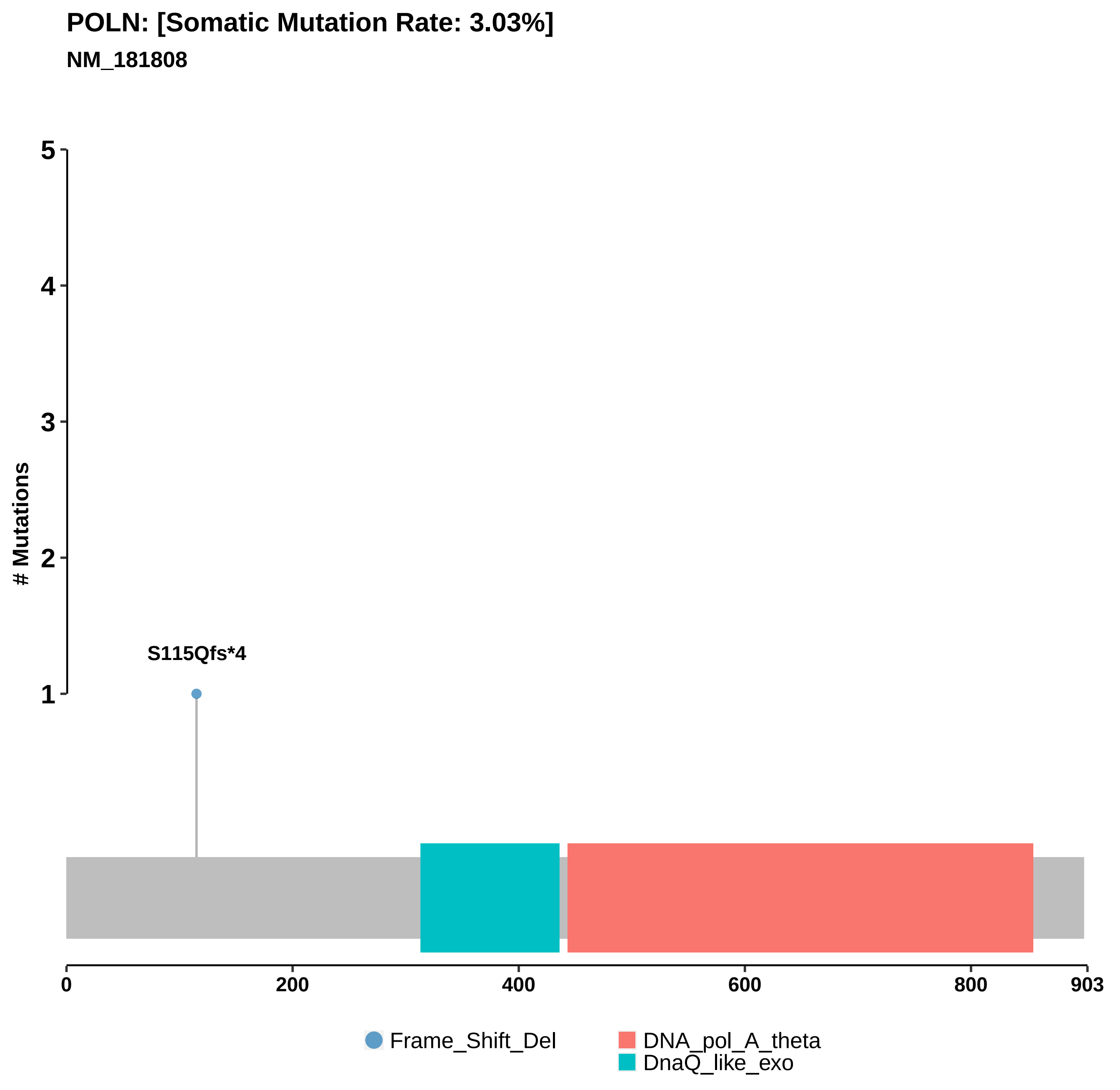

POLN: [Somatic Mutation Rate: 3.03%]
NM_181808
Mutations
5
4
3
2
1
S115Qfs*4
0
200
400
600
800
903
Frame_Shift_Del
DNA_pol_A_theta
DnaQ_like_exo

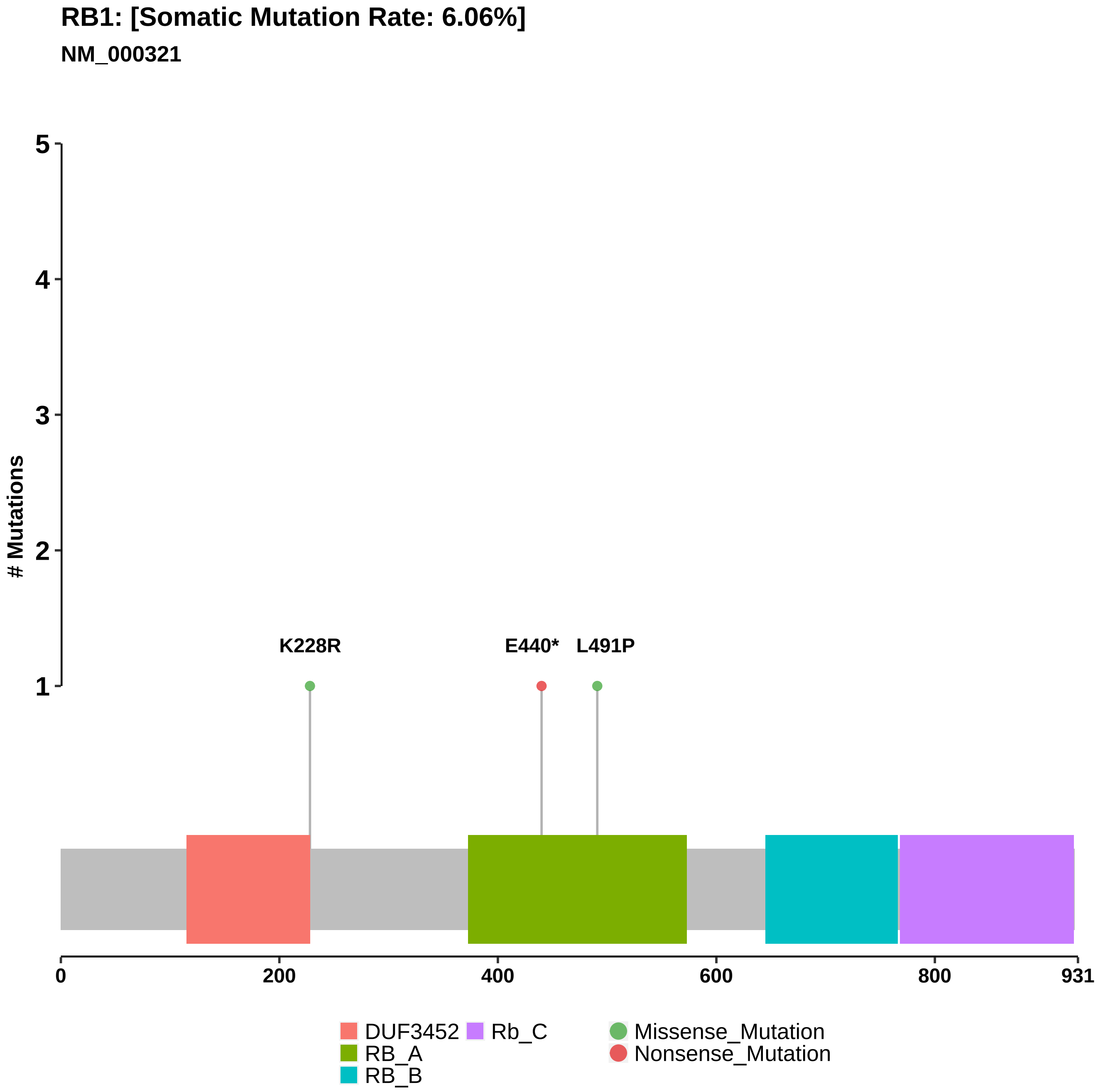
RB1: [Somatic Mutation Rate: 6.06%]
NM_000321
Mutations
5
4
3
2
1
K228R
E440*
L491P
0
200
400
600
800
931
DUF3452
RB_A
RB_B
Rb_C
Missense_Mutation
Nonsense_Mutation

RELA: [Somatic Mutation Rate: 6.06%]
NM_021975

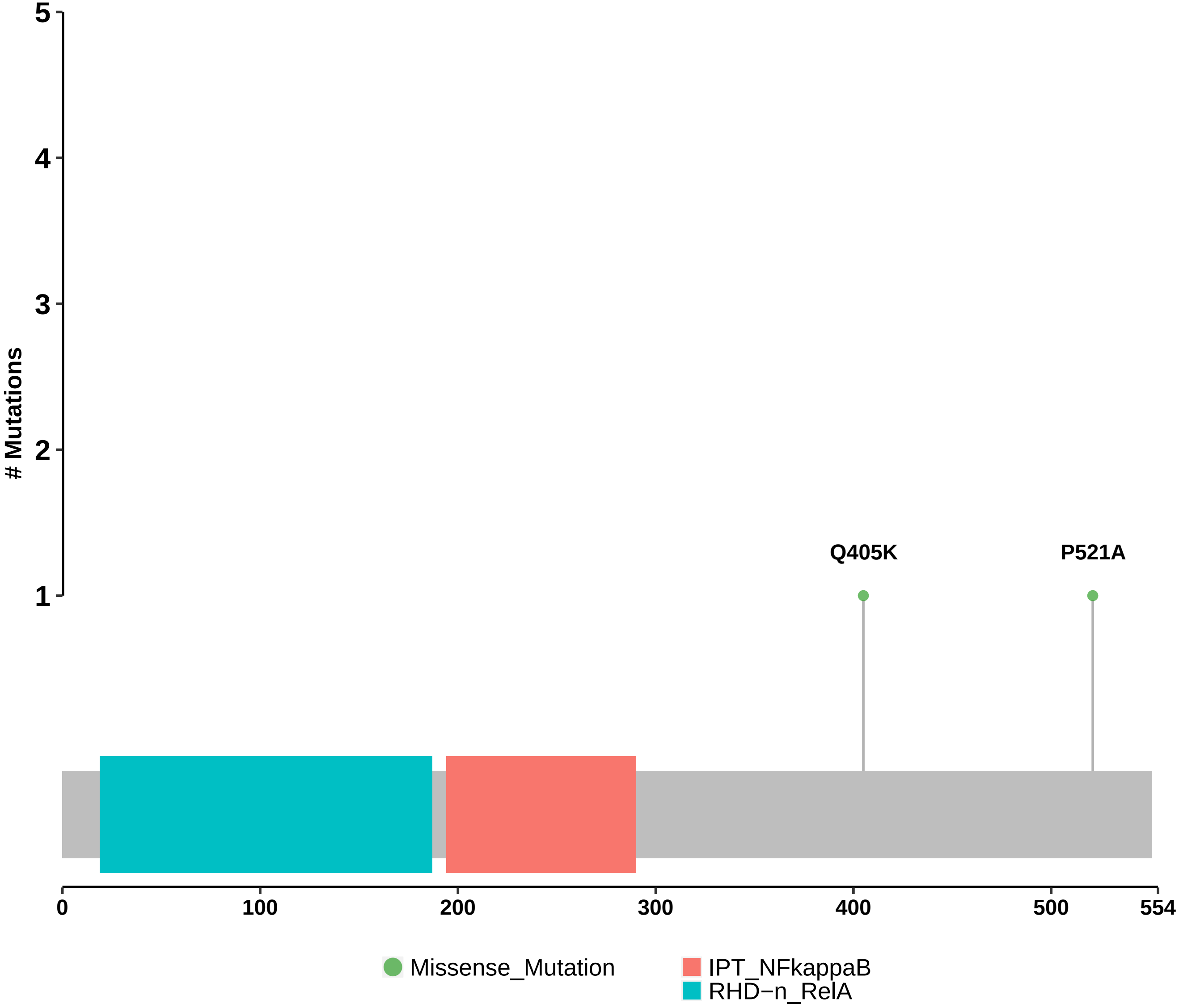

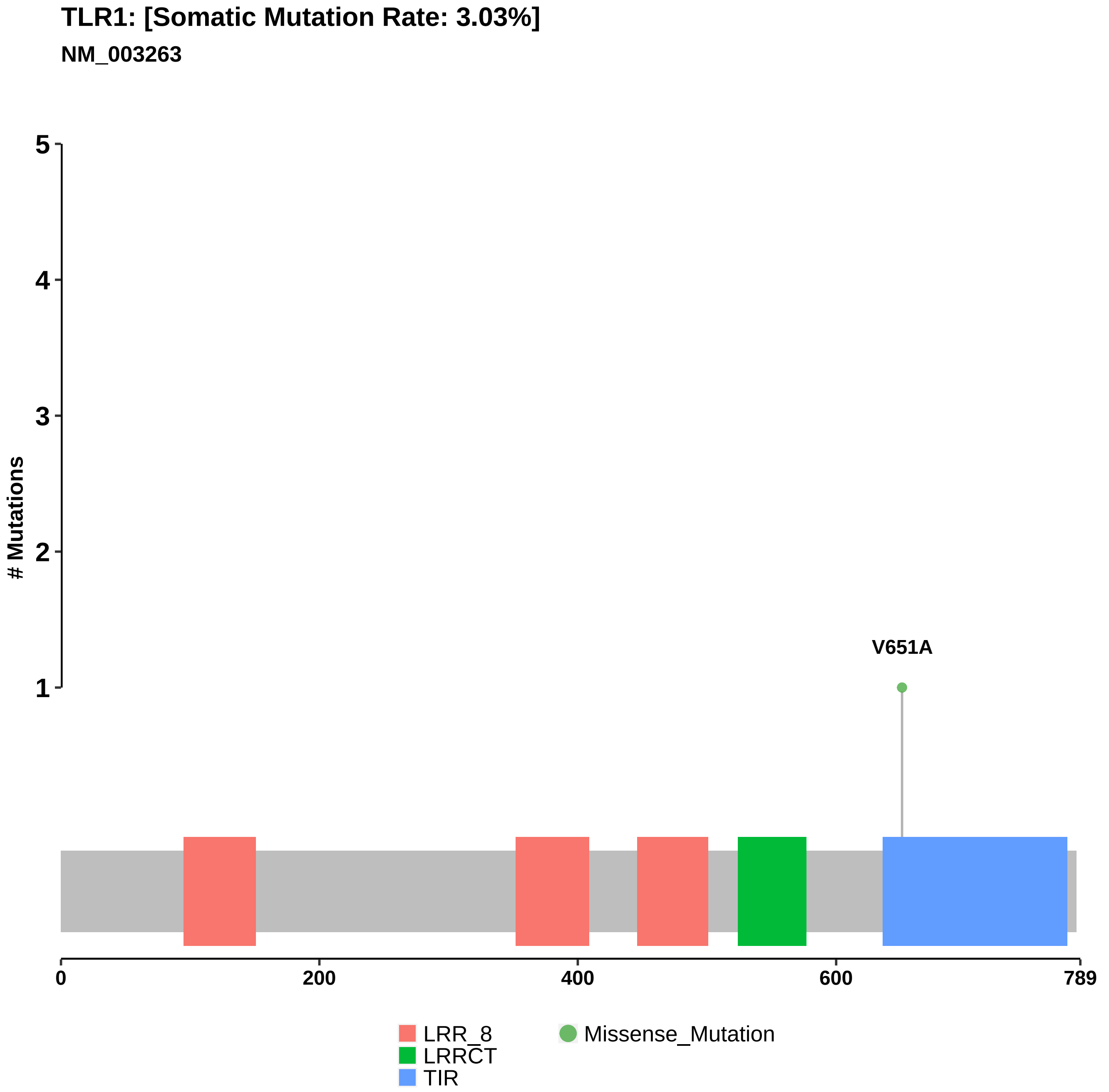
TLR1: [Somatic Mutation Rate: 3.03%]
NM_003263
5
4
3
2
1
Mutations
V651A
0
200
400
600
789
LRR_8
LRRCT
TIR
Missense_Mutation

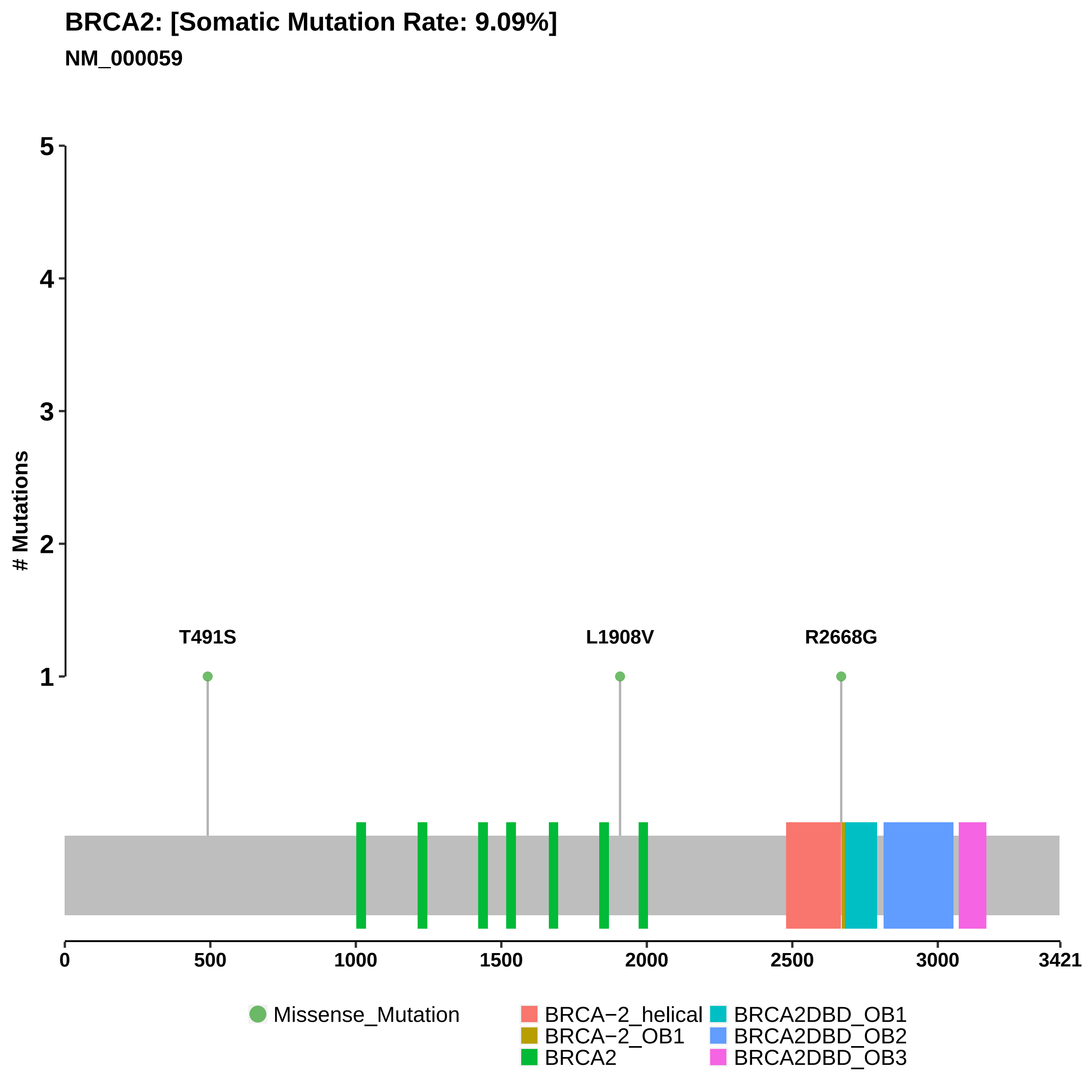

BRCA2: [Somatic Mutation Rate: 9.09%]
NM_000059
Mutations
5
4
3
2
1
T491S
L1908V
R2668G
0
500
1000
1500
2000
2500
3000
3421
Missense_Mutation
BRCA−2_helical
BRCA−2_OB1
BRCA2
BRCA2DBD_OB1
BRCA2DBD_OB2
BRCA2DBD_OB3

TLR6: [Somatic Mutation Rate: 6.06%]

NM_006068

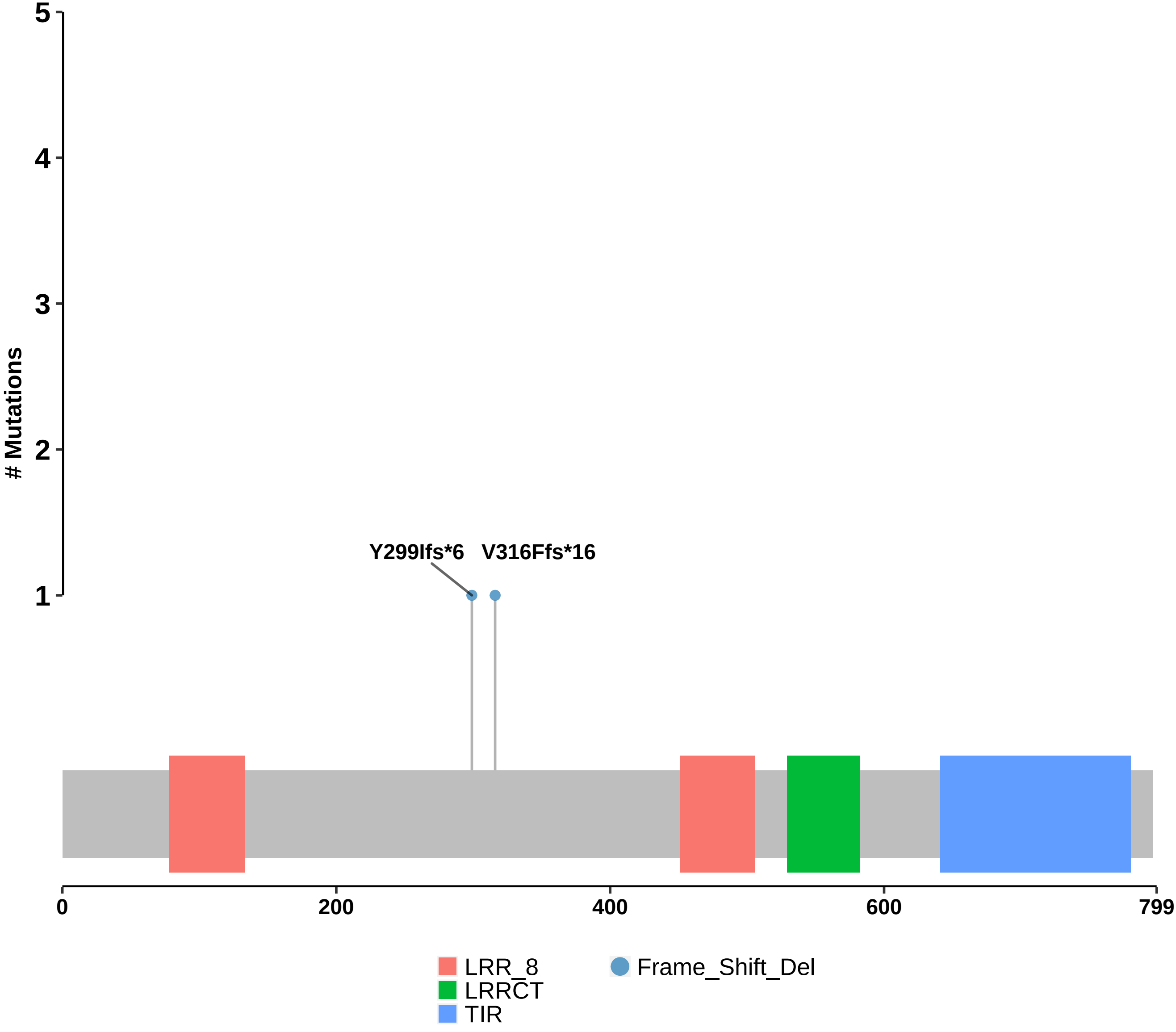

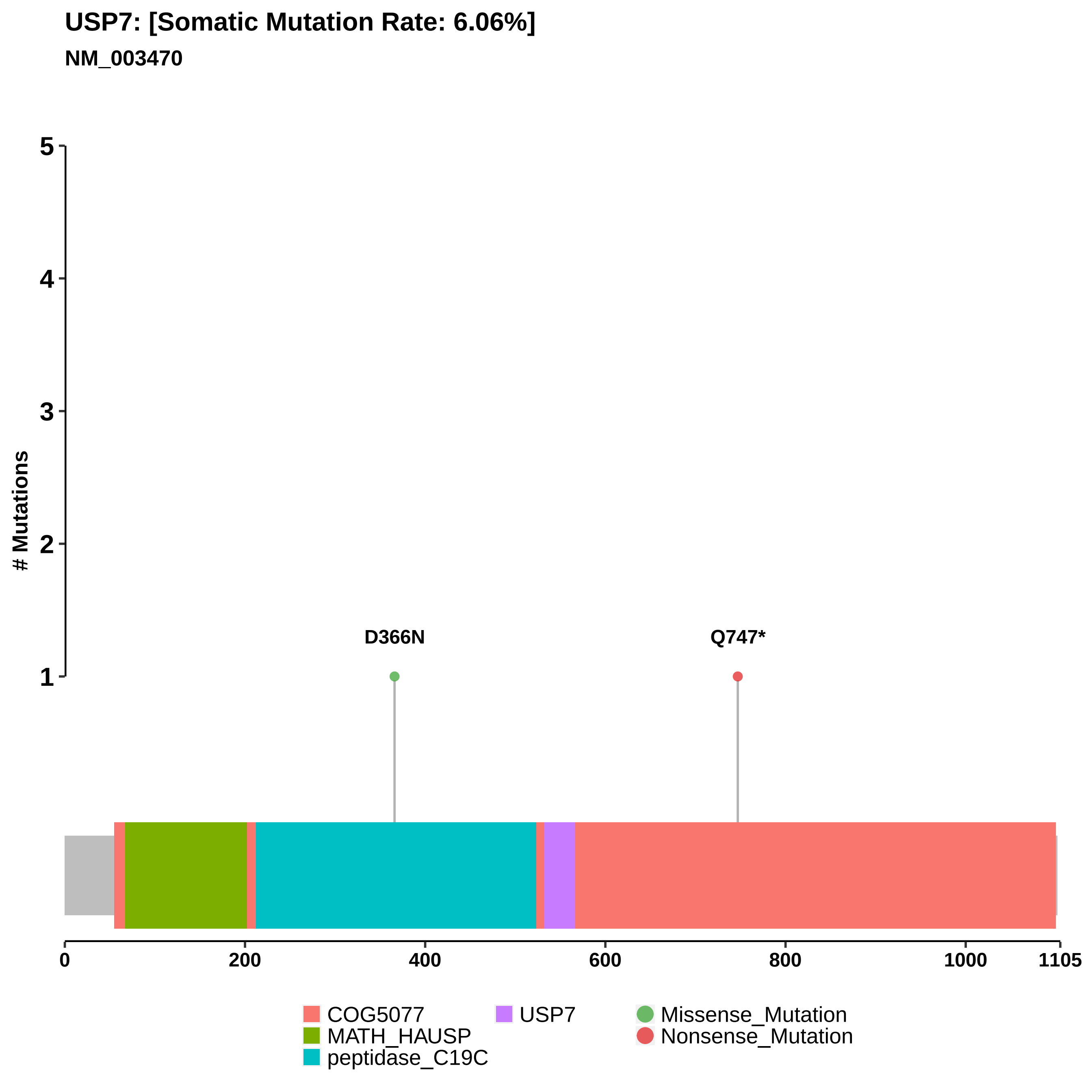
USP7: [Somatic Mutation Rate: 6.06%]
NM_003470
Mutations
5
4
3
2
1
D366N
Q747*
0
200
400
600
800
1000
1105
COG5077
MATH_HAUSP
peptidase_C19C
USP7
Missense_Mutation
Nonsense_Mutation

BAD: [Somatic Mutation Rate: 3.03%]

NM_004322

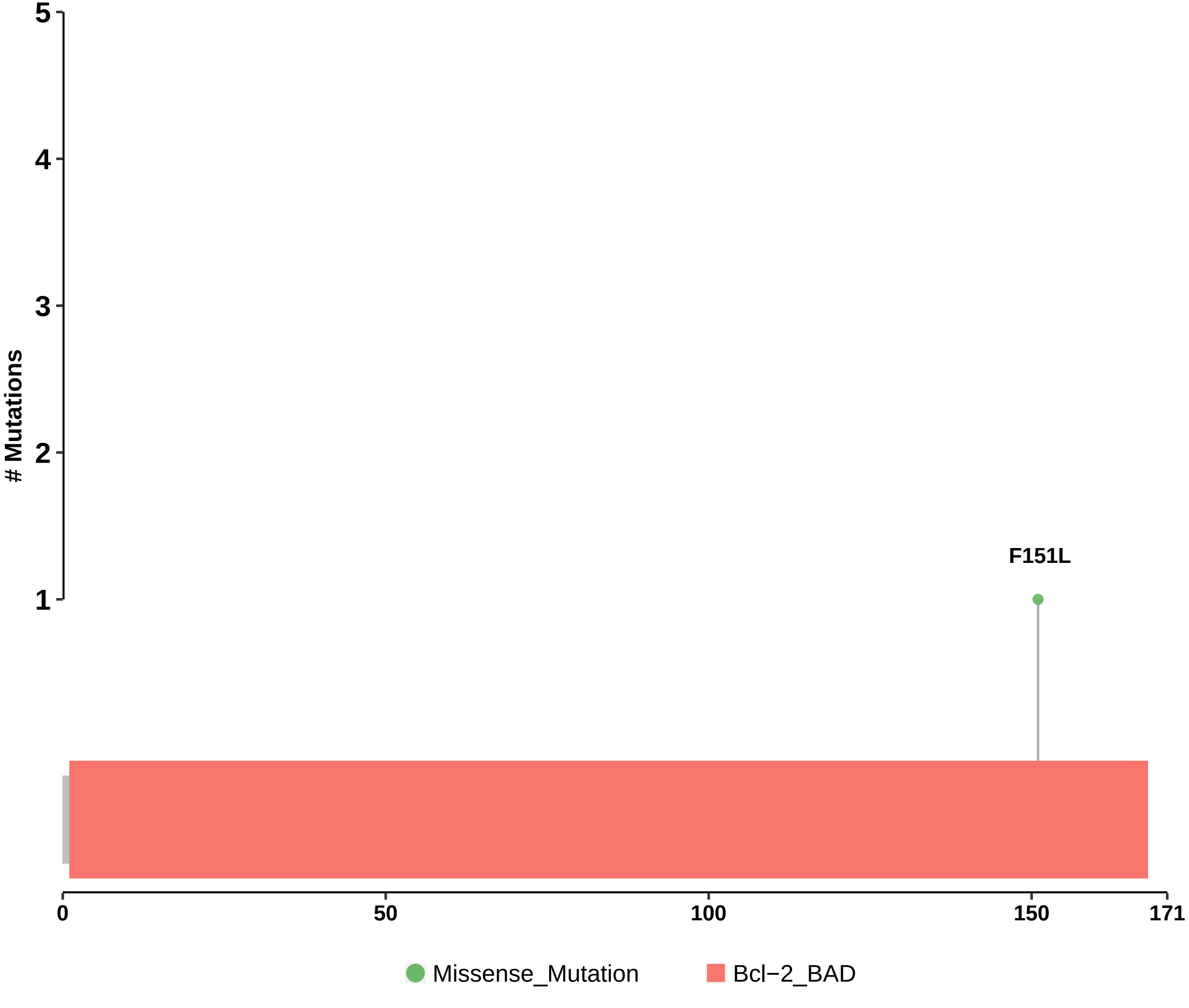

BAK1: [Somatic Mutation Rate: 3.03%]

NM_001188

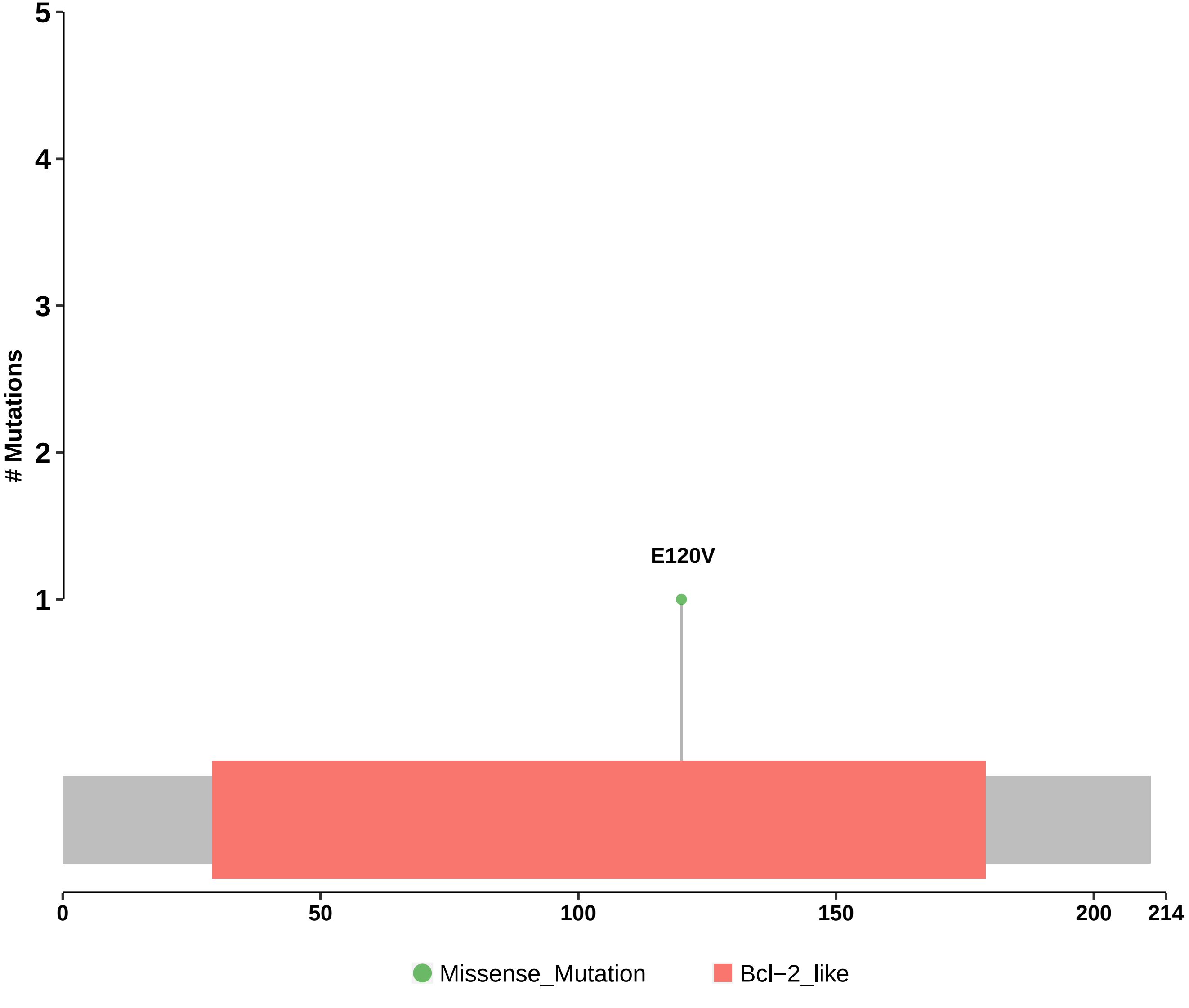

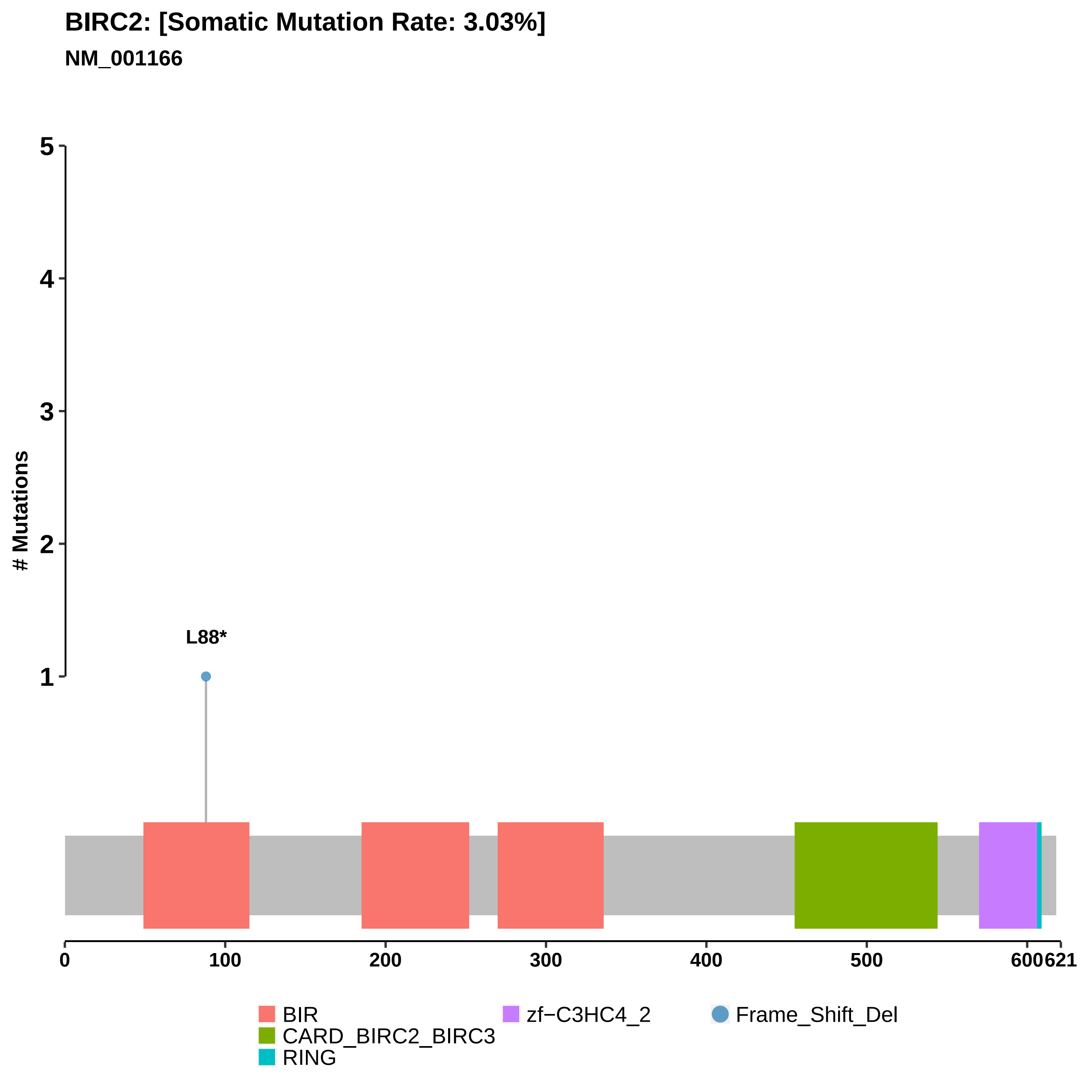
BIRC2: [Somatic Mutation Rate: 3.03%]
NM_001166
5
4
3
2
1
Mutations
L88*
0
100
200
300
400
500
600
621
BIR
CARD_BIRC2_BIRC3
RING
zf−C3HC4_2
Frame_Shift_Del

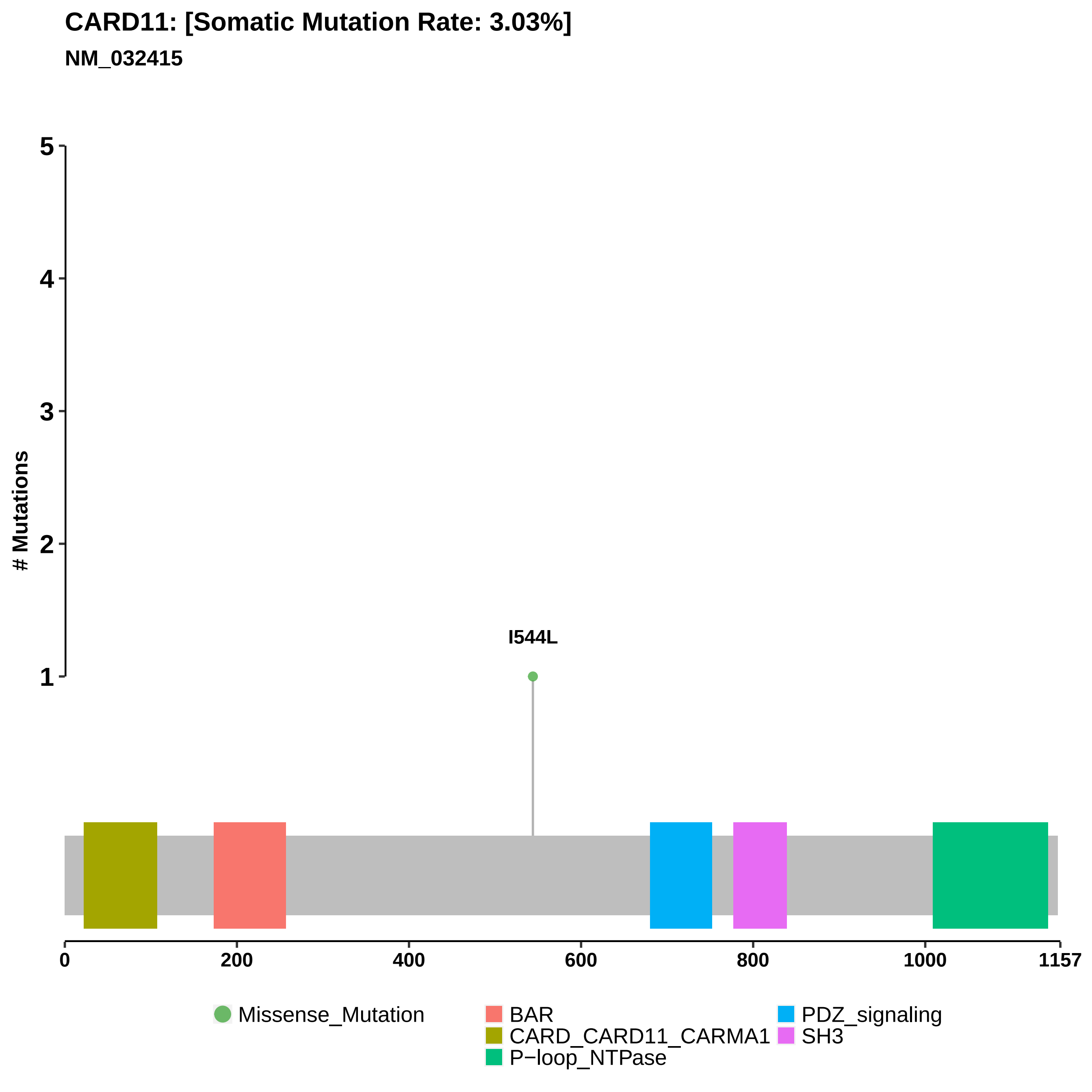
CARD11: [Somatic Mutation Rate: 3.03%]
NM_032415
Mutations
5
4
3
2
1
I544L
0
200
400
600
800
1000
1157
Missense_Mutation
BAR
CARD_CARD11_CARMA1
P−loop_NTPase
PDZ_signaling
SH3

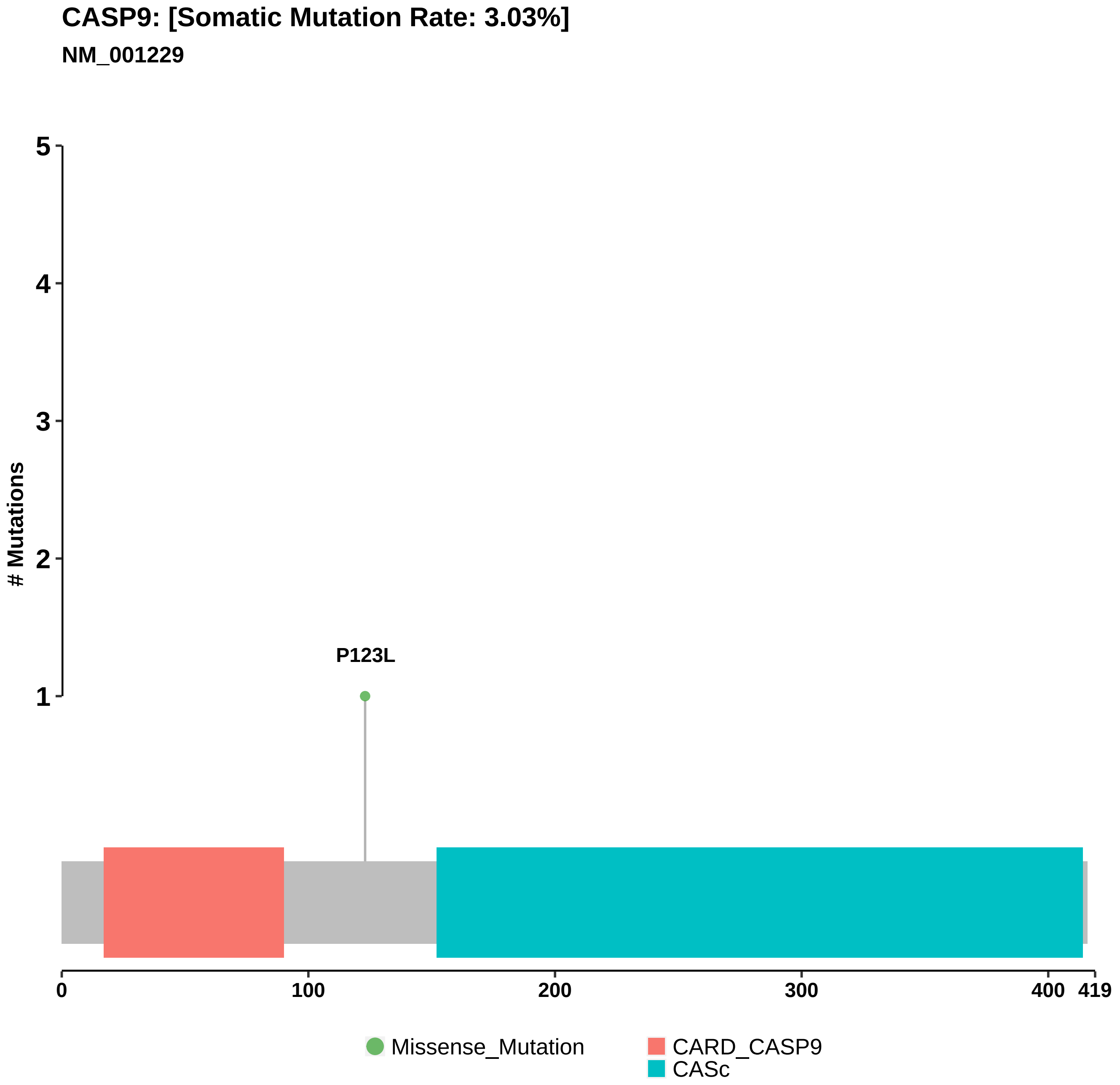
CASP9: [Somatic Mutation Rate: 3.03%]
NM_001229
Mutations
5
4
3
2
1
P123L
0
100
200
300
400
419
Missense_Mutation
CARD_CASP9
CASc

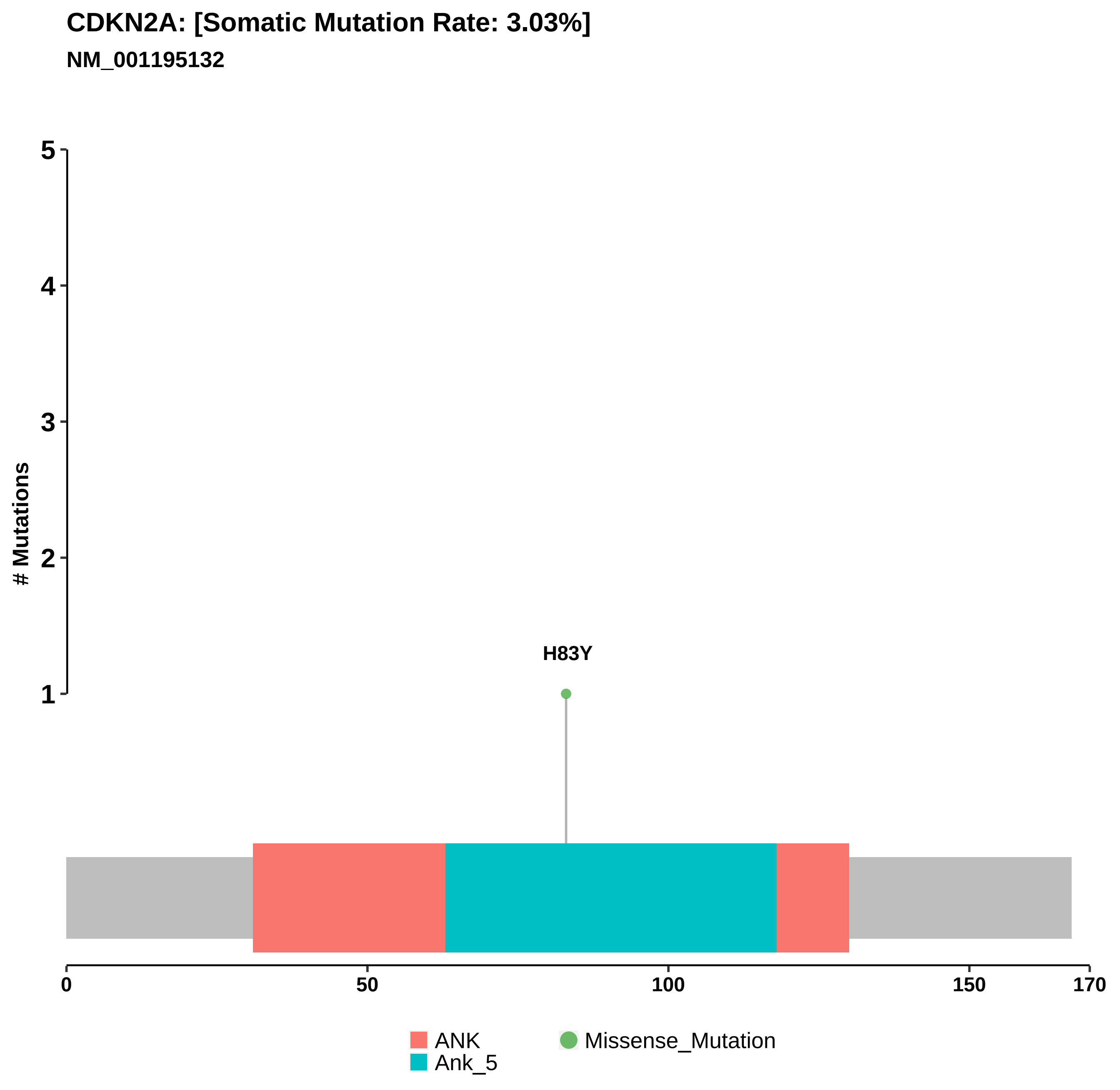

CDKN2A: [Somatic Mutation Rate: 3.03%]
NM_001195132
Mutations
5
4
3
2
1
H83Y
0
50
100
150
170
ANK
Ank_5
Missense_Mutation

CFLAR: [Somatic Mutation Rate: 3.03%]

NM_003879

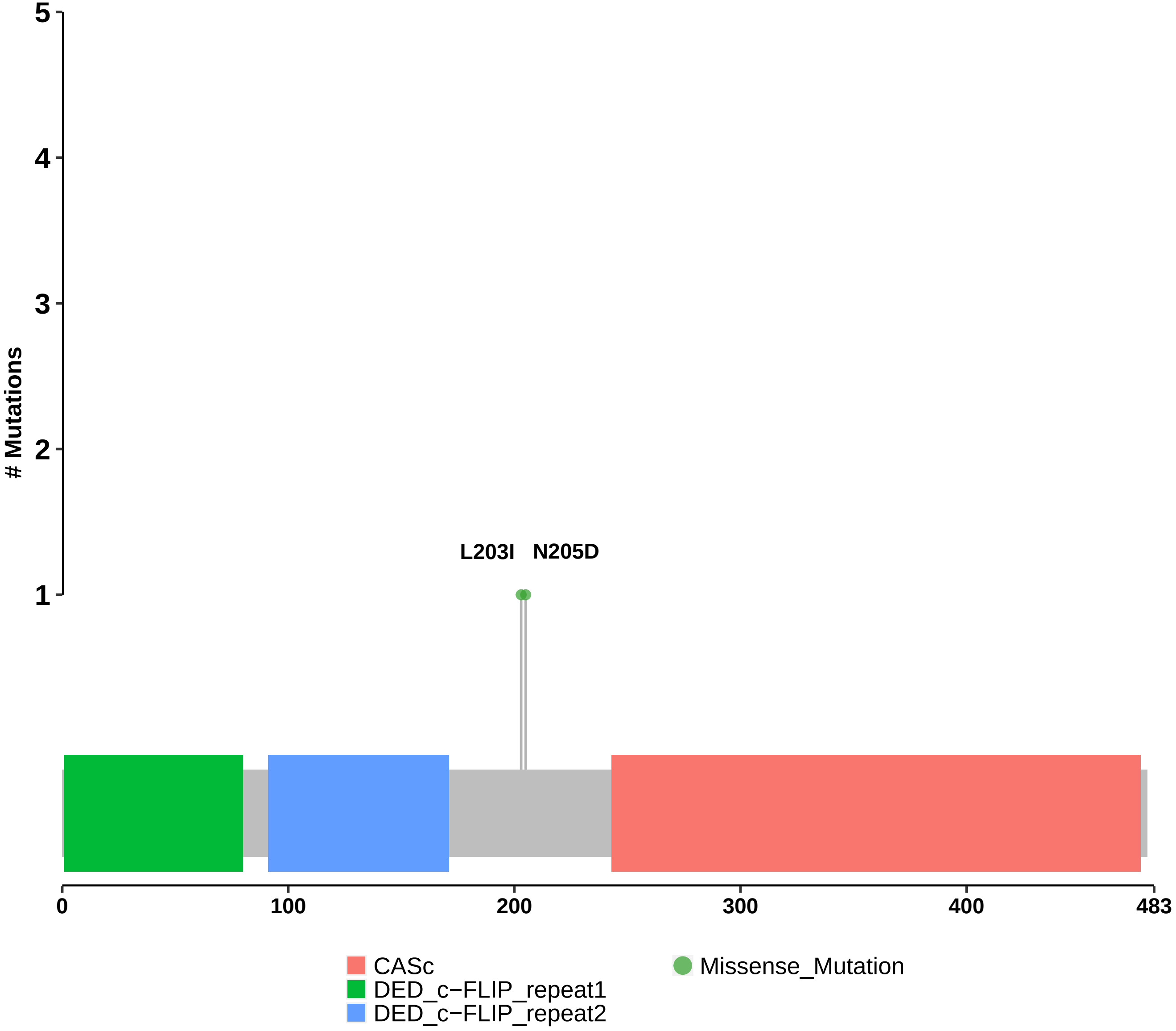

E2F1: [Somatic Mutation Rate: 3.03%]

NM_005225

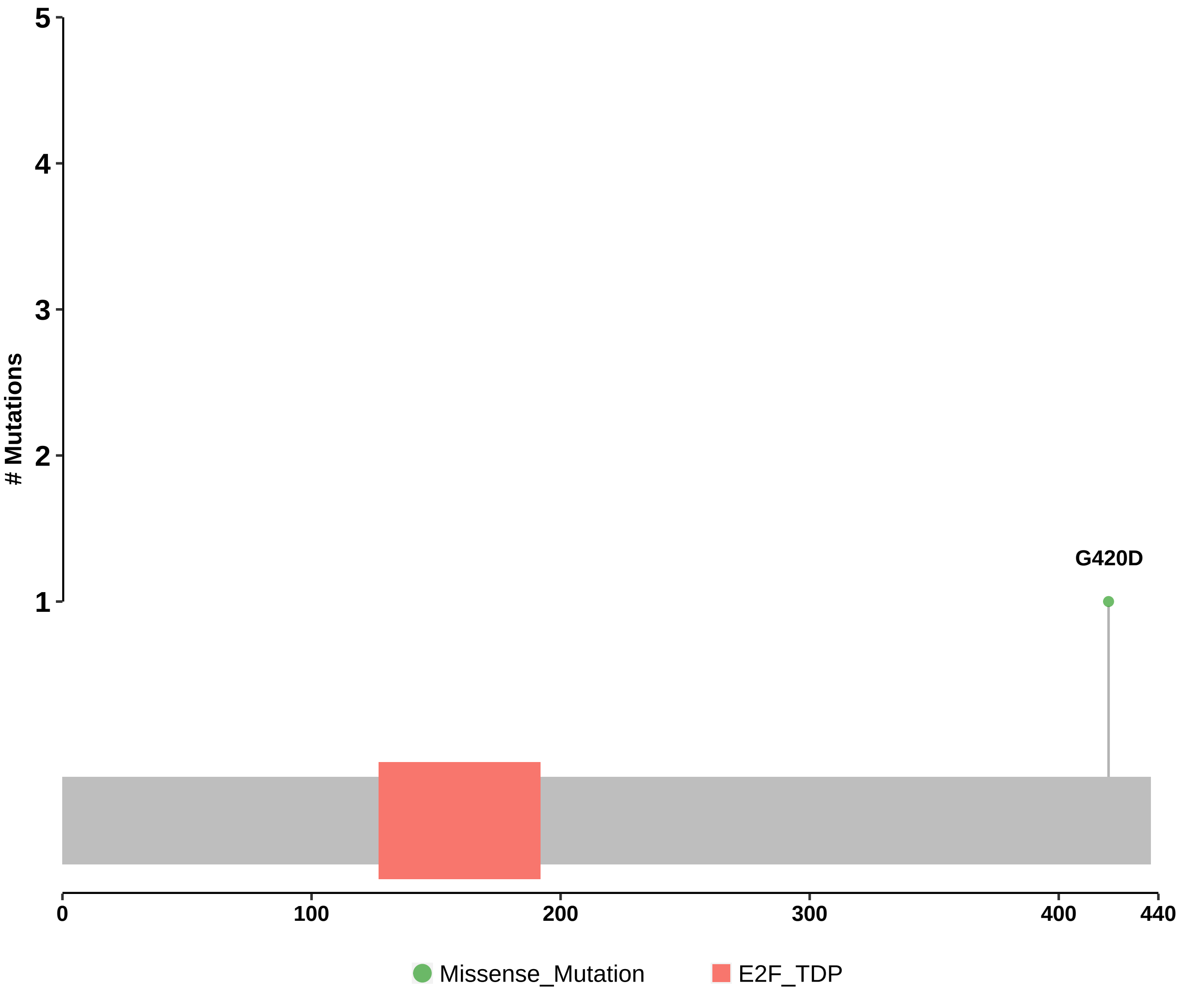

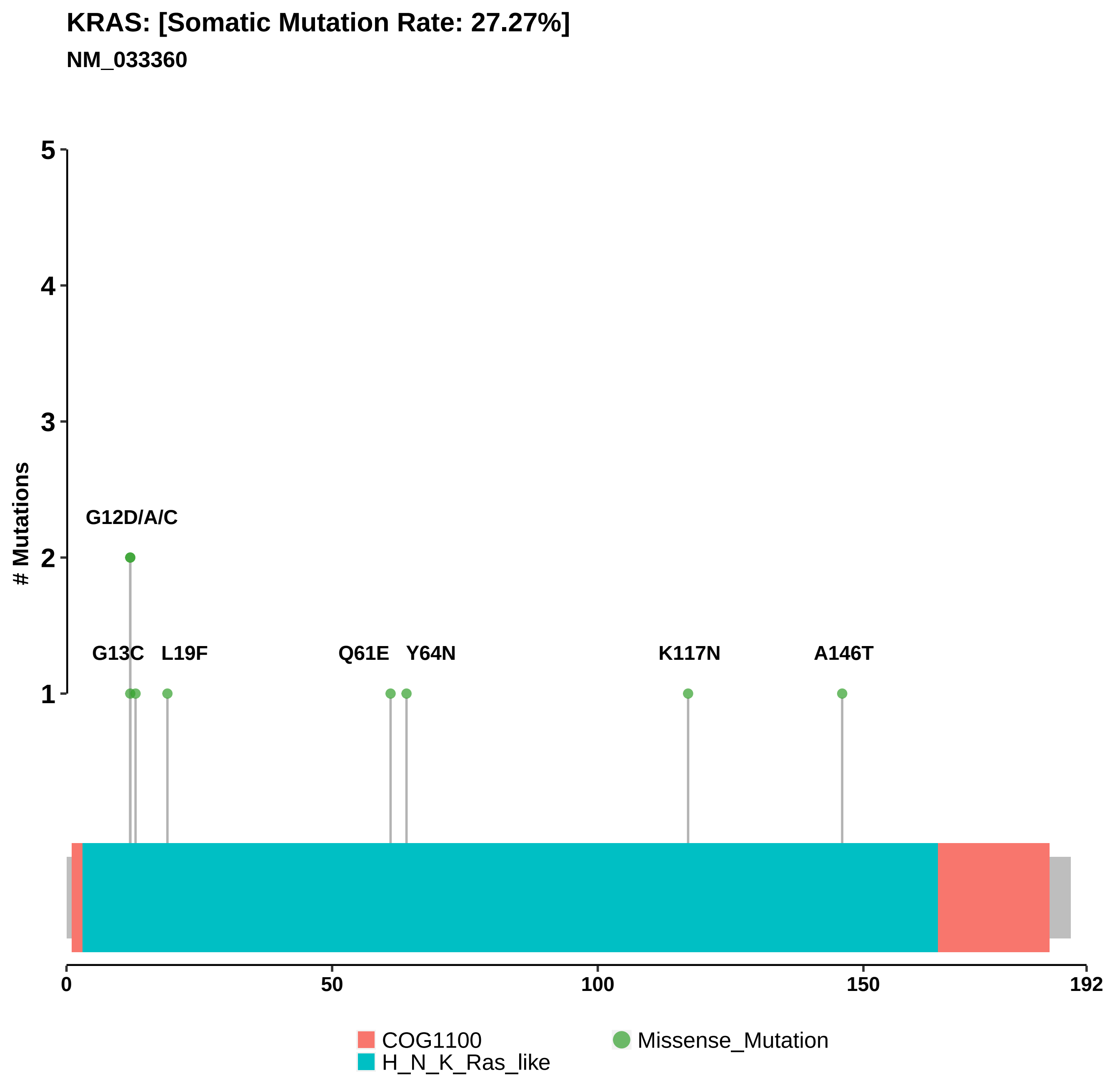
KRAS: [Somatic Mutation Rate: 27.27%]
NM_033360
Mutations
5
4
3
2
1
G12D/A/C
G13C
L19F
Q61E
Y64N
K117N
A146T
0
50
100
150
192
COG1100
H_N_K_Ras_like
Missense_Mutation

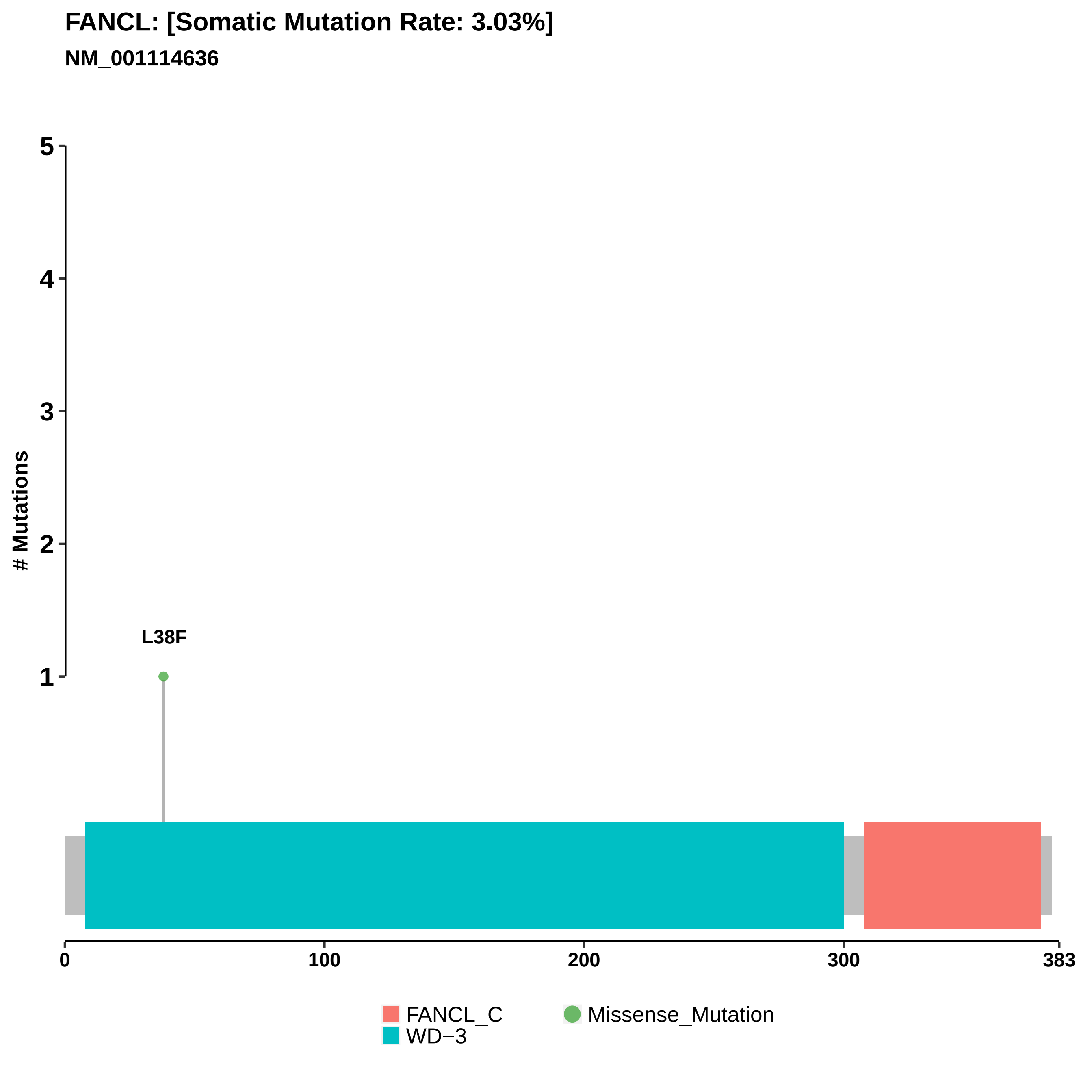
FANCL: [Somatic Mutation Rate: 3.03%]
NM_001114636
Mutations
5
4
3
2
1
L38F
0
100
200
300
383
FANCL_C
WD−3
Missense_Mutation

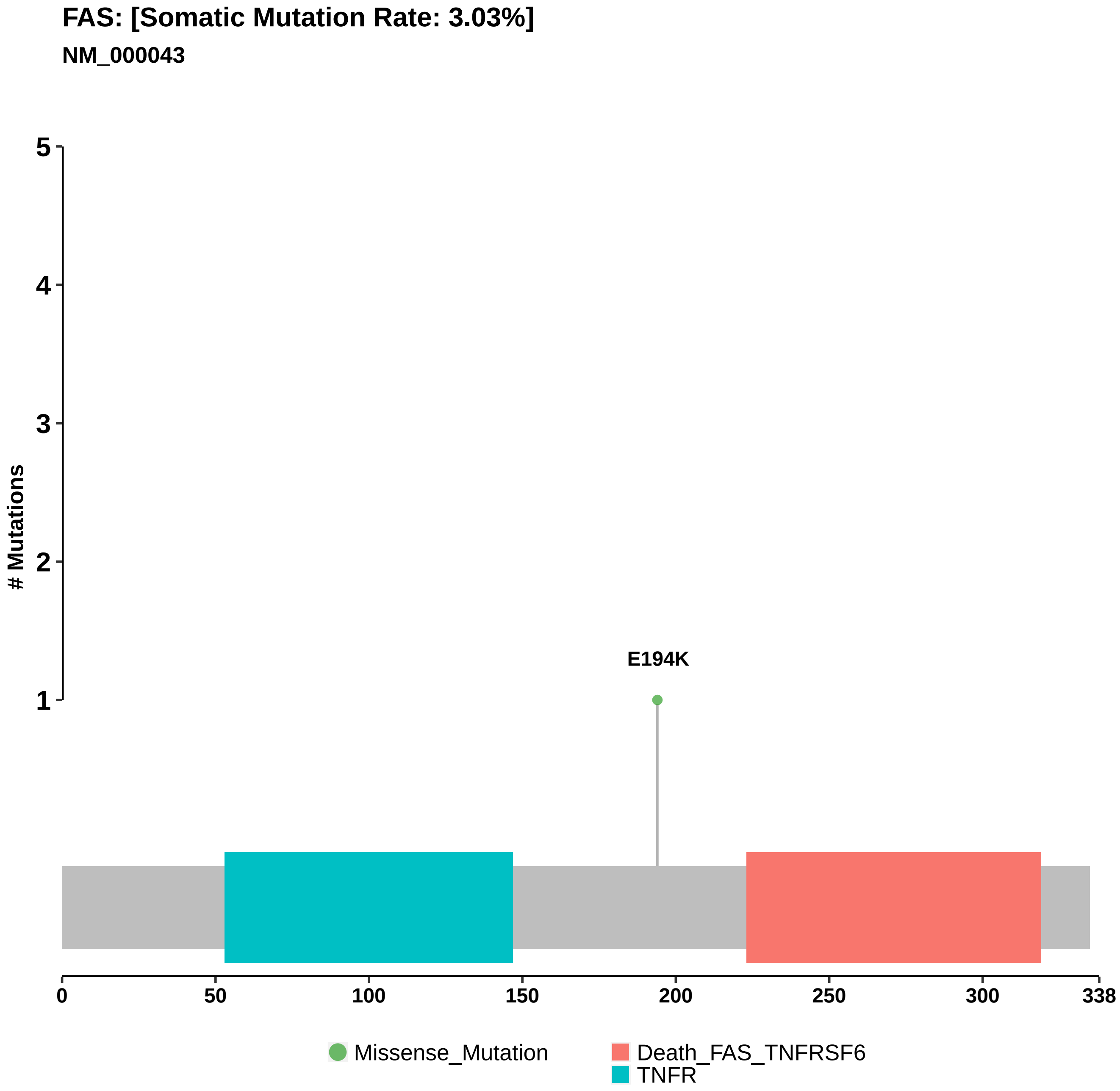
FAS: [Somatic Mutation Rate: 3.03%]
NM_000043
Mutations
5
4
3
2
1
E194K
0
50
100
150
200
250
300
338
Missense_Mutation
Death_FAS_TNFRSF6
TNFR

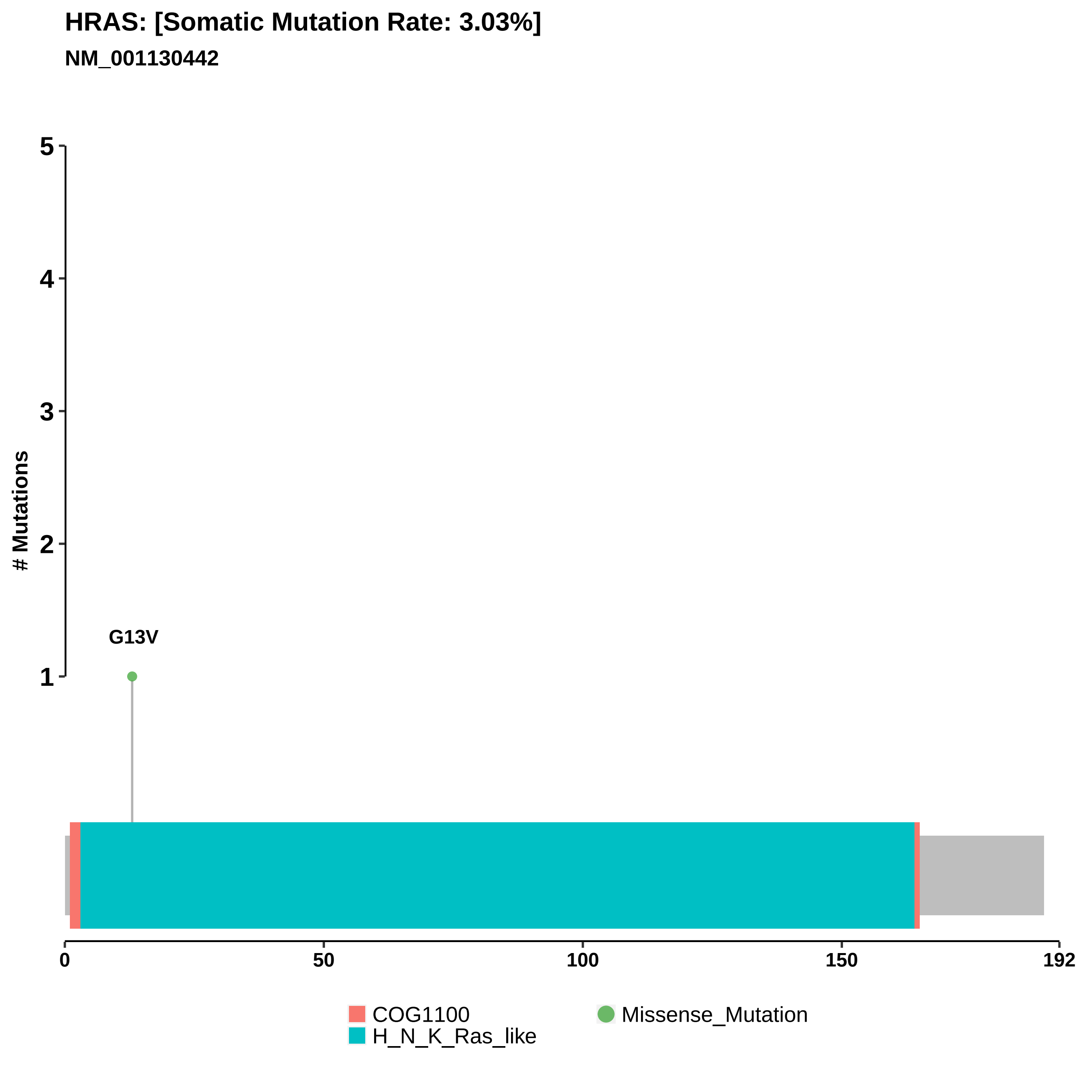
HRAS: [Somatic Mutation Rate: 3.03%]
NM_001130442
Mutations
5
4
3
2
1
G13V
0
50
100
150
192
COG1100
H_N_K_Ras_like
Missense_Mutation

MCL1: [Somatic Mutation Rate: 3.03%]

NM_021960

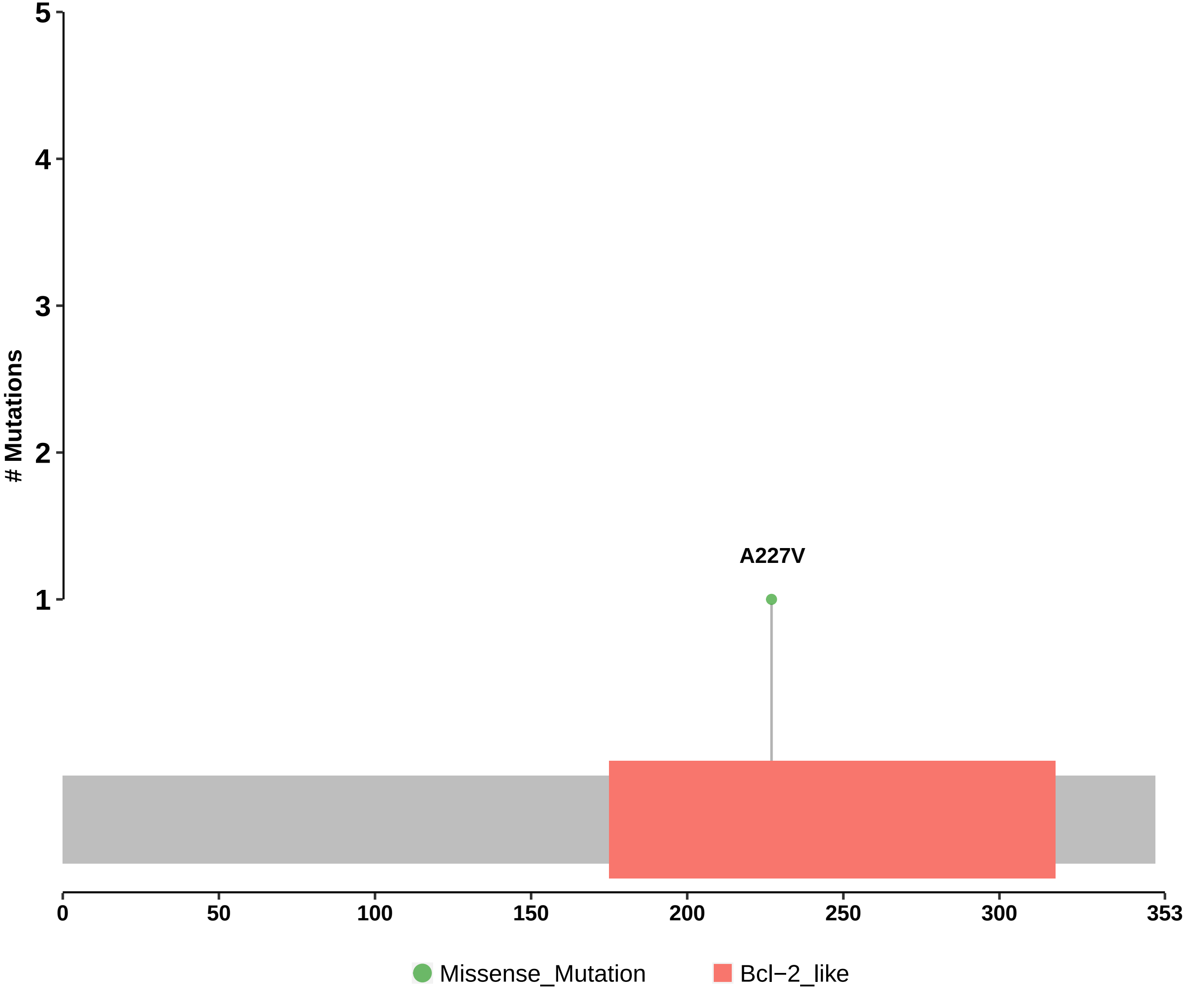

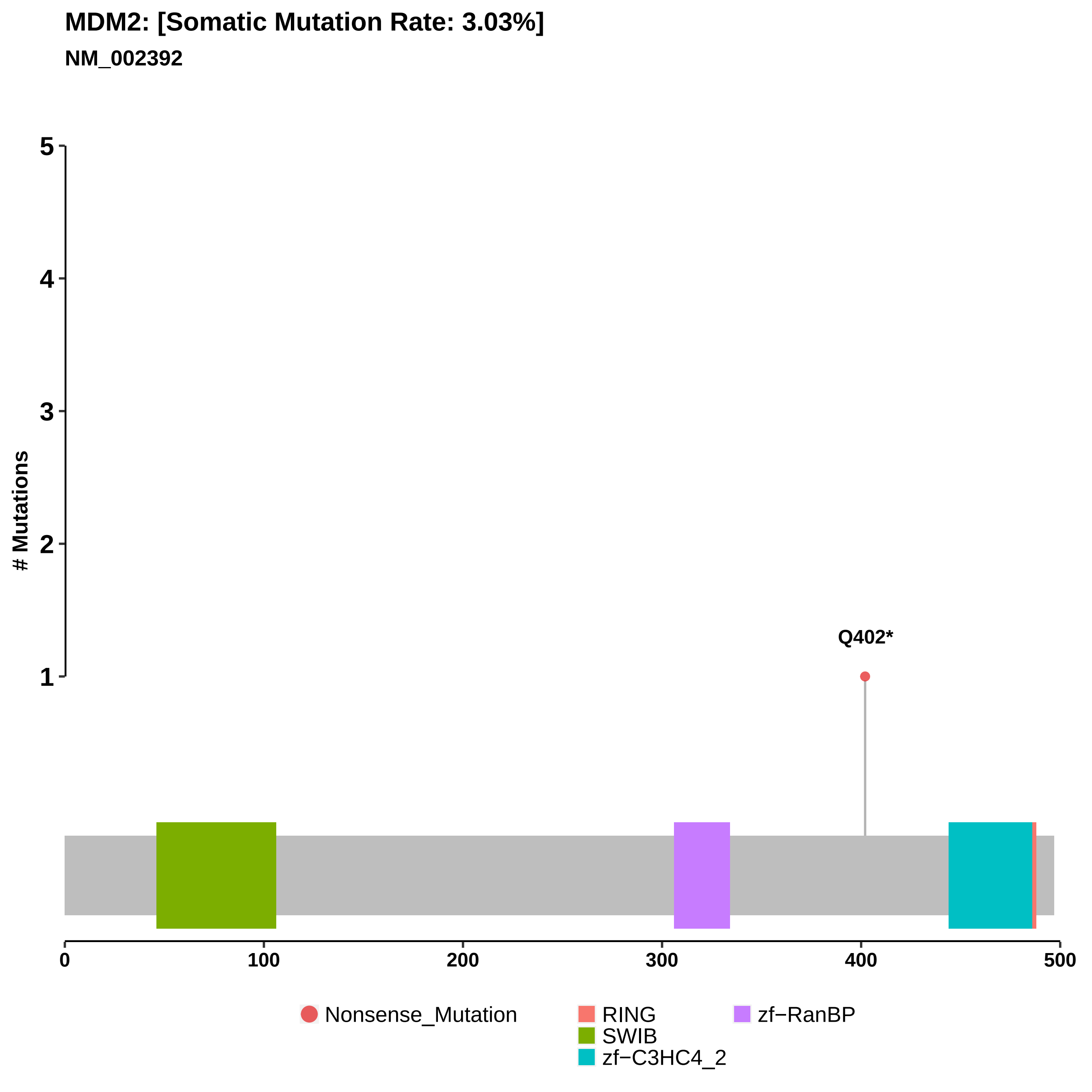
MDM2: [Somatic Mutation Rate: 3.03%]
NM_002392
Mutations
5
4
3
2
1
Q402*
0
100
200
300
400
500
Nonsense_Mutation
RING
SWIB
zf−C3HC4_2
zf−RanBP

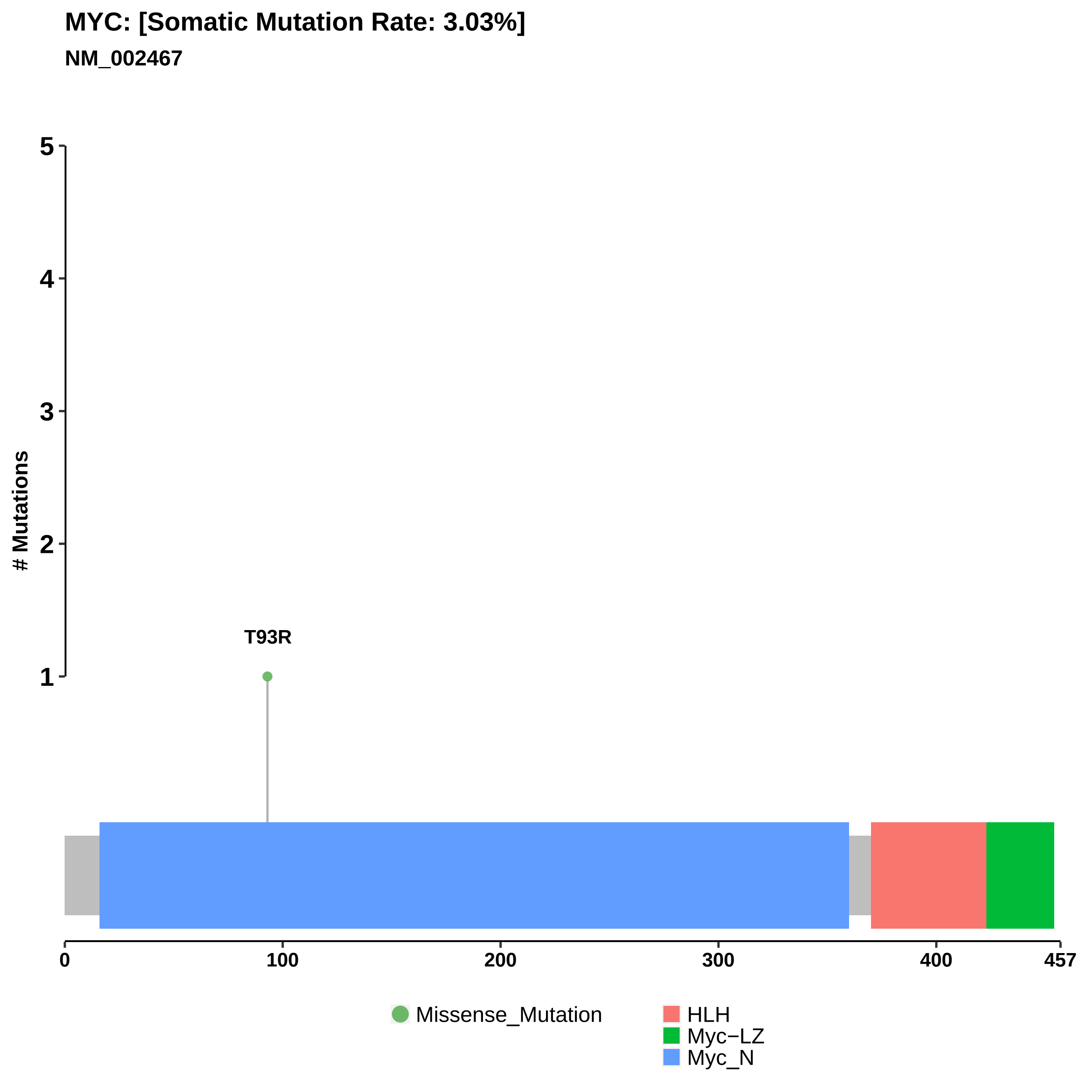
MYC: [Somatic Mutation Rate: 3.03%]
NM_002467
Mutations
5
4
3
2
1
T93R
0
100
200
300
400
457
Missense_Mutation
HLH
Myc−LZ
Myc_N

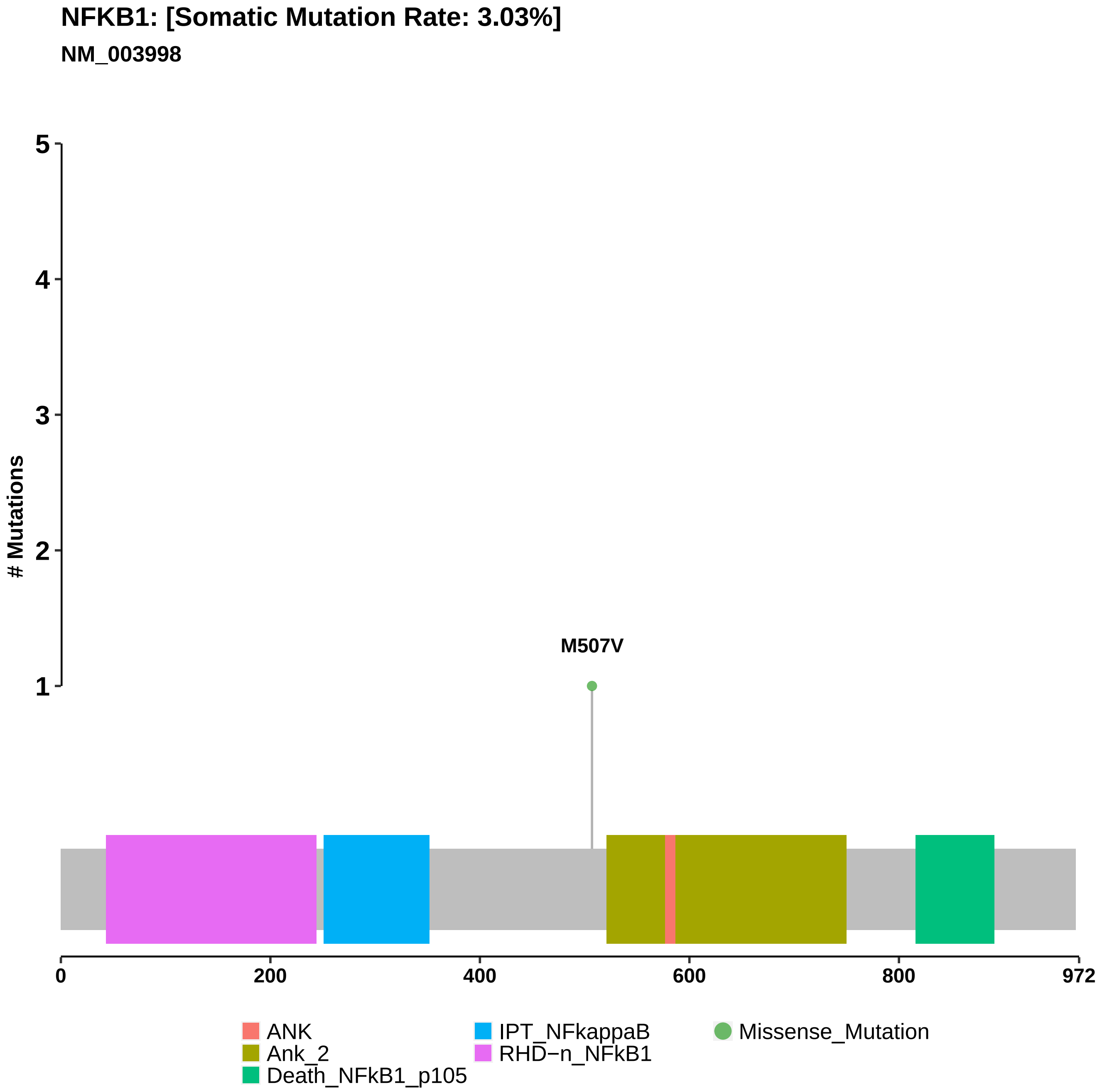
NFKB1: [Somatic Mutation Rate: 3.03%]
NM_003998
Mutations
5
4
3
2
1
M507V
0
200
400
600
800
972
ANK
Ank_2
Death_NFkB1_p105
IPT_NFkappaB
RHD−n_NFkB1
Missense_Mutation

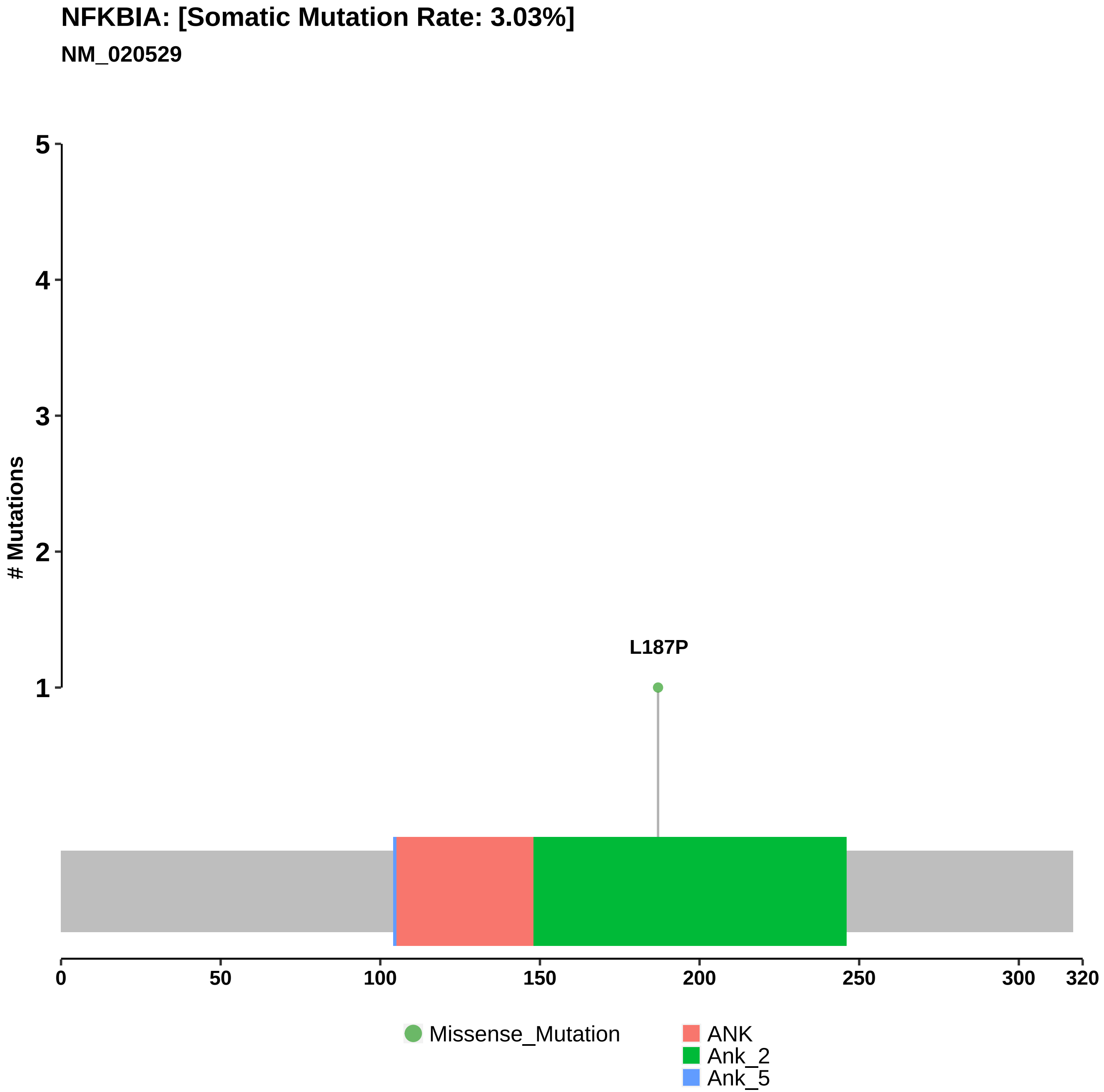
NFKBIA: [Somatic Mutation Rate: 3.03%]
NM_020529
Mutations
5
4
3
2
1
L187P
0
50
100
150
200
250
300
320
Missense_Mutation
ANK
Ank_2
Ank_5

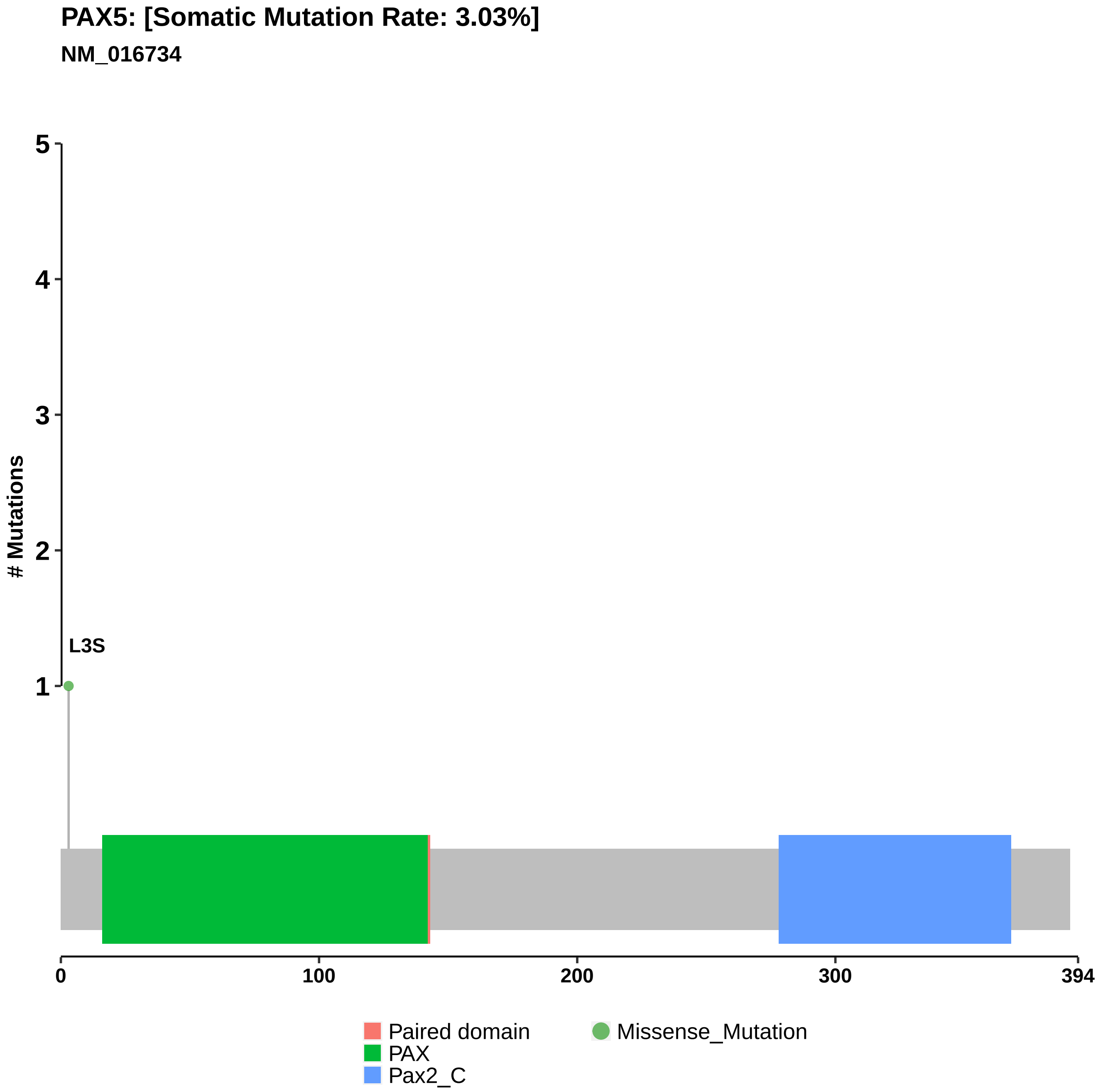

PAX5: [Somatic Mutation Rate: 3.03%]
NM_016734
Mutations
5
4
3
2
1
L3S
0
100
200
300
394
Paired domain
PAX
Pax2_C
Missense_Mutation

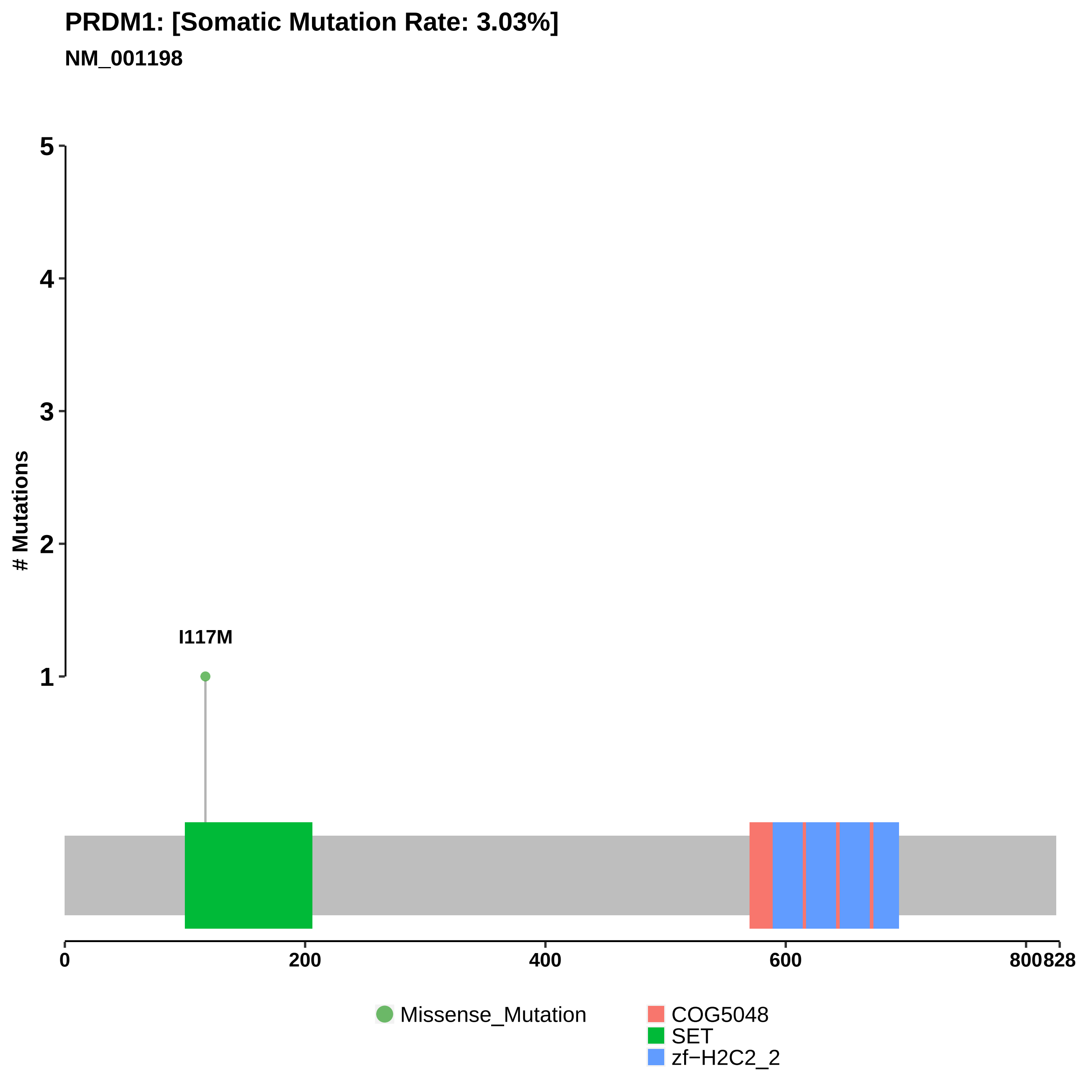

PRDM1: [Somatic Mutation Rate: 3.03%]
NM_001198
Mutations
5
4
3
2
1
I117M
0
200
400
600
800828
Missense_Mutation
COG5048
SET
zf−H2C2_2

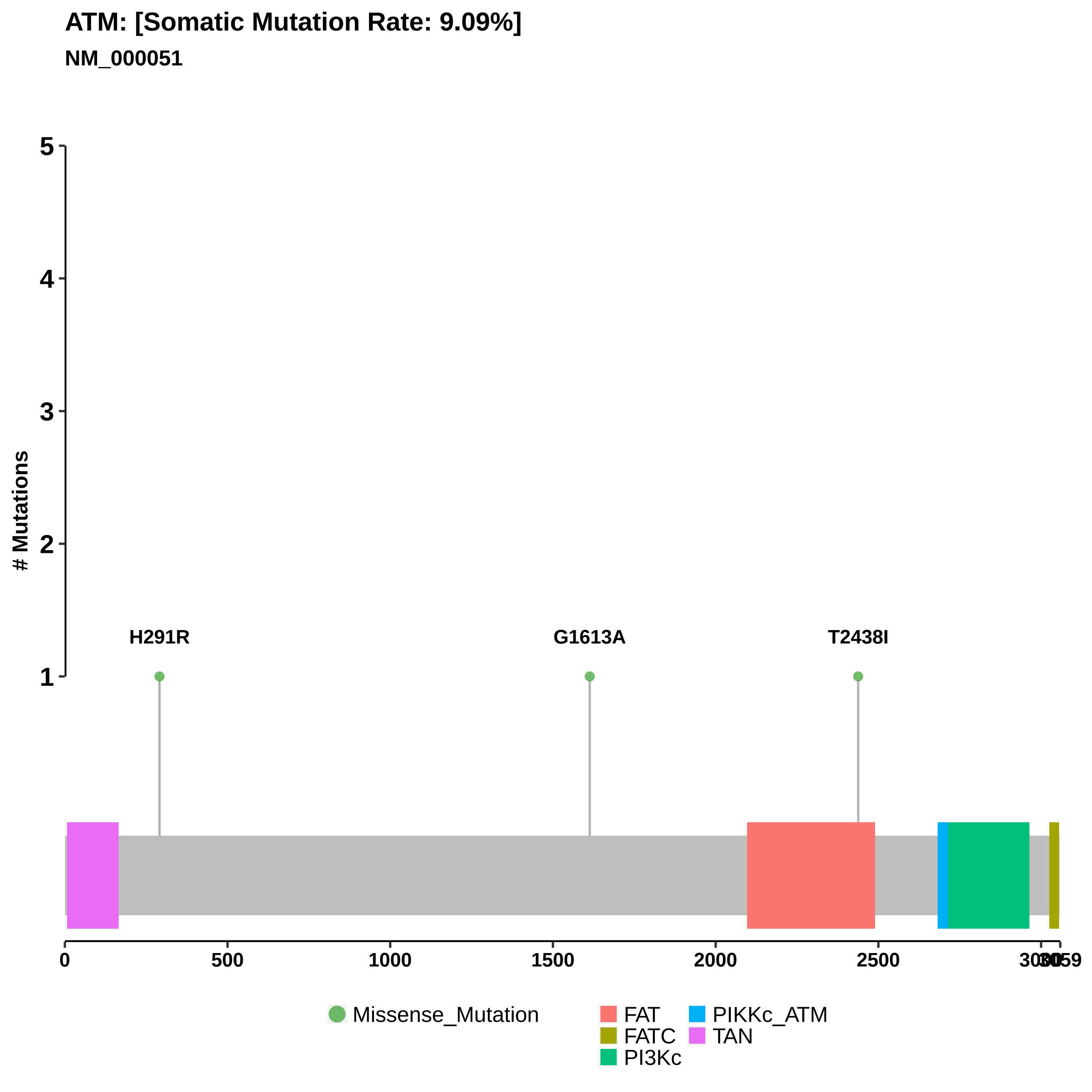
ATM: [Somatic Mutation Rate: 9.09%]
NM_000051
Mutations
5
4
3
2
1
H291R
G1613A
T2438I
0
500
1000
1500
2000
2500
3000
3059
Missense_Mutation
FAT
FATC
PI3Kc
PIKKc_ATM
TAN

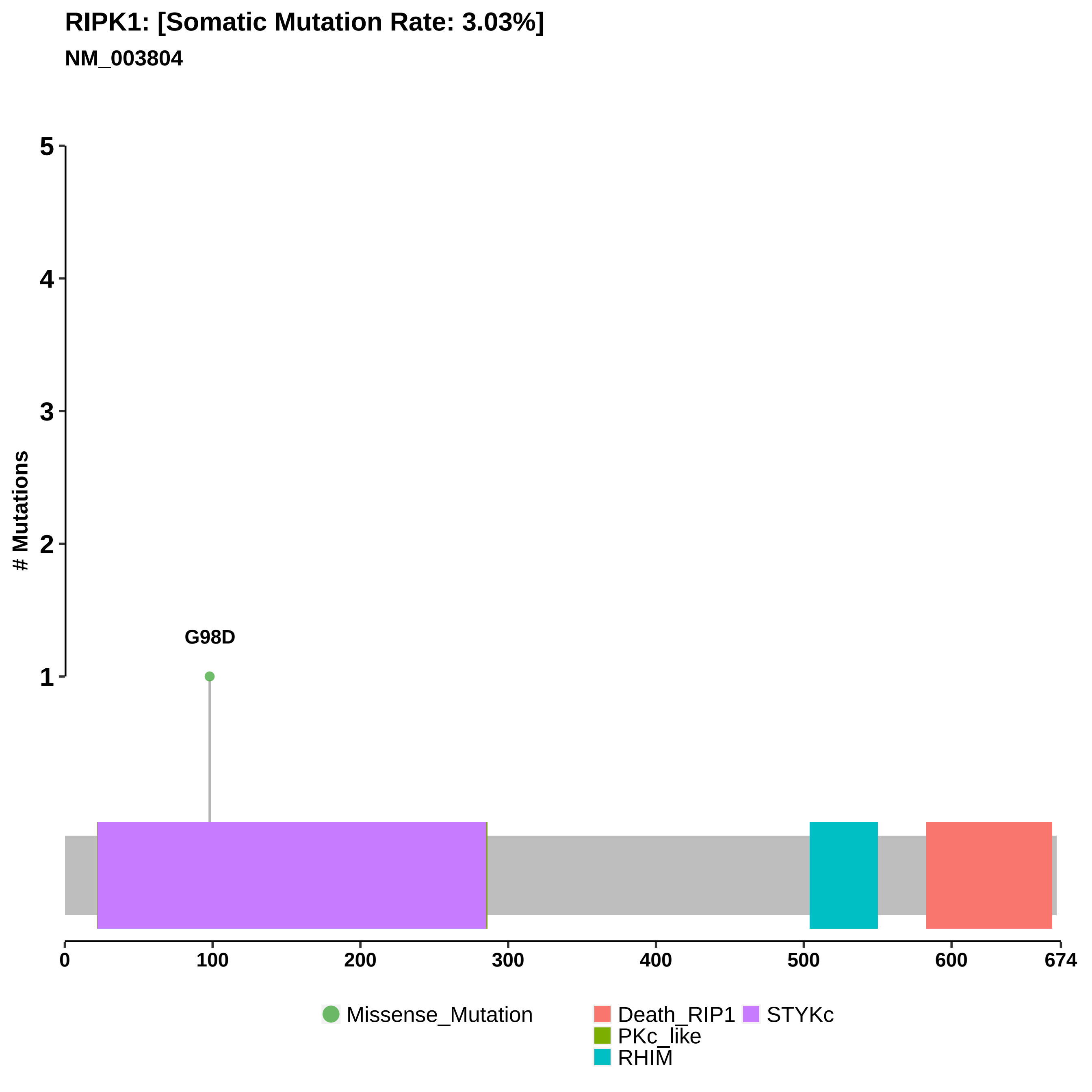
RIPK1: [Somatic Mutation Rate: 3.03%]
NM_003804
Mutations
5
4
3
2
1
G98D
0
100
200
300
400
500
600
674
Missense_Mutation
Death_RIP1
PKc_like
RHIM
STYKc

FGFR3: [Somatic Mutation Rate: 12.12%]

NM_001163213

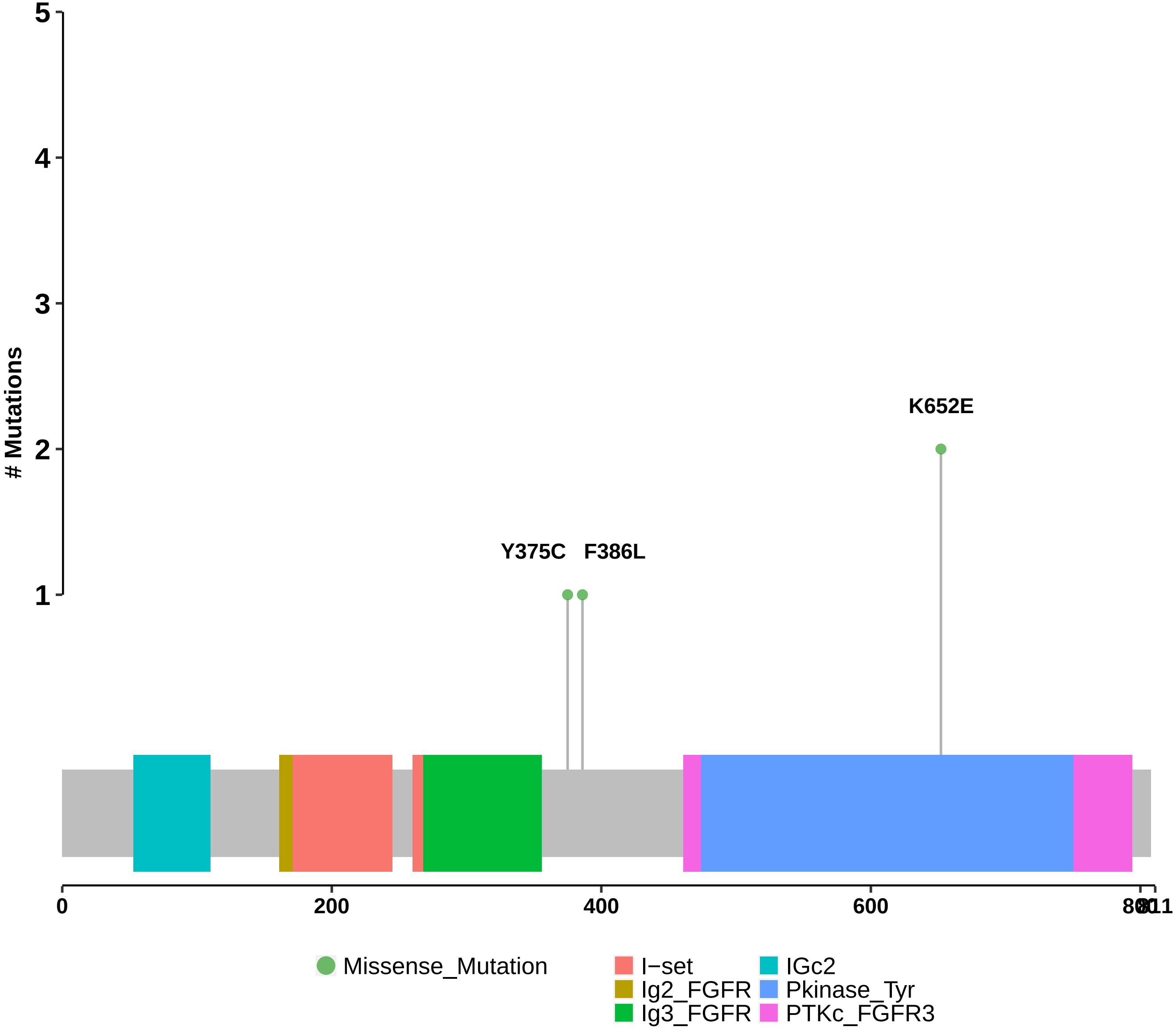

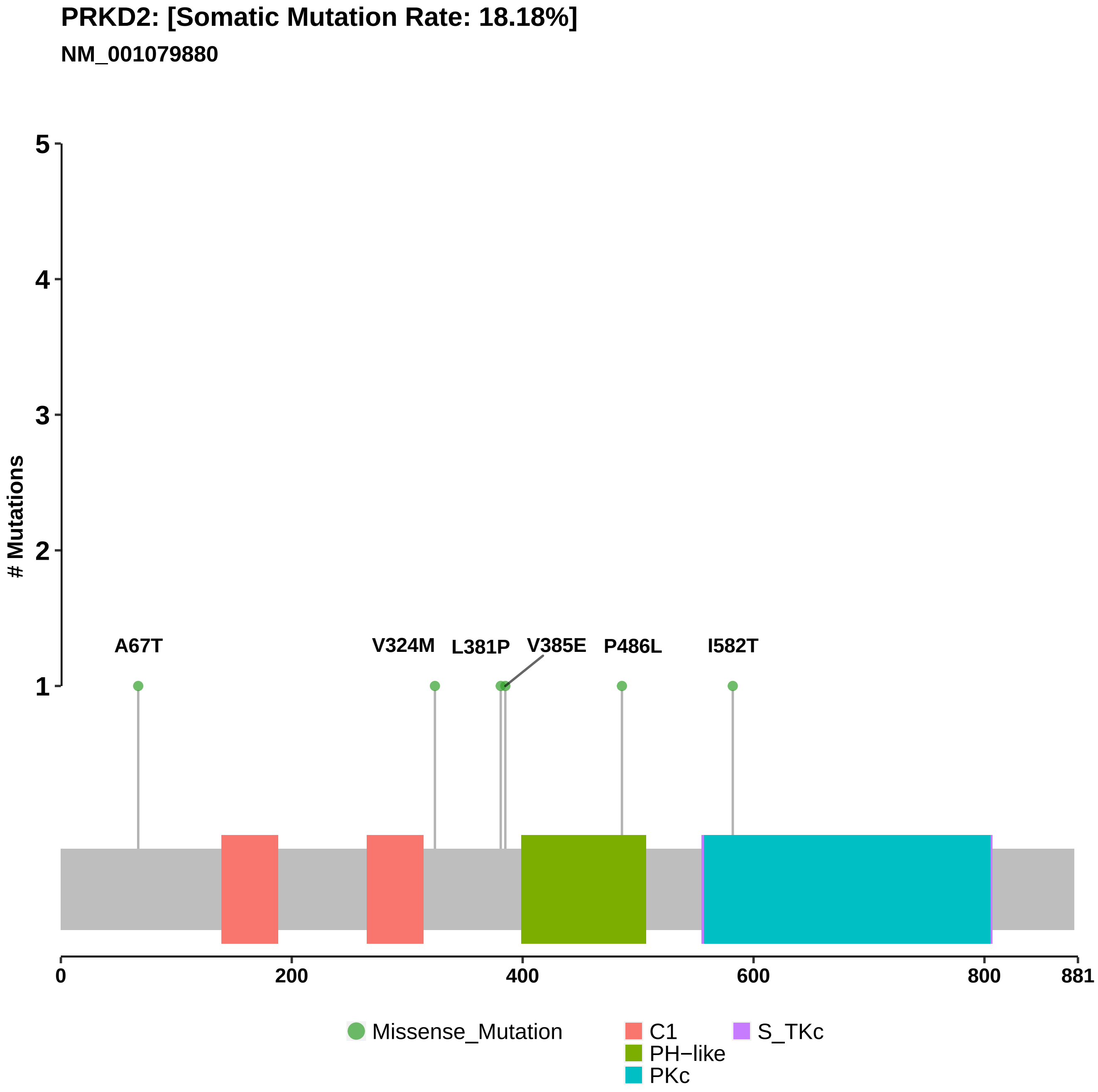
PRKD2: [Somatic Mutation Rate: 18.18%]
NM_001079880
Mutations
5
4
3
2
1
A67T
V324M
L381P
V385E
P486L
I582T
0
200
400
600
800
881
Missense_Mutation
C1
PH−like
PKc
S_TKc

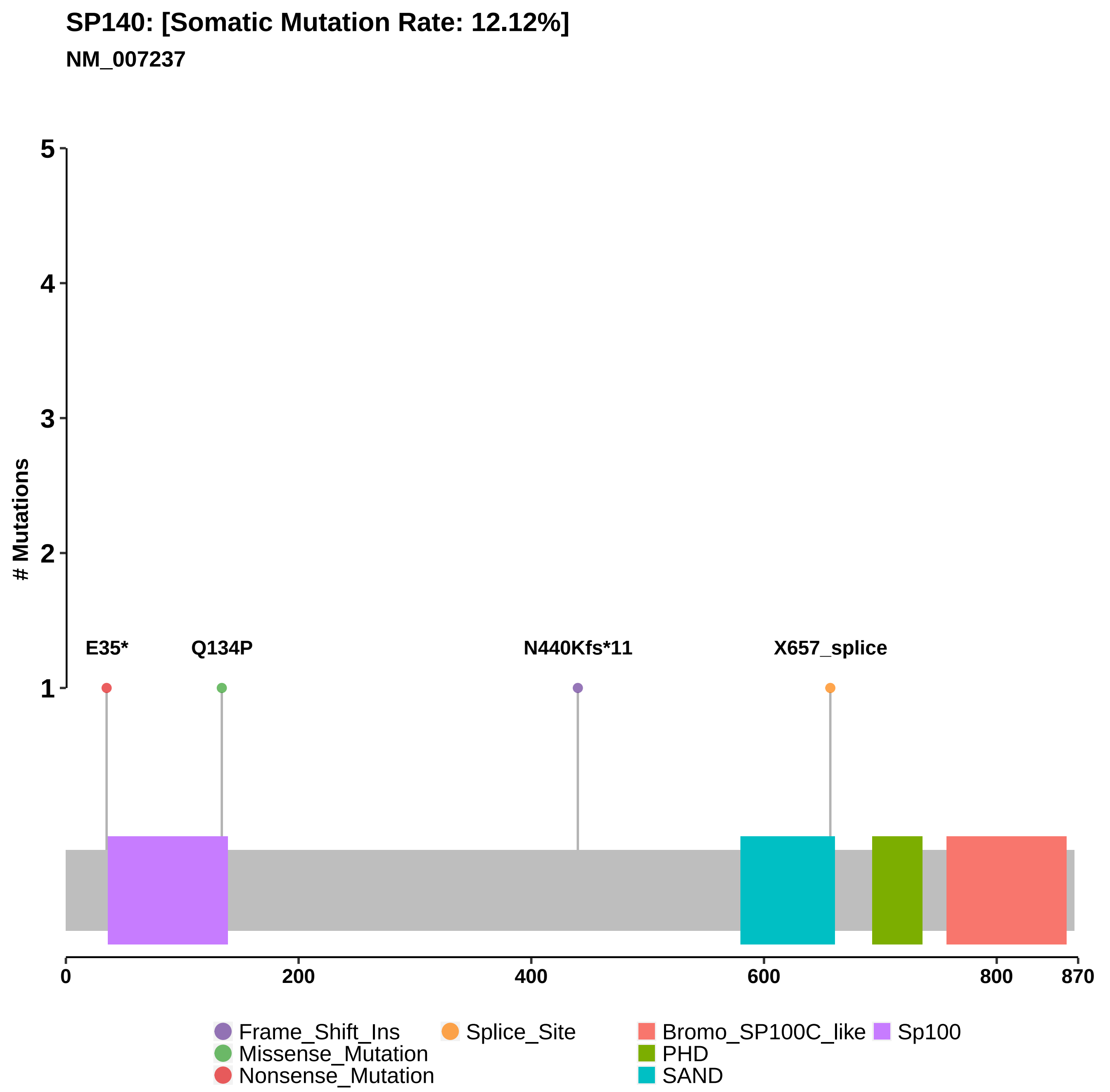
SP140: [Somatic Mutation Rate: 12.12%]
NM_007237
Mutations
5
4
3
2
1
E35*
Q134P
N440Kfs*11
X657_splice
0
200
400
600
800
870
Frame_Shift_Ins
Missense_Mutation
Nonsense_Mutation
Splice_Site
Bromo_SP100C_like
PHD
SAND
Sp100

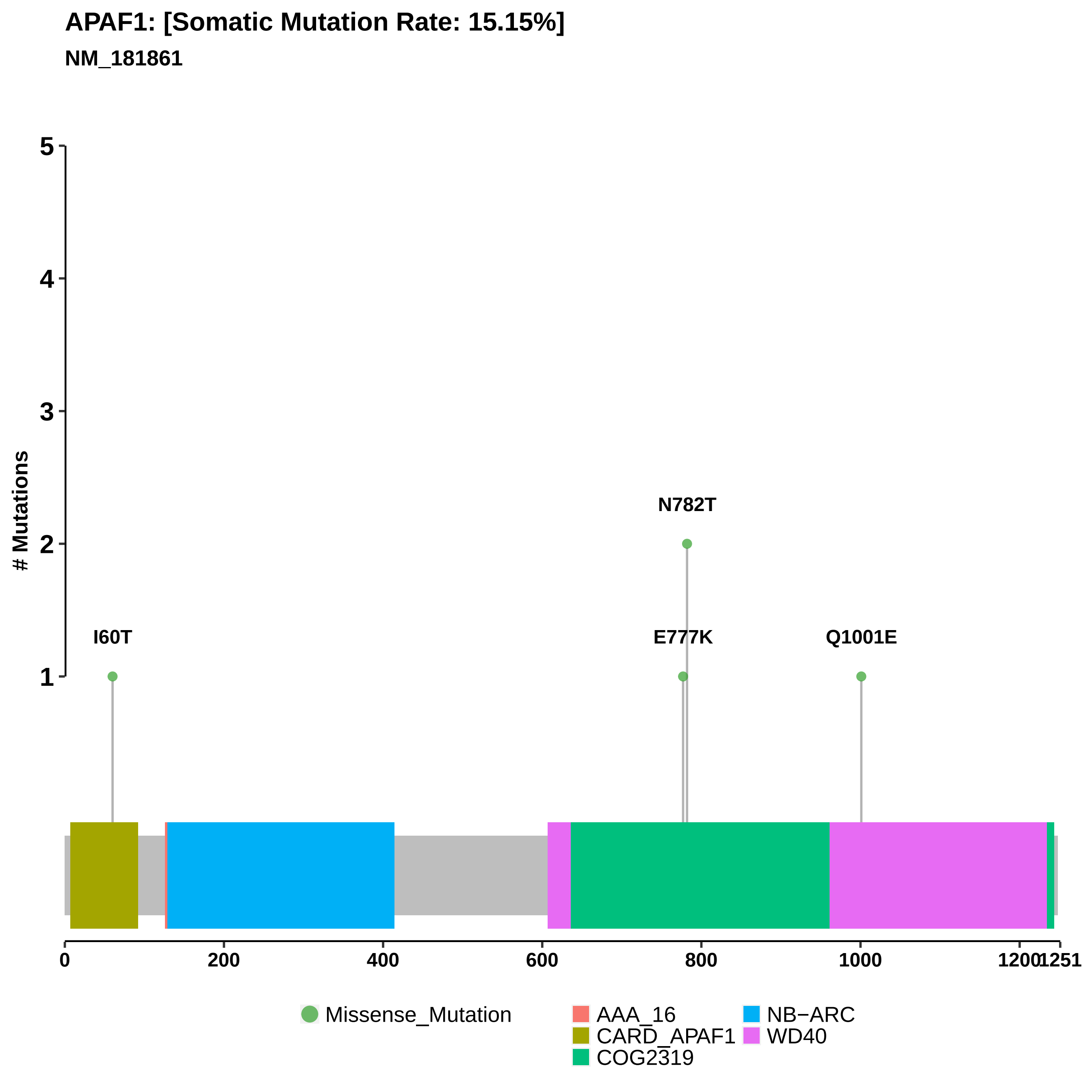
APAF1: [Somatic Mutation Rate: 15.15%]
NM_181861
Mutations
5
4
3
2
1
I60T
N782T
E777K
Q1001E
0
200
400
600
800
1000
1200
1251
Missense_Mutation
AAA_16
CARD_APAF1
COG2319
NB−ARC
WD40

IFNAR1: [Somatic Mutation Rate: 6.06%]

NM_000629

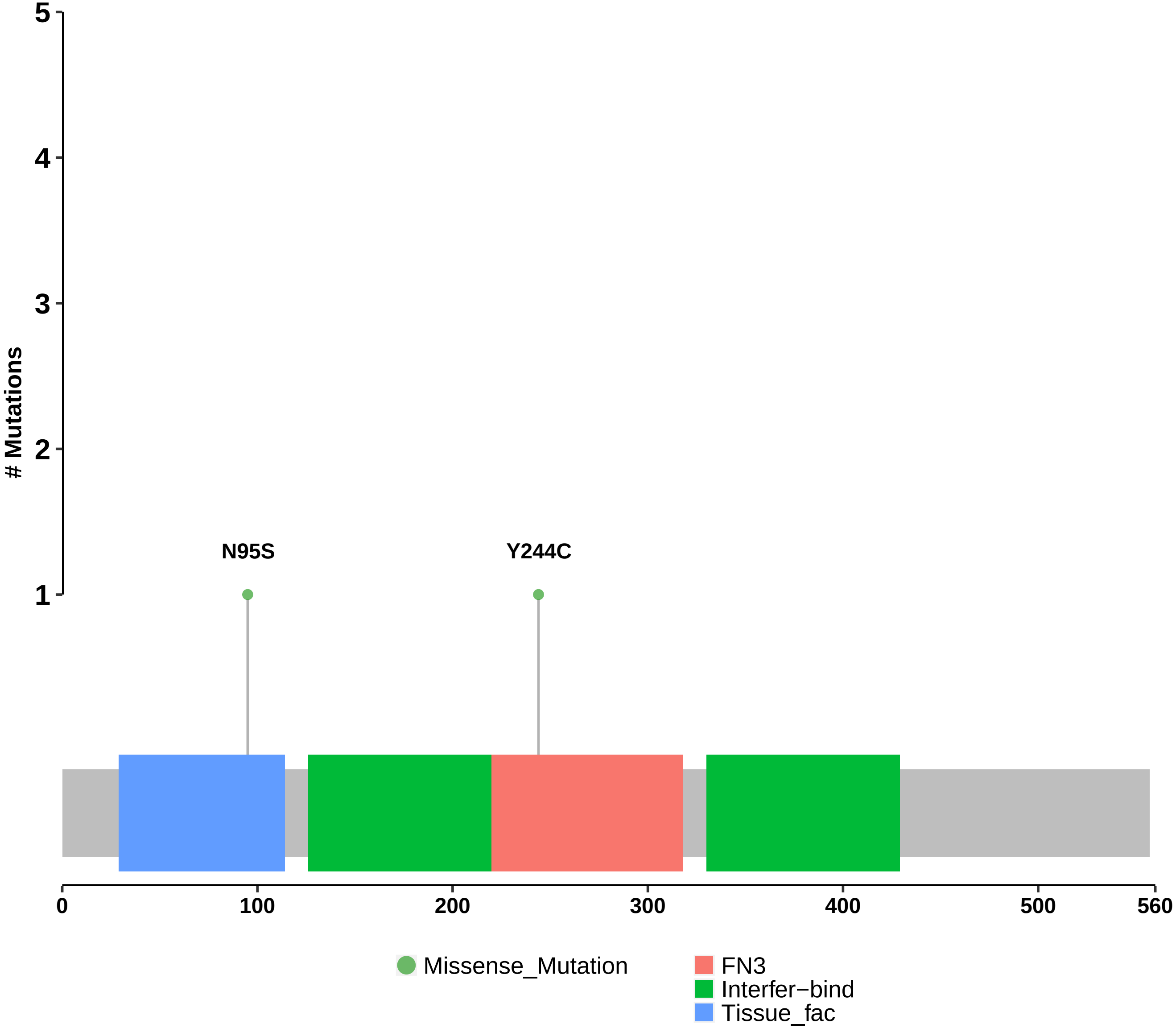

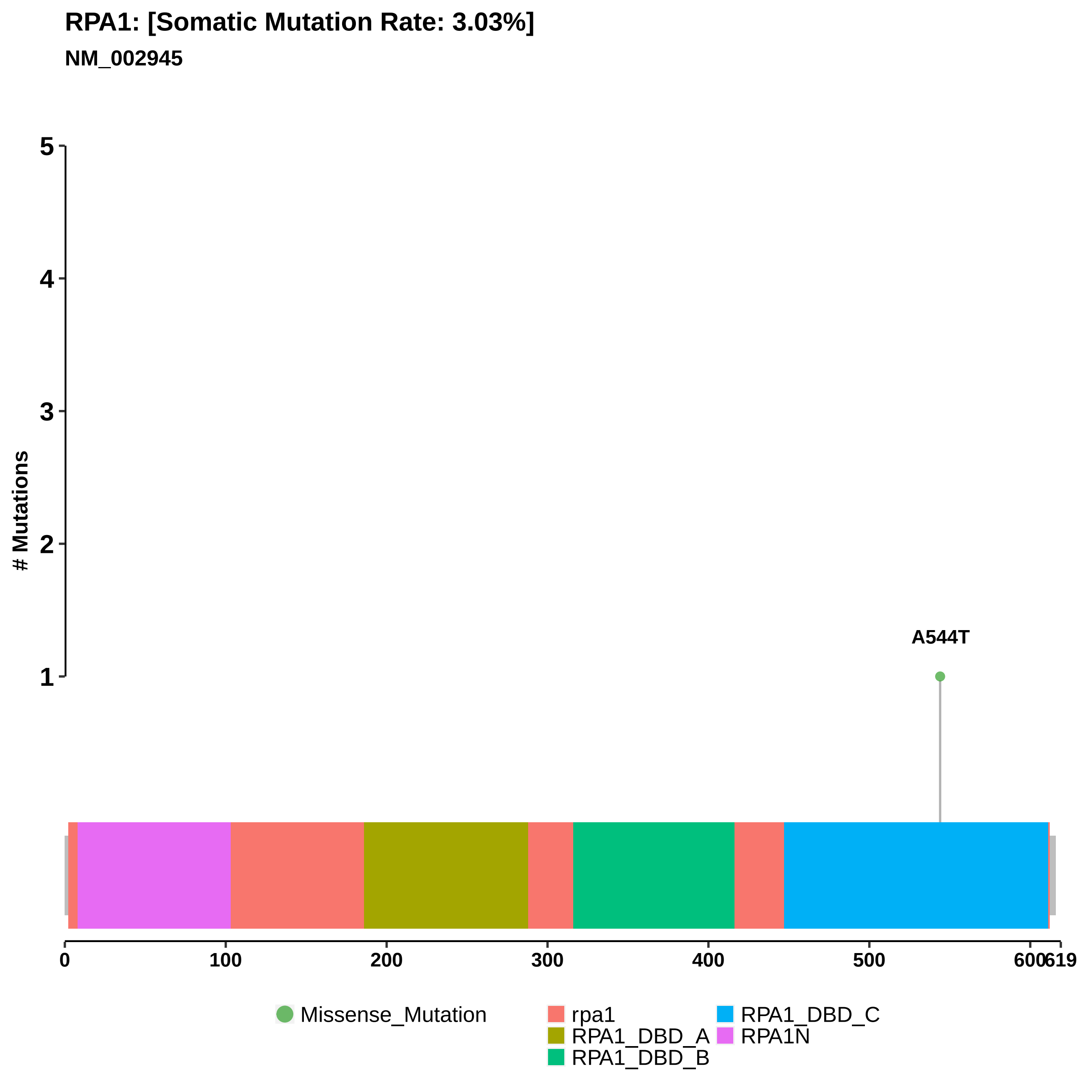

RPA1: [Somatic Mutation Rate: 3.03%]
NM_002945
Mutations
5
4
3
2
1
A544T
0
100
200
300
400
500
600
619
Missense_Mutation
rpa1
RPA1_DBD_A
RPA1_DBD_B
RPA1_DBD_C
RPA1N

RPA2: [Somatic Mutation Rate: 3.03%]

NM_002946

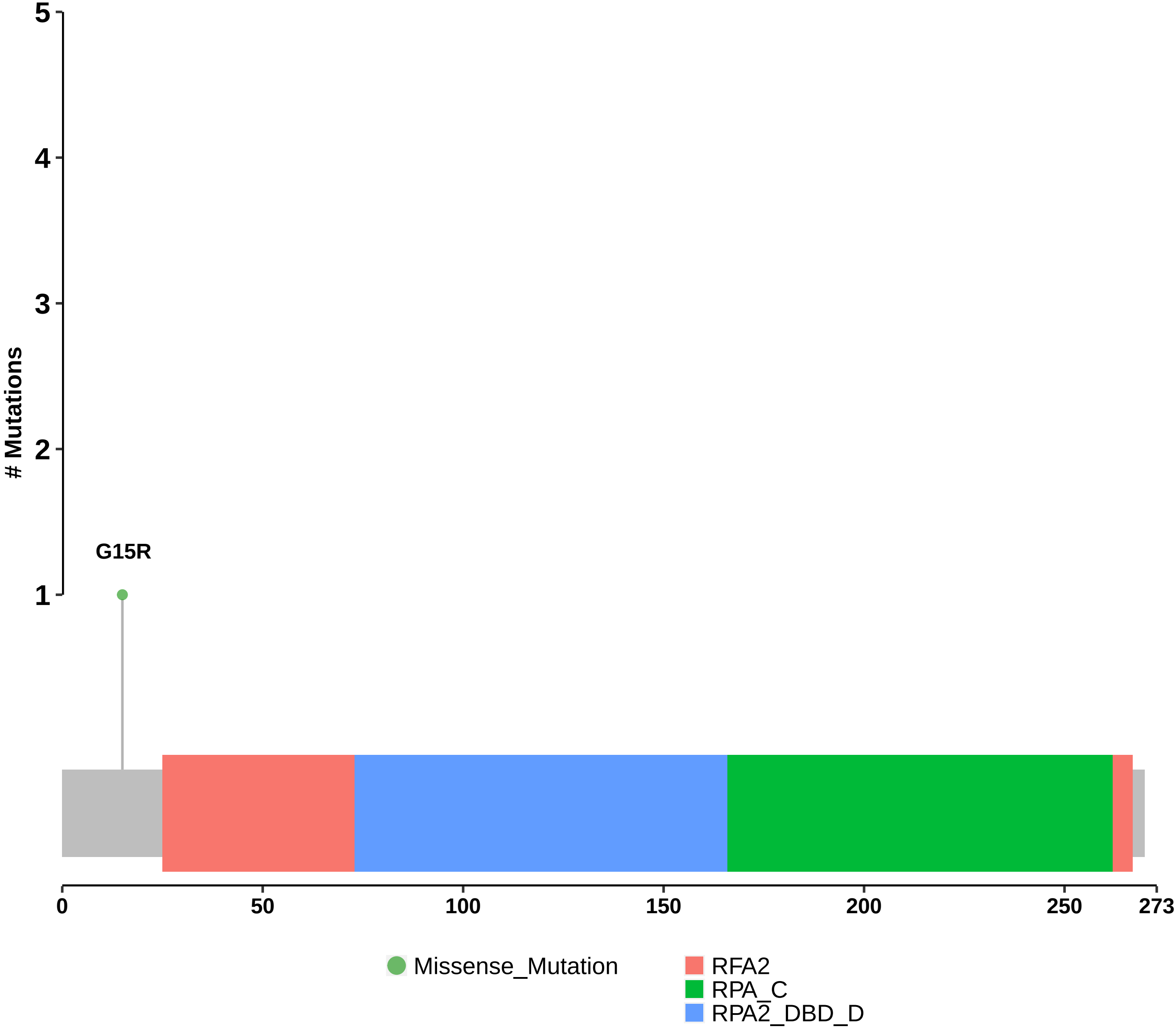

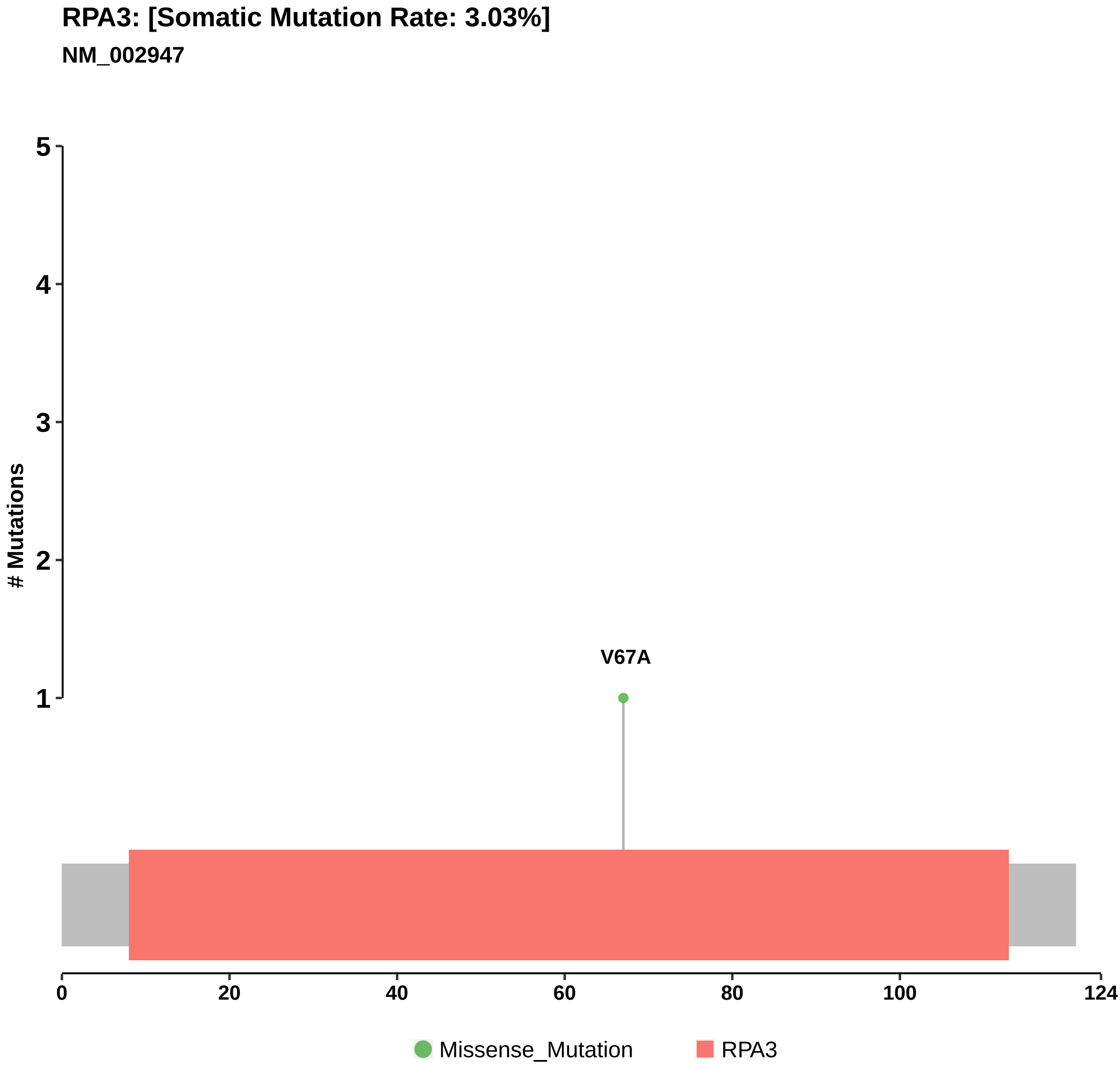
RPA3: [Somatic Mutation Rate: 3.03%]
NM_002947
Mutations
5
4
3
2
1
V67A
0
20
40
60
80
100
124
Missense_Mutation
RPA3

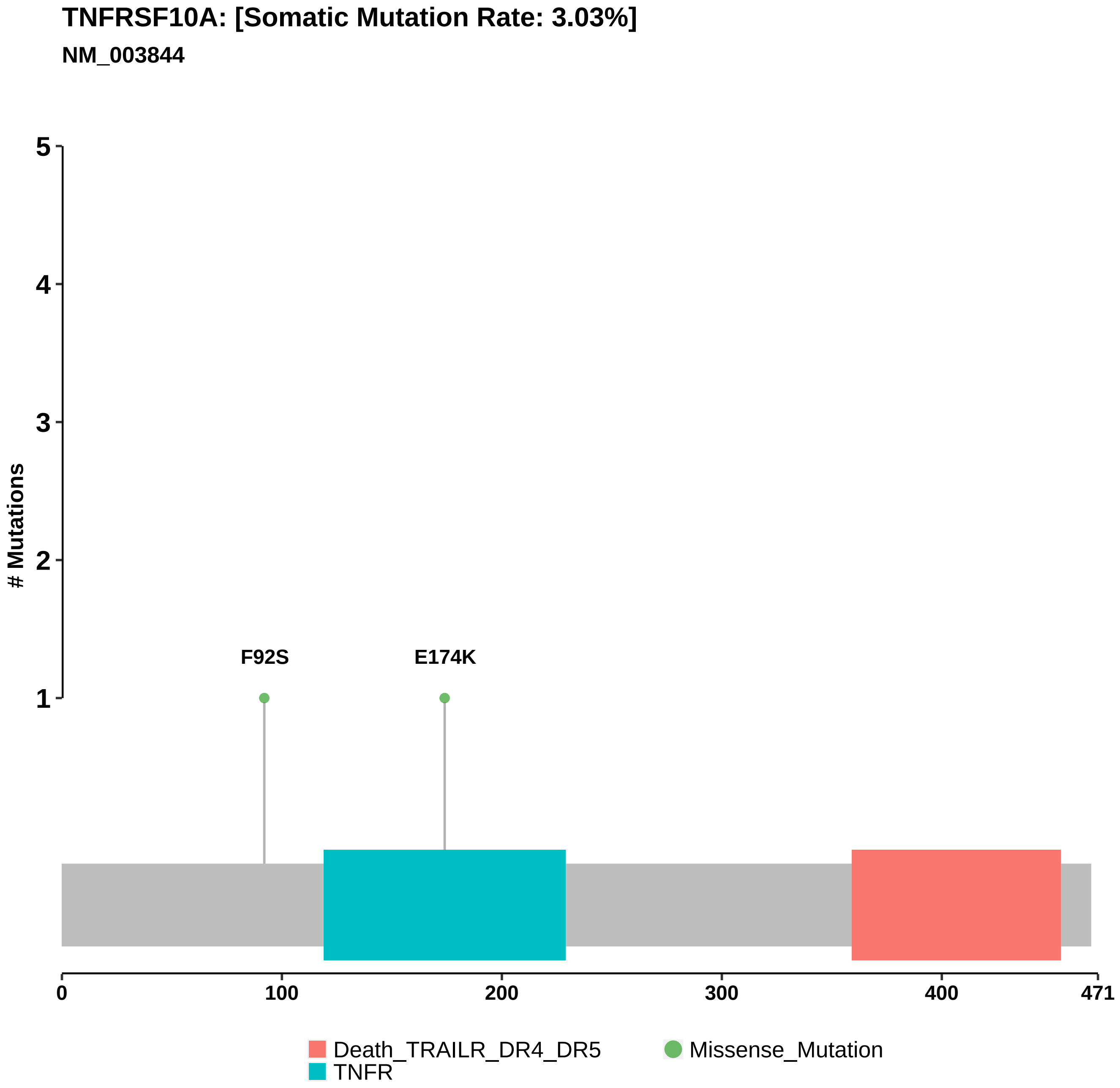

TNFRSF10A: [Somatic Mutation Rate: 3.03%]
NM_003844
Mutations
5
4
3
2
1
F92S
E174K
0
100
200
300
400
471
Death_TRAILR_DR4_DR5
TNFR
Missense_Mutation

TNFSF10: [Somatic Mutation Rate: 3.03%]

NM_003810

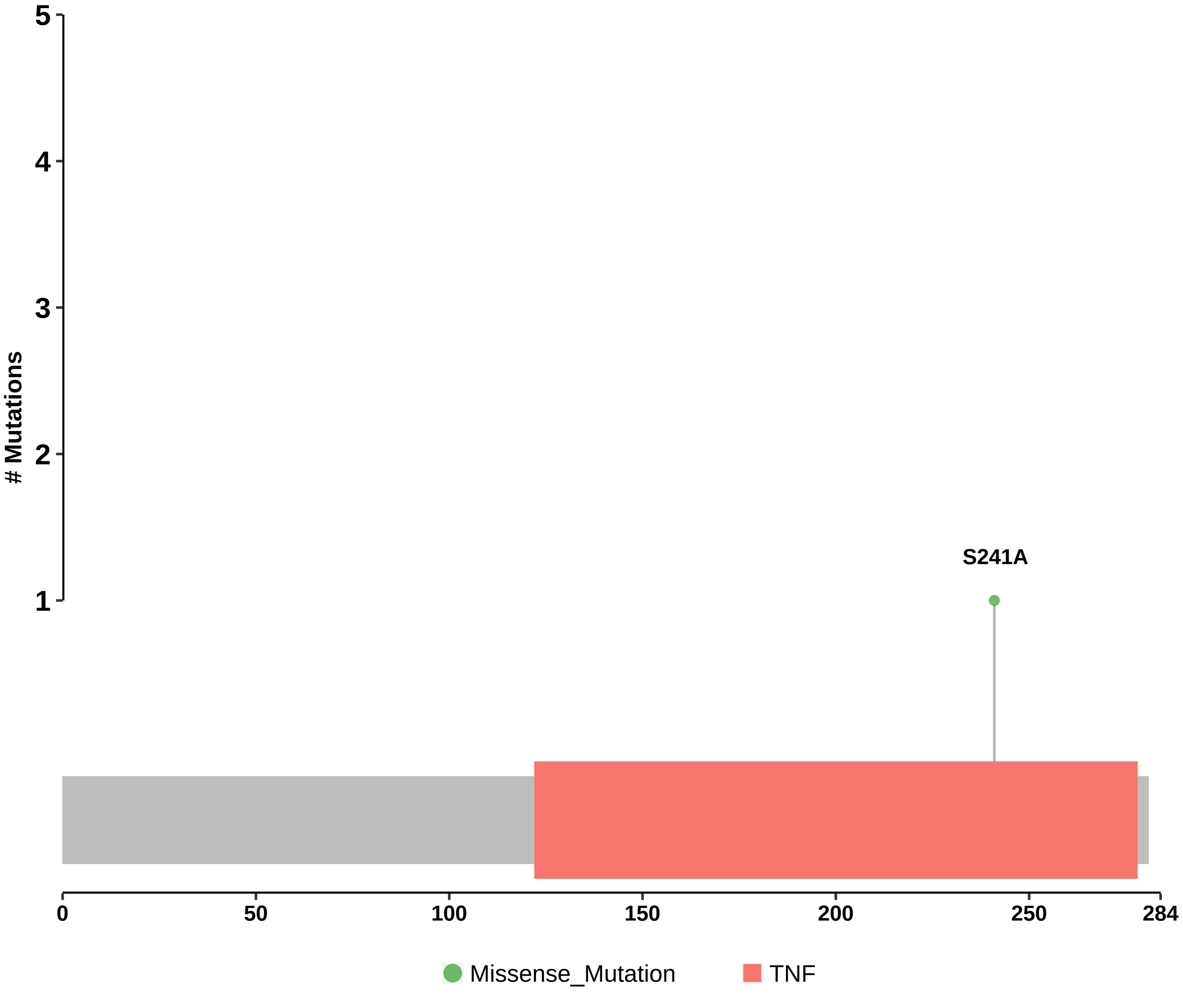

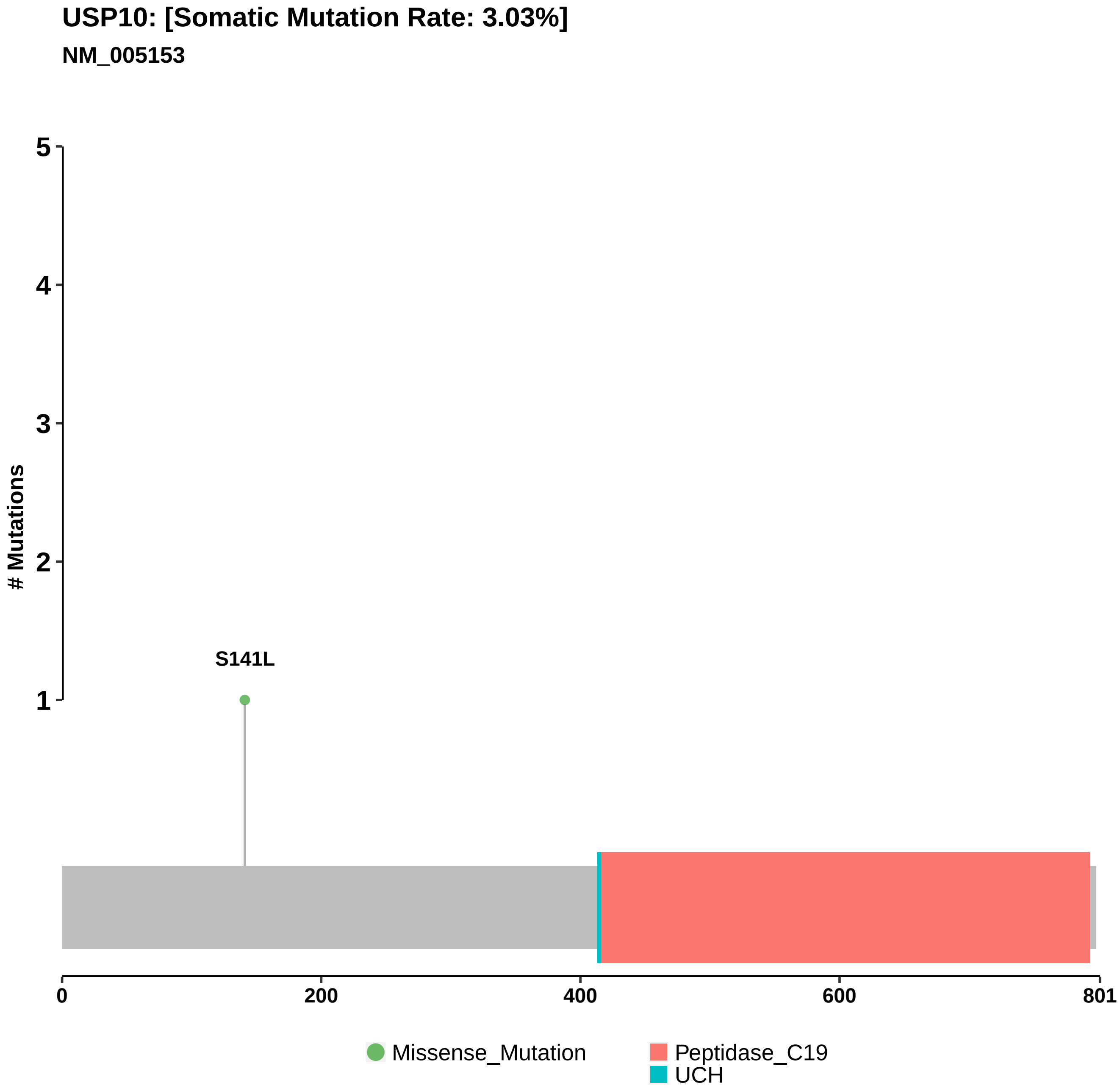

USP10: [Somatic Mutation Rate: 3.03%]
NM_005153
Mutations
5
4
3
2
1
S141L
0
200
400
600
801
Missense_Mutation
Peptidase_C19
UCH

XBP1: [Somatic Mutation Rate: 3.03%]

NM_001079539

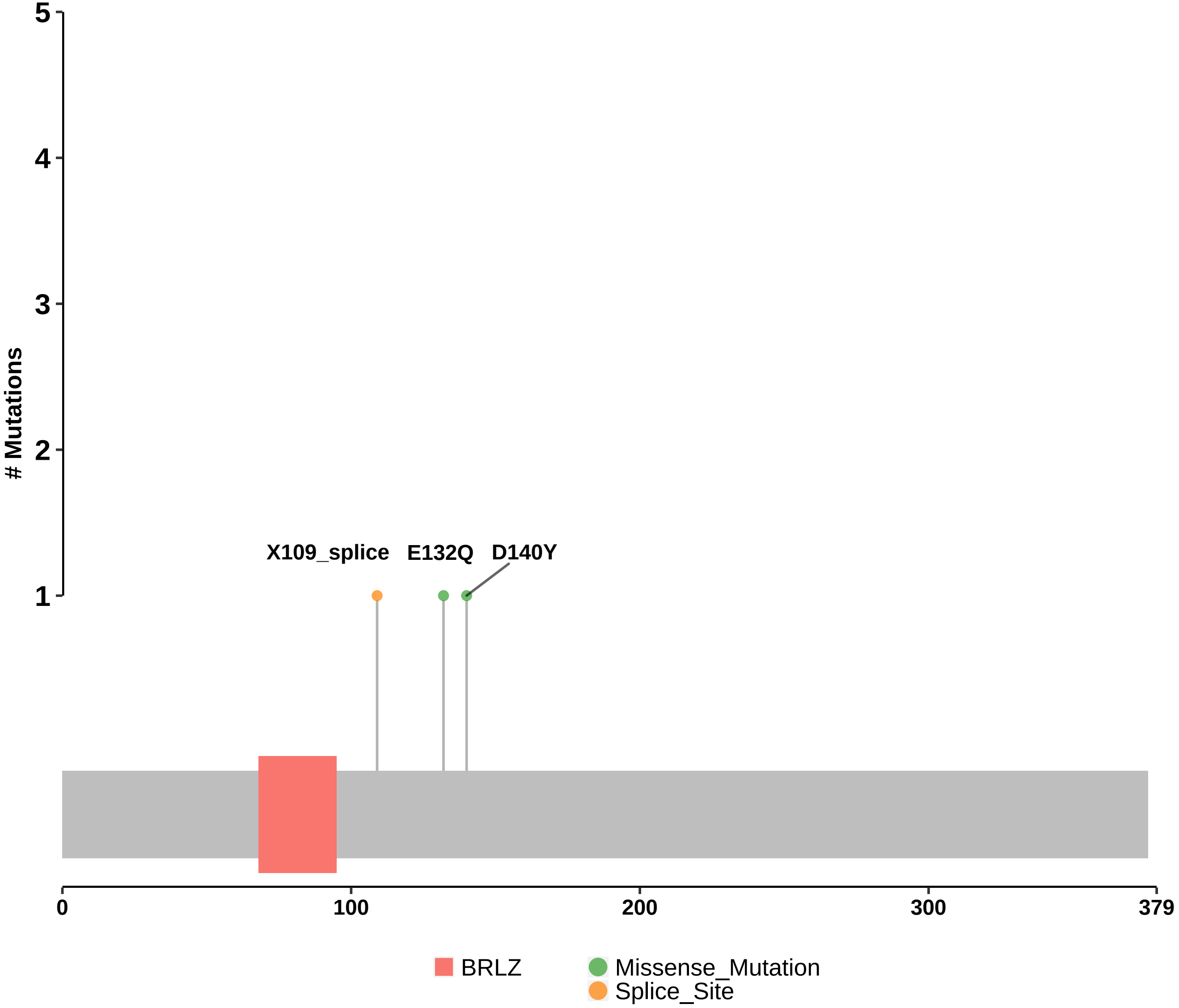

XRCC1: [Somatic Mutation Rate: 3.03%]

NM_006297

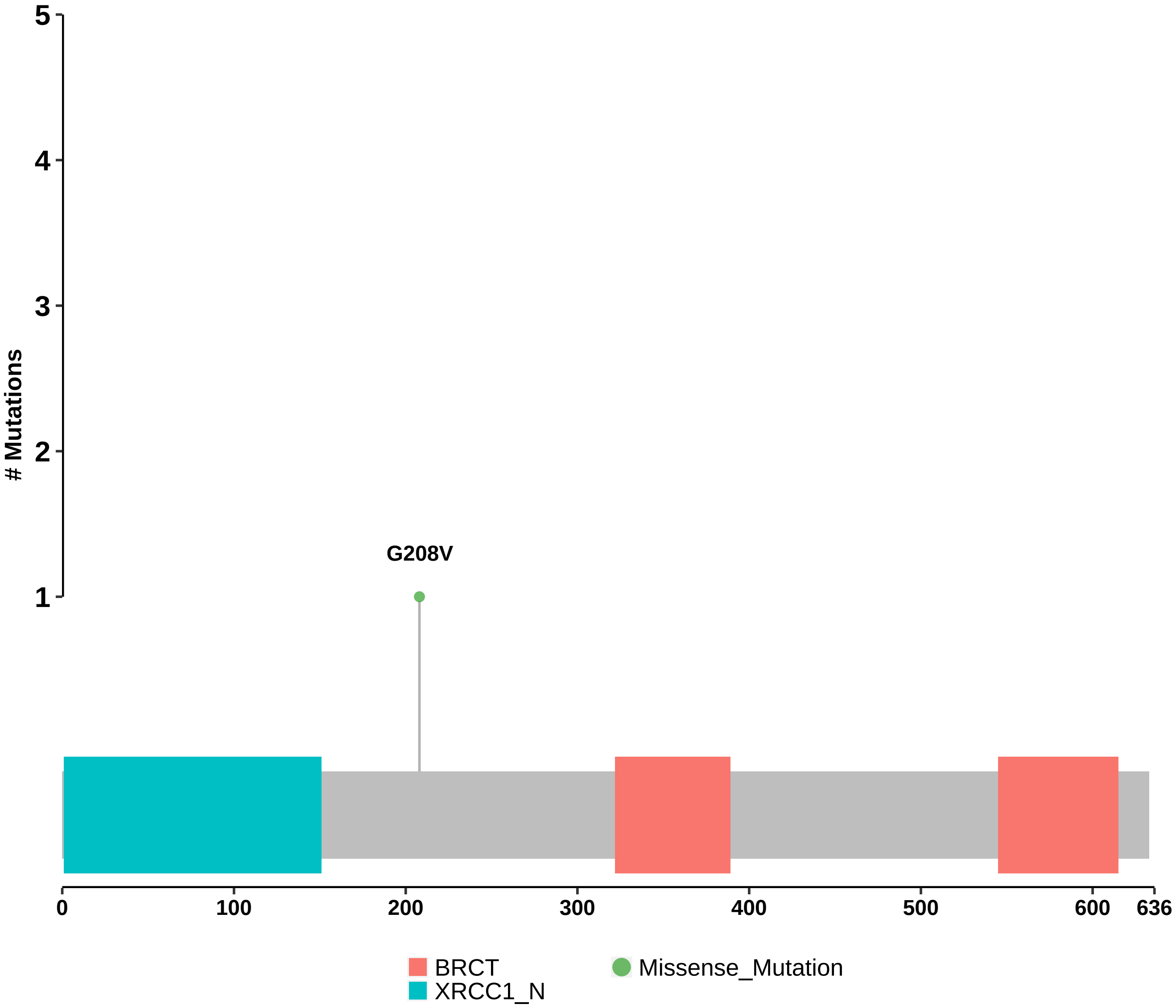

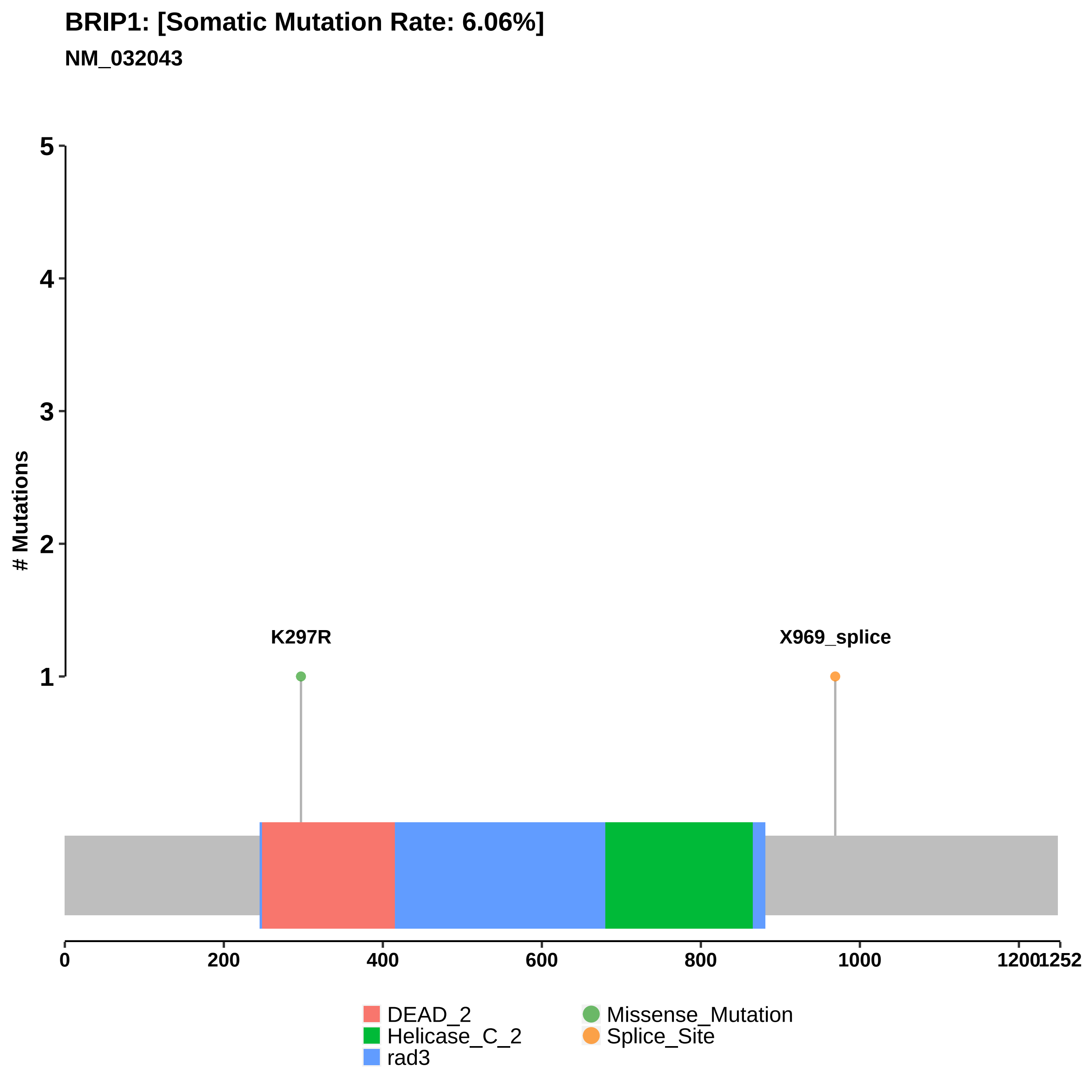

BRIP1: [Somatic Mutation Rate: 6.06%]
NM_032043
Mutations
5
4
3
2
1
K297R
X969_splice
0
200
400
600
800
1000
1200
1252
DEAD_2
Helicase_C_2
rad3
Missense_Mutation
Splice_Site

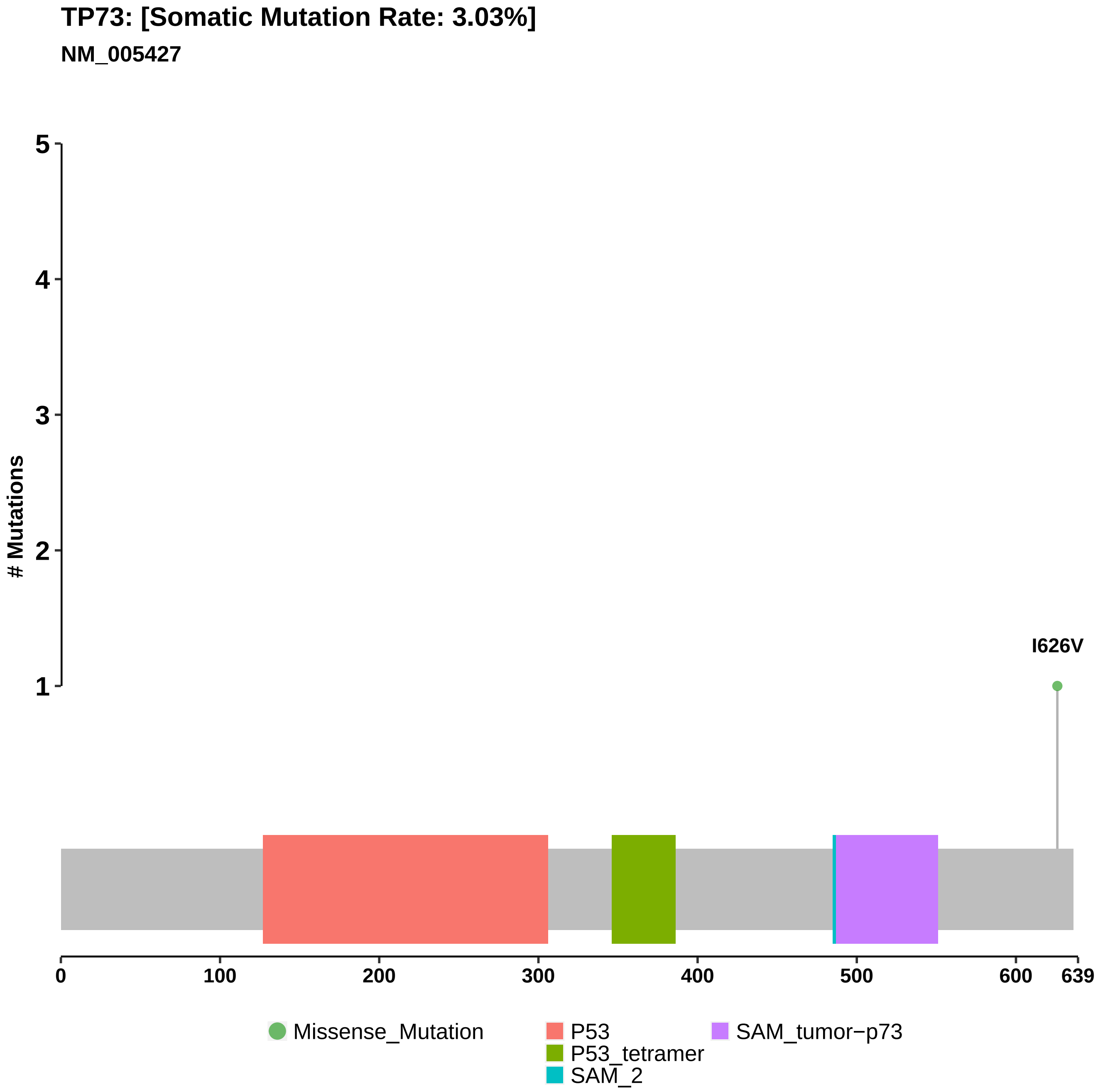
TP73: [Somatic Mutation Rate: 3.03%]
NM_005427
Mutations
5
4
3
2
1
I626V
0
100
200
300
400
500
600
639
Missense_Mutation
P53
P53_tetramer
SAM_2
SAM_tumor−p73

RECQL4: [Somatic Mutation Rate: 15.15%]

NM_004260

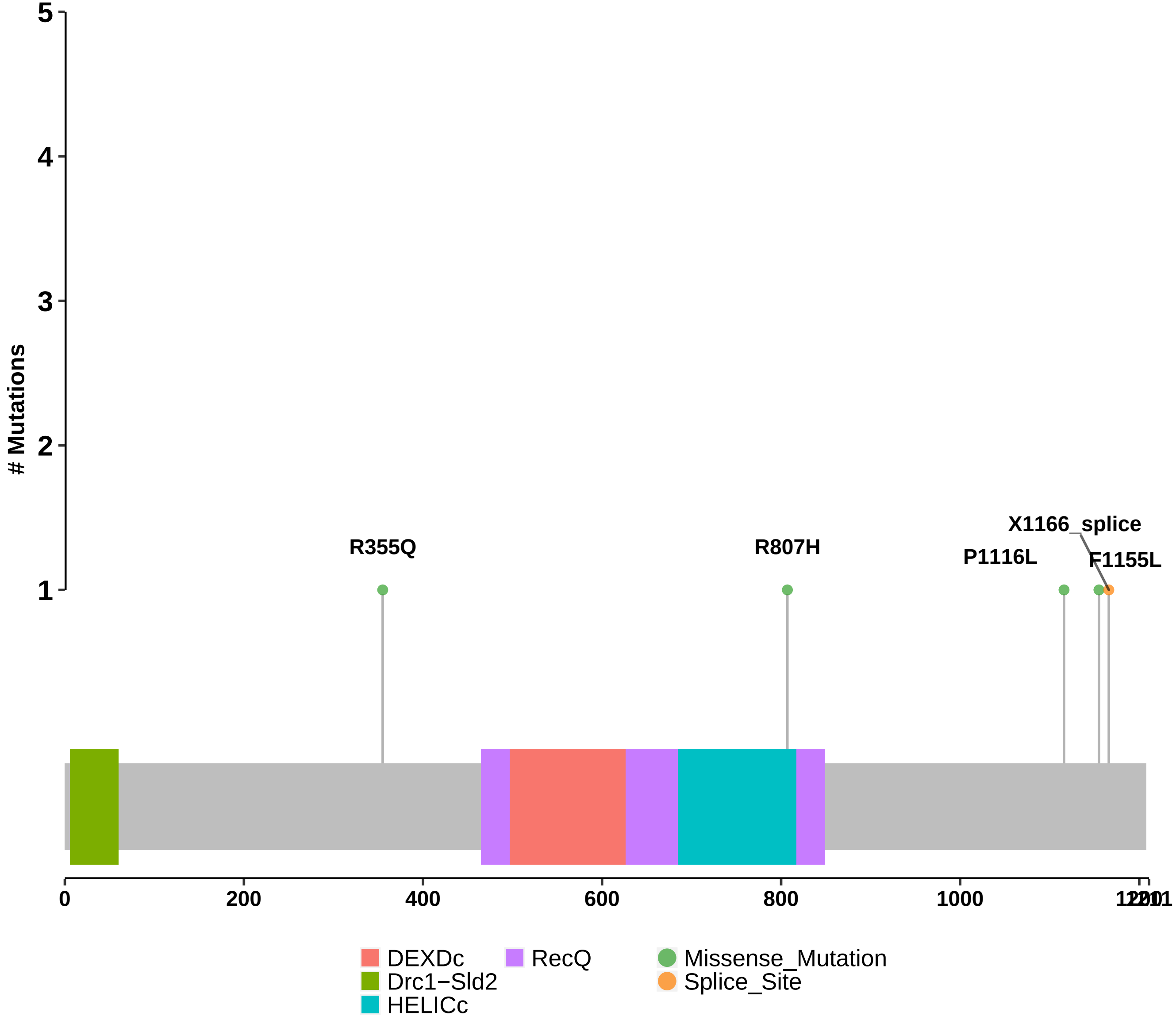

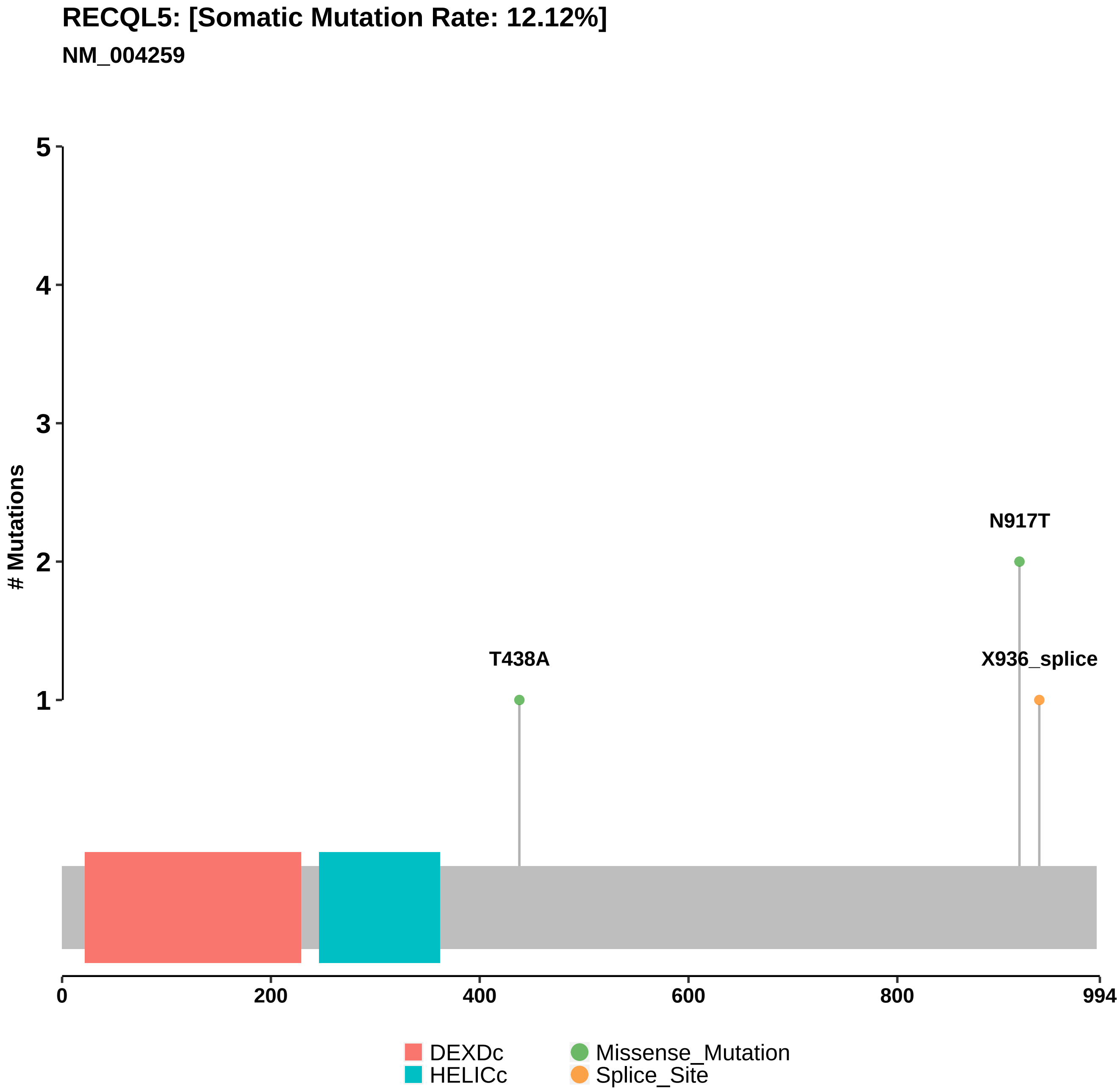
RECQL5: [Somatic Mutation Rate: 12.12%]
NM_004259
Mutations
5
4
3
2
1
N917T
T438A
X936_splice
0
200
400
600
800
994
DEXDc
HELICc
Missense_Mutation
Splice_Site

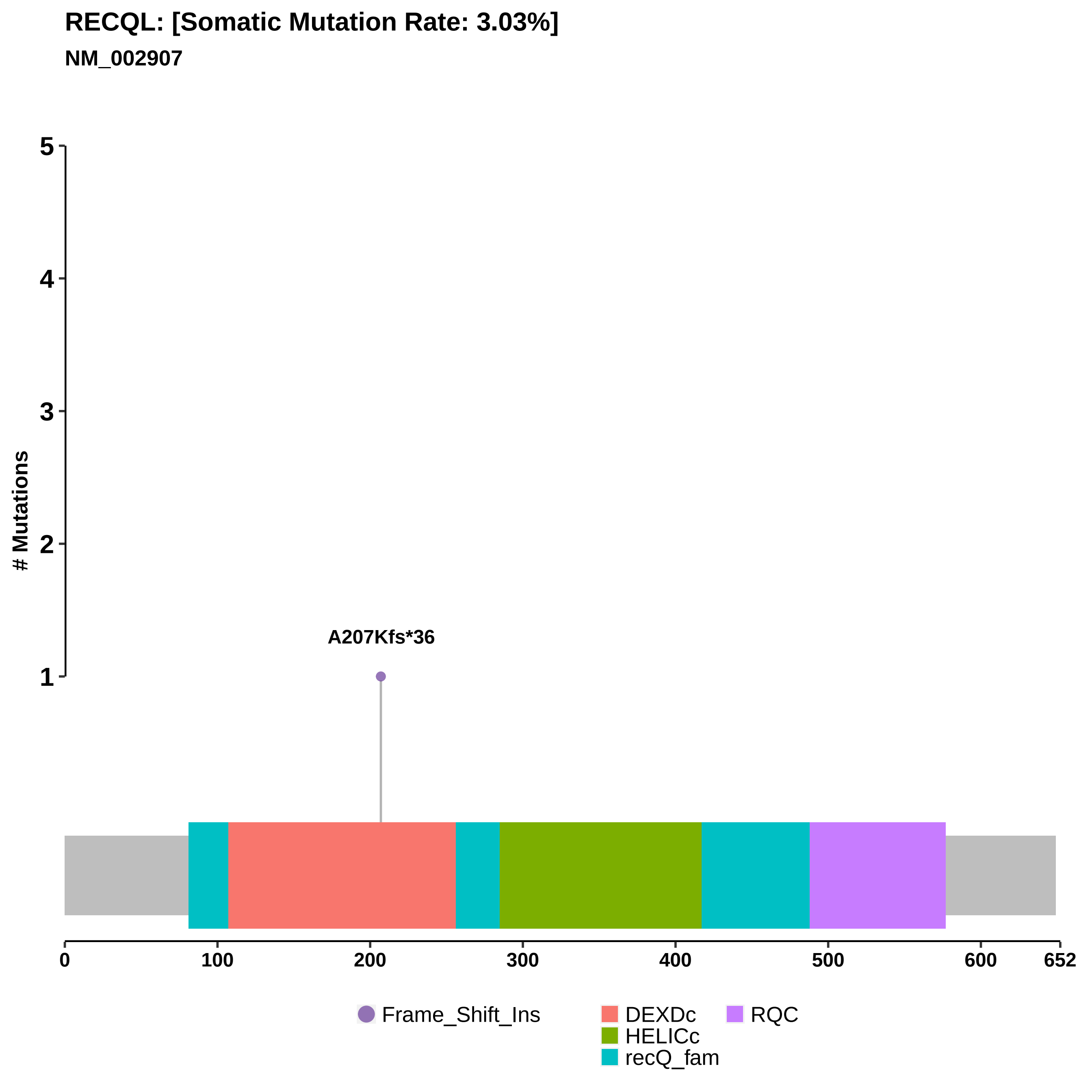
RECQL: [Somatic Mutation Rate: 3.03%]
NM_002907
Mutations
5
4
3
2
1
A207Kfs*36
0
100
200
300
400
500
600
652
Frame_Shift_Ins
DEXDc
HELICc
recQ_fam
RQC

BLM: [Somatic Mutation Rate: 15.15%]

NM_000057

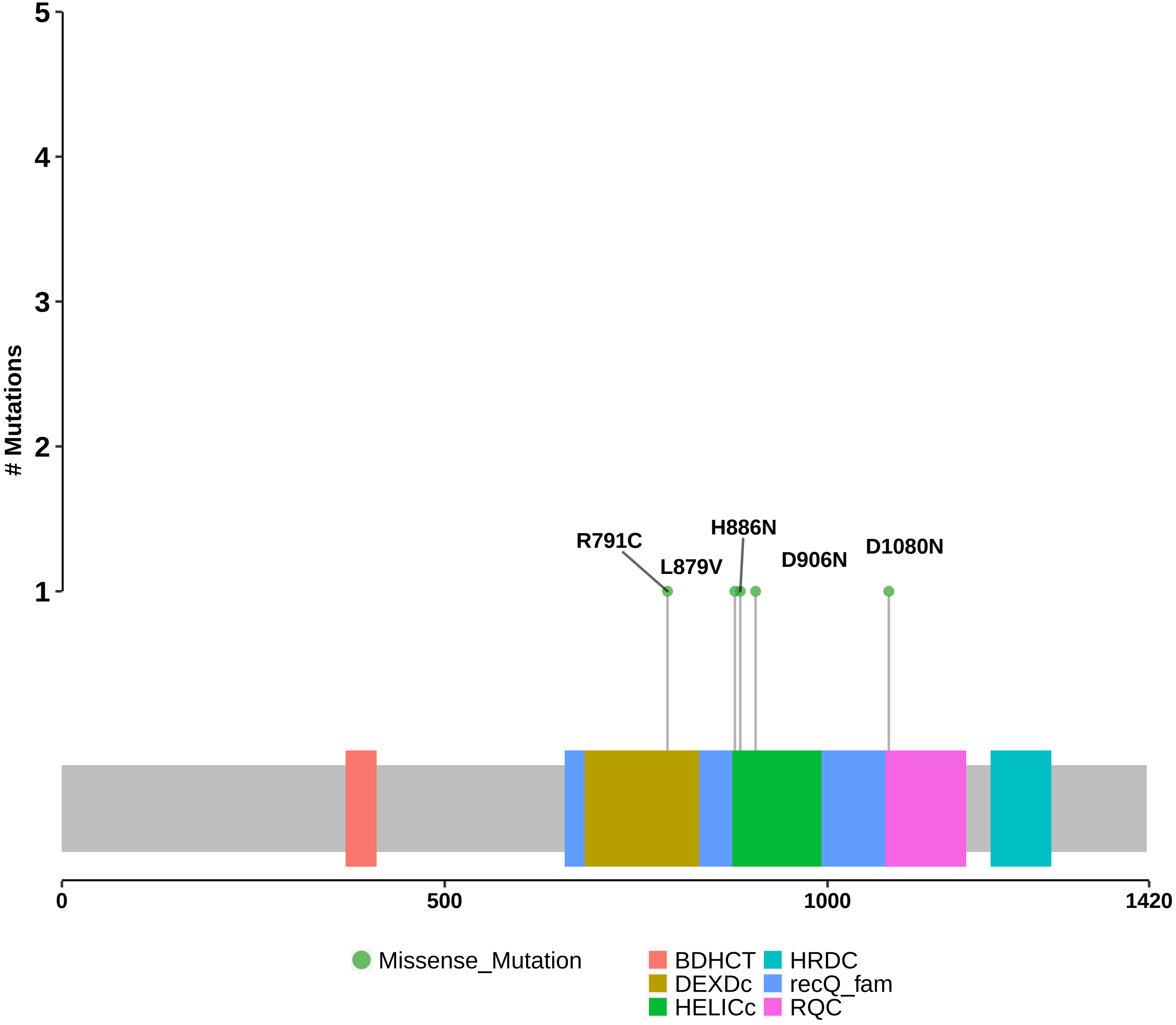

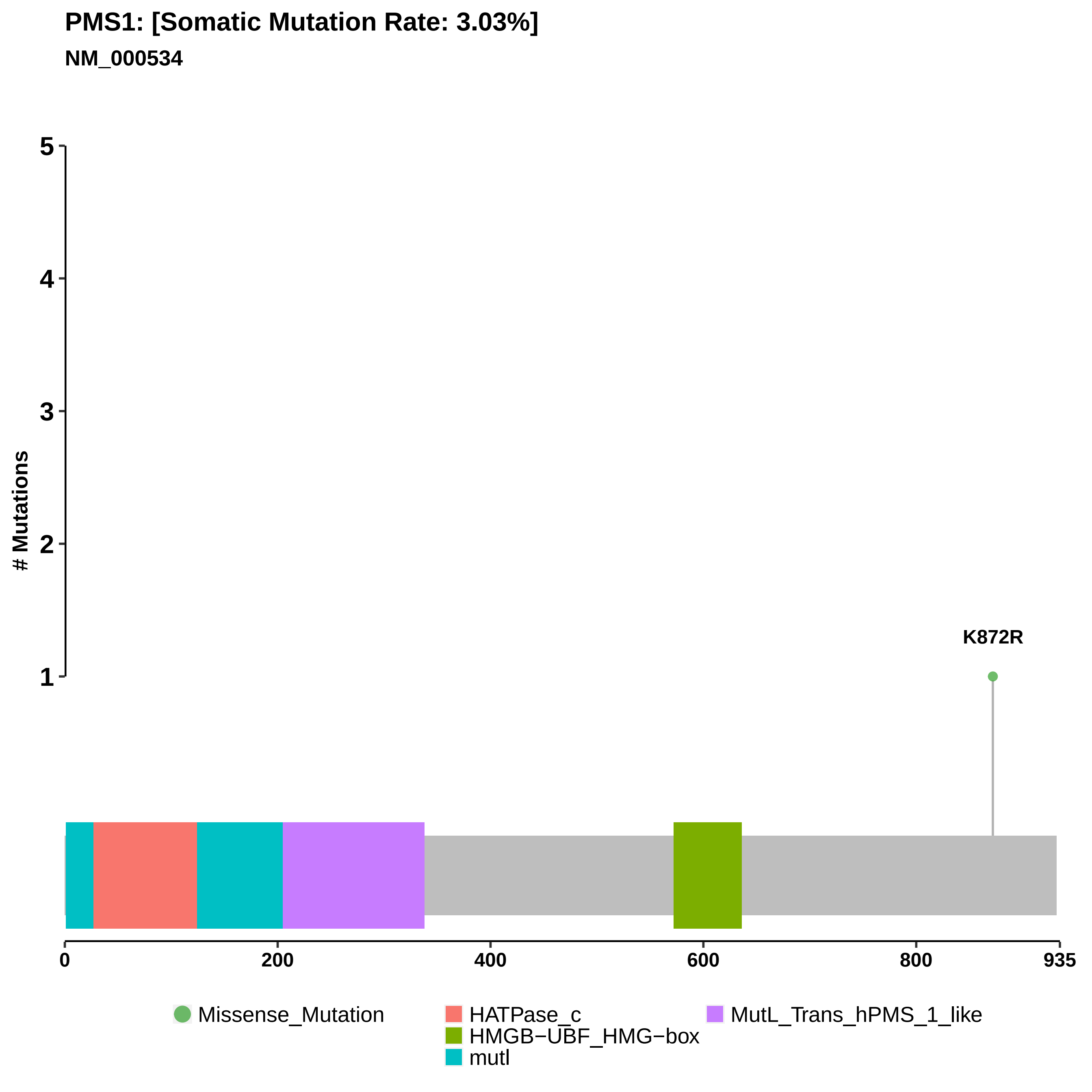
PMS1: [Somatic Mutation Rate: 3.03%]
NM_000534
Mutations
5
4
3
2
1
K872R
0
200
400
600
800
935
Missense_Mutation
HATPase_c
HMGB−UBF_HMG−box
mutl
MutL_Trans_hPMS_1_like

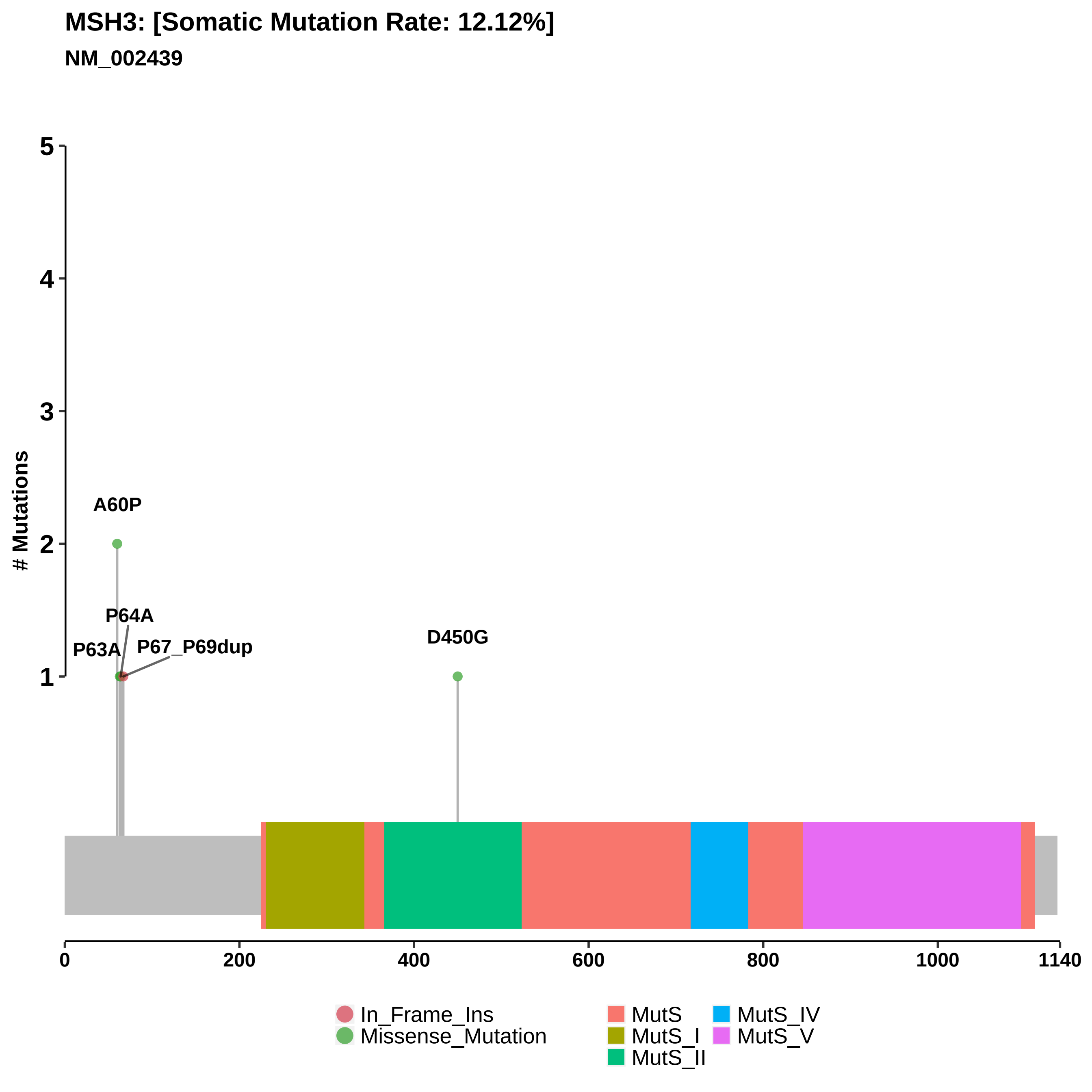
MSH3: [Somatic Mutation Rate: 12.12%]
NM_002439
Mutations
5
4
3
2
1
A60P
P64A
P63A
P67_P69dup
D450G
0
200
400
600
800
1000
1140
In_Frame_Ins
Missense_Mutation
MutS
MutS_I
MutS_II
MutS_IV
MutS_V

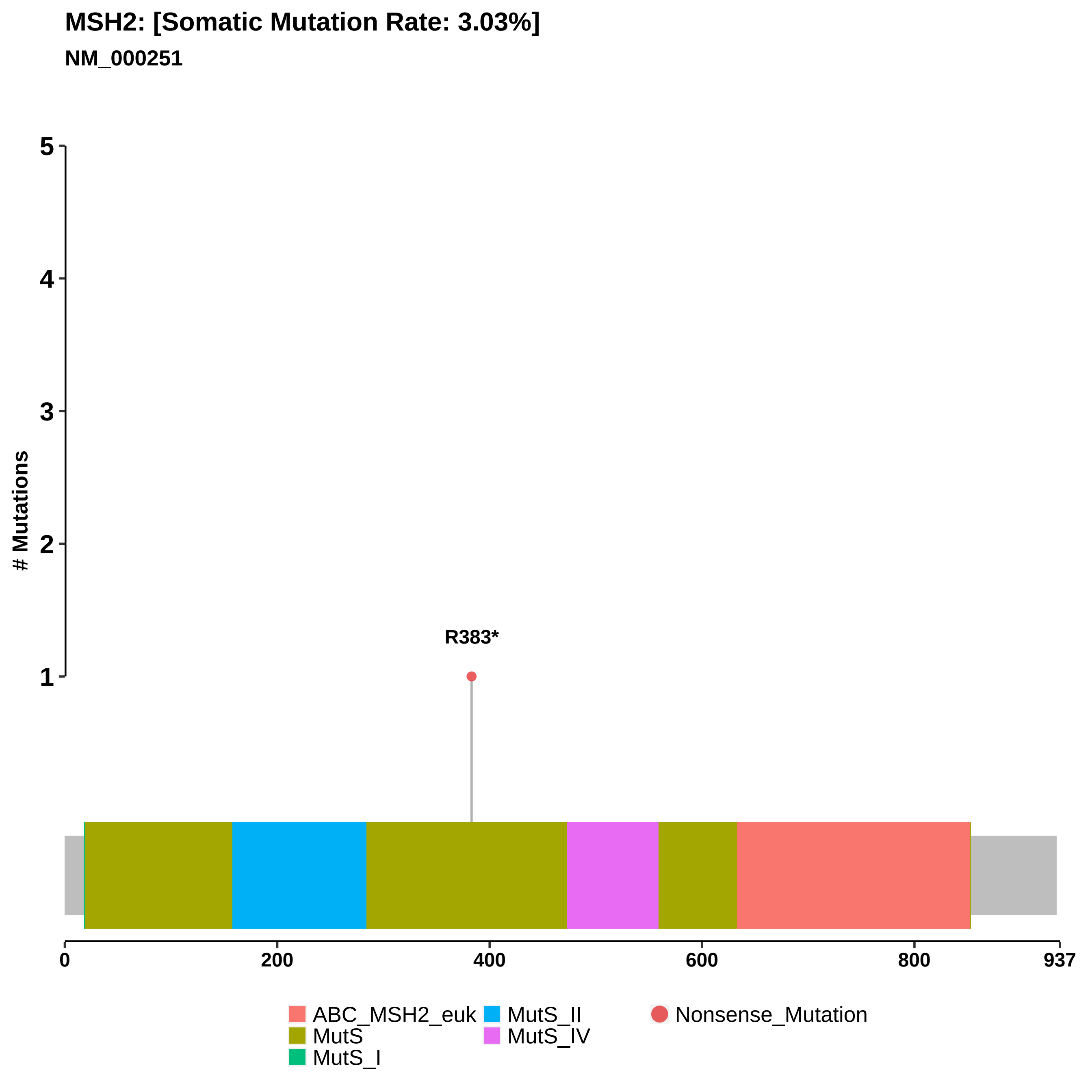
MSH2: [Somatic Mutation Rate: 3.03%]
NM_000251
Mutations
5
4
3
2
1
R383*
0
200
400
600
800
937
ABC_MSH2_euk
MutS
MutS_I
MutS_II
MutS_IV
Nonsense_Mutation

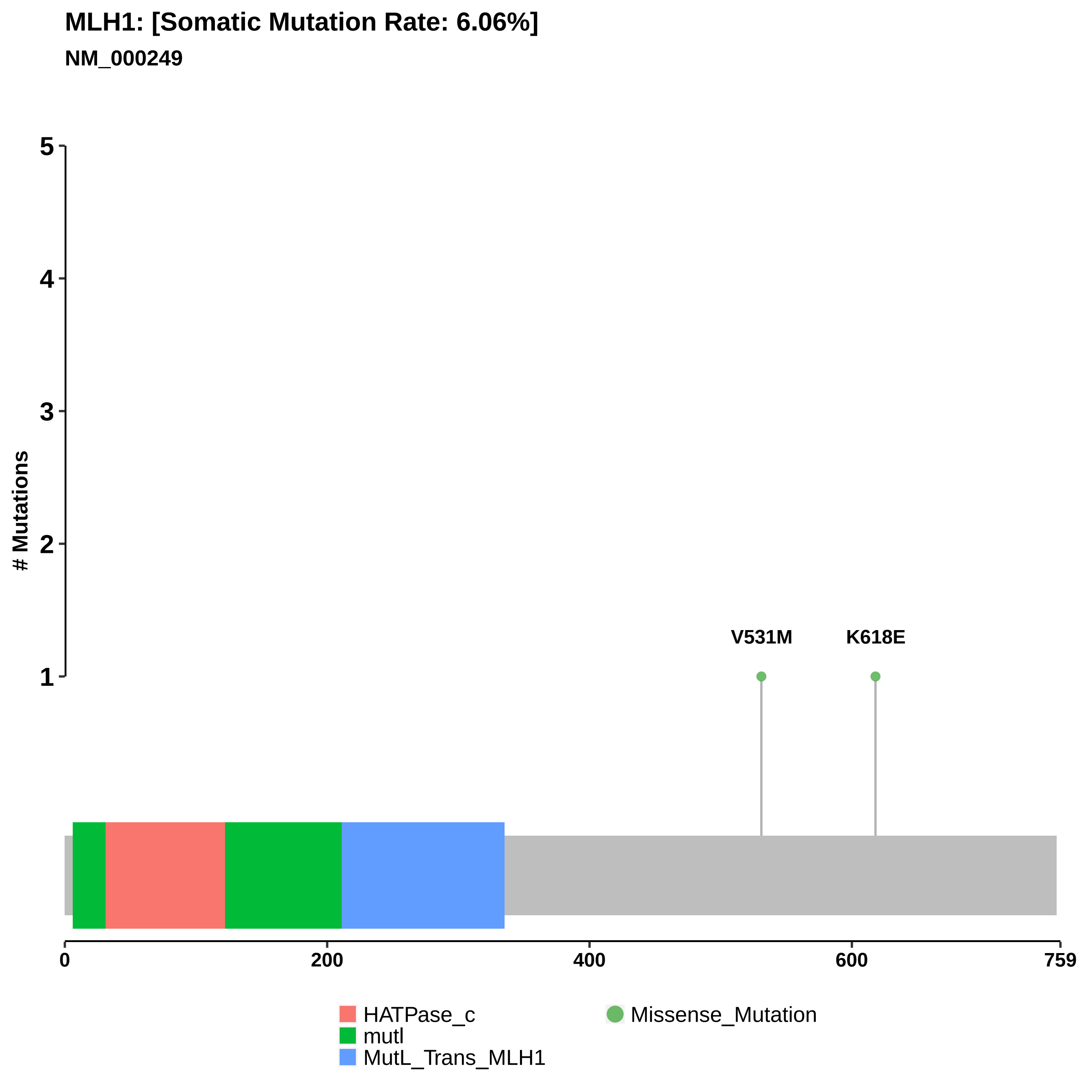
MLH1: [Somatic Mutation Rate: 6.06%]
NM_000249
Mutations
5
4
3
2
1
V531M
K618E
0
200
400
600
759
HATPase_c
mutl
MutL_Trans_MLH1
Missense_Mutation

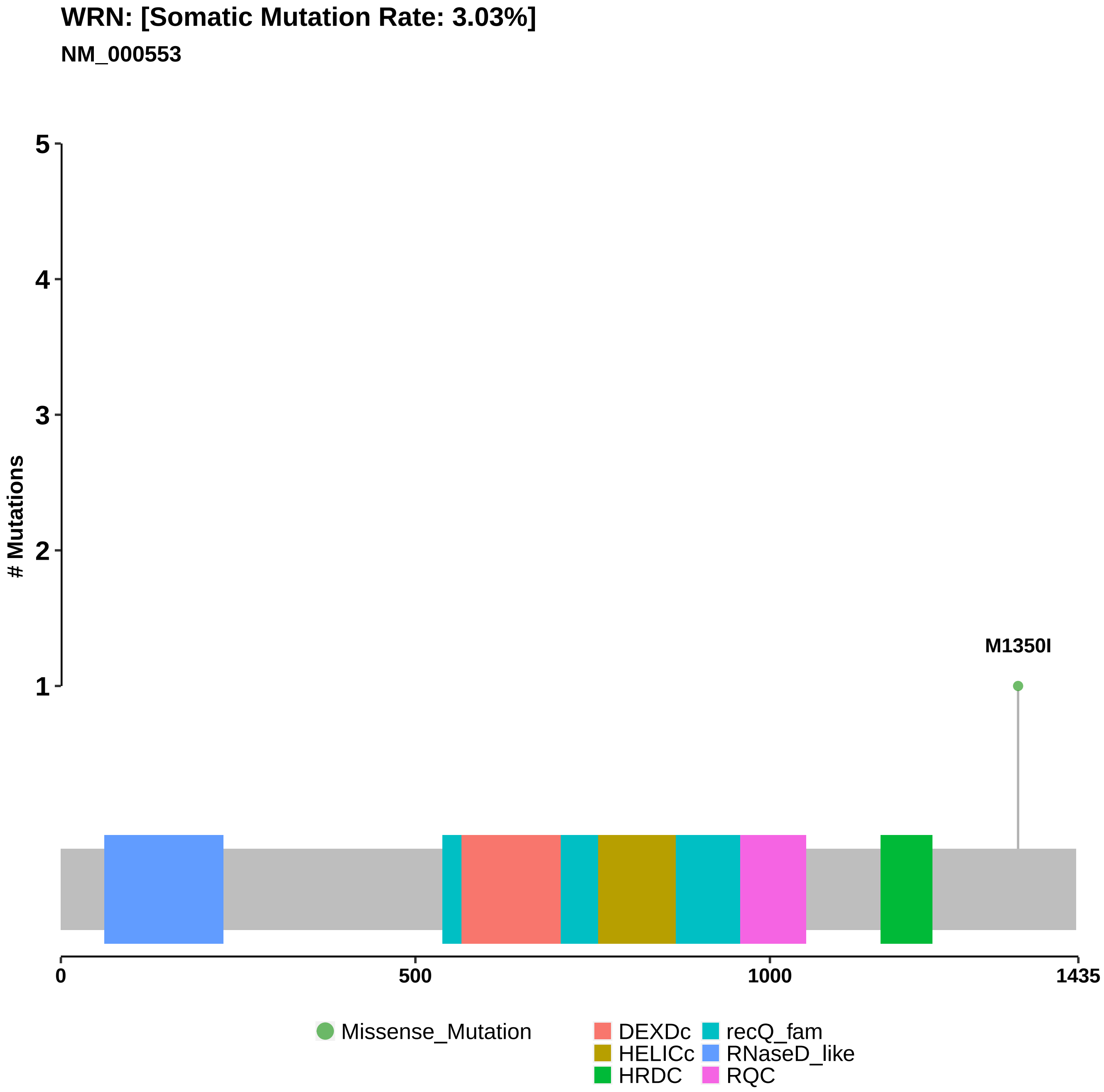
WRN: [Somatic Mutation Rate: 3.03%]
NM_000553
Mutations
5
4
3
2
1
M1350I
0
500
1000
1435
Missense_Mutation
DEXDc
HELICc
HRDC
recQ_fam
RNaseD_like
RQC

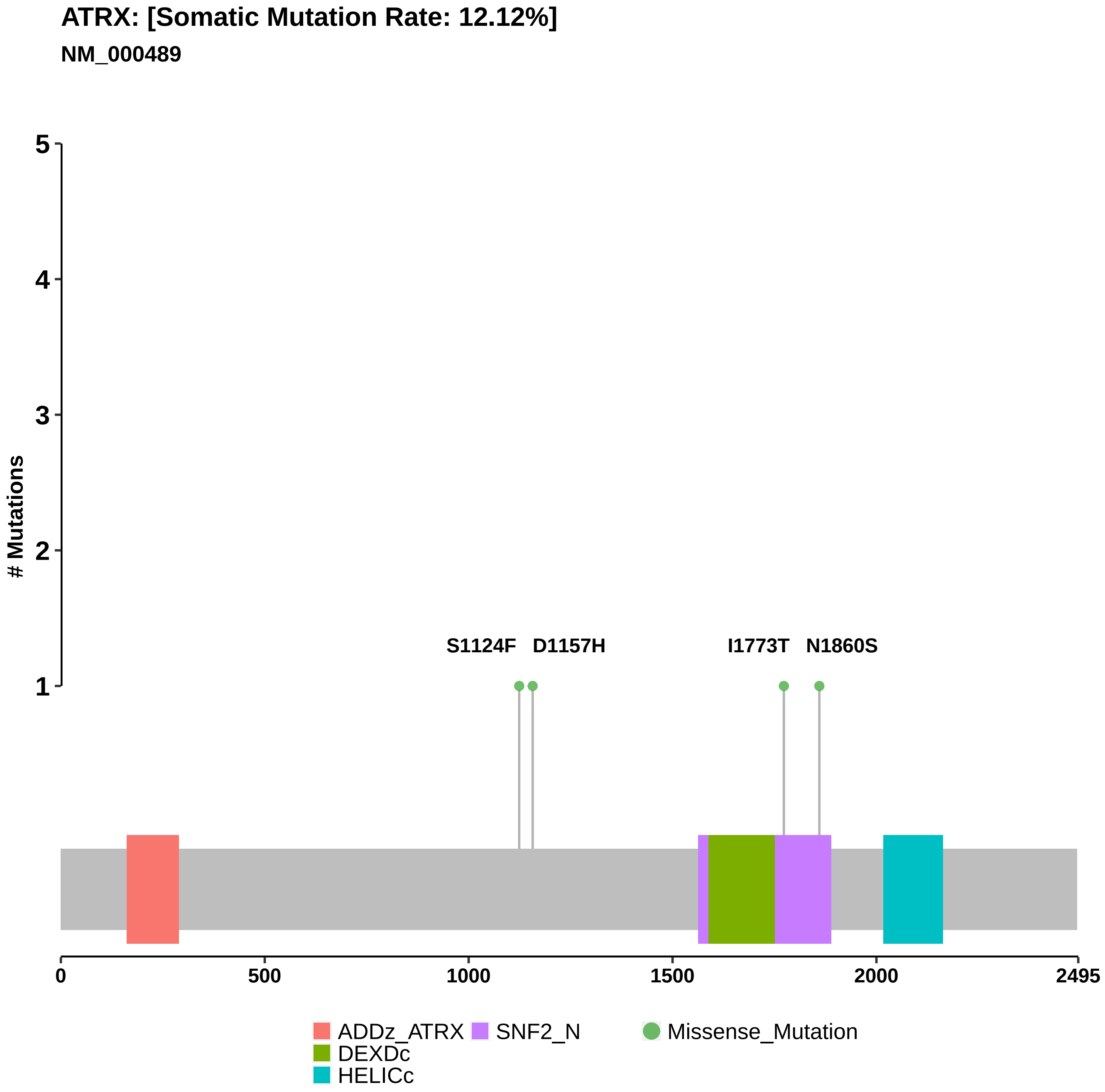

ATRX: [Somatic Mutation Rate: 12.12%]
NM_000489
Mutations
5
4
3
2
1
S1124F
D1157H
I1773T
N1860S
0
500
1000
1500
2000
2495
ADDz_ATRX
DEXDc
HELICc
SNF2_N
Missense_Mutation

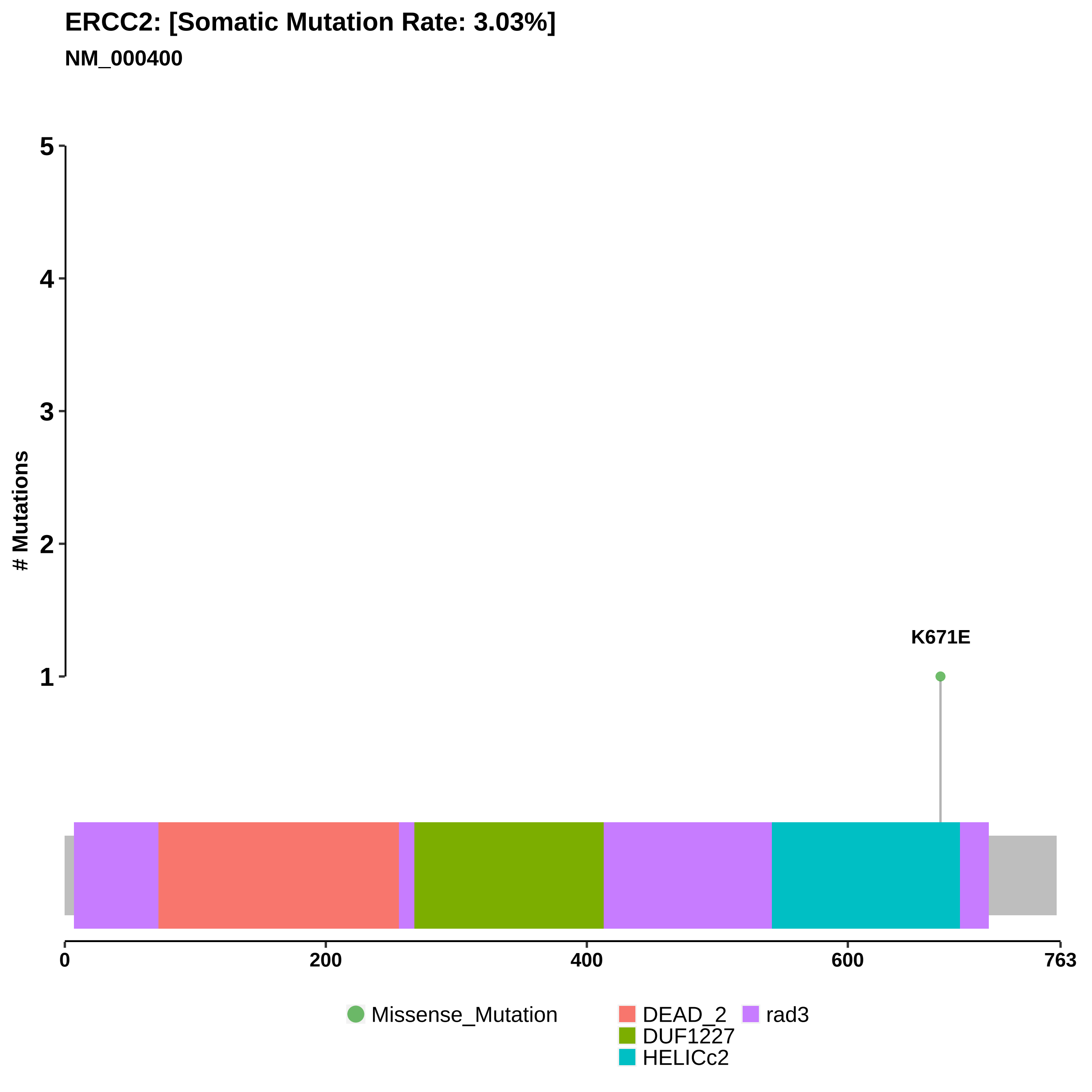
ERCC2: [Somatic Mutation Rate: 3.03%]
NM_000400
Mutations
5
4
3
2
1
K671E
0
200
400
600
763
Missense_Mutation
DEAD_2
DUF1227
HELICc2
rad3

CASP7: [Somatic Mutation Rate: 3.03%]
NM_001267057

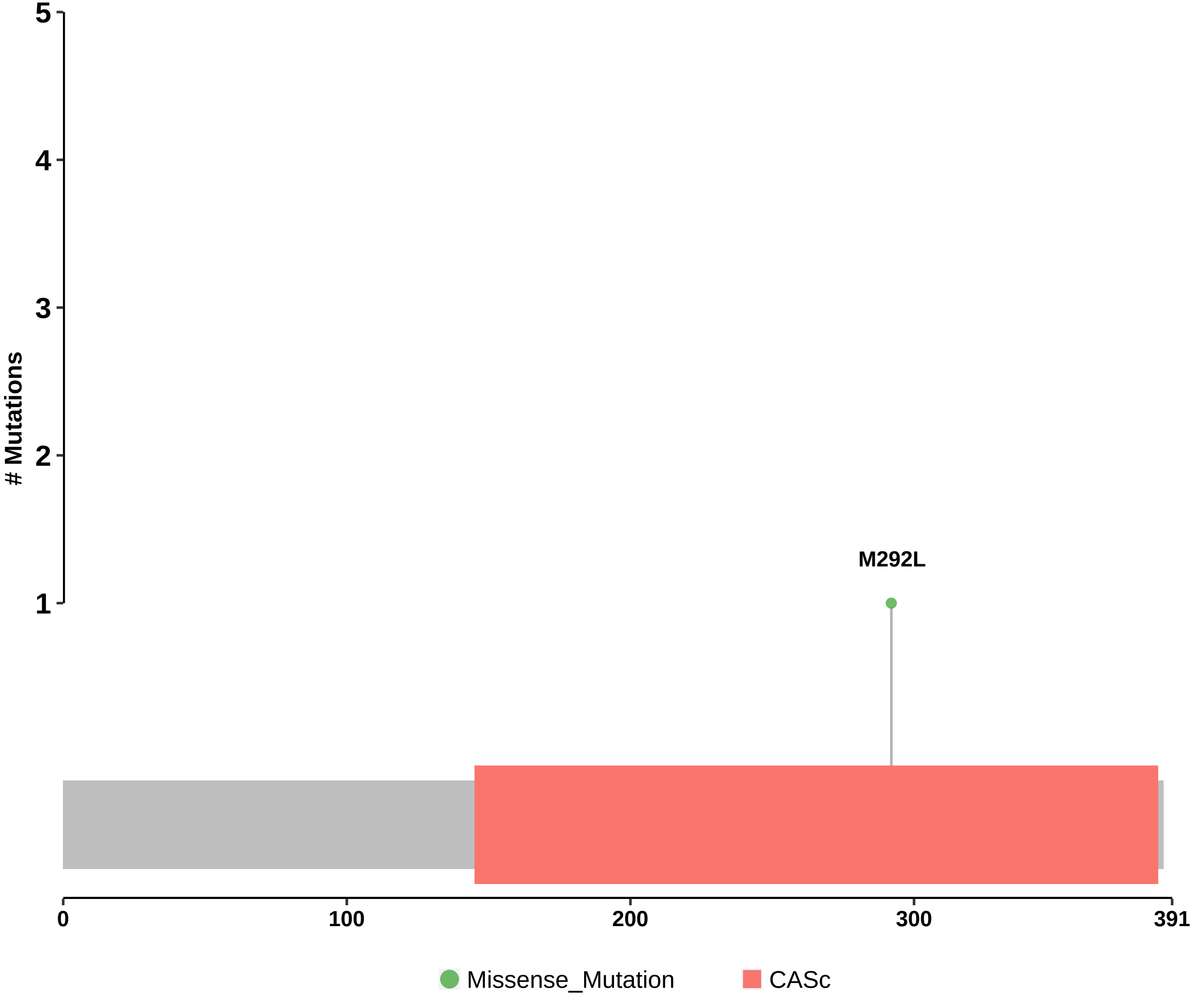

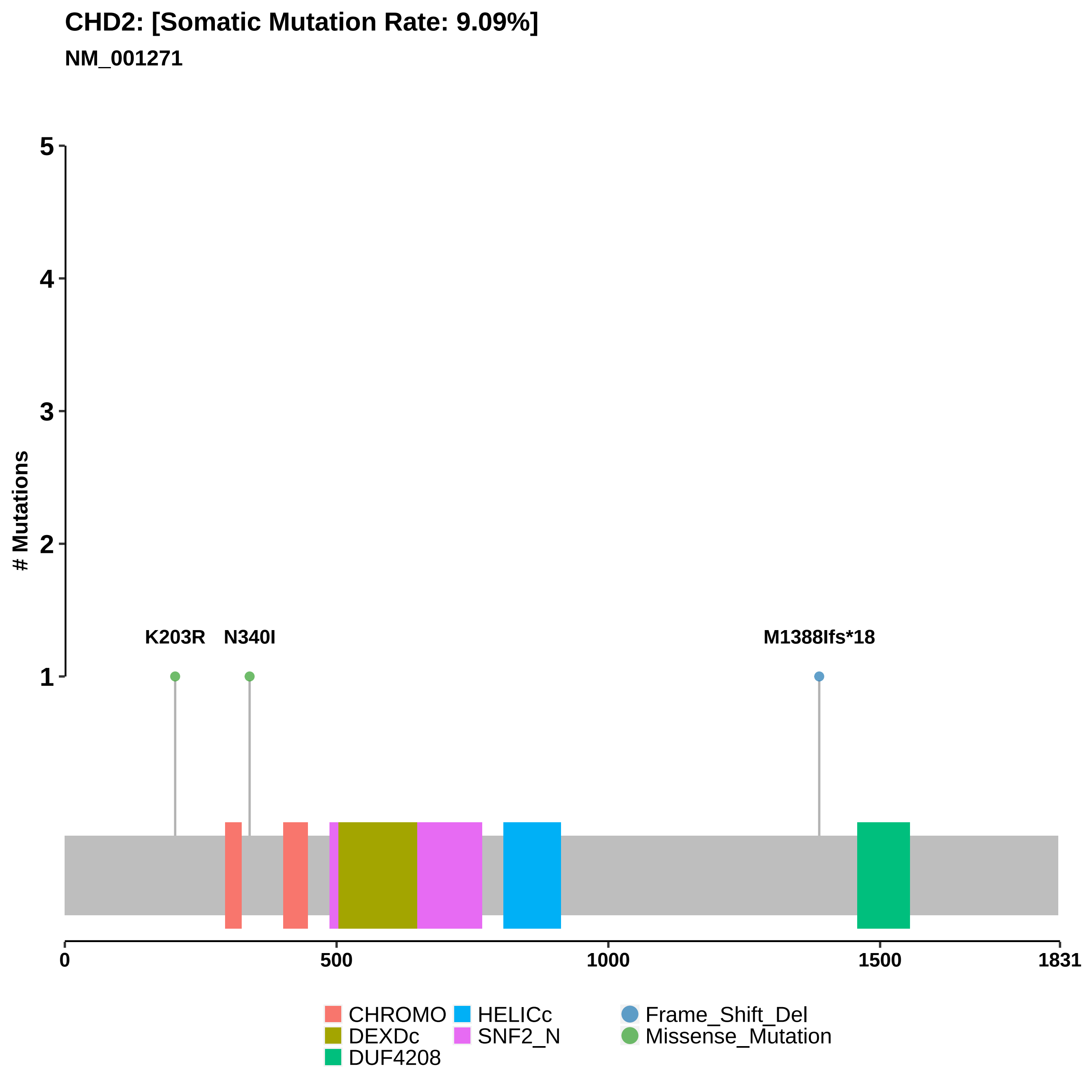
CHD2: [Somatic Mutation Rate: 9.09%]
NM_001271
Mutations
5
4
3
2
1
K203R
N340I
M1388Ifs*18
0
500
1000
1500
1831
CHROMO
DEXDc
DUF4208
HELICc
SNF2_N
Frame_Shift_Del
Missense_Mutation

TET2: [Somatic Mutation Rate: 12.12%]

NM_001127208

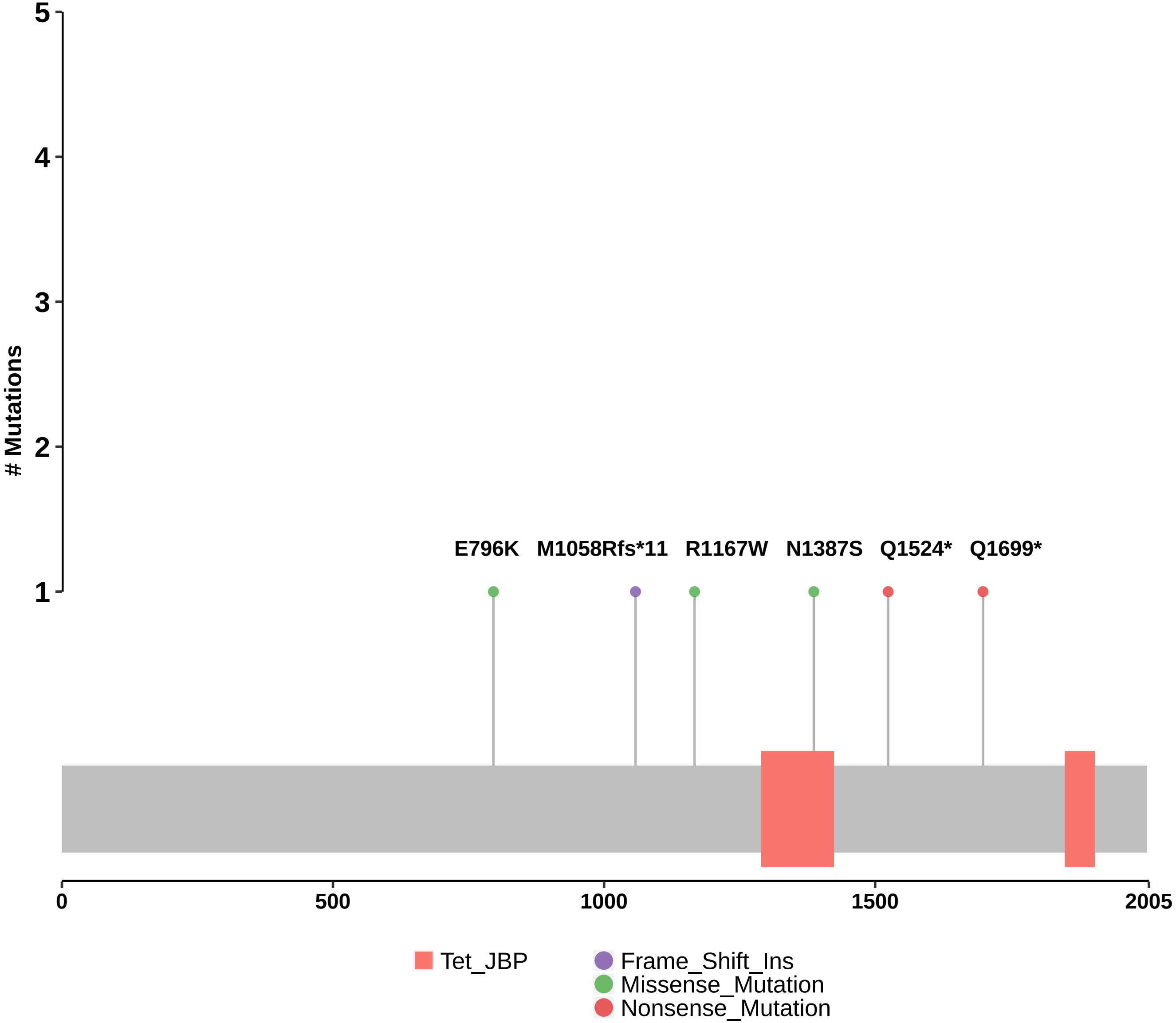

DOT1L: [Somatic Mutation Rate: 12.12%]

NM_032482

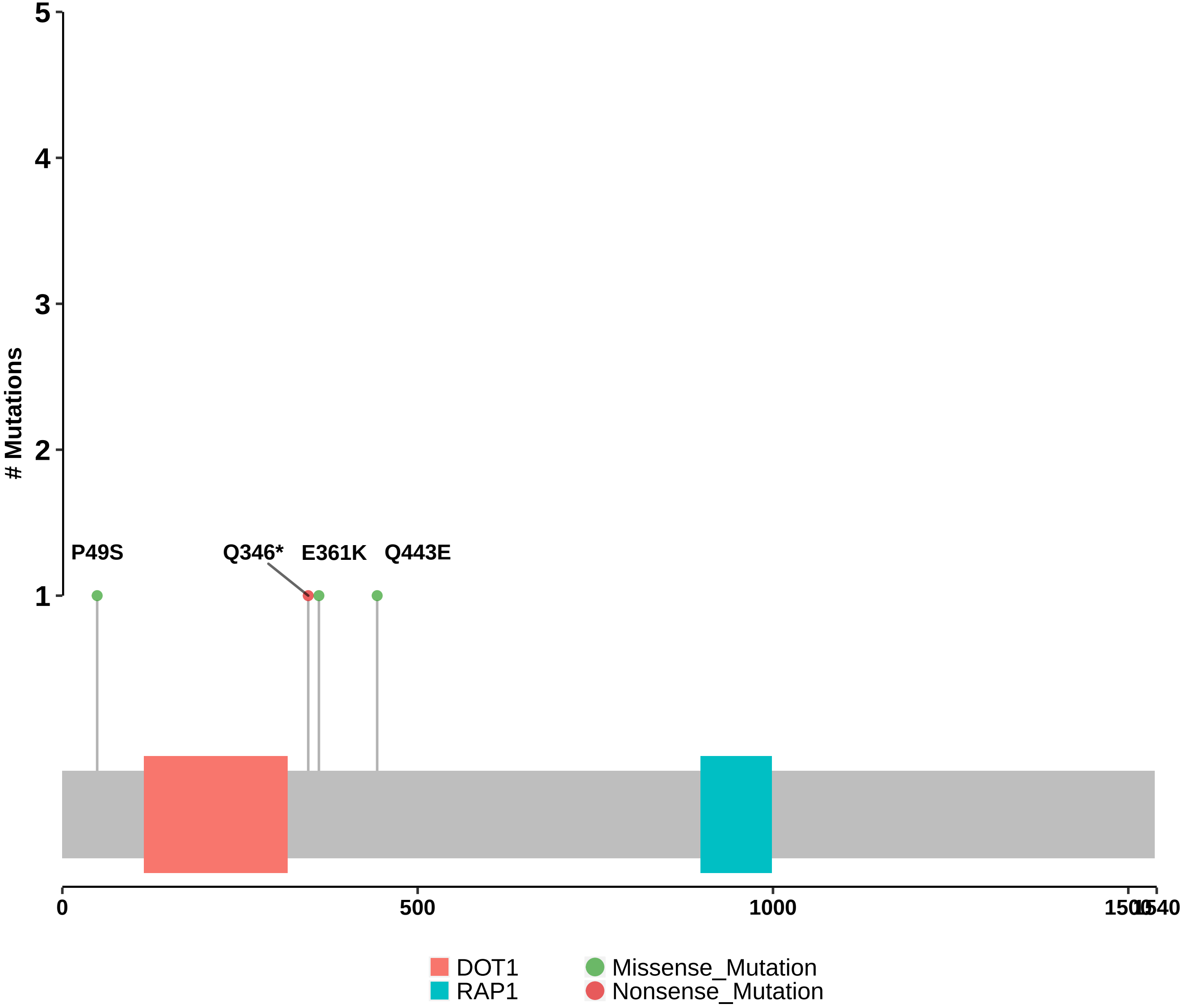

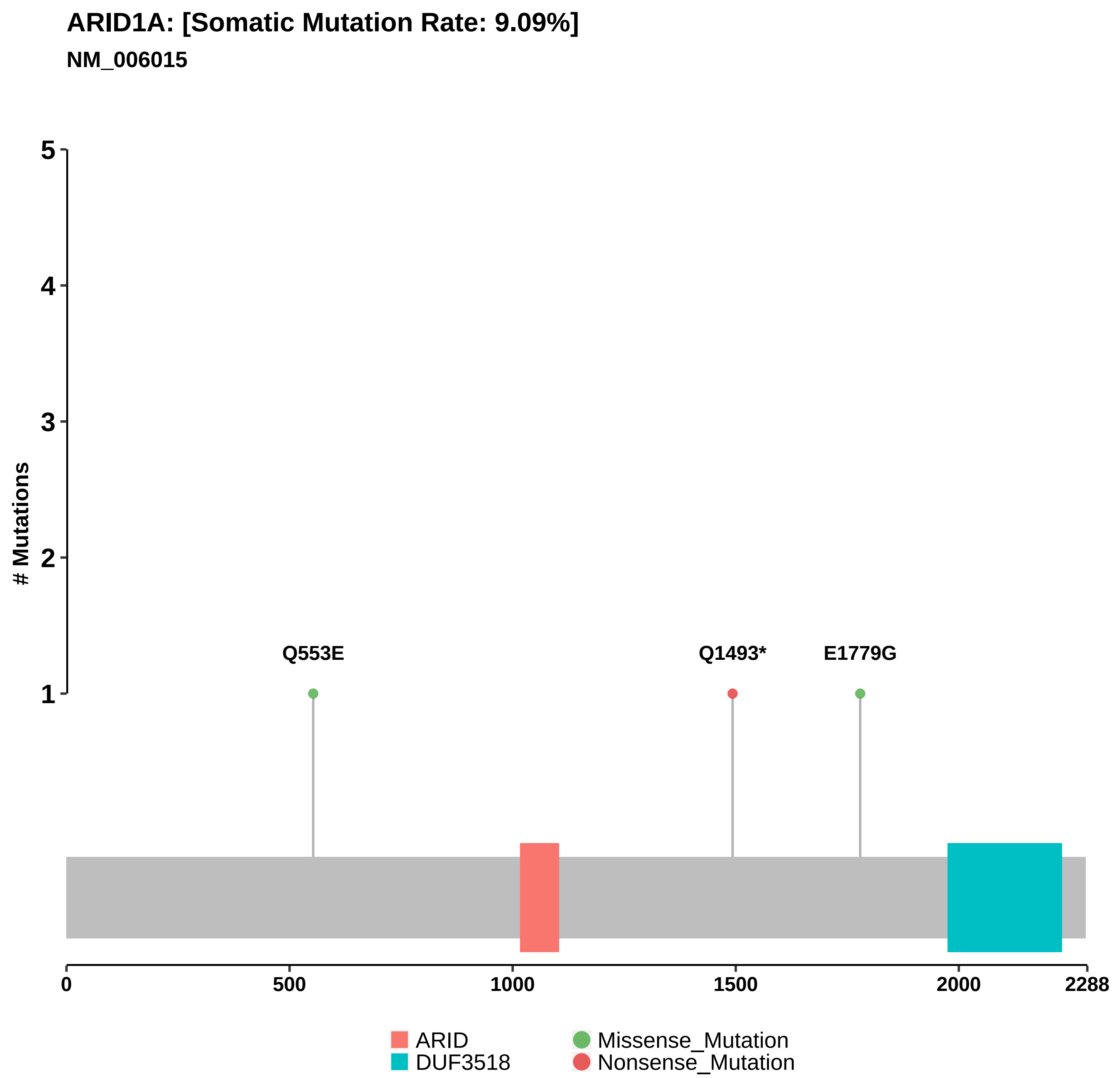

ARID1A: [Somatic Mutation Rate: 9.09%]
NM_006015
Mutations
5
4
3
2
1
Q553E
Q1493*
E1779G
0
500
1000
1500
2000
2288
ARID
DUF3518
Missense_Mutation
Nonsense_Mutation

DDX58: [Somatic Mutation Rate: 9.09%]

NM_014314

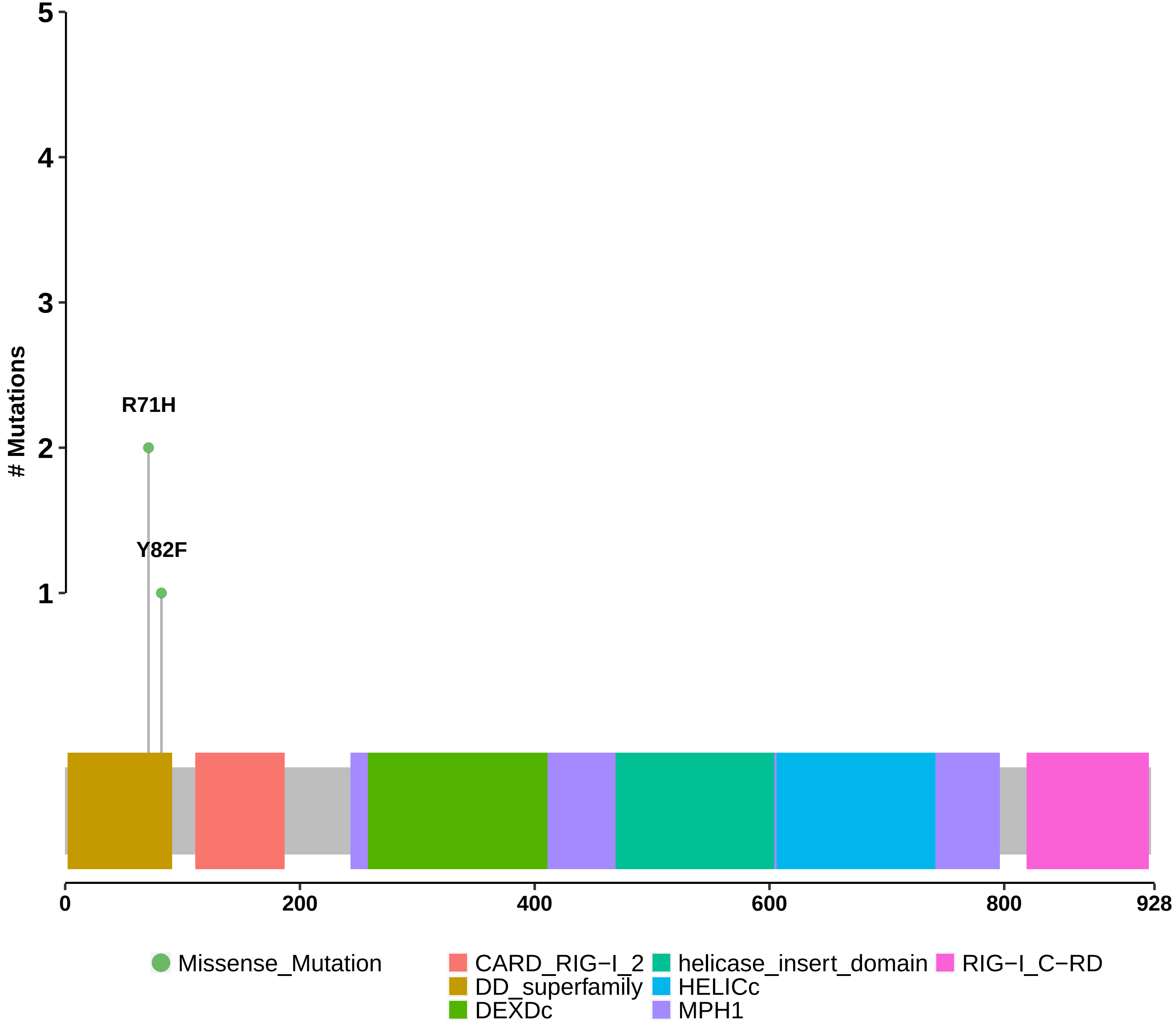

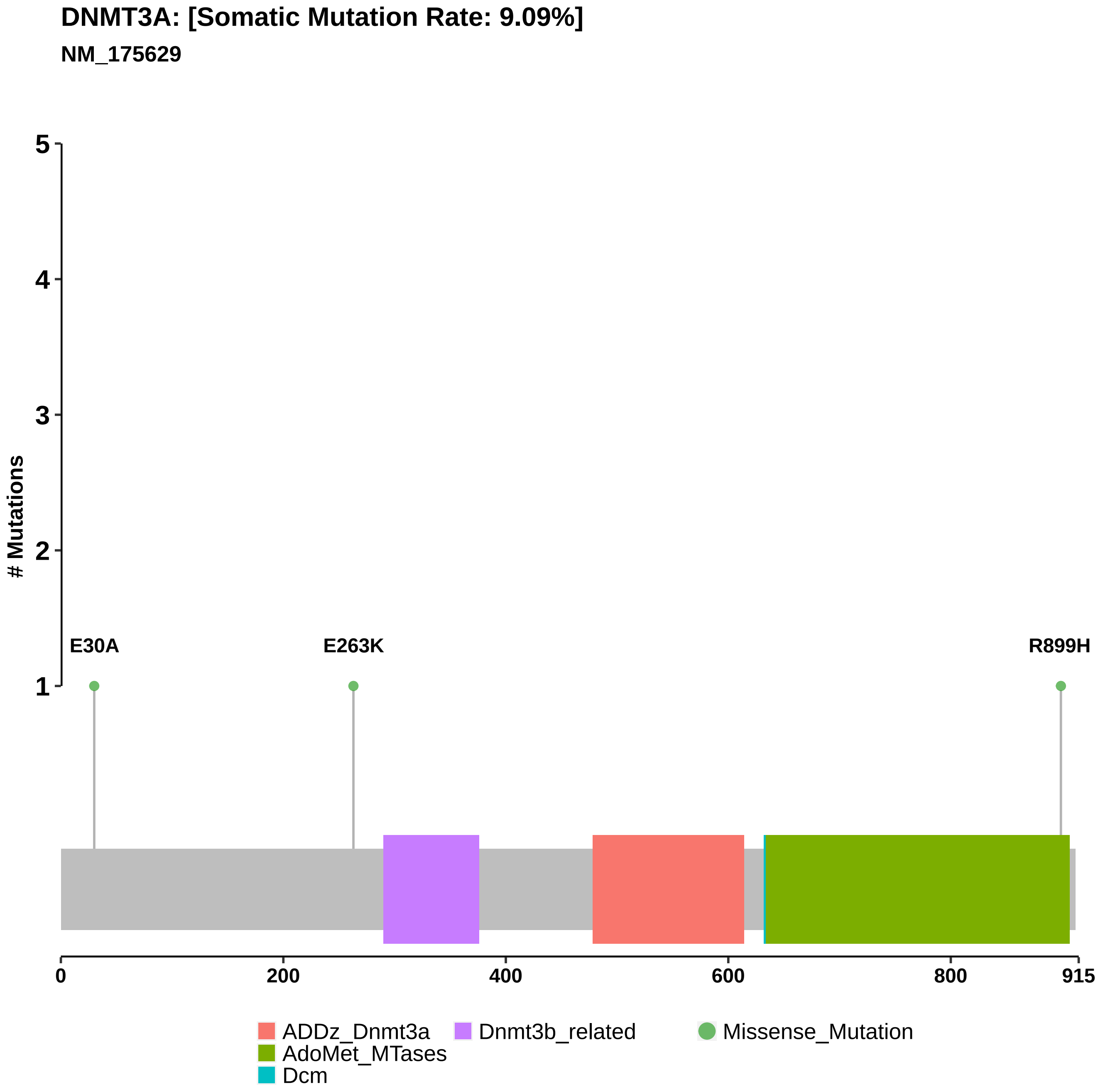
DNMT3A: [Somatic Mutation Rate: 9.09%]
NM_175629
Mutations
5
4
3
2
1
E30A
E263K
R899H
0
200
400
600
800
915
ADDz_Dnmt3a
AdoMet_MTases
Dcm
Dnmt3b_related
Missense_Mutation

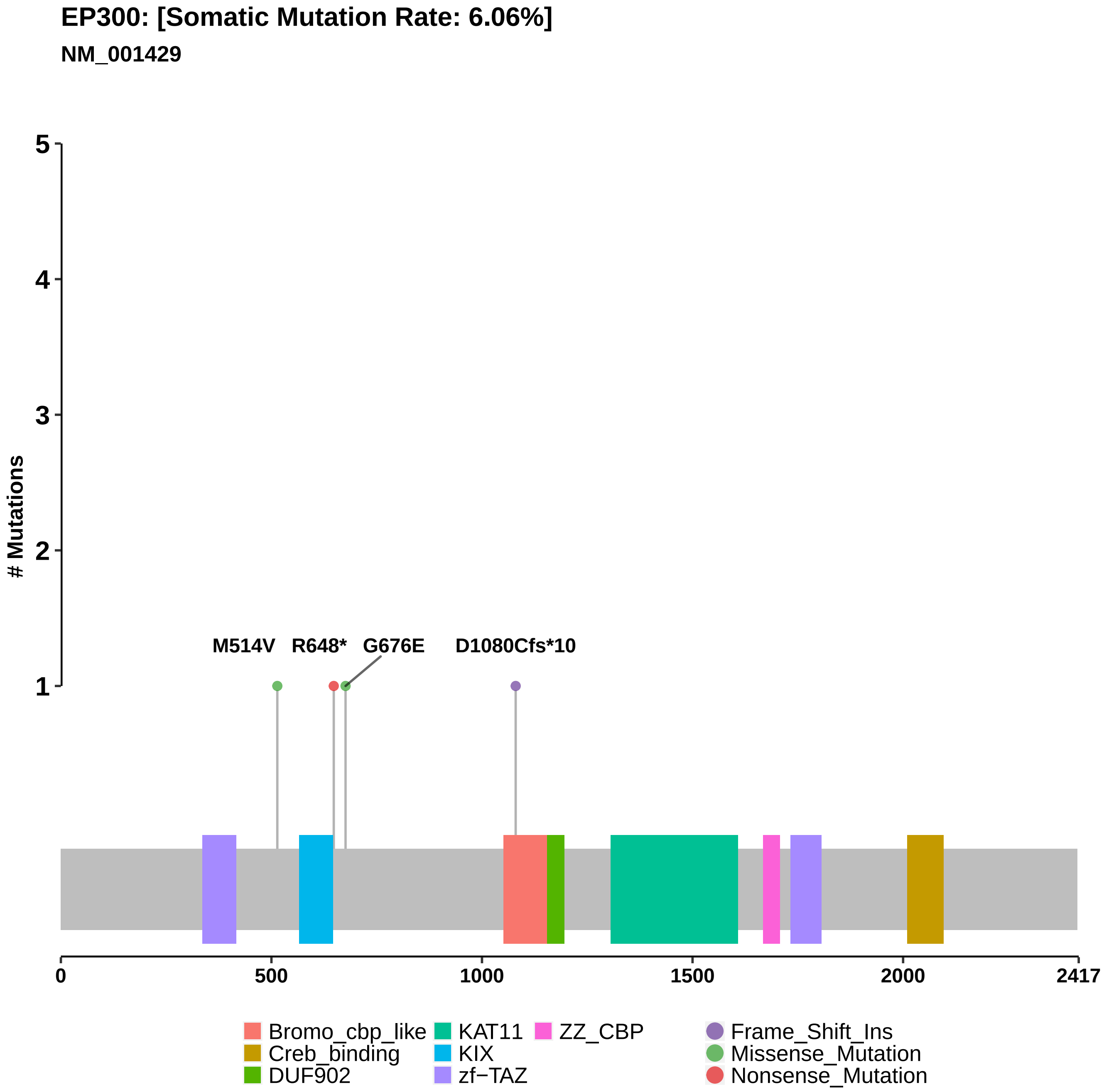
EP300: [Somatic Mutation Rate: 6.06%]
NM_001429
Mutations
1
2
3
4
5
M514V
R648*
G676E
D1080Cfs*10
0
500
1000
1500
2000
2417
Bromo_cbp_like
Creb_binding
DUF902
KAT11
KIX
zf−TAZ
ZZ_CBP
Frame_Shift_Ins
Missense_Mutation
Nonsense_Mutation

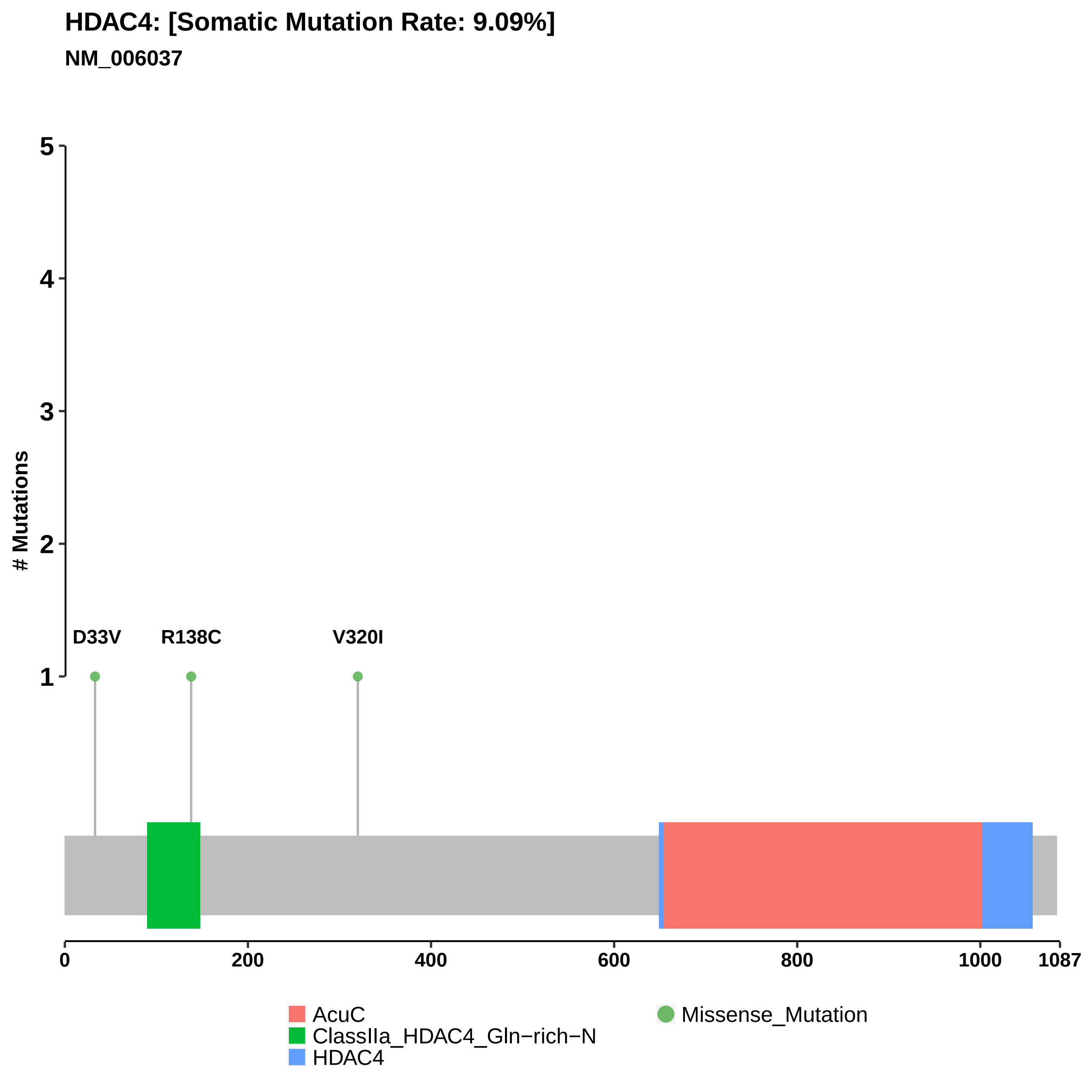
HDAC4: [Somatic Mutation Rate: 9.09%]
NM_006037
Mutations
5
4
3
2
1
D33V
R138C
V320I
0
200
400
600
800
1000
1087
AcuC
ClassIIa_HDAC4_Gln−rich−N
HDAC4
Missense_Mutation

HDAC7: [Somatic Mutation Rate: 9.09%]

NM_015401

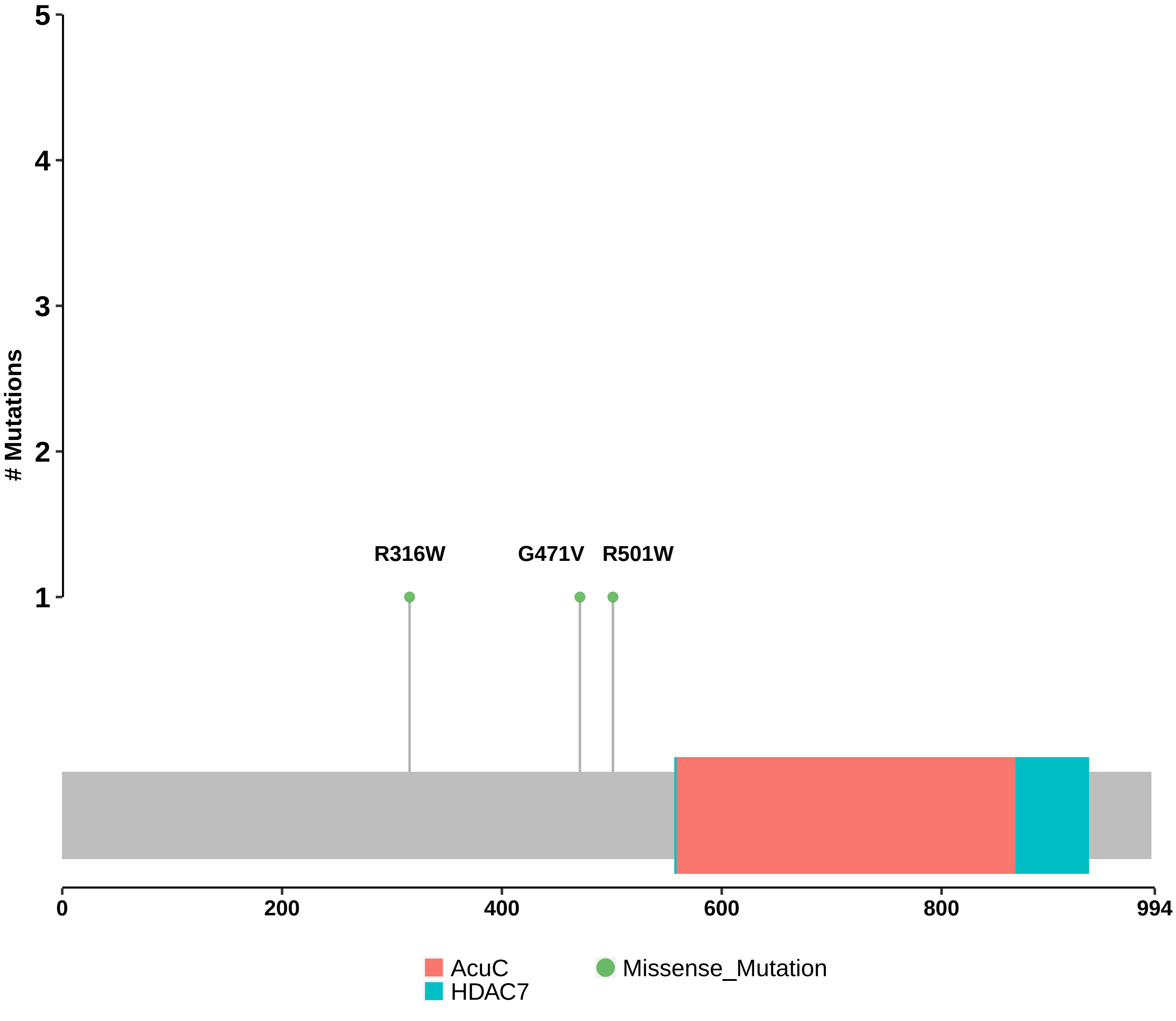

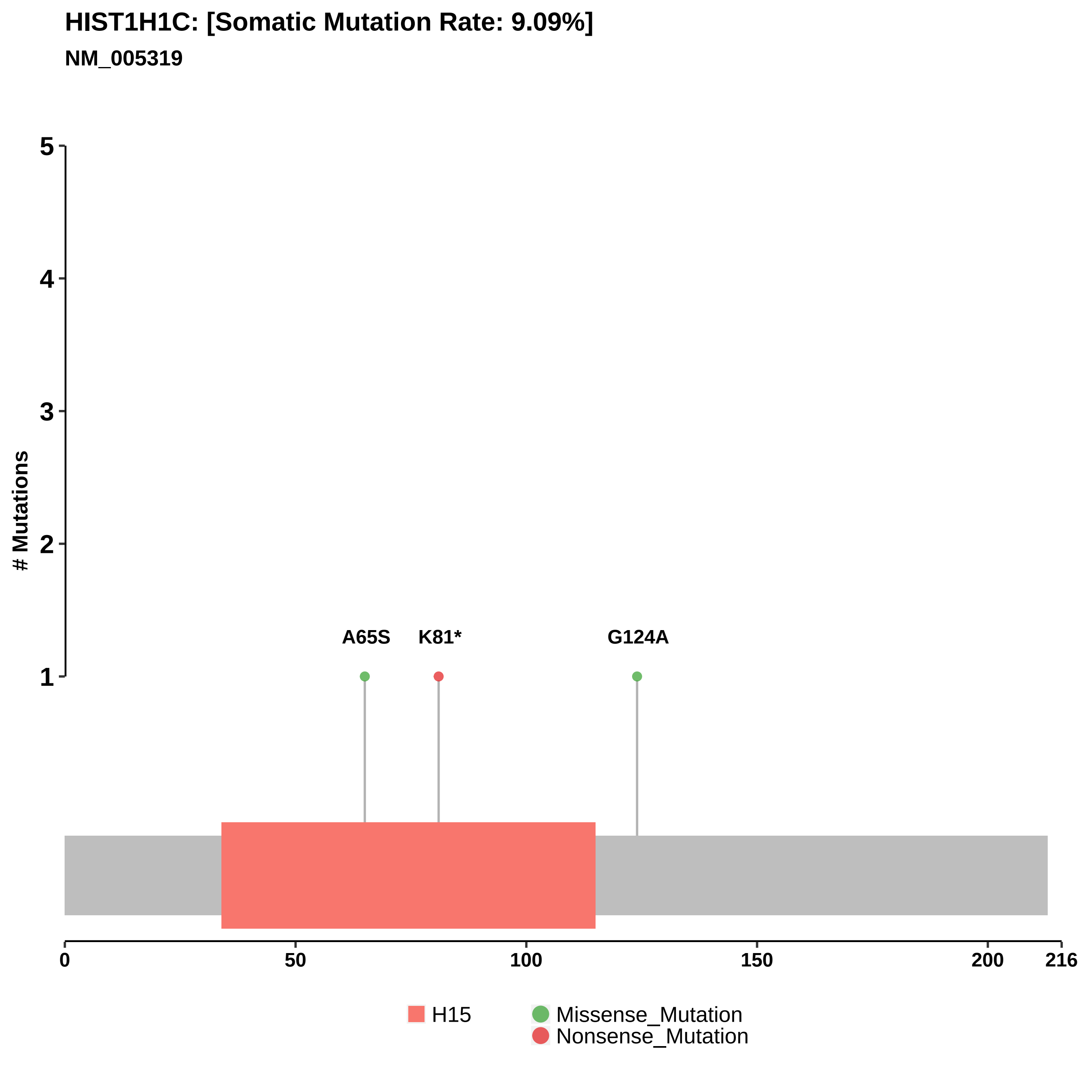
HIST1H1C: [Somatic Mutation Rate: 9.09%]
NM_005319
Mutations
5
4
3
2
1
A65S
K81*
G124A
0
50
100
150
200
216
H15
Missense_Mutation
Nonsense_Mutation

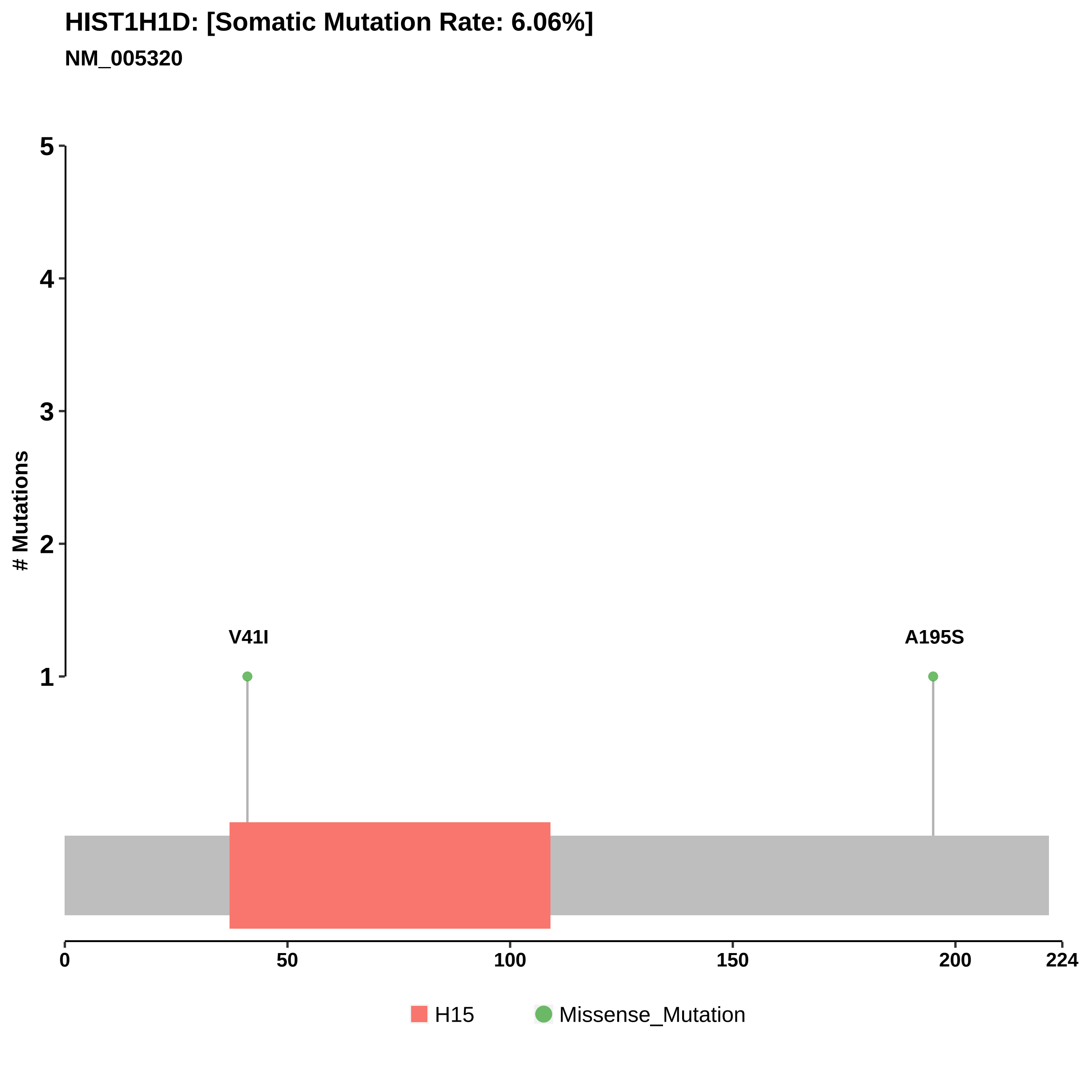
HIST1H1D: [Somatic Mutation Rate: 6.06%]
NM_005320
Mutations
5
4
3
2
1
V41I
A195S
0
50
100
150
200
224
H15
Missense_Mutation

IFIH1: [Somatic Mutation Rate: 9.09%]
NM_022168

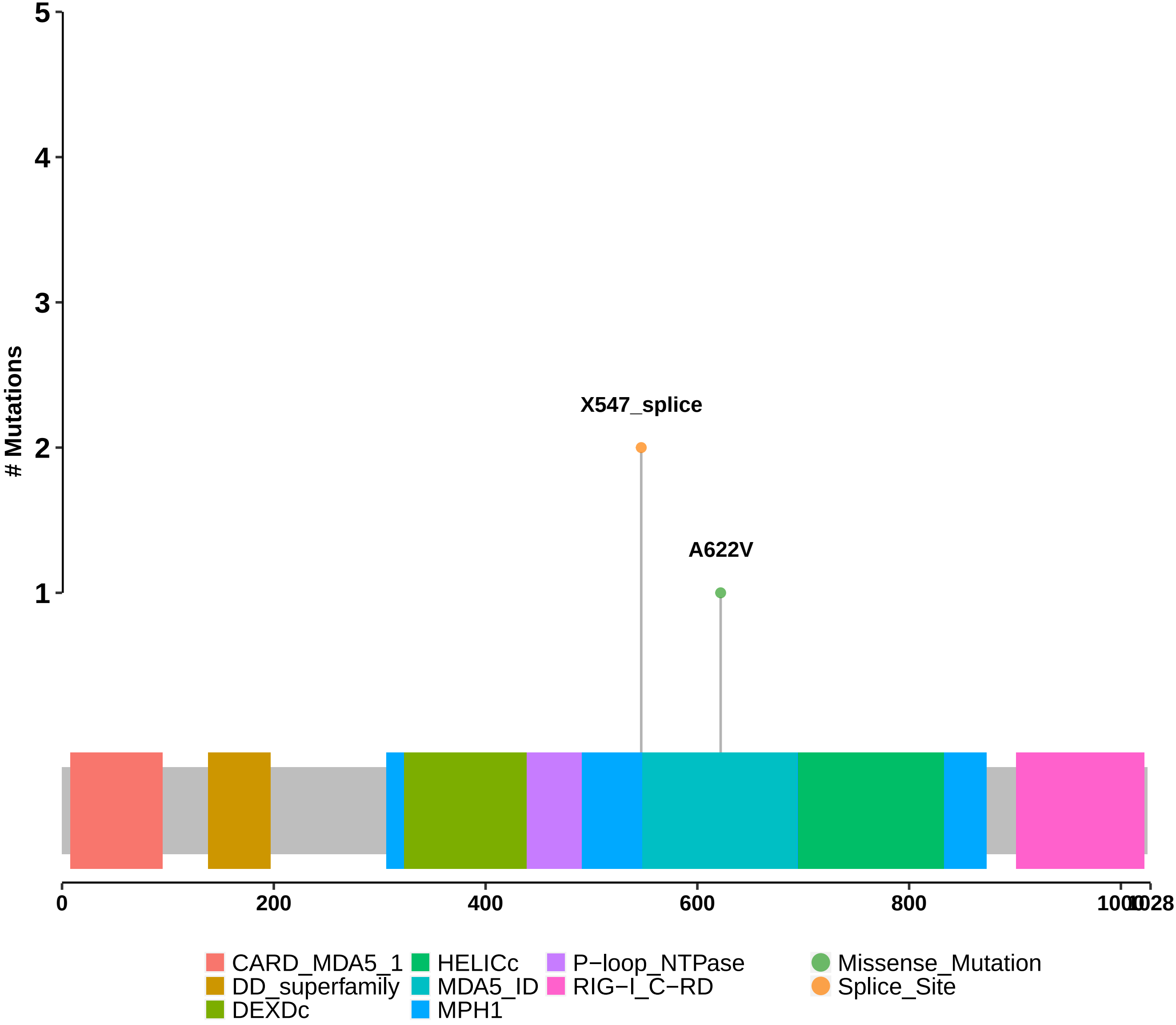

KDM5A: [Somatic Mutation Rate: 6.06%]

NM_001042603

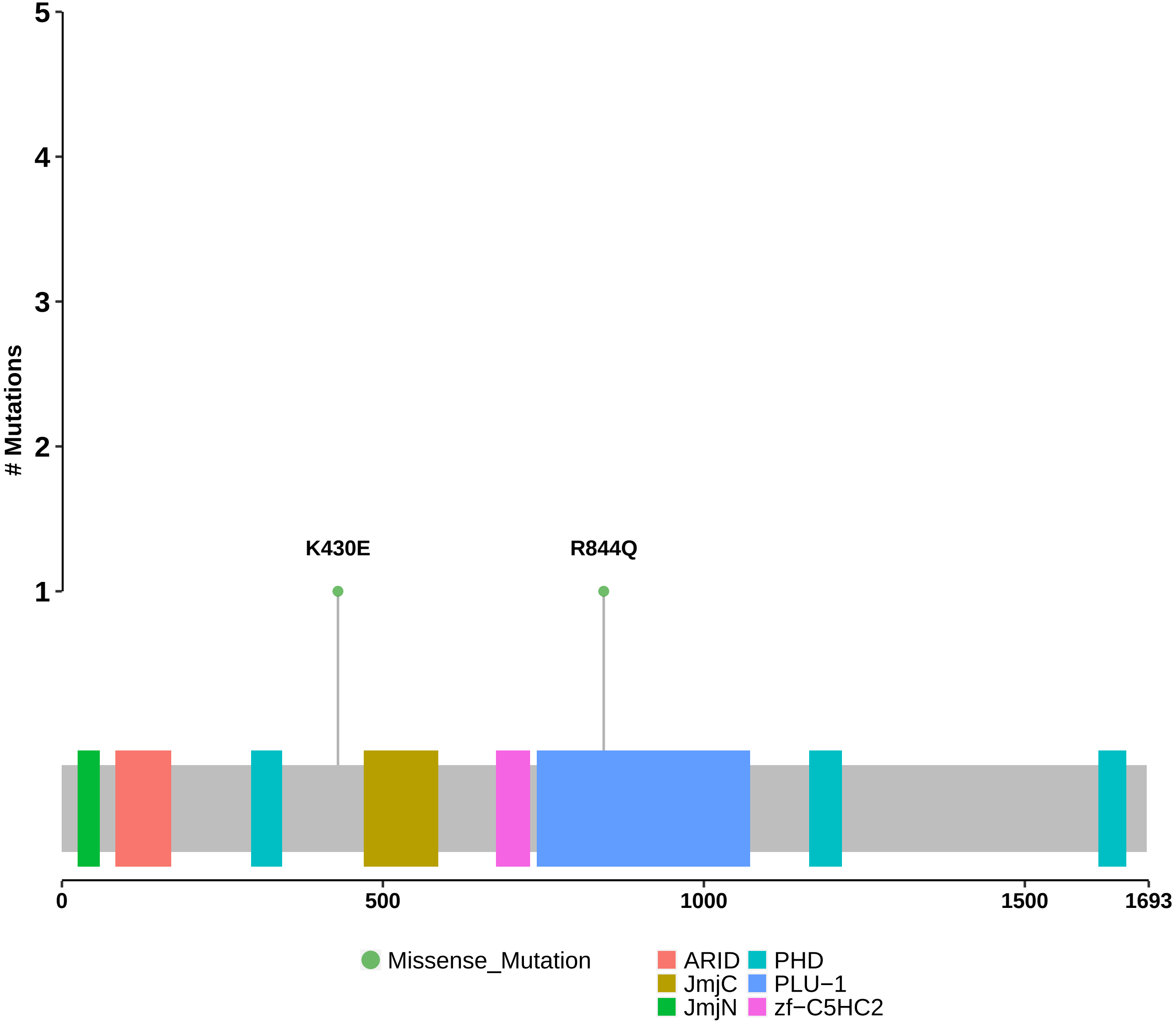

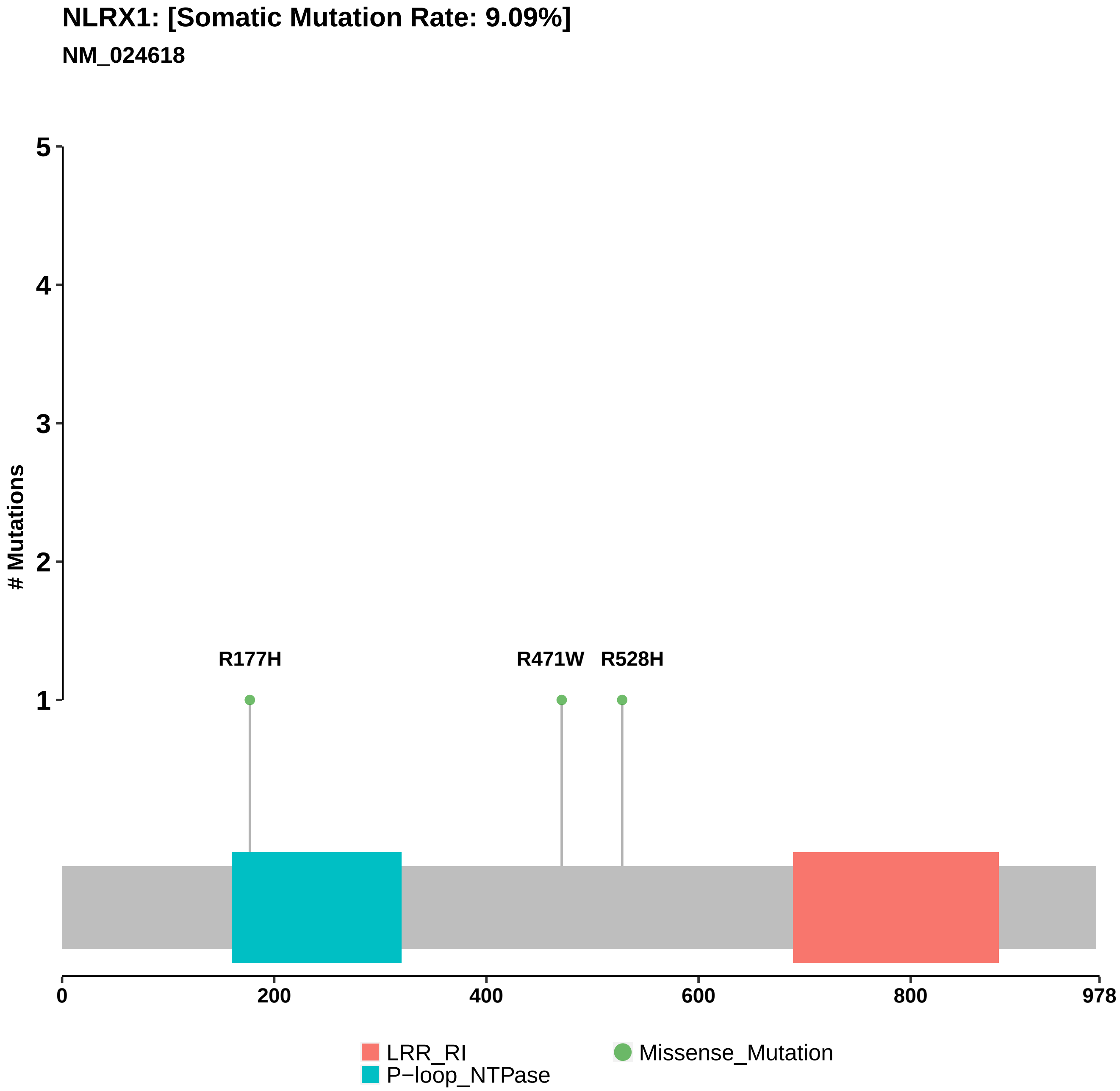

NLRX1: [Somatic Mutation Rate: 9.09%]
NM_024618
Mutations
5
4
3
2
1
R177H
R471W
R528H
0
200
400
600
800
978
LRR_RI
P−loop_NTPase
Missense_Mutation

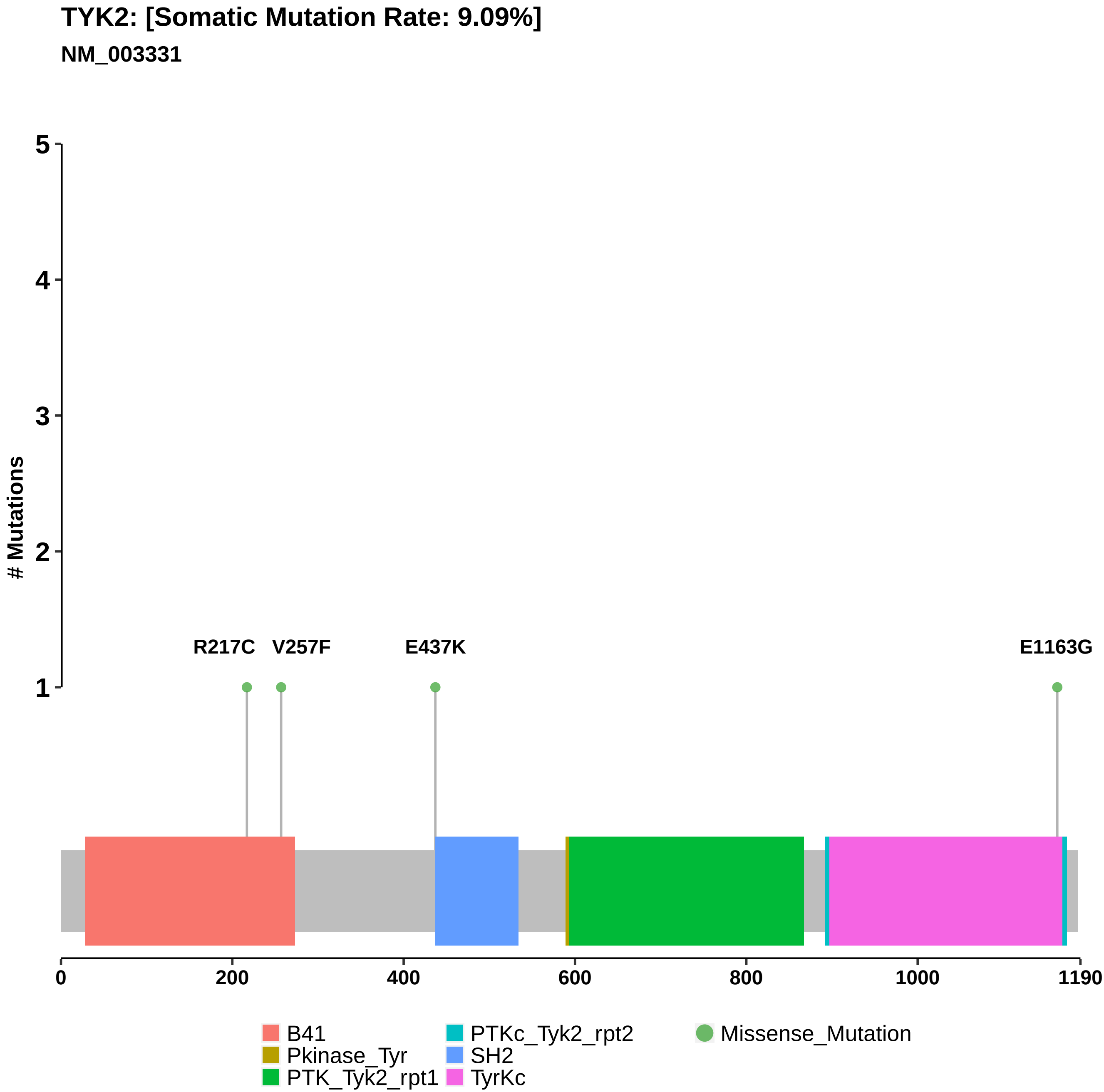
TYK2: [Somatic Mutation Rate: 9.09%]
NM_003331
Mutations
5
4
3
2
1
R217C
V257F
E437K
E1163G
0
200
400
600
800
1000
1190
B41
Pkinase_Tyr
PTK_Tyk2_rpt1
PTKc_Tyk2_rpt2
SH2
TyrKc
Missense_Mutation

WHSC1: [Somatic Mutation Rate: 6.06%]
NM_001042424

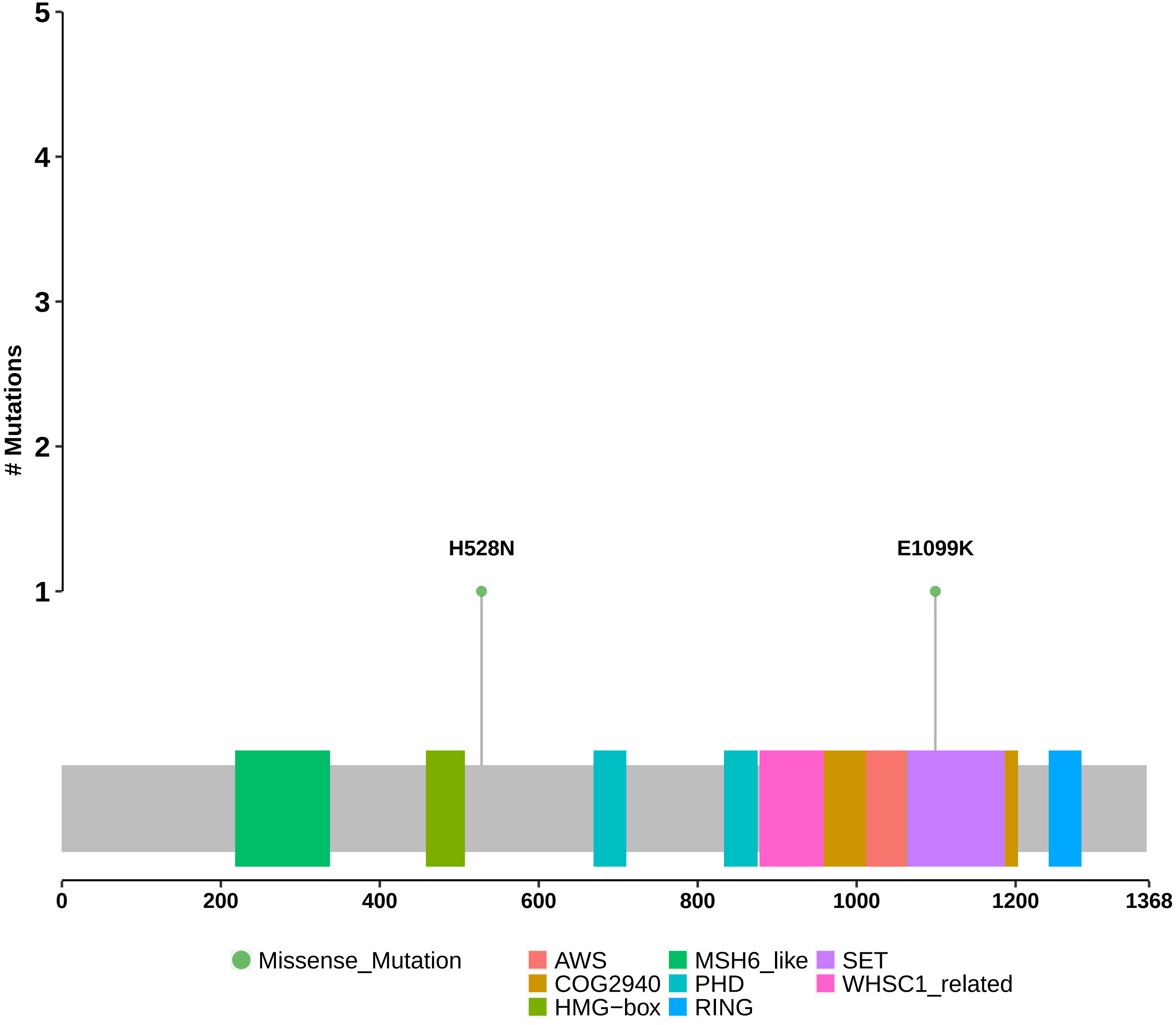

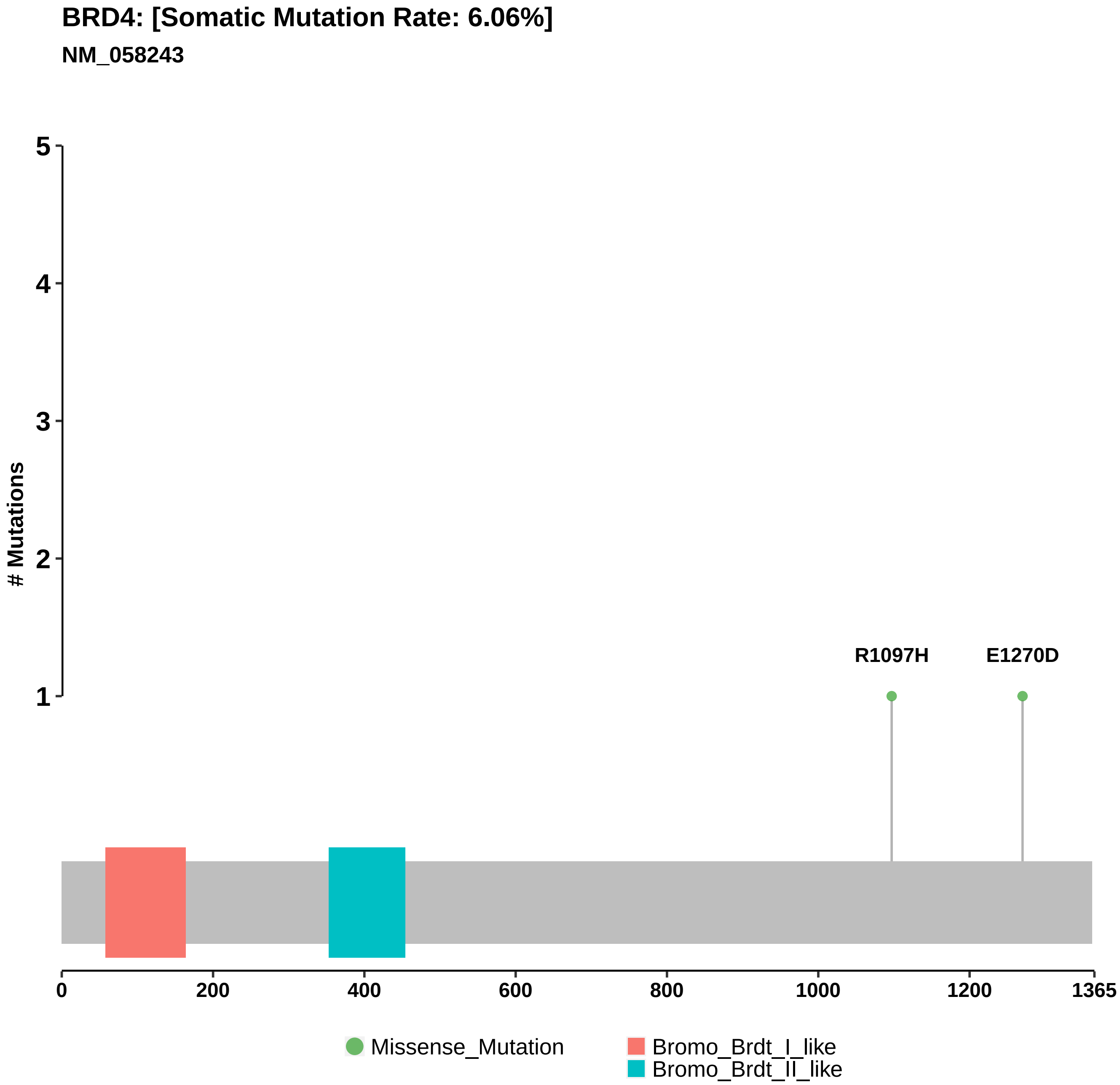
BRD4: [Somatic Mutation Rate: 6.06%]
NM_058243
Mutations
5
4
3
2
1
R1097H
E1270D
0
200
400
600
800
1000
1200
1365
Missense_Mutation
Bromo_Brdt_I_like
Bromo_Brdt_II_like
